# A Multi-Target Nitrogen-Fused Azole Drug Platform Derived from a Pyrazoline-Thiadiazole Moiety: In Vivo Antimicrobial Validation and Comprehensive Anticancer Investigation Supported by Computational Studies

**DOI:** 10.3390/pharmaceutics18040424

**Published:** 2026-03-30

**Authors:** Hagar S. El-Hema, Marwa A. Abed, Mohamed A. Hawata, Eman S. Nossier, Najla A. Altwaijry, Asmaa Saleh, Mariam Hassan, Rasha A. Hashem, Modather F. Hussein, Ahmed T. Elhendawy, Adel A.-H. Abdel-Rahman

**Affiliations:** 1 Basic Science Department (Chemistry), Thebes Higher Institute for Engineering, Thebes Academy, Maadi 11434, Cairo, Egypt; 2 Chemistry Department, Faculty of Science, Menoufia University, Shebin El-Kom 32511, Menoufia, Egypt; marwaabed.183@gmail.com (M.A.A.); drmohamedhwata@gmail.com (M.A.H.); 3 Pharmaceutical Medicinal Chemistry and Drug Design Department, Faculty of Pharmacy (Girls), Al-Azhar University, Cairo 11754, Egypt; dr.emannossier@gmail.com; 4 The National Committee of Drugs, Academy of Scientific Research and Technology, Cairo 11516, Egypt; 5 Department of Pharmaceutical Sciences, College of Pharmacy, Princess Nourah bint Abdulrahman University, P.O. Box 84428, Riyadh 11671, Saudi Arabia; naaltwaijry@pnu.edu.sa (N.A.A.); asali@pnu.edu.sa (A.S.); 6 Department of Microbiology and Immunology, Faculty of Pharmacy, Cairo University, Cairo 12613, Egypt; mariam.hassan@pharma.cu.edu.eg (M.H.); rasha.hashem@pharma.cu.edu.eg (R.A.H.); 7 Department of Microbiology and Immunology, Faculty of Pharmacy, Galala University, New Galala City 43511, Suez, Egypt; 8 Chemistry Department, College of Science, Jouf University, P.O. Box 2014, Sakaka 72341, Aljouf, Saudi Arabia; mfhussin@ju.edu.sa; 9 Physics Department, Faculty of Science, Galala University, New Galala City 43511, Suez, Egypt; ahmed.taher@gu.edu.eg

**Keywords:** pyrazoline-thiadiazole hybrids, anticancer agents, antimicrobial agents, apoptosis, MRSA, computational studies

## Abstract

**Background:** Cancer patients are highly susceptible to microbial infections due to immune suppression, necessitating therapeutic strategies that integrate anticancer efficacy with effective antimicrobial intervention. Chalcone-derived nitrogen-fused heterocycles represent a promising platform for developing multi-target agents with relevance to antimicrobial drug delivery, particularly for localized infections. **Methods:** A series of chalcone-based pyrazoline-thiadiazole nitrogen-fused azole hybrids was synthesized via thiosemicarbohydrazide-functionalized intermediates and fully characterized. Antiproliferative activity was evaluated against MCF-7, HepG-2, HeLa, and HCT-116 cell lines, alongside selectivity toward WI-38 normal fibroblasts. Antibacterial, antibiofilm, and in vivo efficacy were assessed against methicillin-resistant *Staphylococcus aureus* (MRSA USA300) and *Acinetobacter baumannii* AB5057. Mechanistic investigations included cell-cycle analysis, apoptosis assays, ERK2, RIPK3, p53, BAX/Bcl-2 quantification, DNA gyrase inhibition, molecular docking, molecular dynamics simulations, and density functional theory calculations. **Results:** Compound **13** exhibited potent cytotoxicity, particularly against MCF-7 (IC_50_ = 3.87 ± 0.2 µM), outperforming doxorubicin (IC_50_ = 4.17 ± 0.2 µM), with high selectivity indices (SI = 10.7 for MCF-7). Mechanistically, compound **13** induced G_2_/M arrest (40.16% vs. 14.15% control), increased apoptosis to 32.89%, up-regulated ERK2 (3.17-fold), RIPK3 (11.97-fold), and p53 (3.54-fold), and markedly increased the BAX/Bcl-2 ratio (~42-fold). Compounds **7** and **13** displayed bactericidal activity against MRSA and *A. baumannii* (MIC/MBC = 10 mg/mL), potent antibiofilm effects, and significant in vivo efficacy in an MRSA skin infection model. Compound **13** reduced bacterial load by ~5 log units, outperforming vancomycin. DNA gyrase inhibition (IC_50_ = 17.10 ± 0.17 µM) and computational studies supported target engagement. **Conclusions:** Pyrazoline-thiadiazole-based nitrogen-fused azole hybrids, particularly compound **13**, demonstrated quantifiable anticancer and antimicrobial efficacy with strong in vivo validation, supporting their potential as multi-target candidates relevant to antimicrobial drug delivery in infection-prone cancer patients.

## 1. Introduction

The global burden of cancer has escalated dramatically over recent decades, affecting all ages and across diverse geographic regions [1,2,3]. While genetic predisposition and hormonal fluctuations remain key contributors, modern lifestyle and environmental factors play an increasingly significant role. High-calorie, ultra-processed diets, sedentary behavior, and exposure to endocrine-disrupting chemicals have been associated with earlier onset and more aggressive disease [4,5,6,7,8,9]. New therapeutic approaches that address issues like acquired resistance in cancer patients have been made possible by recent developments in our understanding of the biology of cancer [10,11]. As a result, the creation of novel treatments that target different pathways is essential for stopping the spread of cancer and eventually raising survival rates [12].

Protein kinases such as AKT, EGFR/VEGFR, MAPK, BRAF, JAK, C-Met, and BTK regulate essential cellular processes, including growth, differentiation, and apoptosis, which makes them highly attractive therapeutic targets in oncology. These findings emphasize that small-molecule inhibitors built on heterocyclic scaffolds offer a rational and effective means of modulating dysregulated signaling pathways in cancer [13,14,15,16,17,18,19,20,21,22,23,24]. Within this framework, the ERK arm of the MAPK pathway has emerged as a critical regulator of tumorigenic behavior, controlling proliferation, survival, angiogenesis, and metastatic progression. Persistent ERK activation is a common feature across multiple cancer types, and its inhibition disrupts downstream phosphorylation cascades necessary for tumor cell persistence, thereby promoting apoptotic responses [25,26,27,28,29]. Concurrently, regulated necrotic cell death has gained significant interest as an alternative therapeutic mechanism. RIPK3 acts as a central switch between necroptosis and apoptosis, where its activation triggers MLKL-mediated membrane disruption, while its inhibition may redirect signaling toward caspase-8-dependent apoptosis. This dual role positions RIPK3 as an important complementary target alongside ERK, especially in malignancies that exhibit apoptotic resistance [30,31,32,33,34,35,36].

**Figure 1:**
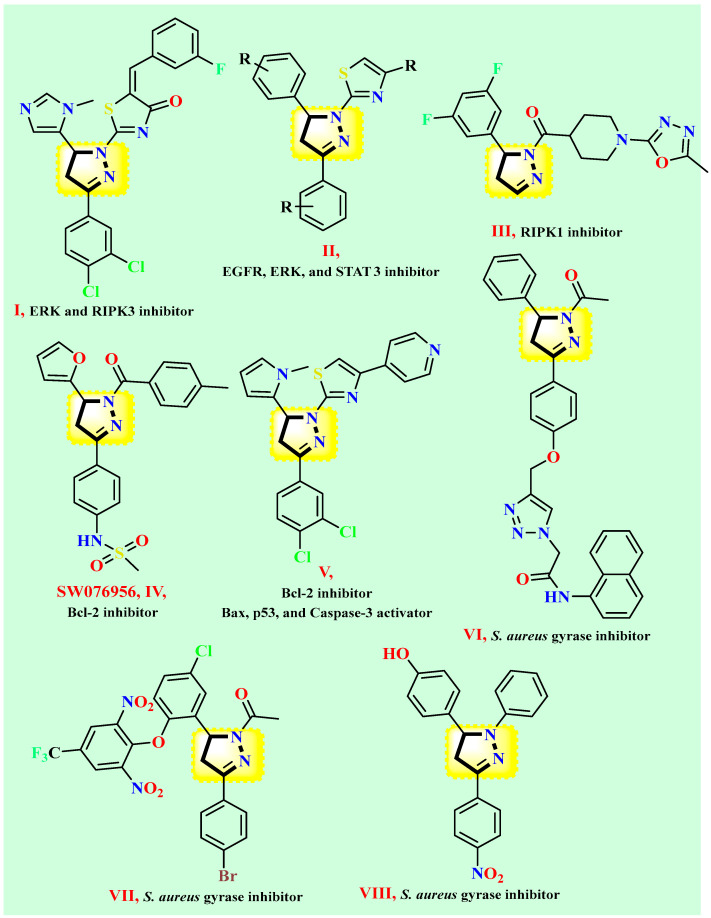
Representative pyrazoline-based derivatives with dual anticancer and antimicrobial activities targeting numerous signaling pathways.

The pyrazoline ring system is particularly well suited for modulating such kinases due to its electron-rich aromatic structure, which enables precise interactions within ATP-binding pockets [37,38,39,40,41,42]. Numerous pyrazoline derivatives have demonstrated potent ERK inhibition, and structurally related analogues show strong selectivity toward RIPK1/3 with favorable pharmacokinetic profiles [43,44,45,46]. Representative pyrazoline-based derivatives include **I** [45], which displayed promising activities towards ERK and RIPK3 in the in vitro kinase assay with significant cytotoxicity against PC-3 and MCF-7 cancer cell lines. Compound **II** inhibits the activity of anti-apoptotic proteins in carcinoma cells, such as EGFR, ERK, and STAT3, thereby promoting apoptosis [47]. Additionally, **III** is a particular and effective RIPK1 inhibitor that suppresses macrophage-mediated adaptive immunological tolerance in pancreatic cancer [48]. In the area of Bcl-2 inhibition as an additional channel in pyrazoline-based anticancer therapy, compound **IV** (SW076956) emerged as a strong inhibitor, targeting the Bcl-2 protein involved in apoptosis [49]. The 1,3,5-trisubstituted-1*H*-pyrazole derivative **V** potentially inhibited Bcl-2 and activated pro-apoptotic proteins Bax, p53, and Caspase-3 [50] (Figure 1). On the other hand, Kumar et al. reported efficient potency of **VI** against *S. aureus* targeting DNA gyrase [51]. With an IC_50_ of 0.125 μg/mL, pyrazoline derivative **VII**, which contains the dinitrobenzotrifluoride moiety, revealed the highest inhibitory effect against *S. aureus* DNA gyrase [52]. Pyrazoline derivative **VIII**, which targets DNA gyrase, also exhibited good action against *S. aureus*, with a mean inhibition of 15.2 ± 0.04 mm at 100 μg/mL [53] (Figure 1).

**Figure 2:**
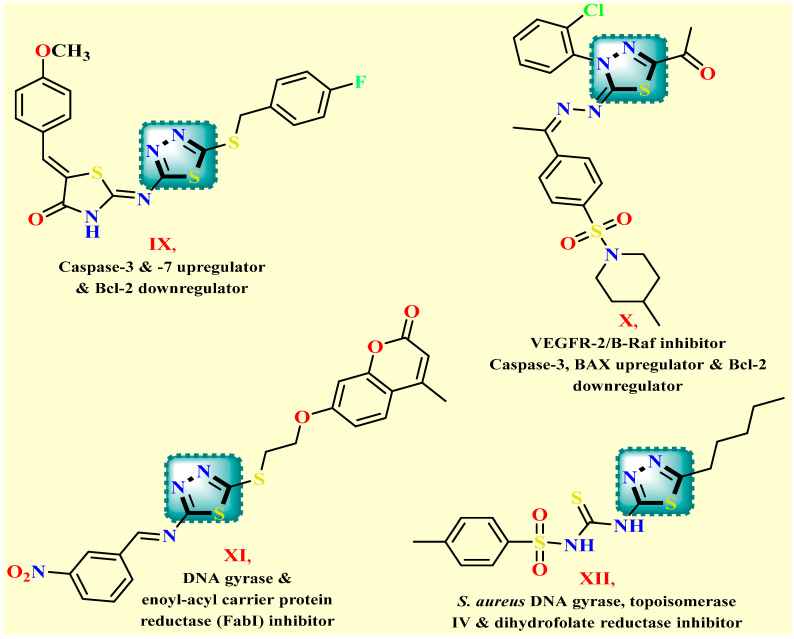
Schematic illustrating the dual anticancer and antimicrobial activities of 1,3,4-thiadiazole derivatives via different pathways.

From an alternative perspective, 1,3,4-thiadiazole derivatives are emphasized because of their diverse biological actions, which include anticancer, anti-inflammatory, antibacterial, anticonvulsant, antiviral, antituberculosis, and antioxidant [54,55,56,57,58,59,60]. Compound **IX** causes cancer cells to produce ROS, which leads to nuclear disintegration, chromosomal condensation, and cell shrinkage—all of which indicate that the cancer cells are undergoing apoptosis. Compound **IX** further demonstrates the apoptotic process by dose-dependently upregulating caspase-3 and -7 and downregulating Bcl-2 protein. When compared to the conventional anticancer medication sorafenib, compound **IX** exhibits notable anti-angiogenic activities in ACHN cells [61]. Compound **X** effectively caused apoptosis and G2/M phase arrest, which were linked to caspase-3 activation and Bax/Bcl-2 expression regulation. Additionally, compound **X** displays promise as a dual VEGFR-2/B-Raf inhibitor with the ability to progress in the treatment of breast cancer [62]. By inhibiting DNA gyrase and enoyl-acyl carrier protein reductase (FabI), coumarin-thiadiazole hybrid **XI** may be a promising lead compound for the creation of a novel antibacterial medication against MDR infections [63]. With IC_50_ values of 1.22, 53.78, and 0.23, respectively, **XII** was a potent inhibitor of *S. aureus* enzymes, including DNA gyrase, DNA topoisomerase IV (Topo IV), and dihydrofolate reductase [64] (Figure 2).

Cancer patients are particularly susceptible to opportunistic bacterial infections due to immunosuppression induced by the disease or chemotherapy. Among these, Methicillin-resistant *Staphylococcus aureus* (MRSA) represents a major clinical concern because of its high virulence and multidrug resistance [65]. Pyrazole/pyrazoline derivatives have demonstrated promising antibacterial activity against MRSA in several studies [66,67]. Therefore, developing compounds capable of simultaneously targeting cancer cells and MRSA offers a strategic advantage: it may improve therapeutic outcomes by addressing both tumor proliferation and bacterial infections in immunocompromised patients, while potentially reducing treatment complexity and overall healthcare costs [68].

**Figure 3:**
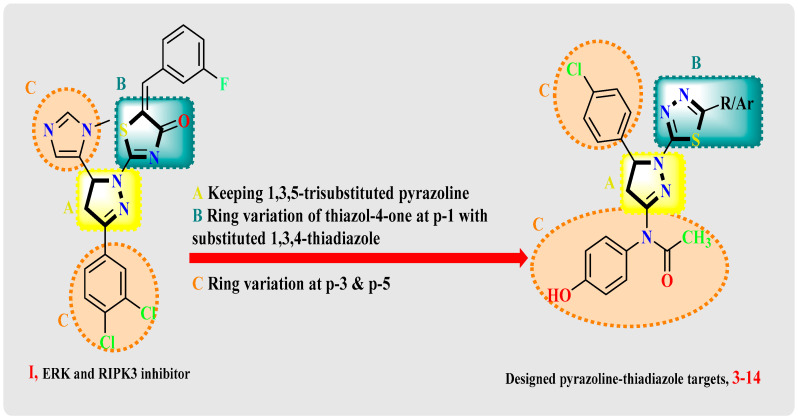
Rational design strategy of pyrazoline-thiadiazole-based derivatives **3**–**14** as cytotoxic agents targeting ERK and RIPK3, inspired by lead **I** and against methicillin-resistant *Staphylococcus aureus* (MRSA) targeting *S. aureus* DNA gyrase.

In light of these insights, and guided by lead compound **I** [45] alongside diverse drug-design strategies including keeping of 1,3,5-pyrazoline core, ring variation in thiazol-4-one at p-1 with substituted 1,3,4-thiadiazole and ring variation at p-3 & p-5, we designed and synthesized a new sequence of 2-1*H*-pyrazoline-thiadiazole-based derivatives **3**–**14** (Figure 3), confirming their structures via IR, ^1^H NMR, ^13^C NMR, and mass spectrometry. All derivatives were assessed for their antiproliferative activity against HeLa, HepG-2, and MCF-7 cells, including selectivity toward normal WI-38 fibroblasts. The most active derivative will be further investigated for its effects on BAX, Bcl-2, p53 expression, apoptosis induction, cell cycle progression, and ERK/RIPK3 signaling. The outcomes of this study are expected to provide molecular insights into the dual regulatory role of these compounds in cancer cell death and survival and to establish a foundation for the development of multifunctional pyrazole-based anticancer agents capable of overcoming chemoresistance and improving therapeutic selectivity. Furthermore, these compounds were evaluated in vitro for their antibacterial activity against methicillin-resistant *Staphylococcus aureus* (MRSA, USA300) as a representative Gram-positive strain and *Acinetobacter baumannii* AB5057 as a representative Gram-negative strain. The pyrazoline-thiadiazole-based derivatives demonstrated a strong potency, were subjected to biofilm formation, in vivo *MRSA* skin infection model, and in vitro *S. aureus* DNA gyrase inhibitory assessment. To enhance understanding and provide deeper insights, docking simulations, Molecular dynamics, and DFT were also conducted.

## 2. Results and Discussion

### 2.1. Chemistry

**Scheme 1:**
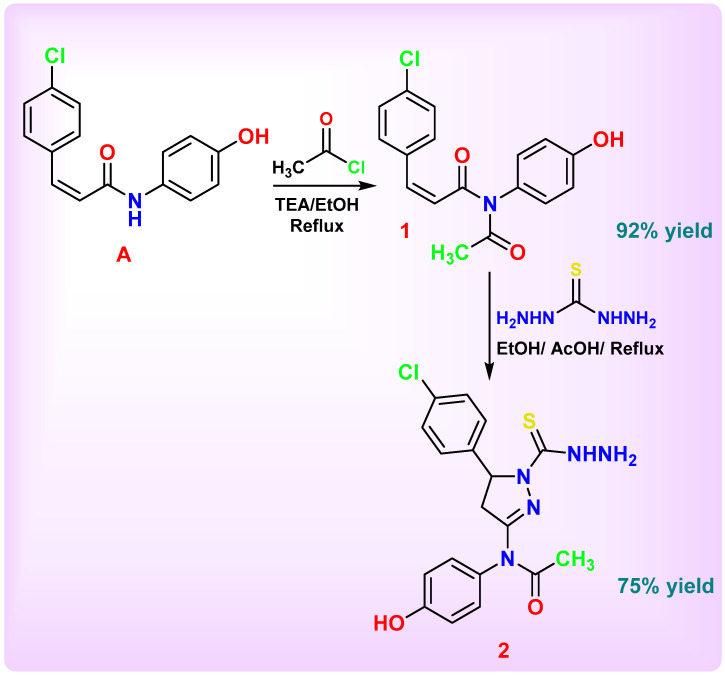
Synthesis of *N*-acetyl α,β-unsaturated chalcone and thiohydrazide pyrazoline **1** and **2**-based derivatives.

**Scheme 2:**
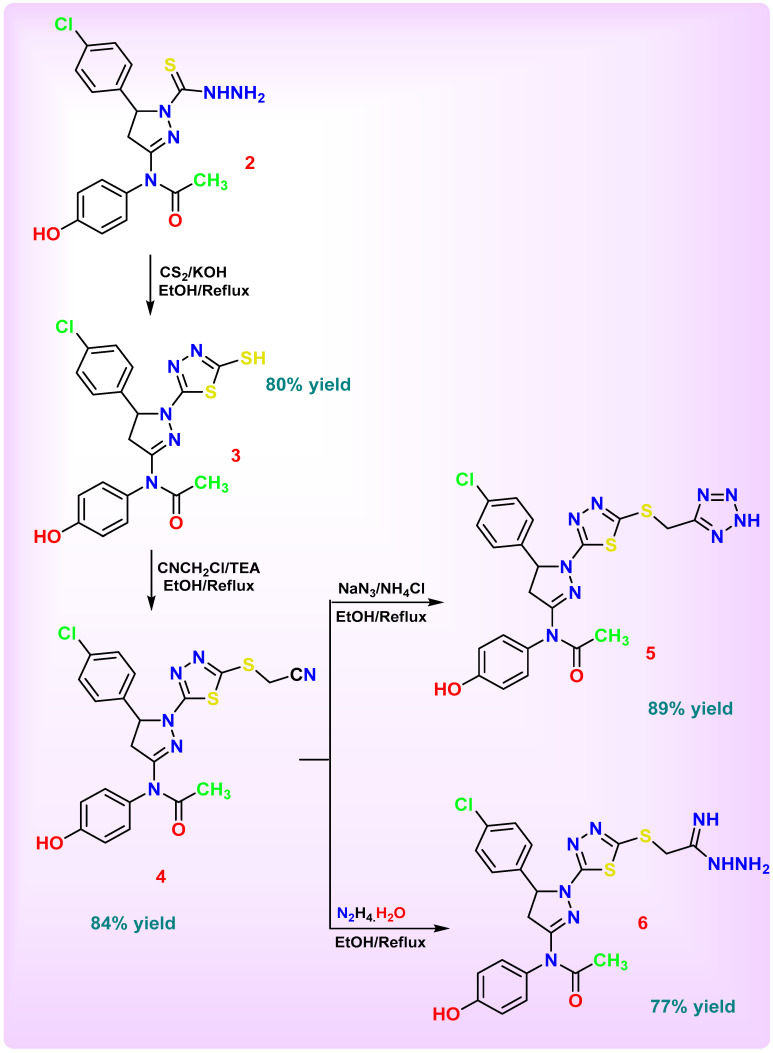
Synthesis of 2-1*H*-pyrazoline-thiadiazole-based derivatives **3**–**6**.

**Scheme 3:**
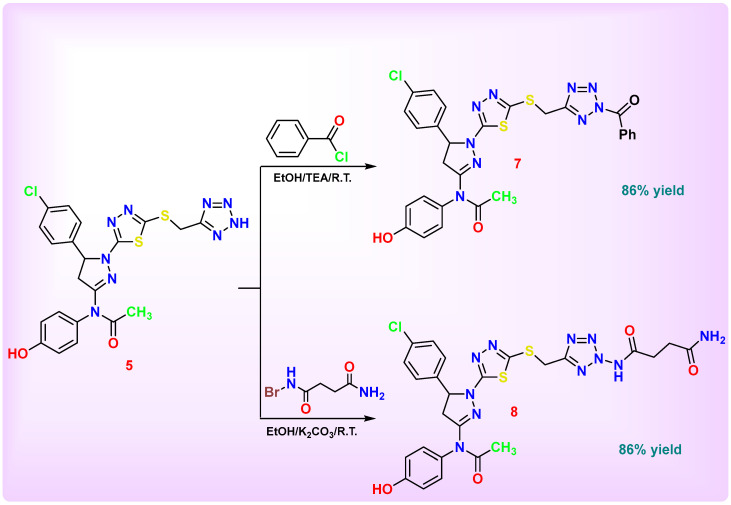
Synthesis of pyrazoline-thiadiazole-based derivatives **7** and **8**.

**Scheme 4:**
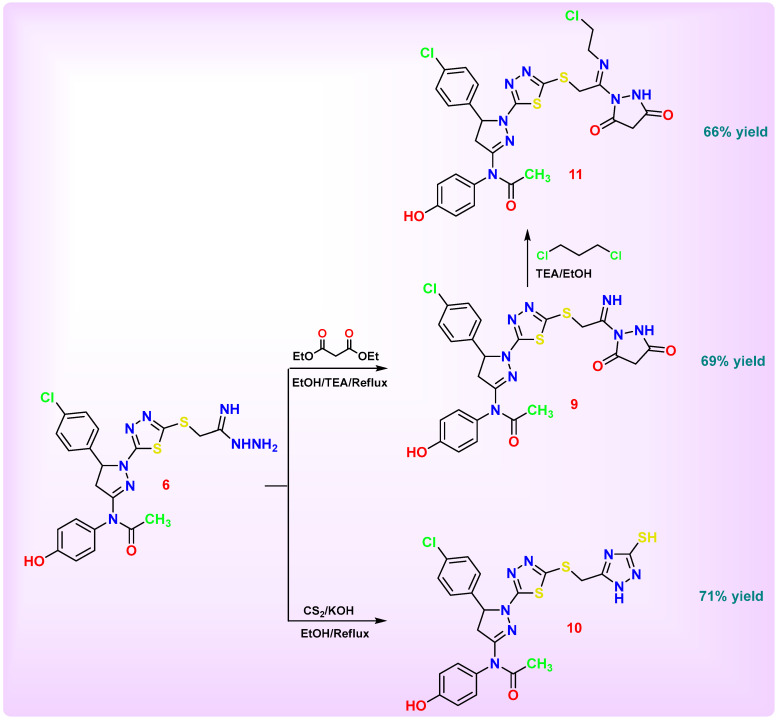
Synthesis of 2-1*H*-pyrazoline-thiadiazole-based derivatives **9**–**11**.

**Scheme 5:**
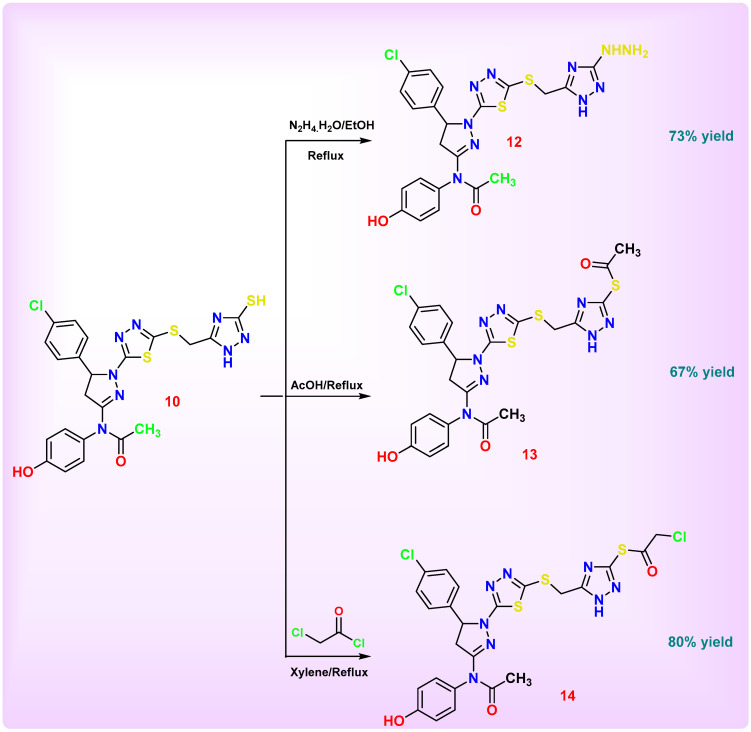
Synthesis of 2-1*H*-pyrazoline-thiadiazole-based derivatives **12**–**14**.

The main objective of this work was to design and synthesize a novel series of nitrogen-rich heterocyclic derivatives based on a key thiadiazolyl-pyrazoline scaffold for subsequent biological evaluation. Scheme 1, Scheme 2, Scheme 3, Scheme 4 and Scheme 5 illustrate the synthetic pathways for the newly designed chlorophenyl-thiadiazol-1*H*-pyrazol-hydroxyphenyl carbamic chloride derivatives **1**–**14**.

In Scheme 1, the key intermediate (*Z*)-3-(4-chlorophenyl)-*N*-(4-hydroxyphenyl)acrylamide (**A**) was first synthesized according to the reported procedure [69]. Compound **A** was then treated with chloroacetly chloride, affording the *N*-acetyl α,β-unsaturated chalcone derivative (**1**). Subsequent reaction of compound **1** with thiocarbazide yielded the hydrazine carbonothioyl 4,5-dihydro-1*H*-pyrazoline derivative **2**. This compound **2** represents the target intermediate, from which a new series of derivatives was further developed and evaluated for their biological activities.

The nucleophilic nitrogen of the NH group in compound **A** attacked the electrophilic carbonyl carbon of acetyl chloride, resulting in the formation of an acetyl derivative. This reaction introduces a reactive –COCH_3_ functionality, converting the amide into the corresponding acetyl derivative **1.** The IR spectrum of compound **1** displayed characteristic absorption bands at 3447 cm^−1^ due to the hydroxyl (O–H) group, at 3054 and 3093 cm^−1^ corresponding to aromatic C–H stretching vibrations, at 2984 cm^−1^ corresponding to aliphatic C–H stretching vibrations. A strong absorption band at 1734 cm^−1^ was attributed specifically to the C=O group of the acyl chloride, while the band at 1627 cm^−1^ appeared as a broad peak, resulting from the overlapping of amide C=O and aromatic C=C stretching vibrations. The ^1^H NMR spectrum exhibited singlet signal at δ 2.66 ppm due to methyl protons and multiplet signals in the range δ 7.18–8.05 ppm integrating for ten protons, corresponding to eight aromatic protons and two olefinic protons of the chalcone moiety. A broad singlet at δ 9.64 ppm was assigned to the hydroxyl (OH) proton. The ^13^C NMR spectrum showed signals at δ 26.0 ppm due to methyl carbon, 115.5 ppm corresponding to the chalcone carbon adjacent to the carbonyl group, multiple aromatic carbons resonating between δ 118.1 and 131.6 ppm (11C), and a signal at δ 140.0 ppm assigned to the substituted aromatic carbon bearing chlorine. The carbonyl carbons appeared at δ 151.1 and 161.0 ppm, corresponding to acyl chloride and amide groups, respectively, while the phenolic carbon (Ar–C–OH) resonated at δ 153.7 ppm.

Transformation involved the nucleophilic attack of the hydrazine moiety on the chalcone double bond (CH=CH) followed by intramolecular cyclization and thionation, resulting in the generation of a pyrazoline nucleus bearing a thiohydrazide substituent **2**. Consequently, the characteristic absorptions of CH=CH (chalcone) and C=O (amide) disappeared, confirming the loss of conjugated enone and amide functionalities. New signals corresponding to C=N (pyrazoline), C=S (thiocarbonyl), and NHNH_2_ (hydrazine) appeared, clearly indicating the successful ring closure and thiohydrazide formation. The IR spectrum of compound **2** revealed absorption bands at 3297 and 3160 cm^−1^ due to NH_2_ and NH stretching vibrations, while the C=N stretching of the newly formed pyrazoline ring appeared at 1668 cm^−1^. A distinct absorption at 1106 cm^−1^ was assigned to the C=S group. The ^1^H NMR spectrum displayed a doublet at δ 2.05 ppm (2H) assigned to the CH_2_ group of the pyrazoline ring, and a triplet at δ 4.58 ppm (1H) attributed to the CH proton of the same ring. A singlet at δ 3.61 ppm and another at δ 8.52 ppm (both D_2_O-exchangeable) were assigned to NH_2_ and NH groups. The ^13^C NMR spectrum exhibited characteristic signals at δ 33.9 and 59.1 ppm corresponding to CH_2_ and CH carbons of the pyrazoline ring, respectively. The imine carbon (C=N, pyrazoline) appeared at δ 140.8 ppm, while thiocarbonyl (C=S) carbon was observed at δ 183.1 ppm, respectively.

In Scheme 2, thiohydrazide pyrazoline derivative **2** was first treated with carbon disulfide in the presence of a base to furnish the thiadiazole-2-thiol derivative **3**. Compound **3** was subsequently alkylated with chloroacetonitrile, affording thiadiazol-thio acetonitrile derivative **4**. The latter intermediate **4** was then elaborated via two divergent pathways: reaction with sodium azide produced the corresponding tetrazole derivative **5**, whereas treatment with hydrazine hydrate gave the acetamido-hydrazide derivative **6**. These newly obtained scaffolds (**3**–**6**) served as key building blocks for the subsequent generation of a small library of derivatives, which were advanced for biological evaluation.

Compound **3** was obtained through intramolecular cyclization and sulfur insertion, where the thiohydrazide group (-C=S-NHNH_2_) was replaced by a thiadiazole-2-thiol ring, as confirmed by the disappearance of the C=S and NHNH_2_ bands and the appearance of two C=N bonds within the thiadiazole ring and an external S–H group attached to it. The IR spectrum of compound **3** displayed an absorption distinct band at 2560 cm^−1^ confirmed the presence of the thiol (S-H) group, while the broad absorption at 1638 cm^−1^ was assigned to overlapping two C=N (pyrazoline and thiadiazole) and aromatic C=C stretching vibrations, supporting the formation of the fused heterocyclic system. The ^1^H NMR spectrum exhibited a singlet at δ 12.61 ppm corresponding to the S-H proton of the thiadiazole ring. The ^13^C NMR spectrum showed two additional characteristic signals at δ 173.4 and 180.9 ppm were attributed to C=N carbons within the newly formed thiadiazole ring (C=N–N and C=N-SH), confirming the successful ring formation. The mass spectrum exhibited a molecular ion peak at *m*/*z* 466 (M^+^) corresponding to the molecular formula and a base peak at *m*/*z* 364, supporting its proposed structure.

Compound **4** was obtained when compound **3**’s thiol group underwent nucleophilic substitution with chloroacetonitrile, leading to the formation of a new thioether linkage (-S-CH_2_-CN). The disappearance of the SH band and the appearance of characteristic CN and SCH_2_ absorptions in the spectra confirmed this transformation. The IR spectrum of compound **4** exhibited a sharp band at 2208 cm^−1^ indicated the presence of a nitrile (C≡N) group, confirming successful substitution by chloroacetonitrile. The ^1^H NMR spectrum showed a singlet at δ 3.78 ppm assigned to the new methylene group (SCH_2_-CN). The ^13^C NMR spectrum exhibited signals at δ 18.1 ppm for the methylene carbon of SCH_2_-CN, while the nitrile carbon (C≡N) appeared at δ 118.0 ppm.

Compound **5** was obtained via a cycloaddition reaction, resulting in the formation of a tetrazole ring derivative. During this transformation, the cyano group was completely consumed, giving rise to new nitrogen-nitrogen and carbon–nitrogen bonds characteristic of the tetrazole moiety, along with the appearance of an amino proton signal. The IR spectrum exhibited absorption bands at 3216 cm^−1^ (ν N-H), while the overlapping carbon-nitrogen absorptions appeared as a broad band at 1629 cm^−1^, confirming conjugation within the heteroaromatic system. Additionally, a distinct absorption at 1504 cm^−1^ corresponded to the nitrogen–nitrogen stretching of the newly formed tetrazole ring. The ^1^H NMR spectrum showed a characteristic singlet at δ 4.52 of methylene linked to sulfur, shifted downfield from δ 3.87 in compound **4**, indicating the conversion of the methylene-cyano group into a methylene-tetrazole unit. A D_2_O-exchangeable singlet at δ 12.42 ppm (s, 1H) corresponded to the amino proton. The ^13^C NMR spectrum displayed resonances at δ 29.7 (methylene linked to sulfur), shifted from δ 18.1 in compound **4** due to the conversion from the methylene-cyano group to the methylene-tetrazole unit, and at δ 162.3 (carbon-nitrogen of tetrazole), confirming successful ring formation. Mass spectrometric analysis revealed a molecular ion peak at *m*/*z* = 548, consistent with the proposed molecular formula, and a base peak at *m*/*z* = 492.

Compound **6** was synthesized through a nucleophilic addition reaction, in which the cyano group was transformed into an acetamido-hydrazide functionality containing a carbon–nitrogen double bond linked to a hydrazide fragment (C=NH–NH–NH_2_). This transformation proceeded via nucleophilic attack of hydrazine on the electrophilic carbon of the nitrile group, followed by rearrangement to yield the new amidine-hydrazide linkage. The IR spectrum exhibited characteristic absorption bands at 3263 and 3200 cm^−1^ (ν N–H and N–H_2_), confirming the formation of the hydrazide moiety, while the carbon–nitrogen double bond absorption appeared at 1716 cm^−1^. The ^1^H NMR spectrum showed resonances corresponding to the newly formed hydrazide protons at δ 3.39 (s, 2H, amino protons, D_2_O exchangeable), δ 4.27 (s, 1H, secondary amine proton, D_2_O exchangeable), and δ 9.17 (s, 1H, imine proton, D_2_O exchangeable). The ^13^C NMR spectrum supported the proposed structure, showing a signal at δ 159.6 assigned to the carbon-nitrogen double bond (C=NH), confirming the successful formation of the acetamido-hydrazide group.

Compound **7** was synthesized via acylation reaction, during which the tetrazole NH proton was replaced by the acyl group to form a phenyl(2*H*-tetrazol-2-yl)methanone derivative. This transformation involved nucleophilic attack of the tetrazole nitrogen on the carbonyl carbon of benzoyl chloride, resulting in the formation of a new C=O–Ph linkage. The disappearance of the tetrazole NH signal and the appearance of a new acyl carbonyl confirmed the successful acylation. The IR spectrum exhibited new absorption band at 1674 cm^−1^, corresponding to the acyl group. In the ^1^H NMR spectrum, the aromatic protons of the introduced Ph–C=O group appeared in the aromatic region and overlapped with the existing aromatic proton signals, resulting in multiplet signals rather than distinct new peaks; however, the tetrazole NH signal at δ 12.4 ppm observed in compound **5** disappeared. Carbonyl carbon of the acyl groups was detected at δ 168.4 (C=O–Ph) in ^13^C-NMR spectrum.

Compound **8** was synthesized through a substitution–acylation reaction. In this transformation, the NH group of the tetrazole ring was substituted by a 4-(l2-azanyl)-4-oxobutanamide moiety, with the concurrent liberation of hydrogen bromide (HBr). The introduction of this acylamide chain significantly altered the electronic environment of the tetrazole nucleus, confirming the replacement of the tetrazole NH proton by a succinimide-derived amide linkage. The IR spectrum displayed a broad absorption band at 3421 cm^−1^, corresponding to the overlapping stretching vibrations of OH and NH_2_ groups. Additional characteristic peaks appeared at 3160 cm^−1^ due to NH, two strong carbonyl bands were observed at 1747 and 1704 cm^−1^, assigned to the newly introduced amide and acyl carbonyls. The ^1^H-NMR spectrum displayed a doublet at a triplet at δ 2.34 ppm (4H, 2CH_2_-C=O) corresponding to the succinimide side chain, exchangeable signals appeared at δ 6.61 (1H, NH), and 7.22 (2H, NH_2_). The ^13^C-NMR spectrum confirmed these findings, showing resonances at δ 35.7–35.8 (2CH_2_–C=O), respectively. Carbonyl signals were found at δ 179.0 and 179.9 (2C=O amide).

The synthetic pathway illustrated in Scheme 4 involves the reaction of compound **6** with diethyl malonate and carbon disulfide in the presence of a basic medium, affording two distinct products, compounds **9** and **10**. The formation of compound **9** can be attributed to the intramolecular cyclization of the hydrazide moiety (–NHNH_2_), leading to the construction of a 1,2-pyrazolidine-3,5-dione ring system. In contrast, compound **10** resulted from a different cyclization route, in which condensation with carbon disulfide promoted the generation of a 1*H*-1,2,4-triazole-3-thiol nucleus. Subsequently, compound **9** underwent a nucleophilic substitution reaction with 1,2-dichloroethane, yielding compound **11** with the elimination of HCl. This transformation confirmed the successful alkylation of the pyrazolidine ring system, introducing an additional aliphatic linkage and expanding the heterocyclic framework.

The disappearance of the hydrazide group (–NHNH_2_) observed in the precursor **6** confirmed the occurrence of intramolecular cyclization of **9**. In the IR spectrum, the absence of NHNH_2_ bands was accompanied by the appearance of a new absorption at 3335 cm^−1^ corresponding to the NH group and two strong carbonyl stretching bands around 1700 cm^−1^, characteristic of the newly formed imide (2 C=O) functionality within the 1,2-pyrazolidine-3,5-dione ring system. The ^1^H NMR spectrum further supported this transformation, where the signal corresponding to NHNH_2_ protons vanished, while singlet signal at δ 2.00 ppm due to CH_2_ pyrazolidine formed and a new downfield singlet appeared at δ 11.26 ppm, assigned to the NH proton of the pyrazolidine-3,5-dione moiety. Similarly, the ^13^C NMR spectrum confirmed ring closure by the appearance of two new deshielded carbonyl carbons at δ 170.0 and 172.7 ppm, typical of imide carbonyls.

The spectroscopic data clearly confirm the successful cyclization of the hydrazide precursor **6** into a triazole-thiol system **10**. The disappearance of the hydrazide group (-NHNH_2_) indicates completion of the ring-closure reaction. In the IR spectrum, new absorption bands appeared at 3402 cm^−1^ corresponding to the NH stretching vibration and at 2558 cm^−1^ due to the SH stretching of the newly formed thiol group, while new signals at 1664 and 1612 cm^−1^ are assigned to C=N stretching vibrations of the triazole ring, confirming the successful formation of the 1*H*-1,2,4-triazole-3-thiol moiety. In the ^1^H NMR spectrum, the disappearance of the hydrazide protons was accompanied by the appearance of two new downfield singlets at δ 12.12 ppm (-SH) and δ 12.7 ppm (–NH), characteristic of thiol and triazole NH protons, respectively, verifying the incorporation of both functionalities in the new ring system. The ^13^C NMR spectrum supported these findings through the appearance of two new signals at δ 145.0 ppm and 163.1 ppm, assigned to C=N (triazole–SH) and C=N triazole–NH carbons, respectively, which were absent in the precursor spectrum. Mass spectrometric analysis revealed a molecular ion peak at *m*/*z* = 579, consistent with the proposed molecular formula, and a base peak at *m*/*z* = 166.

The spectroscopic data of compound **11** provide clear evidence for the successful formation of the new heterocyclic system through alkylation of compound **9** with 1,2-dichloroethane, accompanied by the elimination of HCl. The IR spectrum shows the disappearance of the characteristic band of the imine group (C=NH), confirming its participation in the reaction. In contrast, the appearance of new absorption bands at 1668 and 1655 cm^−1^ corresponds to C=N stretching vibrations, indicating the generation of a new imine linkage within the extended heterocyclic system. The ^1^H NMR spectrum revealed the emergence of two new triplet signals at δ 1.75 and δ 3.05 ppm, attributed to the two methylene groups (–CH_2_-N and –CH_2_-Cl) introduced via 1,2-dichloroethane, confirming the successful alkylation step. Meanwhile, the previous C=NH-related proton signal observed in the precursor spectrum disappeared, verifying that the imine hydrogen was consumed during ring expansion and substitution. The ^13^C NMR spectrum further supports this structural transformation through the appearance of two new carbon signals at δ 40.05 and 40.34 ppm corresponding to the two methylene carbons (–CH_2_-N and –CH_2_-Cl). Moreover, the absence of the C=NH carbon signal and the presence of a new C=N resonance at δ 156.5 ppm confirm the formation of a substituted imine-type environment. Collectively, these spectral features confirm the conversion of compound **9** into compound **11** via nucleophilic substitution with 1,2-dichloroethane, leading to the formation of new methylene linkages and the loss of the imine functionality. Mass spectrometric analysis revealed a molecular ion peak at *m*/*z* = 667, consistent with the proposed molecular formula, and a base peak at *m*/*z* = 302.

Scheme 5 demonstrates the versatile reactivity of compound **10** through functional transformation of its thiol group (–SH) under different conditions. Treatment with hydrazine hydrate converted the thiol into a hydrazino group (–NHNH_2_), yielding compound **12** via nucleophilic substitution. Reaction with glacial acetic acid led to selective S-acylation of the thiol group, affording the corresponding S-acetylated derivative (compound **13**). Similarly, treatment with 2-chloroacetyl chloride resulted in S-alkylation, yielding the chloroacetyl thioether derivative (compound **14**). The absence of cyclization in both cases can be attributed to the limited nucleophilicity of adjacent nitrogen centers relative to the highly reactive thiol group, in addition to possible steric constraints imposed by the bulky polyheteroaromatic framework, which disfavors effective intramolecular attack. Furthermore, the reaction conditions preferentially promote intermolecular S-functionalization over intramolecular ring closure. These findings highlight the dominant role of the thiol moiety as a soft nucleophile that selectively undergoes S-functionalization under electrophilic conditions.

The IR spectrum of compound **12** showed the disappearance of the thiol group (–SH) present in compound **10** and the appearance of new absorption bands at 3412–3196 cm^−1^, corresponding to the stretching vibrations of amino and secondary amine groups (–NH_2_, –NH). This confirms the successful conversion of the thiol moiety into a hydrazino function (–NHNH_2_). In the ^1^H NMR spectrum, the absence of the SH proton signal (previously at δ 12.12 ppm) and the appearance of new exchangeable signals at δ 4.50 ppm (–NH_2_) and δ 9.22 ppm (–NH) confirm the introduction of the hydrazino functionality. The ^13^C NMR spectrum further supports this transformation, showing characteristic signals at δ 146.3 and 163.3 ppm, corresponding to C=N–triazole–NH carbons. Mass spectrometric analysis revealed a molecular ion peak at *m*/*z* = 577, consistent with the proposed molecular formula, and a base peak at *m*/*z* = 299.

The IR spectrum of compound **13** exhibited a disappearance of SH stretching band indicating the replacement of hydrogen by acetyl group. The ^1^H NMR spectrum revealed characteristic signals at δ 2.20 ppm for methyl group and broad singlet at δ 12.25 ppm (NH-pyrazole, D_2_O exchangeable) confirm amide NH functionality. Importantly, the NH proton signal shifted slightly from δ 12.72 ppm in the precursor to δ 12.25 ppm. The ^13^C NMR spectrum displayed resonances for 2CH_3_ (δ 24.3, 25.3 ppm), CH_2_-S (δ 28.3 ppm), CH_2_-diazole (δ 32.5 ppm), and C=O (δ 151.6, 170.0 ppm). These results collectively confirm the successful formation of compound **13**, where the thiol (–SH) group was converted into a SCH_3_. The downshift of both IR (3402 → 3169 cm^−1^) and NH proton (12.72 → 12.25 ppm) signals strongly supports the cyclization and formation of the triazole-fused heterocyclic system.

The IR spectrum of compound **14** revealed the disappearance of the thiol (–SH) absorption band observed in the precursor compound **10** and the emergence of new absorption bands at 1679 cm^−1^, attributed to new acetyl group. The ^1^H NMR spectrum exhibited characteristic aliphatic signal at δ 4.53 ppm for CH_2_-Cl. The ^13^C NMR spectrum displayed signal for CH_2_-Cl δ 48.0 ppm. These spectral changes collectively confirm that compound **14** was formed via conversion of the thiol (–SH) group into a SCOCH_2_Cl.

In conclusion, a novel series of fourteen heterocyclic derivatives was efficiently synthesized and structurally confirmed through detailed spectroscopic analyses. The designed compounds incorporate diverse heterocyclic frameworks such as thiadiazol, tetrazole, triazole, pyrazolidine-3,5-dione, and triazolo–triazine rings, expected to enhance their biological performance. All synthesized derivatives (**1**–**14**) will be further evaluated for their anticancer and antimicrobial activities to explore their potential as promising multifunctional therapeutic agents. The presence of multiple heteroatoms (N, O, S), conjugated π-systems, and diverse pharmacophoric motifs within these frameworks suggests that the newly synthesized compounds may exhibit significant biological potential. Consequently, all target molecules will be subjected to in vitro anticancer and in vivo antimicrobial screening to assess their efficacy and to establish structure activity relationships (SAR) that may guide further molecular optimization toward potent dual-action therapeutic candidates.

### 2.2. Biological Evaluation

#### 2.2.1. Antiproliferative In Vitro Potency

The cytotoxic potential of the synthesized compounds **1**–**14** was evaluated against three human cancer cell lines, namely HeLa (cervical carcinoma), HepG-2 (hepatocellular carcinoma), and MCF-7 (breast adenocarcinoma), in addition to the non-cancerous WI-38 human lung fibroblast cell line to assess safety. Cytotoxicity was determined using the MTT assay [70,71,72,73,74,75], which revealed variable micromolar IC_50_ values for the tested compounds, with several derivatives exhibiting activities comparable to the reference drug doxorubicin (Table 1 and Table S1).

Compound **13** exhibited potent cytotoxic activity across all tested cancer cell lines, particularly against MCF-7 (IC_50_ = 3.87 ± 0.2 µM), 5.06 ± 0.3 µM against HepG-2, and 7.81 ± 0.6 µM against HeLa. Notably, its potency against MCF-7 cells surpassed that of doxorubicin (IC_50_ = 4.17 ± 0.2 µM), indicating a pronounced antiproliferative effect against breast cancer cells. Compound **4** also displayed excellent inhibitory activity, especially toward MCF-7 (IC_50_ = 6.15 ± 0.4 µM) and HepG-2 (12.71 ± 1.0 µM), confirming a strong cytotoxic response within the low micromolar range. In addition, compound **7** demonstrated significant inhibition against HeLa (22.35 ± 1.6 µM) and MCF-7 (15.56 ± 1.3 µM), while compound **12** showed moderate cytotoxicity toward HepG-2 (21.46 ± 1.6 µM) and MCF-7 (23.12 ± 1.7 µM). Furthermore, compounds **1**, **9**, and **14** exhibited activities spanning the low to moderate micromolar range, with IC_50_ values between 19.50 and 44.30 µM across the tested cell lines. Interestingly, compound **1** showed effective inhibition of HeLa (IC_50_ = 9.74 ± 0.8 µM) and HepG-2 (8.61 ± 0.6 µM) cells, while displaying comparatively weaker activity against MCF-7 cells (11.62 ± 0.9 µM). On the other hand, compounds **3**, **5**, **8**, and **10** were weakly cytotoxic or inactive, exhibiting IC_50_ values exceeding 60 µM against all evaluated cancer cell lines.

All synthesized derivatives were further evaluated against normal WI-38 lung fibroblast cells to assess selectivity and safety. The most promising compounds, namely **13**, **4**, and **7**, demonstrated acceptable IC_50_ values of 41.42 ± 2.6, 55.13 ± 3.2, and 58.60 ± 3.5 µM, respectively, indicating relatively low toxicity toward normal cells when compared with doxorubicin (IC_50_ = 6.72 ± 0.5 µM). The Selectivity Index (SI) was calculated as the ratio between IC_50_ values obtained for WI-38 normal cells and those for the corresponding cancer cell lines. Compound **13** exhibited the highest selectivity toward tumor cells, with SI values of 10.7 for MCF-7, 8.2 for HepG-2, and 5.3 for HeLa. Similarly, Compound **4** demonstrated strong selectivity, showing SI values of 8.9 for MCF-7, 4.3 for HepG-2, and 3.3 for HeLa. These findings clearly identify Compounds **13** and **4** as the most promising anticancer leads, combining potent cytotoxic activity with favorable selectivity toward malignant cells.

##### Structure Activity Relationship (SAR) Study

Considering the cytotoxicity data summarized in Table 1, the chalcone, 4,5-dihydro-1*H*-pyrazoline-1-carbothiohydrazide, and 2-1*H*-pyrazoline-thiadiazole-based derivatives **1**–**14** exhibited a clear structure-activity relationship governed by electronic conjugation, molecular planarity, and hydrophobic balance.

The parent chalcone derivative, *N*-acetyl-3-(4-chlorophenyl)-*N*-(4-hydroxyphenyl)acrylamide (**1**), displayed moderate to strong cytotoxic activity (IC_50_ = 8–12 µM), which can be attributed to its planar acrylamide linker and balanced hydrophilic lipophilic profile, favoring π–π stacking and hydrogen-bonding interactions. In contrast, structural conversion into the hydrazinecarbonothioyl–pyrazoline analogue **2** and the thiadiazole derivative **3** markedly reduced potency (IC_50_ = 30–80 µM), likely due to increased polarity, steric congestion, and compromised membrane permeability. Restoration of lipophilicity through cyano–alkylthio substitution in compound **4** significantly enhanced cytotoxic potency, affording low micromolar IC_50_ values (6–16 µM) across all tested cell lines, particularly MCF-7 cells. This improvement highlights the beneficial role of electron-withdrawing substituents in reinforcing conjugation and non-covalent interactions. Conversely, further polarity-enhancing modifications, such as tetrazole **5** or acetamido-hydrazide **6** formation, resulted in diminished activity, whereas moderate hydrophobic acylation of the tetrazole scaffold in compound **7** partially restored potency (IC_50_ = 15–22 µM). Excessive polar extension, as observed in compound **8**, again led to reduced cytotoxicity, consistent with limited cellular uptake.

**Figure 4:**
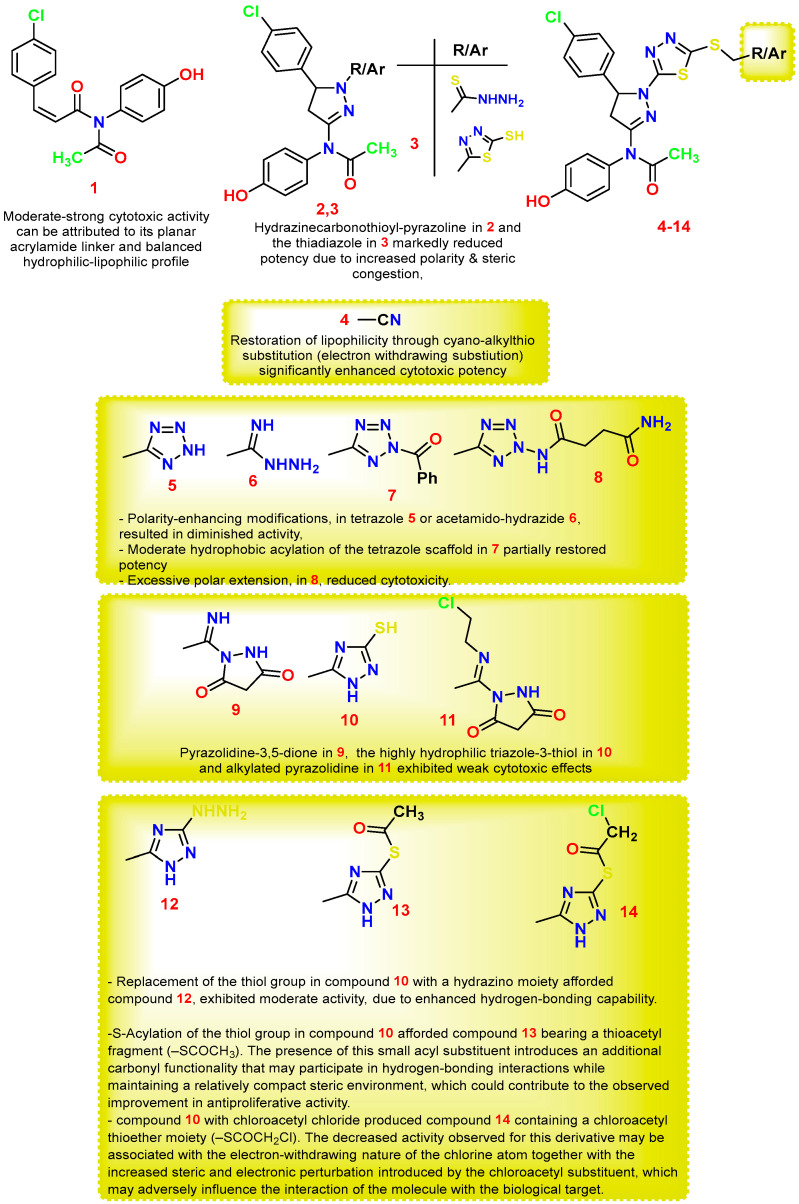
Structure-activity relationship (SAR) overview of the synthesized chalcone, pyrazoline, and pyrazoline-thiadiazole derivatives **1**–**14**, highlighting the impact of structural modifications, electronic conjugation, molecular planarity, and hydrophobic balance on cytotoxic activity against HeLa, HepG-2, and MCF-7 cancer cell lines.

Cyclization of hydrazide intermediates yielded heterocyclic derivatives with variable activity. The pyrazolidine-3,5-dione analogue **9** retained moderate potency, whereas the highly hydrophilic triazole-3-thiol derivative **10** showed weak activity. Alkylation of the pyrazolidine core in compound **11** improved lipophilicity and partially restored cytotoxic effects. Replacement of the thiol group in compound **10** with a hydrazino moiety afforded compound **12**, which exhibited moderate activity, potentially due to enhanced hydrogen-bonding capability. Notably, Compound **13**, obtained via S-acylation of the thiol group with acetic acid to introduce a thioacetyl fragment (–SCOCH_3_), emerged as the most potent derivative, displaying strong antiproliferative activity (IC_50_ = 3.8–7.8 µM) and excellent selectivity (SI = 8–11), surpassing the reference drug sorafenib. The presence of the small thioacetyl substituent introduces an additional carbonyl functionality capable of participating in hydrogen-bonding interactions while maintaining a relatively compact steric environment, which may favor productive interactions with the biological target. In contrast, compound **14**, bearing a chloroacetyl thioether moiety (–SCOCH_2_Cl) introduced via reaction with chloroacetyl chloride, exhibited reduced activity (IC_50_ = 19–32 µM). This decrease may be attributed to the electron-withdrawing nature of the chlorine atom together with the increased steric and electronic perturbation introduced by the chloroacetyl substituent, which could adversely affect the interaction of the molecule with the biological target (Figure 4).

Generally, derivatives maintaining extended aromatic conjugation, moderate lipophilicity, and controlled hydrogen bonding capacity, particularly compounds **13** and **4,** exhibited the most favorable cytotoxic profiles, whereas excessive polarity consistently resulted in loss of activity. These findings underscore the importance of optimizing hydrophilic lipophilic balance and electronic distribution for enhanced antiproliferative performance.

#### 2.2.2. Effect of the 2-1*H*-Pyrazoline-thiadiazole-Based Derivative **13** on ERK2 and RIPK3 Activation and Necroptotic Signaling

Based on the in vitro cytotoxicity screening of the synthesized compounds, the 2-1*H*-pyrazoline-thiadiazole-based derivative **13** emerged as the most potent analogue across all tested cell lines, exhibiting its highest antiproliferative activity against MCF-7 breast cancer cells. Accordingly, 2-1*H*-pyrazoline-thiadiazole-based derivative **13** was selected for further mechanistic evaluation in MCF-7 cells using enzyme-based assays targeting ERK2 and RIPK3.

Treatment of MCF-7 cells with 2-1*H*-pyrazoline-thiadiazole-based derivative **13** resulted in a marked elevation in total ERK2 levels, which increased from 0.738 ± 0.03 pg/mL in untreated control cells to 2.295 ± 0.09 pg/mL, corresponding to an approximately 3.17-fold increase (Tables S2 and S3).

This activation of ERK2 reflects an early cellular stress response, consistent with reports describing transient ERK signaling induction following exposure to cytotoxic agents, often preceding cell cycle arrest and apoptotic signaling.

In parallel, the involvement of necroptotic pathways was assessed through quantification of receptor-interacting protein kinase 3 (RIPK3), a central regulator of programmed necrotic cell death. 2-1*H*-pyrazoline-thiadiazole-based derivative **13** induced a pronounced up-regulation of RIPK3 expression, with levels rising from 29.07 ± 1.41 pg/mL in control cells to 348.14 ± 12.53 pg/mL in treated cells, representing an approximately 11.97-fold increase (Tables S4 and S5).

**Figure 5:**
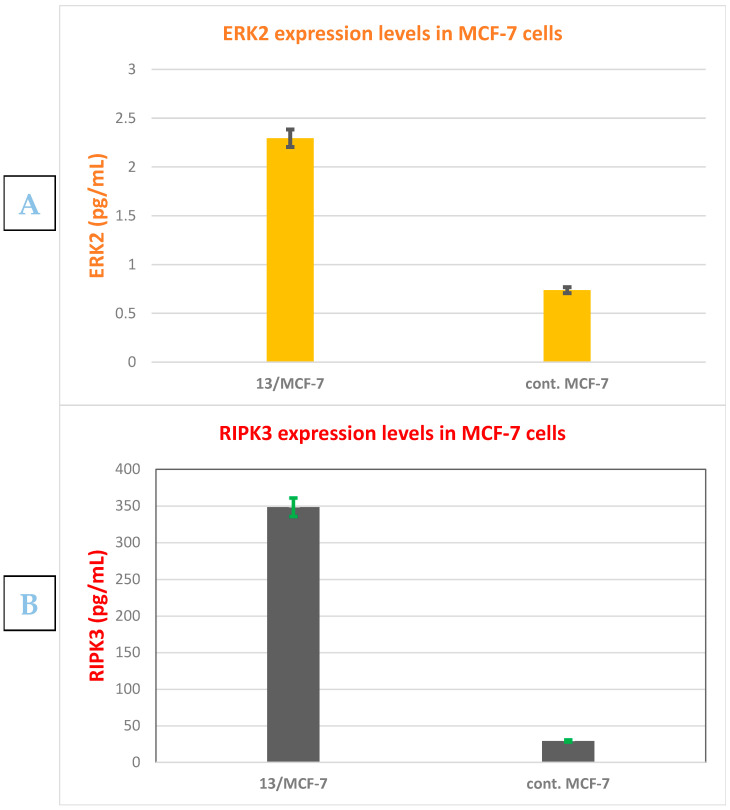
Effect of 2-1*H*-pyrazoline-thiadiazole-based analogue **13** on ERK2 and RIPK3 expression levels in MCF-7 cells. (**A**) ERK2 expression levels. (**B**) RIPK3 expression levels. Data are presented as the mean ± SD.

Therefore, the concurrent activation of ERK2 signaling and the substantial elevation of RIPK3 expression, as summarized in the combined Excel-based Figure 5A,B, indicate that 2-1*H*-pyrazoline-thiadiazole-based derivative **13** triggers an early stress-responsive signaling cascade and suggests the involvement of necroptotic signaling pathways alongside apoptotic cell-death mechanisms. These combined effects likely contribute to the pronounced cytotoxic activity of 2-1*H*-pyrazoline-thiadiazole-based derivative **13** against MCF-7 breast cancer cells.

#### 2.2.3. Cell Cycle Perturbation and G2/M Phase Arrest of the 2-1*H*-Pyrazoline-thiadiazole-Based Derivative **13**

**Figure 6:**
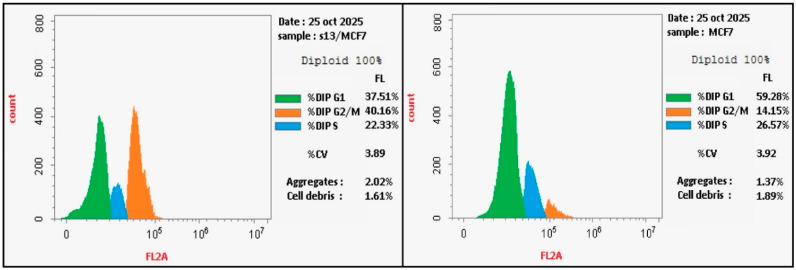
Representative flow-cytometric DNA content histograms showing the effect of the 2-1*H*-pyrazoline-thiadiazole-based analogue **13** on cell-cycle distribution in MCF-7 cells.

**Figure 7:**
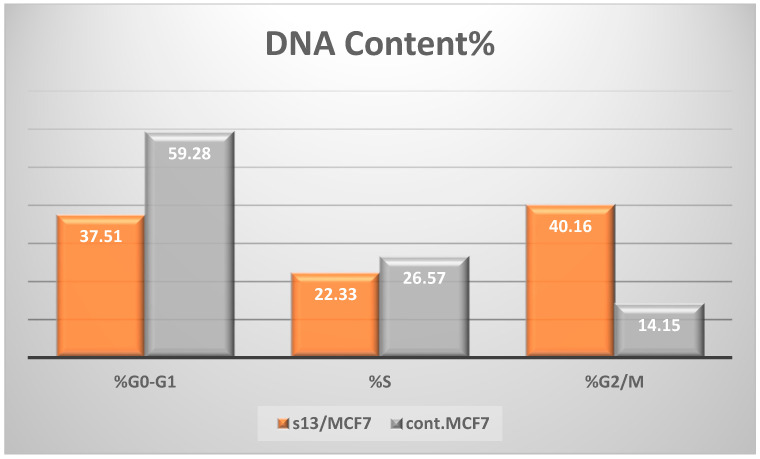
Comparative bar chart showing the percentage distribution of MCF-7 cells across different cell cycle phases (G0/G1, S, and G2/M) after treatment with 2-1*H*-pyrazoline-thiadiazole-based analogue **13** versus control.

Flow-cytometric analysis of cellular DNA content demonstrated that treatment of MCF-7 cells with the 2-1*H*-pyrazoline-thiadiazole-based analogue **13** induced a pronounced cell-cycle arrest at the G2/M phase. The proportion of cells accumulated in the G2/M phase markedly increased from 14.15% in untreated control cells to 40.16% following treatment. Conversely, the percentages of cells in the G0/G1 and S phases decreased substantially, from 59.28% to 37.51% and from 26.57% to 22.33%, respectively (Figure 6 and Figure 7, and Table S6).

This redistribution of cell-cycle phases indicates that 2-1*H*-pyrazoline-thiadiazole-based analogue **13** interferes with cell cycle progression before mitotic entry, consistent with activation of G2/M checkpoint control mechanisms. Such G2/M arrest represents a key antiproliferative effect, which may contribute to growth inhibition and facilitate downstream cell death responses.

#### 2.2.4. Induction of Apoptosis by the 2-1*H*-Pyrazoline-thiadiazole-Based Derivative **13**

**Figure 8:**
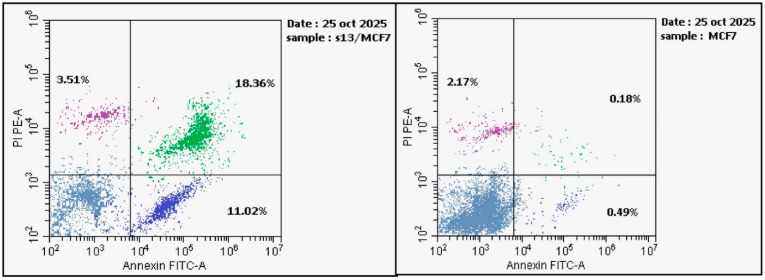
Representative flow cytometry plots illustrating apoptosis induction in MCF-7 cells treated with 2-1*H*-pyrazoline-thiadiazole-based derivative **13**.

**Figure 9:**
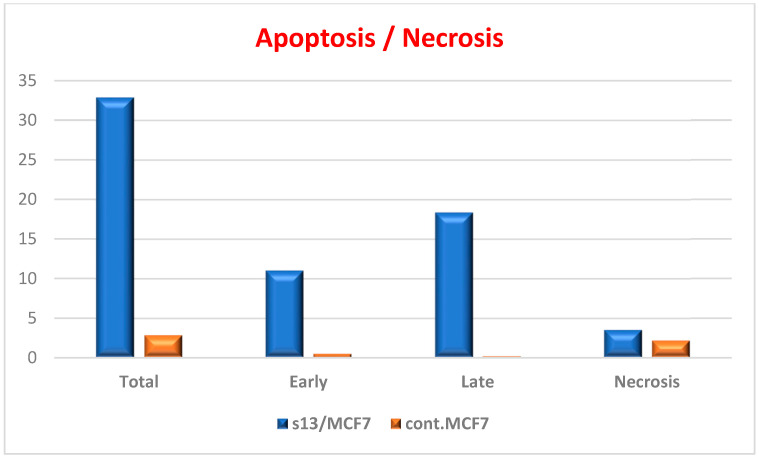
Bar chart showing the percentage of apoptotic MCF-7 cells following treatment with 2-1*H*-pyrazoline-thiadiazole-based derivative **13**.

Annexin V/PI dual-staining analysis demonstrated that treatment of MCF-7 cells with 2-1*H*-pyrazoline-thiadiazole-based analogue **13** markedly induced apoptotic cell death. The total apoptotic population increased from 2.84% in untreated control cells to 32.89% following treatment, corresponding to an approximately 11.6-fold elevation. Both early apoptotic (11.02%) and late apoptotic (18.36%) cell populations were substantially increased. In contrast, the proportion of necrotic cells remained relatively low and showed only a minor change (3.51% in treated cells versus 2.17% in control cells) (Figure 8 and Figure 9, and Table S7). These results indicate that the cytotoxic effect of 2-1*H*-pyrazoline-thiadiazole-based derivative **13** is primarily mediated through the induction of programmed apoptosis rather than non-specific necrosis.

#### 2.2.5. Induction of p53 Tumor Suppressor Protein of the 2-1*H*-Pyrazoline-thiadiazole-Based Derivative **13**

**Figure 10:**
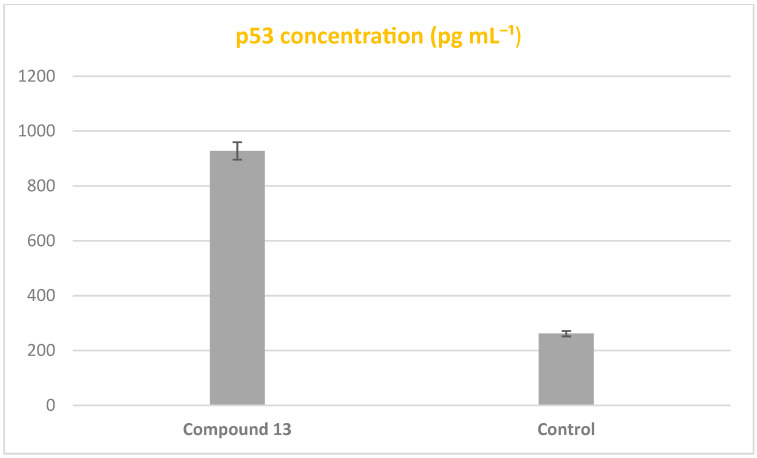
Effect of the 2-1*H*-pyrazoline-thiadiazole-based analogue **13** on p53 protein expression in MCF-7 cells.

Treatment of MCF-7 cells with the 2-1*H*-pyrazoline-thiadiazole-based analogue **13** resulted in a pronounced elevation in the level of the tumor suppressor protein p53, reaching 927.66 ± 32.06 pg/mL compared with 261.54 ± 9.87 pg/mL in untreated control cells. This corresponds to an approximately 3.54-fold increase in p53 expression (Tables S8 and S9, and Figure 10). Such marked up-regulation of p53 suggests activation of p53-associated stress response signaling, which plays a central role in the regulation of cell cycle checkpoints and apoptosis. The observed increase in p53 levels likely reflects a cellular protective response to compound-induced stress, promoting growth arrest and programmed cell death to limit malignant proliferation.

#### 2.2.6. Modulation of Bcl-2 and BAX Expression by the 2-1*H*-Pyrazoline-thiadiazole-Based Derivative **13**

**Figure 11:**
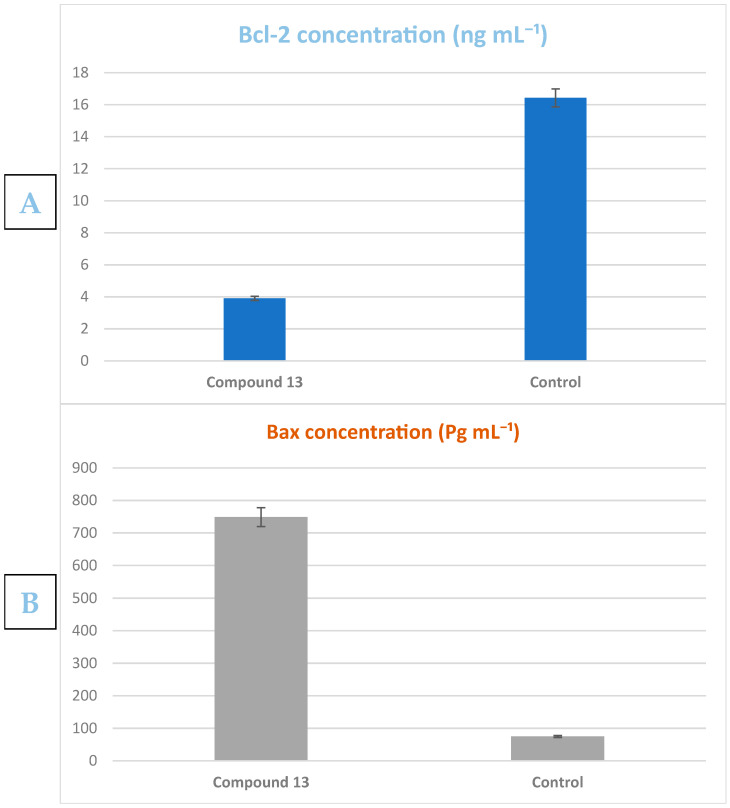
Effect of the 2-1*H*-pyrazoline-thiadiazole-based analogue **13** on Bcl-2 and BAX expression in MCF-7 cells. (**A**) Bcl-2 expression. (**B**) BAX expression.

Treatment of MCF-7 cells with the 2-1*H*-pyrazoline-thiadiazole-based analogue **13** resulted in a pronounced alteration in the expression balance between the anti-apoptotic protein Bcl-2 and the pro-apoptotic protein BAX. The level of Bcl-2 markedly decreased from 16.43 ± 0.56 ng mL^−1^ in untreated control cells to 3.91 ± 0.13 ng mL^−1^ following treatment, corresponding to an approximately 76% reduction (0.24-fold) (Tables S10 and S11, and Figure 11A).

In contrast, BAX expression was strongly up-regulated upon treatment with compound **13**. The concentration of BAX increased from 74.94 ± 2.91 pg mL^−1^ in control cells to 748.88 ± 29.10 pg mL^−1^ in treated cells, representing an approximately 9.99-fold elevation (Tables S12 and S13, and Figure 11B).

This reciprocal modulation of Bcl-2 and BAX indicates that compound **13** substantially shifts the intracellular balance toward a pro-apoptotic state. Consistently, the calculated BAX/Bcl-2 ratio increased by nearly 42-fold relative to control cells, supporting the involvement of mitochondrial-mediated apoptotic signaling. This expression pattern is in agreement with the observed p53 up-regulation, which is known to transcriptionally repress Bcl-2 and induce BAX expression, thereby promoting mitochondrial outer membrane permeabilization and downstream activation of programmed cell-death pathways.

#### 2.2.7. Antimicrobial Efficiency

##### In Vitro Antibacterial Activity

The newly synthesized 2-1*H*-pyrazoline-thiadiazole-based derivatives **1**–**14** were evaluated in vitro for their antibacterial activity against methicillin-resistant *Staphylococcus aureus* (MRSA, USA300) as a representative Gram-positive strain and *Acinetobacter baumannii* AB5057 as a representative Gram-negative strain.

The results demonstrated that all tested derivatives exhibited no detectable antibacterial activity against either bacterial strain, with the exception of compounds **7** and **13**.

Both compounds showed comparable inhibitory effects against MRSA USA300 and *A. baumannii* AB5057, with minimum inhibitory concentration (MIC) values of 10 mg/mL, corresponding to 15.325 μM for compound **7** and 16.6254 μM for compound **13**. Notably, the minimum bactericidal concentration (MBC) values were identical to the corresponding MIC values for both compounds, indicating bactericidal activity at the tested concentrations (Table 2).

##### Structure-Activity Relationship (SAR)

The selective antibacterial activity observed for 2-1*H*-pyrazoline-thiadiazole-based analogues **7** and **13**, in contrast to the remaining synthesized derivatives, can be rationalized by the presence of specific functional groups that collectively optimize the balance between polarity, hydrogen-bonding capacity, and molecular flexibility. While all compounds share the same 2-1*H*-pyrazoline-thiadiazole core, the distinguishing factor lies in the nature of the terminal heterocyclic and aromatic substituents.

In 2-1*H*-pyrazoline-thiadiazole-based analogue **7**, the incorporation of a tetrazole ring tethered to a carbonyl-linked phenyl group (C=O–Ph) plays a pivotal role in conferring antibacterial activity.

The tetrazole moiety, rich in nitrogen atoms, provides multiple hydrogen bond acceptor sites and can act as a bio isosteric replacement for acidic functionalities, thereby enhancing polar interactions with bacterial components. Simultaneously, the adjacent phenyl carbonyl fragment introduces aromaticity and conformational rigidity, which may facilitate transient interactions with bacterial membranes or intracellular targets.

As a result, 2-1*H*-pyrazoline-thiadiazole-based analogue **7** exhibited detectable antibacterial activity against both MRSA USA300 and *Acinetobacter baumannii* AB5057 with an MIC value of 10 mg/mL, corresponding to 15.325 μM, indicating that the coexistence of these polar and aromatic features is sufficient to reach the minimum threshold required for bacterial growth inhibition a property absent in less functionally diversified analogues. Conversely, 2-1*H*-pyrazoline-thiadiazole-based analogue **13** derives its activity from a distinct yet functionally complementary structural motif, triazole rings being acetyl-substituted (SCOCH_3_).

This fused heterocyclic framework increases heteroatom density and electronic delocalization, promoting π–electron interactions and coordination potential. The methyl substituent subtly modulates lipophilicity and steric distribution, which may enhance membrane permeation without excessively compromising aqueous solubility. Consequently, 2-1*H*-pyrazoline-thiadiazole-based analogue **13** displayed antibacterial activity comparable to that of 2-1*H*-pyrazoline-thiadiazole-based analogue **7**, with an identical MIC value of 10 mg/mL against both tested strains, corresponding to 16.6254 μM, suggesting that differences in molar activity primarily reflect molecular weight rather than intrinsic potency.

Overall, this SAR analysis indicates that antibacterial activity in this series is not governed by the core scaffold alone but rather by the synergistic contribution of terminal functional groups, which collectively enable weak yet detectable antibacterial effects at higher concentrations, as reflected by the observed MIC values.

##### Effect of 2-1*H*-Pyrazoline-thiadiazole-Based Analogues **7** and **13** on *MRSA* USA300 and *Acinetobacter baumannii* AB5057 Biofilm Activity

**Figure 12:**
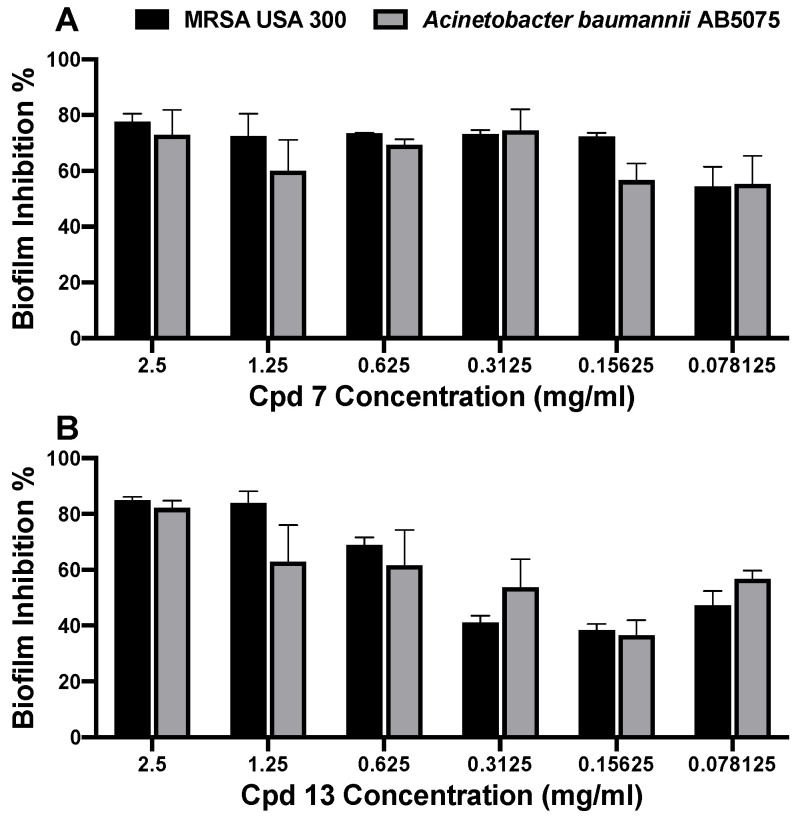
Effect of sub-MBC (2.5–0.078125 mg/mL) of 2-1*H*-pyrazoline-thiadiazole-based analogues **7** (**A**) and **13** (**B**) on biofilm formation of MRSA USA300 and *Acinetobacter baumannii* AB5057. Data are expressed as the mean biofilm inhibition (%) ± SEM.

**Figure 13:**
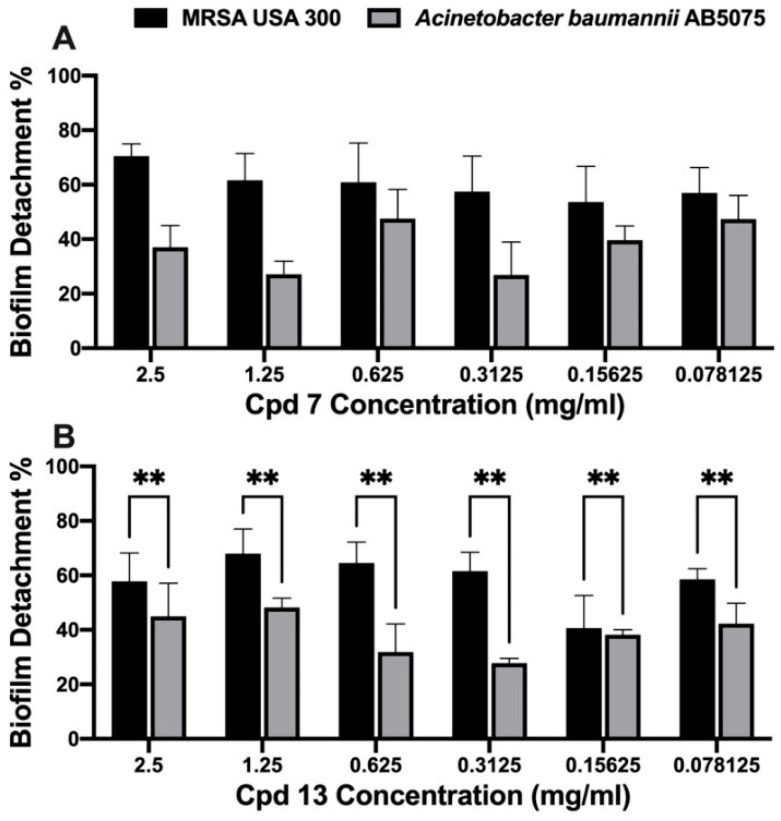
Effect of sub-MBC (2.5–0.078125 mg/mL) of 2-1*H*-pyrazoline-thiadiazole-based analogues **7** (**A**) and **13** (**B**) on pre-formed biofilms of MRSA USA300 and *Acinetobacter baumannii* AB5057. Data are expressed as the mean biofilm detachment (%) ± SEM. ** indicate that the difference is significant at *p* < 0.01, respectively, (Two-way ANOVA, Sidak’s post hoc test).

The antibiofilm activity of 2-1*H*-pyrazoline-thiadiazole-based analogues **7** and **13** was assessed against both biofilm formation and pre-established biofilms of MRSA USA300 and *Acinetobacter baumannii* AB5057. Both compounds significantly inhibited biofilm formation of the two tested strains across all examined concentrations (two-way ANOVA with Sidak’s post hoc test, *p* < 0.05) (Figure 12). In addition, both compounds significantly disrupted pre-formed biofilms of MRSA USA300 and *A. baumannii* AB5057 throughout the tested concentration range (*p* < 0.05) (Figure 13).

As gained, both derivatives demonstrated greater efficacy against MRSA biofilms than against *A. baumannii* AB5057 biofilms. In the biofilm inhibition assay, 2-1*H*-pyrazoline-thiadiazole-based analogue **7** showed pronounced activity, with mean IC_50_ values of 0.0001995 mg/mL (0.000306 μM) against MRSA USA300 and 0.003382 mg/mL (0.0052 μM) against *A. baumannii* AB5057. In contrast, 2-1*H*-pyrazoline-thiadiazole-based analogue **13** exhibited markedly higher IC_50_ values of 0.9680 mg/mL (1.6093 μM) and 19.14 mg/mL (31.821 μM) against the respective strains, indicating reduced inhibitory potency.

A comparable pattern was observed in the biofilm detachment assay. 2-1*H*-pyrazoline-thiadiazole-based analogue **7** displayed DC_50_ values of 0.1166 mg/mL (0.1787 μM) for MRSA USA300 and 0.4684 mg/mL (0.7178 μM) for *A. baumannii* AB5057. Conversely, 2-1*H*-pyrazoline-thiadiazole-based analogue **13** showed weaker detachment activity, with DC_50_ values of 0.2502 mg/mL (~0.40 μM) and 57.25 mg/mL (~90.6 μM) against MRSA USA300 and *A. baumannii* AB5057, respectively.

##### In Vivo MRSA Skin Infection Model

**Figure 14:**
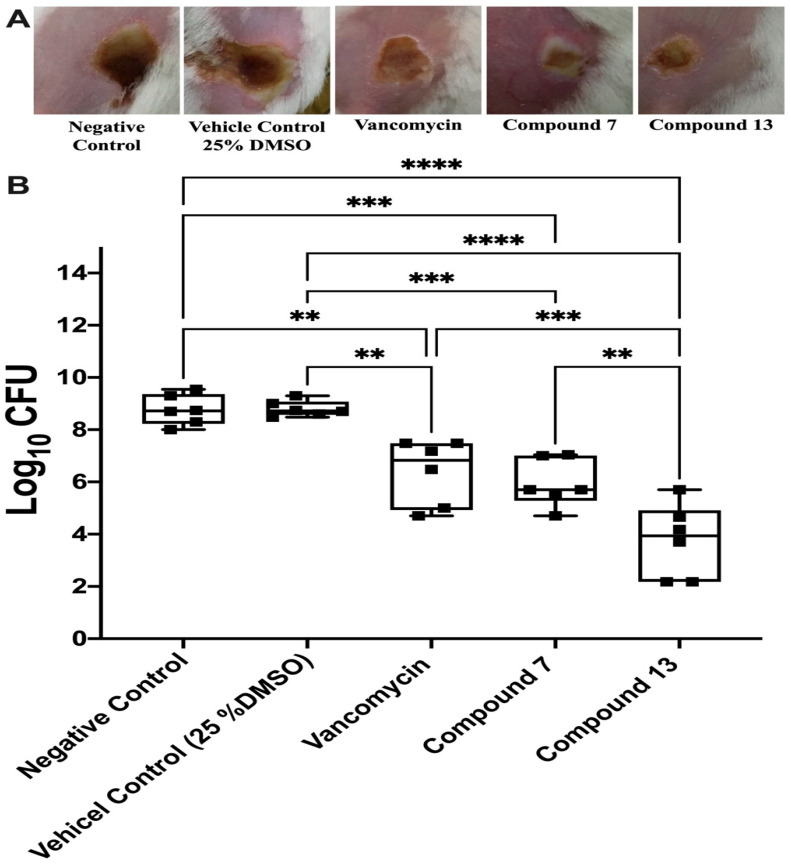
In vivo antibacterial efficacy of 2-1*H*-pyrazoline-thiadiazole-based analogues **7** and **13** in an MRSA skin infection model. (**A**) Representative images of MRSA-induced skin lesions following treatment. (**B**) Bacterial load recovered from infected skin tissues after treatment with the indicated compounds. Each data point in the figure represents a mouse. Results are expressed as the mean ± standard error. **, ***, **** indicate that the difference is significant at *p* < 0.01, <0.001, and <0.0001, respectively (One-way ANOVA, Tukey’s post hoc test).

The in vivo antibacterial efficacy of 2-1*H*-pyrazoline-thiadiazole-based analogues **7** and **13** was evaluated using an MRSA skin infection model in BALB/c mice (n = 6 per group). Both compounds produced a significant reduction in the bacterial load of MRSA USA300 compared with the negative control and vehicle control (DMSO) groups (one-way ANOVA followed by Tukey’s post hoc test, *p* < 0.001), as illustrated in Figure 14, where each data point represents an individual mouse and results are expressed as the mean ± standard error (SEM).

Notably, 2-1*H*-pyrazoline-thiadiazole-based analogue **13** exhibited significantly superior in vivo antibacterial activity compared to 2-1*H*-pyrazoline-thiadiazole-based analogue **7** as well as the reference drug vancomycin (*p* < 0.01). Treatment with 2-1*H*-pyrazoline-thiadiazole-based analogue **7** resulted in reductions of 2.828 and 2.867 log units in bacterial counts relative to the negative control and vehicle control groups, respectively. In contrast, 2-1*H*-pyrazoline-thiadiazole-based analogue **13** achieved a more pronounced decrease, with bacterial loads reduced by 5.001 and 5.04 log units compared to the negative control and DMSO-treated groups, respectively.

##### Assay Against *S. aureus* DNA Gyrase on 2-1*H*-Pyrazoline-thiadiazole-Based Analogues **13** and **7**

**Figure 15:**
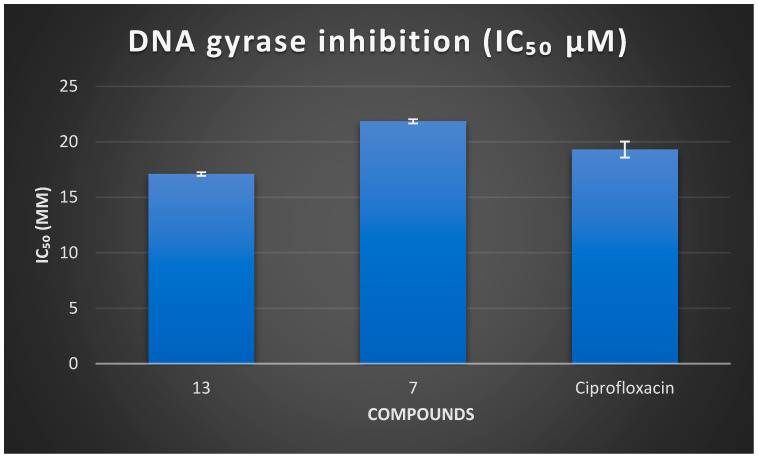
DNA gyrase inhibitory activity of the tested 2-1*H*-pyrazoline-thiadiazole-based analogs **13** and **7** against *S. aureus*. Data are expressed as the mean ± SD of three independent experiments.

2-1*H*-Pyrazoline-thiadiazole-based analogues **13** and **7** were investigated for their antibacterial activity against methicillin-resistant *Staphylococcus aureus* (MRSA), followed by evaluation of their inhibitory effects on *S. aureus* DNA gyrase to gain insight into a possible mechanism of action. DNA gyrase is a well-established antibacterial target involved in bacterial DNA replication. The quantitative DNA gyrase inhibition data, including IC_50_ values and percentage inhibition, are summarized in Table 3, while the graphical representation of the inhibition results is illustrated in Figure 15.

The DNA gyrase inhibition assay revealed that 2-1*H*-pyrazoline-thiadiazole-based analogue **13** exhibited potent inhibitory activity with an IC_50_ value of 17.10 ± 0.17 µM, which was comparable to the reference drug ciprofloxacin (IC_50_ = 19.30 ± 0.72 µM). 2-1*H*-Pyrazoline-thiadiazole-based analogue **7** also showed appreciable inhibitory activity, though to a lesser extent (IC_50_ = 21.85 ± 0.19 µM). The observed inhibition trend was consistent with the antibacterial activity recorded against MRSA.

The superior inhibitory performance of 2-1*H*-pyrazoline-thiadiazole-based analogue **13** relative to 2-1*H*-pyrazoline-thiadiazole-based analogue **7** may be attributed to structural features that enhance its interaction with the DNA gyrase active site, potentially leading to improved binding affinity. Although 2-1*H*-pyrazoline-thiadiazole-based analogue **13** demonstrated comparable enzyme inhibition to ciprofloxacin, differences in molecular scaffolds and physicochemical properties may influence cellular uptake and overall antibacterial activity. Representative gel images and the corresponding instrument-generated quantitative outputs supporting the DNA gyrase inhibition results are provided in Supplementary Tables S14 and S15.

### 2.3. Computational Studies

#### 2.3.1. Molecular Docking Simulation

Based on the potent in vitro inhibitory effects observed against ERK and RIPK3, molecular docking studies were carried out for 1,2,4-triazole-1,3,4-thiadiazole-1*H*-pyrazoline **13** using Molecular Operating Environment (MOE-Dock) software version 2024.0601 [76,77,78,79]. This study aimed to predict the binding affinities of compound **13** toward ERK1/2 and RIPK3 (PDB IDs: 4QP9 and 7XM3, respectively) [80,81]. The X-ray crystallographic structures of ERK2 and RIPK3, along with their co-crystallized ligands, 7-(1-propyl-1*H*-pyrazol-4-yl)-2-(pyridin-4-yl)-5*H*-pyrrolo[2,3-*b*]pyrazine (**35X**) and **GSK’843**, were retrieved from the Protein Data Bank. To validate the docking protocol, the native ligands **35X** and **GSK’843** were initially redocked into their corresponding binding sites, producing low RMSD values of 1.36 and 0.88 Å relative to the experimental poses, in addition to binding energy scores of −10.71 and −11.24 kcal/mol, respectively.

**Figure 16:**
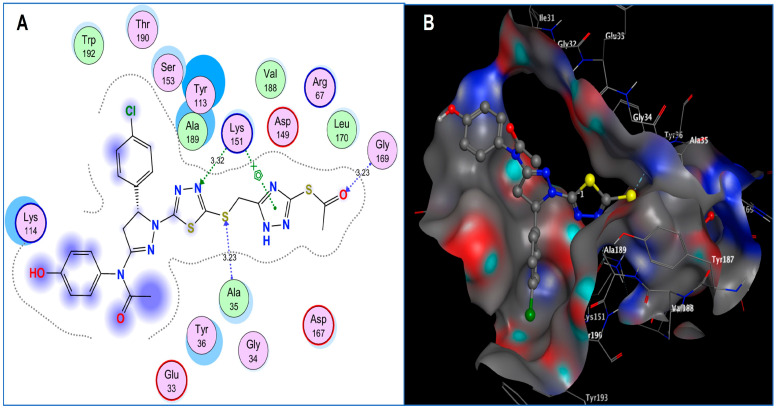
(**A**) and (**B**) diagrams demonstrate two and three-dimensional views of promising 1,2,4-triazole-1,3,4-thiadiazole-1*H*-pyrazoline **13** within the ERK2 active site (PDB code: 4QP9), respectively.

**Figure 17:**
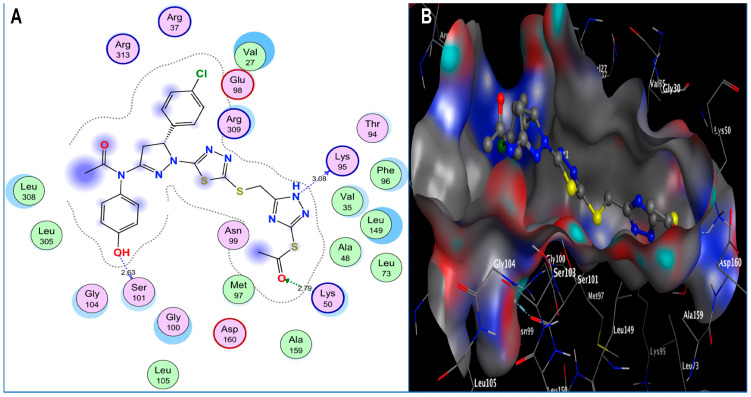
(**A**) and (**B**) diagrams demonstrate two and three-dimensional views of promising 1,2,4-triazole-1,3,4-thiadiazole-1*H*-pyrazoline **13** within the RIPK3 active site (PDB code: 7XM3), respectively.

With high energy scores of –9.89 and –10.75 kcal/mol, respectively, 1,2,4-triazole-1,3,4-thiadiazole-1*H*-pyrazoline **13** was well placed within ERK2 and RIPK3, as seen in Figure 16 and Figure 17, respectively. Regarding the ERK2 receptor, the amino acid **Lys151** afforded an H-bond with thiadiazole nitrogen (distance: 3.32 Å) and an arene-H interaction with the centroid of triazole. The sulfur atom of the thiomethyl chain applied another H-bonding with **Ala35** (distance: 3.23 Å). Additionally, the carbonyl oxygen of the ethanethioate moiety forms a hydrogen bond with **Gly169** (3.23 Å) (Figure 16A,B).

On the other hand, within the RIPK3 active site, the NH of triazole displayed a hydrogen bond donor with the **Lys95** backbone (distance: 3.08 Å). Furthermore, the oxygens of ethanethioate and hydroxyl groups displayed H-bonds with **Lys50** and **Ser101** (distances: 2.79 and 2.63 Å, respectively) (Figure 17A,B).

**Figure 18:**
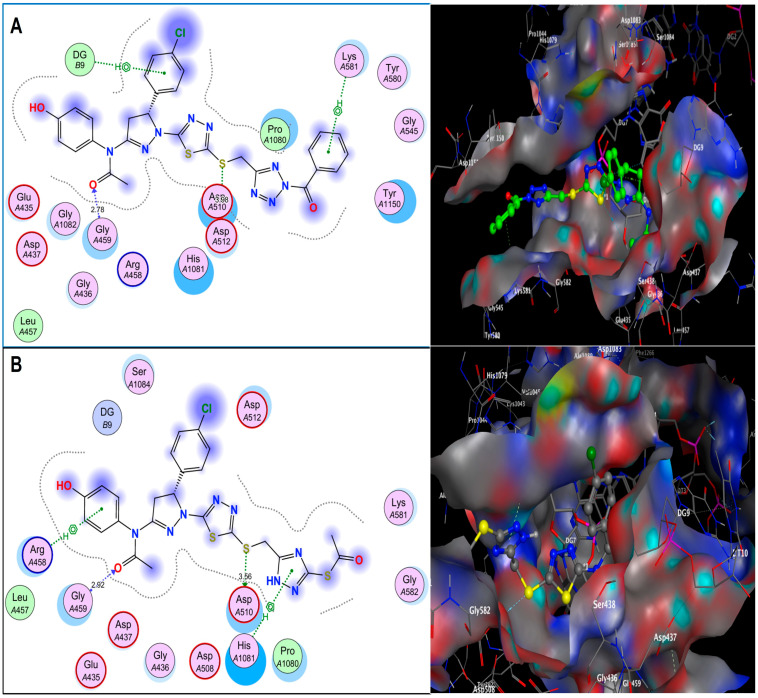
(**A**) and (**B**) diagrams demonstrate two and three-dimensional views of promising 1,3,4-thiadiazole-1*H*-pyrazolines **7** and **13** within the *Staphylococcus aureus* DNA gyrase active site (PDB ID: 2XCT), respectively.

Considering the remarkable in vitro antimicrobial and inhibitory potencies of 2-1*H*-pyrazoline-thiadiazole derivative **7** and **13** against *S. aureus* DNA gyrase, a docking simulation was performed using PDB code 2XCT [82]. Redocking of the native ligand, ciprofloxacin, gave an RMSD value of 0.81 Å with an energy score of –10.52 kcal/mol. Both 1,3,4-thiadiazole-1*H*-pyrazolines **7** and **13** showed good fit within *S. aureus* DNA gyrase with high energy scores of –9.98 and –10.33 kcal/mol, respectively (Figure 18A,B). Acetamide oxygens in **7** and **13** exhibited H-bond acceptors with **Gly459** backbone (distances: 2.78 and 2.92 Å, respectively). The sulfur atom of the thiomethyl chain gave H-bonds with the sidechains of **Asp512** in **7** and **Asp510** in **13** (distances: 3.98 and 3.56 Å, respectively). Multiple hydrophobic interactions, such as arene-H, were observed between the centroids of 4-chlorophenyl and benzoyl with **DG9** and **Lys581,** respectively, in **7,** and between the centroids of 4-hydroxyphenyl and triazole with **Arg458** and **His1081,** respectively, in **13**.

#### 2.3.2. Molecular Dynamics Simulations

Molecular dynamics (MD) simulations were performed to investigate the temporal stability and dynamic interaction patterns of the biologically significant protein–ligand complexes. Guided by the experimental findings, compound **13** was selected for MD investigation with ERK2 and RIPK3, while both compounds **7** and **13** were examined in complex with DNA gyrase.

##### MD Simulation of the ERK2-Compound **13** Complex

**Figure 19:**
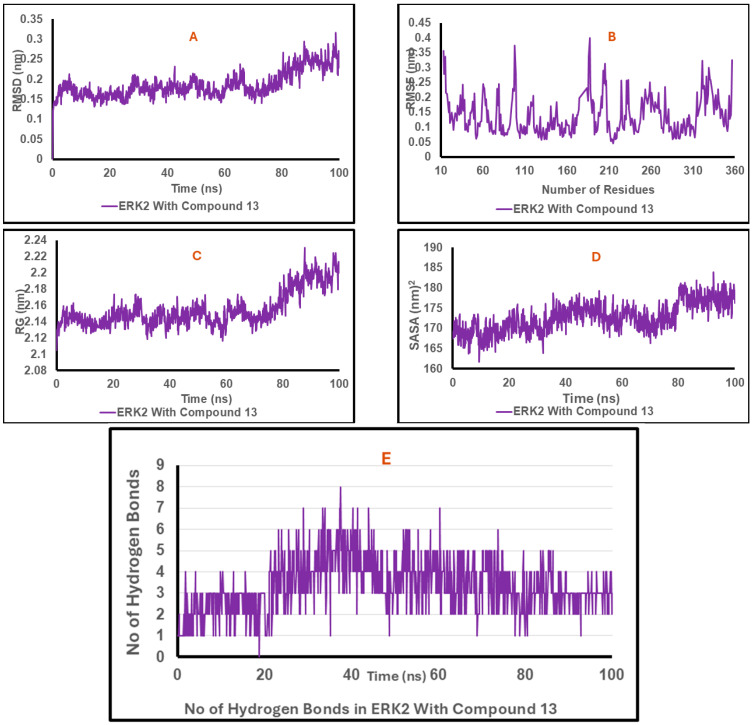
Molecular dynamics analysis of the ERK2-compound **13** complex: (**A**) backbone RMSD, (**B**) residue-wise RMSF, (**C**) radius of gyration (Rg), (**D**) solvent-accessible surface area (SASA), and (**E**) hydrogen bond profile.

The dynamic behavior of the ERK2-compound **13** complex indicated a well-maintained and stable binding mode following the initial equilibration phase. The RMSD profile revealed limited structural deviation, with values remaining within 0.15–0.30 nm, reflecting preservation of the overall protein backbone integrity (Figure 19A). Residue-wise flexibility analysis further supported this stability, as most residues exhibited low RMSF values (0.08–0.20 nm), with higher mobility restricted to terminal and loop regions, while residues lining the binding site remained comparatively rigid (Figure 19B).

Assessment of global compactness demonstrated that ERK2 retained its folded architecture throughout the simulation, as evidenced by stable Rg values ranging from 2.13 to 2.22 nm, with only a slight late-stage increase suggestive of minor conformational breathing rather than structural destabilization (Figure 19C). In parallel, SASA analysis showed consistent solvent exposure, fluctuating narrowly between 166 and 182 nm^2^, with average values around 173–176 nm^2^ and no abrupt increases associated with unfolding events (Figure 19D).

Intermolecular hydrogen bond analysis revealed a dynamic yet persistent interaction pattern between compound **13** and ERK2 (Figure 19E). During the early equilibration period, 1–3 hydrogen bonds were observed, followed by stabilization at 3–5 hydrogen bonds during the mid-simulation phase. Peak hydrogen bonding (6–7 bonds) occurred between 30 and 45 ns, after which the complex maintained an average of 2–4 hydrogen bonds toward the end of the trajectory. Collectively, the RMSD, RMSF, Rg, SASA, and hydrogen bond profiles indicate that compound **13** forms a stable and well-accommodated complex with ERK2, supporting its favorable interaction behavior at the molecular level.

##### Molecular Dynamics Analysis of the RIPK3-Compound **13** Complex

**Figure 20:**
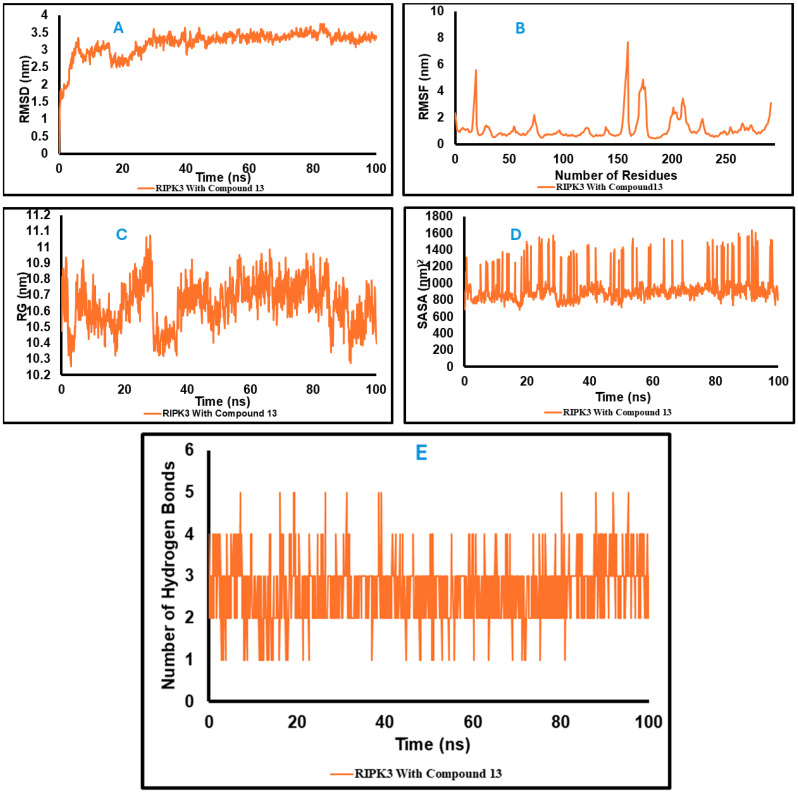
Molecular dynamics analysis of the RIPK3-compound **13** complex. (**A**) RMSD, (**B**) RMSF, (**C**) radius of gyration (Rg), (**D**) solvent-accessible surface area (SASA), and (**E**) hydrogen bond profiles over a 100 ns simulation.

Molecular dynamics (MD) simulations were performed for 100 ns to evaluate the dynamic stability and structural behavior of the RIPK3-compound **13** complex (Figure 20A–E). Protein backbone RMSD analysis (Figure 20A) showed a rapid equilibration during the early stage (0–5 ns), where RMSD increased from ~0.0–0.2 nm to ~2.0–2.3 nm, followed by gradual convergence to a stable plateau after ~20–25 ns. During the production phase (25–100 ns), RMSD values fluctuated narrowly within ~3.0–3.6 nm, with an average of ~3.3–3.5 nm and no progressive drift, indicating a stable protein-ligand complex.

Residue-wise flexibility analysis (RMSF, Figure 20B) indicated that most RIPK3 residues exhibited low to moderate fluctuations (~0.6–1.5 nm), while higher flexibility was restricted to specific regions, including the N-terminal segment (~20–25 residues, ~5.0–5.5 nm) and central loop regions (~150–160 residues, ~7.0–7.5 nm), with additional moderate fluctuations around residues ~170–180 and ~200–215. The radius of gyration (Rg, Figure 20C) remained stable throughout the simulation, fluctuating within ~10.3–11.0 nm and averaging ~10.6–10.8 nm, with only a brief transient compaction event observed around 30–35 ns.

Consistently, solvent-accessible surface area analysis (SASA, Figure 20D) showed stable solvent exposure, with values predominantly within ~800–1000 nm^2^ and transient spikes up to ~1400–1600 nm^2^ attributed to short-lived exposure of flexible regions. Hydrogen bond analysis (Figure 20E) revealed persistent formation of 2–4 intermolecular hydrogen bonds, with occasional transient increases up to 5 bonds and no extended periods of bond loss, indicating continuous ligand engagement. Overall, the combined MD parameters support stable binding of compound **13** to RIPK3 with localized flexibility rather than global destabilization.

##### Molecular Dynamics Analysis of DNA Gyrase Complexes with Compounds **13** and **7**

To rationalize the in vivo anti-MRSA activity of compounds **13** and **7**, molecular dynamics (MD) simulations were conducted on their DNA gyrase complexes to assess binding stability, conformational behavior, and interaction persistence.

##### MD Simulation of the DNA Gyrase-Compound **13** Complex

**Figure 21:**
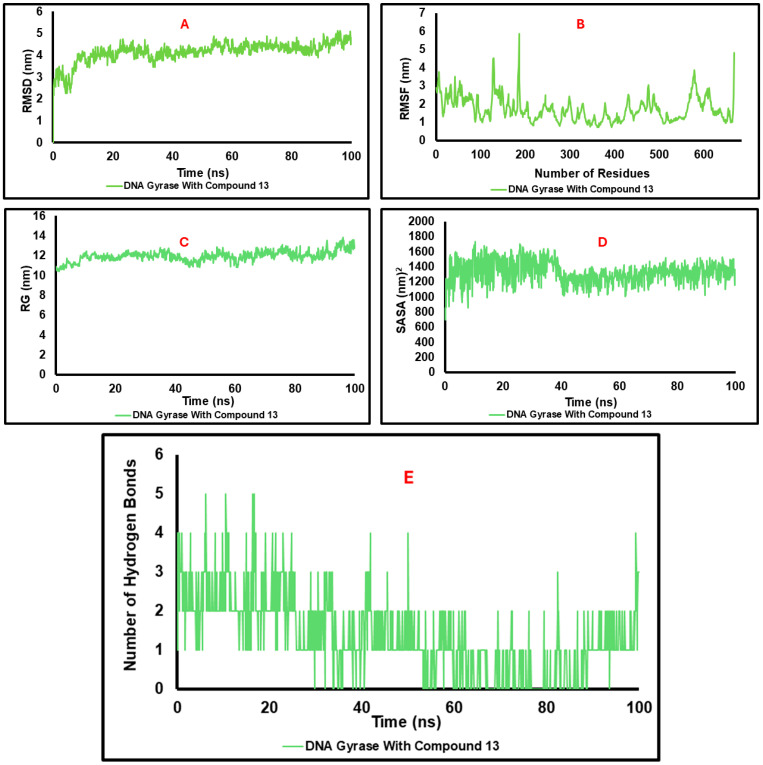
Molecular dynamics simulation analysis of the DNA gyrase-compound **13** complex over 100 ns: (**A**) protein backbone RMSD, (**B**) residue-wise RMSF, (**C**) radius of gyration (Rg), (**D**) solvent-accessible surface area (SASA), and (**E**) number of intermolecular hydrogen bonds.

Molecular dynamics simulations were conducted for 100 ns to assess the conformational stability and dynamic behavior of the DNA gyrase-compound **13** complex (Figure 21A–E). The protein backbone RMSD profile (Figure 21A) exhibited a rapid conformational adjustment during the initial equilibration phase (0–10 ns), where RMSD increased from ~2.5–3.0 nm to ~3.8–4.2 nm, followed by convergence to a stable plateau after ~15–20 ns. During the production phase (20–100 ns), RMSD values fluctuated moderately within ~4.0–4.6 nm, with an average of ~4.2–4.5 nm and no evidence of progressive drift, indicating preservation of global protein stability upon ligand binding.

Residue-wise flexibility analysis (RMSF, Figure 21B) showed that most residues displayed low to moderate fluctuations (~1.0–2.5 nm), while higher flexibility was restricted to discrete regions, including the N-terminal segment (~1–80 residues, ~3.0–3.5 nm), a highly flexible loop around residues ~170–190 (~5.5–6.0 nm), and additional flexible regions near the C-terminus (~580–660 residues). These fluctuations reflect intrinsic loop and terminal dynamics rather than overall structural destabilization.

Global compactness remained largely preserved, as reflected by the radius of gyration profile (Rg, Figure 21C), which fluctuated within ~11.0–13.5 nm and averaged ~12.0–12.5 nm. A slight increase observed toward the final stage of the simulation (80–100 ns) suggests subtle global rearrangements without evidence of protein unfolding. In parallel, SASA analysis (Figure 21D) demonstrated stable solvent exposure, with values predominantly distributed between ~1100 and 1500 nm^2^. Higher fluctuations during early equilibration (~1600–1700 nm^2^) and a transient decrease around ~40–50 ns were attributed to reversible conformational adjustments rather than persistent exposure of the hydrophobic core.

Hydrogen bond analysis (Figure 21E) revealed a dynamic yet persistent interaction pattern between compound **13** and DNA gyrase. An initial prevalence of 2–4 hydrogen bonds (occasionally up to 5) was followed by a more dynamic mid-simulation phase characterized by 0–2 hydrogen bonds, with subsequent stabilization toward the end of the simulation at 1–2 hydrogen bonds. The absence of prolonged interaction loss indicates sustained ligand engagement throughout the trajectory.

Overall, the combined MD parameters support the formation of a dynamically stable and well-equilibrated DNA gyrase-compound **13** complex with localized flexibility.

##### MD Simulation of the DNA Gyrase-Compound **7** Complex

**Figure 22:**
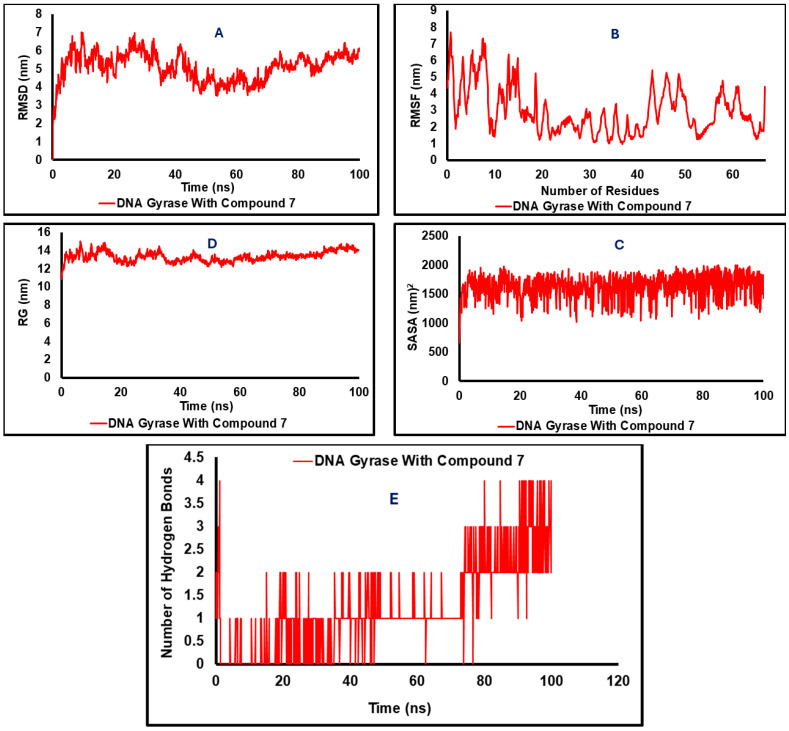
Time-dependent molecular dynamics profiles of the DNA gyrase-compound **7** complex over 100 ns: (**A**) backbone RMSD, (**B**) residue-wise RMSF, (**C**) solvent-accessible surface area (SASA), (**D**) radius of gyration (Rg), and (**E**) evolution of intermolecular hydrogen bonds, highlighting the dynamic binding behavior of compound **7**.

The molecular dynamics trajectory of the DNA gyrase-compound **7** complex (Figure 22A–E) revealed pronounced conformational variability during the simulation. The RMSD profile (Figure 22A) showed a rapid increase during the early equilibration stage (0–5 ns), rising from ~0.0–0.3 nm to ~3.0–3.5 nm, followed by a continued increase reaching ~5.5–6.5 nm within the first 20–30 ns. This behavior reflects substantial conformational rearrangements associated with the intrinsic flexibility and domain motions of DNA gyrase. A partial convergence was observed between ~45 and 65 ns, where RMSD values decreased to ~4.0–4.5 nm, indicating transient structural stabilization. During the later phase (65–100 ns), RMSD values increased again and fluctuated mainly within ~5.0–6.0 nm without continuous drift or divergence, highlighting sustained large-scale dynamics.

Residue-wise flexibility analysis (RMSF, Figure 22B) demonstrated heterogeneous mobility across the protein, with RMSF values ranging from ~1.0 to 7.5 nm. The N-terminal region (residues ~1–15) exhibited the highest flexibility, with peaks of ~6.5–7.5 nm, consistent with solvent-exposed terminal segments. Additional elevated fluctuations were observed within the early sequence (~5–12 residues), whereas the central region (~20–40 residues) displayed comparatively lower RMSF values (~1.2–2.5 nm), suggesting localized and transient stabilization. Increased flexibility reappeared toward the C-terminal region (~45–60 residues), where RMSF values reached ~4.0–5.5 nm, corresponding to flexible loop or domain regions involved in large-scale conformational motions.

Overall, the distribution of RMSF values indicates pronounced localized flexibility rather than uniform structural restraint.

The radius of gyration profile (Rg, Figure 22C) reflected a relatively expanded protein conformation, with values fluctuating within ~12.0–14.5 nm. Following an initial increase during equilibration, Rg values stabilized around ~13.0–13.5 nm for most of the simulation, with moderate fluctuations characteristic of conformational breathing. A slight upward trend toward the later stage (approximately 70–100 ns), where Rg approached ~14.0–14.5 nm, suggests subtle global rearrangements without evidence of unfolding.

Consistent with this behavior, the SASA profile (Figure 22D) showed relatively high and fluctuating values throughout the simulation, predominantly ranging between ~1300 and 1900 nm^2^. After an initial rise during equilibration, SASA values remained elevated (~1600–1750 nm^2^) with continuous fluctuations, indicating persistent solvent exposure of surface regions and the absence of sustained protein compaction.

The hydrogen bond profile (Figure 22E) revealed a time-dependent interaction pattern between DNA gyrase and compound **7**. During the early (0–20 ns) and intermediate (20–70 ns) phases, hydrogen bonding was weak and intermittent, with values mainly between 0 and 1 and occasional transient formation of up to 2 bonds. In contrast, the later stage (70–100 ns) showed increased interaction persistence, with hydrogen bonds stabilizing predominantly between 2 and 3 and sporadic increases up to 4 bonds. This delayed stabilization suggests gradual accommodation of compound **7** within the binding site.

Comparative molecular dynamics analysis demonstrated that compound **13** exhibits superior binding stability toward DNA gyrase compared to compound **7**. Compound **13** showed lower RMSD and RMSF fluctuations, preserved protein compactness, and maintained more persistent hydrogen bonding, whereas compound **7** displayed higher conformational flexibility, increased solvent exposure, and delayed hydrogen bond stabilization. These observations are consistent with the stronger in vivo anti-MRSA activity of compound **13**, supporting its designation as the lead compound.

#### 2.3.3. Quantum Chemical Calculations and Electronic Analysis

##### DFT Calculated Electronic and Reactivity Descriptors of Compounds **1**–**14**

The DFT calculated global reactivity descriptors of compounds **1**–**14** provided detailed insight into their electronic behavior, stability, and chemical reactivity (Table 4). The HOMO energies, indicative of electron-donating ability, ranged from −0.18583 eV (compound **12**) to −0.24586 eV (compound **2**), identifying compound **12** as the strongest electron donor and compound **2** as the least nucleophilic derivative. Conversely, the LUMO energies, reflecting electron-accepting capacity, varied from −0.14888 to −0.19140 eV, with compound **1** exhibiting the lowest LUMO value and thus the strongest electrophilic character.

The HOMO-LUMO energy gap (ΔE), a key indicator of molecular reactivity and kinetic stability, ranged from 0.02562 to 0.09148 eV. Compound **1** displayed the narrowest gap, suggesting enhanced chemical reactivity and reduced kinetic stability, whereas compound **2** exhibited the widest gap, consistent with higher stability and lower reactivity. Compounds **10** and **12** also showed relatively small ΔE values, supporting their elevated reactive potential.

The ionization potential (I) ranged from 0.18583 to 0.24586 eV, indicating that compound **2** required the highest energy for electron removal, while compound **12** was more susceptible to oxidation. Trends in electron affinity (A) and electronegativity (χ) further corroborated these observations, with compound **1** showing the highest χ value (0.20421), reflecting a strong tendency to attract electrons. The chemical hardness (η) was lowest for compound **1** (0.01281), identifying it as the softest and most reactive molecule, whereas compound **2** exhibited the highest hardness (0.04574), indicating greater rigidity and stability. Accordingly, softness (σ) followed an inverse trend, reaching a maximum for compound **1** (78.06).

The electrophilicity index (ω) reached its highest value for compound **1** (ω = 3.26), followed by compound **12** (ω = 1.66), while compound **2** showed the lowest ω value, indicating weaker electrophilic character. Furthermore, the charge-transfer descriptors (ΔNmax and ΔN) revealed that compound **1** possessed the highest charge-transfer capability (ΔN = 7.97), suggesting enhanced charge-transfer capability in intermolecular interactions. Compounds **12** and **10** also exhibited elevated ΔN values, consistent with their enhanced reactivity profiles.

Overall, the DFT analysis identified compound **1** as the most electronically activated and flexible derivative, characterized by minimal ΔE, lowest hardness, highest softness, and maximum electrophilicity, whereas compound **2** emerged as the most stable and least reactive member of the series, underscoring the strong dependence of electronic behavior on structural features.

##### Frontier Molecular Orbital and Electronic Distribution Analysis

DFT orbital analysis revealed a consistent donor acceptor electronic pattern across the investigated derivatives, as illustrated by the HOMO, LUMO, ESP, DOS, and ELF representations (Table S16). The HOMO density was predominantly localized over the hydroxyphenyl–amide–pyrazoline core, identifying this central scaffold as the principal electron donating region. In contrast, the LUMO distribution was mainly shifted toward the thiadiazol-linked heterocyclic tail, indicating that the nature of the substituted heterocycles governs the localization and strength of the electron-accepting domain. This spatial separation of frontier orbitals establishes an efficient intramolecular charge-transfer framework. The corresponding numerical descriptors (E_HOMO, E_LUMO, ΔE, η, σ, χ, ω, and ΔN) for all compounds are summarized in Table S16. Compound **1** exhibited the most pronounced push pull character, where the conjugated acrylamide linker generated the smallest ΔE, highest softness, and largest ΔN values. Consistently, the ESP and ELF maps showed intense electron density localized around the phenolic and amide oxygen atoms, confirming its highly polarizable and electronically activated nature. In compounds **2**–**5**, incorporation of hydrazinecarbonothioyl, mercapto-thiadiazole, cyanomethylthio, and tetrazole moieties resulted in more localized LUMO regions and relatively larger ΔE values, indicating increased electronic stability while retaining moderate reactivity. Compound **6**, bearing a hydrazinyl–iminoethyl substituent, showed a noticeable reduction in ΔE accompanied by increased softness and ΔN values. The corresponding ESP and ELF visualizations revealed multiple electron-rich regions distributed along the thiadiazole–S–(imino–hydrazinyl) chain, highlighting its enhanced interaction potential. In compounds **7**–**12**, functionalization with benzoyl-tetrazole, succinimide–tetrazole, pyrazolidine-3,5-dione, triazole, and hydrazinyl–triazole motifs generated extended nitrogen and carbonyl rich acceptor domains. The DOS and ELF maps clearly indicated that occupied states were primarily derived from the hydroxyphenyl core, whereas unoccupied states and reactive centers were localized within the heterocyclic tail, confirming efficient yet spatially confined charge transfer. Finally, compounds **13** and **14**, bearing S-functionalized thioether derivatives displayed well-defined electronic architectures, characterized by relatively localized LUMO distributions and pronounced ELF maxima around the heteroatom-rich regions. Collectively, compounds **6**, **10**, **12**, **13**, and **14** exhibited efficient donor–acceptor separation, controlled electronic softness, and significant heteroatom density, reflecting balanced electronic distribution and favorable charge delocalization across the molecular framework.

##### Non-Covalent Interaction Analysis Based on Reduced Density Gradient (NCI–RDG) Method

The non covalent interaction (NCI) analysis, performed using the reduced density gradient (RDG) approach, was employed to characterize the nature, strength, and spatial distribution of weak intermolecular interactions within compounds **1**–**14** (Table S17). The sign(λ_2_)ρ versus RDG scatter plots and the corresponding 3D isosurface maps collectively revealed a well-defined interplay of attractive, dispersive, and repulsive interactions governing the overall interaction landscape of the investigated derivatives.

In general, blue regions appearing at negative sign(λ_2_)ρ values were associated with strong attractive interactions, primarily arising from hydrogen bonding and dipole-dipole interactions involving phenolic hydroxyl groups, amide carbonyl functionalities, and heteroatom-rich moieties such as thiadiazole, tetrazole, triazole, and hydrazinyl units. These regions defined localized interaction hotspots and reflected the presence of strong, directional non covalent contacts.

Green regions, centered around sign(λ_2_)ρ values close to zero, corresponded to van der Waals interactions distributed over aromatic rings, the pyrazoline core, and conjugated linkers. These interactions contributed to conformational adaptability and overall structural stabilization without imposing strong directional constraints. In contrast, red regions at positive sign(λ_2_)ρ values were attributed to steric repulsion and were mainly localized around bulky heterocyclic substituents, indicating regulated steric congestion without inducing unfavorable structural strain.

Compounds bearing highly functionalized and heteroatom rich substituents particularly derivatives **6**, **9**, **10**, **12**, **13**, and **14** exhibited more pronounced attractive interaction features, as evidenced by the increased intensity and spatial extension of blue isosurfaces. The corresponding RDG isosurface representations summarized in Table S17 visually confirmed these interaction patterns, where blue, green, and red surfaces consistently represented attractive, dispersive, and repulsive interactions, respectively. Overall, the NCI–RDG analysis highlights the diversity and balance of non-covalent interaction profiles across the compound series, governed by the nature and spatial arrangement of heteroatoms and substituent frameworks.

##### Quantum Rationale for the Extreme DFT Reactivity of Compound **1**

Compound **1** emerged as the most electronically activated derivative within the investigated series based on its density functional theory (DFT) descriptors, reflecting an extreme reactivity profile governed by its structural architecture and functional group distribution. Quantitatively, compound **1** exhibited the smallest HOMO–LUMO energy gap (ΔE = 0.02562 eV), the lowest chemical hardness (η = 0.01281), and the highest softness (σ = 78.06), collectively indicating exceptional electronic flexibility and a strong propensity for charge redistribution. This behavior was further supported by its high electrophilicity index (ω = 3.26) and maximal charge transfer capacity (ΔN = 7.97), confirming its pronounced ability to participate in electron exchange processes.

From a structural perspective, the extreme electronic activation of compound **1** can be directly attributed to its conjugated framework integrating a hydroxyphenyl acetamide moiety, a pyrazoline core, and an electron deficient chlorophenyl–acrylamide segment. The extended π-conjugation, reinforced by the acrylamide linker, facilitates efficient orbital overlap across the molecular backbone, while the presence of both electron donating (phenolic –OH, amide nitrogen) and electron withdrawing (carbonyl groups, chloro substituted aromatic ring) functionalities establishes a strong intramolecular push pull system.

Frontier molecular orbital analysis revealed that the HOMO density is predominantly localized over the hydroxyphenyl–amide–pyrazoline region, highlighting this segment as the principal electron donating domain, whereas the LUMO distribution is shifted toward the acrylamide linked chlorophenyl moiety, identifying it as the main electron accepting region. This pronounced spatial separation between HOMO and LUMO orbitals rationalizes the minimal energy gap and explains the exceptional donor acceptor character of compound **1**. Consistently, electrostatic potential (ESP) maps displayed intense negative potential localized around the phenolic oxygen and amide carbonyl groups, while relatively positive regions were distributed over the conjugated linker and aromatic framework, further confirming strong electronic polarization.

Density of states (DOS) analysis demonstrated a high density of frontier states near the Fermi level, supporting facile electronic excitation and charge transfer, whereas electron localization function (ELF) maps revealed highly delocalized electron density across the conjugated backbone, indicative of strong electronic communication between functional fragments. Complementary NCI-RDG analysis showed extensive blue regions at negative sign(λ_2_)ρ values corresponding to strong attractive interactions, in addition to widespread green regions associated with dispersive van der Waals stabilization, while steric repulsion was minimal and well confined. Collectively, these eight computational descriptors and visualizations converge to depict compound **1** as an electronically overactivated system, where extensive delocalization, strong push pull effects, and maximal charge transfer capacity dominate its quantum mechanistic behavior.

**Figure 23:**
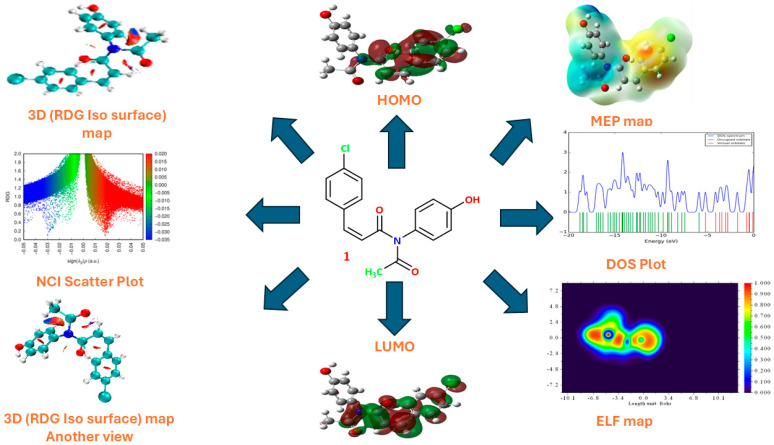
Integrated quantum chemical representations of compound **1**, including HOMO and LUMO distributions, MEP, DOS, ELF, and NCI-RDG analyses, illustrating its highly polarized electronic structure, extensive charge delocalization, and pronounced donor acceptor character, consistent with its extreme theoretical reactivity.

Overall, the DFT and NCI–RDG results unequivocally establish compound **1** as the most theoretically reactive derivative in the series. Its electronic profile is driven by synergistic effects of conjugation, heteroatom rich functionality, and polarized aromatic substitution, resulting in extreme softness, minimal kinetic resistance to electronic excitation, and highly accessible reactive sites. This combination rationalizes why compound **1** represents the upper limit of electronic activation from a purely theoretical standpoint within the investigated framework (Figure 23).

##### Quantum Basis for the Optimized Electronic Profile and Superior Biological Activity of Compound **13**

Compound **13** emerged as the most biologically effective derivative in the investigated series owing to its optimized quantum-mechanical profile rather than excessive electronic activation. Unlike compound **1**, which represents a theoretical maximum in electronic reactivity, compound **13** exhibits a balanced set of DFT descriptors that favor selective and stable target engagement. Quantitatively, compound **13** shows a moderate HOMO–LUMO energy gap (ΔE = 0.06396 eV), intermediate chemical hardness (η = 0.03198), and controlled softness (σ = 31.27), reflecting sufficient electronic flexibility while maintaining kinetic stability. Its electrophilicity index (ω = 1.03) and charge-transfer capability (ΔN = 2.84) further indicate an ability to undergo efficient electronic redistribution without excessive polarization. Structurally, this optimized behavior is governed by the molecular architecture of compound **13**, which incorporates a hydroxyphenyl acetamide moiety, a pyrazoline core, and a thiadiazole-linked fragment connected via a sulfur bridge. Frontier molecular orbital analysis reveals that the HOMO density is predominantly localized over the hydroxyphenyl–amide–pyrazoline segment, identifying this region as the principal electron-donating domain, while the LUMO density is concentrated over the thiadiazole–azole fragment enriched with nitrogen and sulfur atoms, acting as an electron-accepting domain. This clear donor–acceptor separation promotes efficient intramolecular charge transfer while avoiding excessive delocalization. Electrostatic potential (MEP) maps further support this interpretation by showing pronounced negative potential around the phenolic oxygen, amide carbonyl, and azole nitrogen atoms, contrasted with relatively positive regions over aromatic and alkyl fragments, generating strong electrostatic complementarity. Density of states (DOS) analysis confirms the presence of accessible frontier states near the Fermi level, while electron localization function (ELF) maps reveal well-defined electron density localized around heteroatom clusters rather than being diffusely delocalized.

**Figure 24:**
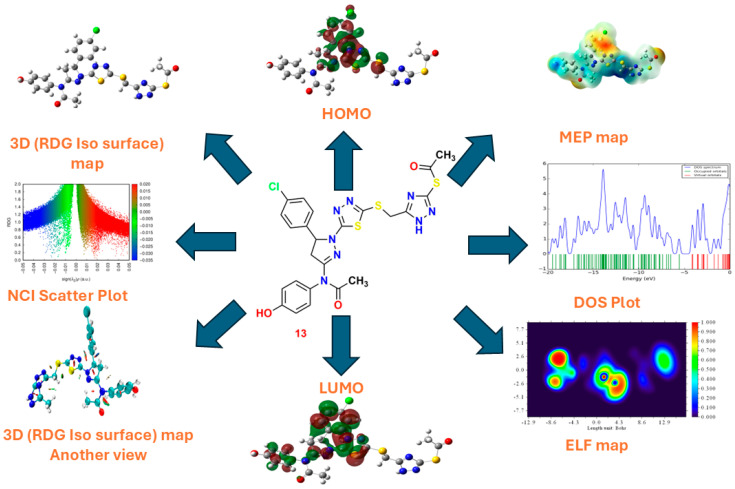
Integrated quantum chemical visualization of compound **13** showing HOMO and LUMO distributions, MEP, DOS, ELF, and NCI-RDG analyses, highlighting its balanced donor acceptor separation, controlled electronic flexibility, and well-organized non-covalent interaction landscape consistent with its superior biological activity.

Complementary NCI-RDG analysis demonstrates that compound **13** possesses intense attractive interaction regions associated with hydrogen bonding and dipole-dipole interactions, accompanied by extensive dispersive stabilization and well-regulated steric repulsion. The molecular framework, although not fused, maintains sufficient conformational organization through conjugated heteroaromatic segments, while the sulfur linkage contributes to electronic communication without imposing excessive rigidity. Collectively, these quantum-chemical descriptors and visual analyses explain why compound **13** achieves superior biological performance: not through maximal reactivity, but via an optimized balance between electronic organization, structural adaptability, and interaction selectivity (Figure 24).

## 3. Experimental

### 3.1. Chemistry

Detailed information regarding the instruments utilized for melting point determination, spectral analyses (IR, mass, ^1^H NMR, and ^13^C NMR), and elemental analysis was provided in the Supplementary Information (SI).

*N*-Acetyl-3-(4-chlorophenyl)-*N*-(4-hydroxyphenyl)acrylamide (**1**)

To a stirred solution of compound **A** (2.73 g, 10 mmol) in absolute ethanol (20 mL), acetyl chloride (0.71 mL, 10 mmol) was added dropwise in the presence of three drops of triethylamine (TEA) as a base catalyst. The reaction mixture was heated under reflux for 2 h, during which the formation of the desired product was confirmed by TLC. After completion, the mixture was allowed to cool naturally to room temperature. The precipitate was filtered, washed with cold ethanol, recrystallized from chloroform, and dried to yield compound **1** as an off-white crystalline powder in 92% yield, m.p. 280–282 °C. IR (KBr, ν_max_ cm^−1^): 3447 (OH), 2984 (C-H aliphatic), 3054, 3093 (C–H, Ar), 1734, 1627 (2C=O, C=C, Ar). ^1^H NMR (400 MHz, DMSO-*d_6_*): δ 2.66 (s, 3H, CH_3_), 7.18–8.05 (m, 10H, Ar–H and 2CH of chalcone), 9.64 (br s, 1H, OH). ^13^C NMR (100 MHz, DMSO-*d_6_*): δ 26.0 (1C, CH_3_), 115.5 (1C, chalcone C=O), 118.1–131.5 (11C, Ar–C), 140.0 (1C, C chalcone–Ph–Cl), 151.0 (1C, C=O–Cl), 153.6 (1C, Ar–C–OH), 161.0 (1C, amide C=O). Anal. Calcd for C_17_H_14_ClNO_3_ (315.07): C, 64.67; H, 4.47; N, 4.44; %. Found C, 64.67; H, 4.51; N, 4.44%.

*N*-[5-(4-Chlorophenyl)-1-(hydrazinecarbonothioyl)-4,5-dihydro-1*H*-pyrazol-3-yl)-*N*-(4-hydroxyphenyl]acetamide (**2**)

A mixture of compound **1** (3.15 g, 10 mmol) and hydrazinecarbothiohydrazide (1.06 g, 10 mmol) in absolute ethanol (20 mL) was treated with a few drops of glacial AcOH and refluxed for 6 h. The mixture was then cooled to room temperature, concentrated under reduced pressure, and the obtained solid was filtered, washed with cold ethanol, recrystallized from benzene, and dried to yield compound **2** as a yellow crystals in 75% yield, m.p. 288–290 °C. IR (KBr, ν_max_ cm^−1^): 3417 (OH), 3297, 3160 (NH_2_, NH), 3030 (C–H, Ar), 2978, 2918 (C–H, aliphatic), 1733 (C=O), 1668 (C=N), 1640 (C=C, Ar), 1106 (C=S).

^1^H NMR (400 MHz, DMSO-*d_6_*): δ 2.05 (d, 2H, CH_2_, pyrazole), 2.68 (s, 3H, CH_3_), 3.61 (s, 2H, NH_2_, D_2_O exchangeable), 4.58 (t, 1H, CH, pyrazole), 6.33–7.86 (m, 8H, Ar-H), 8.52 (s, 2H, NH_2_, D_2_O exchangeable), 9.61 (br s, 1H, OH). ^13^C NMR (100 MHz, DMSO-*d_6_*): δ 26.0 (1C, CH_3_), 33.8 (CH_2_, pyrazole), 59.1 (CH, pyrazole), 100.0–128.3 (12C, Ar-C), 140.7 (C=N, pyrazole), 151.0 (C=O), 155.9 (Ar-C-OH), 183.1 (C=S). Anal. Calcd for C_18_H_18_ClN_5_O_2_S (403.09): C, 53.53; H, 4.49; N, 17.34%. Found C, 53.55; H, 4.49; N, 17.36%.

*N*-[5-(4-Chlorophenyl)-1-(5-mercapto-1,3,4-thiadiazol-2-yl)-4,5-dihydro-1*H*-pyrazol-3-yl]-*N*-(4-hydroxyphenyl)acetamide (**3**)

Compound **2** (4.03 g, 10 mmol) was dissolved in absolute ethanol (20 mL), followed by the slow addition of carbon disulfide (0.60 mL, 10 mmol) in the presence of potassium hydroxide KOH (0.56 g, 10 mmol). The resulting mixture was refluxed for 5 h, during which the reaction progress was tracked by thin-layer chromatography (TLC). Upon completion, the hot reaction solution was poured over crushed ice, producing a dense dark yellow precipitate that was immediately collected by filtration. The solid was thoroughly washed with cold water, dried, and recrystallized from ethanol to give compound **3** in 80% yield, m.p. 298–300 °C. IR (KBr, ν_max_ cm^−1^): 3424 (OH), 3040 (C–H, Ar), 2981, 2924 (C–H, aliphatic), 2560 (SH), 1734 (C=O), 1638 (C=N, C=C, Ar). ^1^H NMR (400 MHz, DMSO-*d_6_*): δ 2.01 (d, 2H, CH_2_, pyrazole), 2.70 (s, 3H, CH_3_), 4.52 (t, 1H, CH, pyrazole), 6.62–8.19 (m, 8H, Ar–H), 9.61 (br s, 1H, OH), 12.61 (s, 1H, SH). ^13^C NMR (100 MHz, DMSO-*d_6_*): δ 26.5 (1C, CH_3_), 34.9 (CH_2_, pyrazole), 59.4 (CH, pyrazole), 100.0–134.8 (11C, Ar–C), 141.1 (C=N, pyrazole), 151.0 (C=O), 153.6 (Ar–C–OH), 173.3 (C=N, thiadiazole–N), 180.9 (C=N, thiadiazole–SH). MS: *m*/*z* = 445.04 [M+], base peak: 364 (100%). Anal. Calcd for C_19_H_16_ClN_5_O_2_S_2_ (445.04): C, 51.17; H, 3.62; N, 15.71%. Found C, 51.18; H, 3.65; N, 15.71%.

*N*-{{5-(4-Chlorophenyl)-1-{5-[(cyanomethyl)thio]-1,3,4-thiadiazol-2-yl}-4,5-dihydro-1*H*-pyrazol-3-yl}}-*N*-(4-hydroxyphenyl)acetamide (**4**)

In a round-bottom flask, compound **3** (4.45 g, 10 mmol) was dissolved in absolute ethanol (10 mL), followed by the addition of chloroacetonitrile (0.66 mL, 10 mmol) and a three drops of triethylamine (TEA). The reaction mixture was then subjected to reflux for 8 h, during which the progress of the reaction was periodically monitored by TLC. Upon completion, the mixture was allowed to cool to room temperature and slowly poured onto crushed ice, producing a dark gray precipitate. The obtained solid was filtered, thoroughly washed with cold water, recrystallized from DCM, and dried under vacuum to yield compound **4** in 84% yield, m.p. 276–278 °C. IR (KBr, ν_max_ cm^−1^): 3410 (OH), 3074 (C–H, Ar), 2977, 2921 (C–H, aliphatic), 2208 (C≡N), 1727 (C=O), 1658 (C=N, C=C, Ar). ^1^H NMR (400 MHz, DMSO-*d_6_*): δ 2.05 (d, 2H, CH_2_, pyrazole), 2.69 (s, 3H, CH_3_), 3.78 (s, 2H, CH_2_–CN), 4.48 (t, 1H, CH, pyrazole), 7.18–7.84 (m, 8H, Ar–H), 9.61 (br s, 1H, OH). ^13^C NMR (100 MHz, DMSO-*d_6_*): δ 18.0 (CH_2_–CN), 26.5 (1C, CH_3_), 36.3 (CH_2_, pyrazole), 56.8 (CH, pyrazole), 101.8–134.3 (11C, Ar–C), 118.0 (CN), 143.6 (C=N, pyrazole), 150.8 (C=O), 153.0 (Ar–C–OH), 160.2 (C=N, thiadiazole–S), 176.5 (C=N, thiadiazole–N). Anal. Calcd for C_21_H_17_ClN_6_O_2_S_2_ (484.05): C, 52.01; H, 3.53; N, 17.33%. Found C, 52.00; H, 3.53; N, 17.34%.

*N*-{{{1-{{5-{[(2*H*-Tetrazol-5-yl)methyl]thio}-1,3,4-thiadiazol-2-yl}}-5-(4-chlorophenyl)-4,5-dihydro-1*H*-pyrazol-3-yl}}}-*N*-(4-hydroxyphenyl)acetamide (**5**)

A suspension of compound **4** (4.84 g, 10 mmol) and sodium azide (0.65 g, 10 mmol) in absolute ethanol (10 mL) was treated with ammonium chloride (0.53, 10 mmol) as a mild basic catalyst. The reaction mixture was refluxed for 6 h, and its progress was monitored by TLC. After completion, the mixture was cooled to room temperature and poured onto crushed ice, leading to the formation of a solid, which was collected by filtration, washed thoroughly with cold water, recrystallized from methanol, and dried under vacuum to yield compound **5** as a bright black powder in 89% yield, m.p. 288–290 °C. IR (KBr, ν_max_ cm^−1^): 3480 (OH), 3216 (NH), 3080 (C–H, Ar), 2982, 2924, 2853 (C–H, aliphatic), 1725 (C=O), 1629 (C=N), 1595 (C=C, Ar), 1504 (N=N). ^1^H NMR (400 MHz, DMSO-*d_6_*): δ 2.03 (d, 2H, CH_2_, pyrazole), 2.62 (s, 3H, CH_3_), 4.52 (s, 2H, CH_2_–S), 4.76 (t, 1H, CH, pyrazole), 7.06–8.02 (m, 8H, Ar–H), 9.75 (br s, 1H, OH), 12.42 (s, 1H, NH, D_2_O exchangeable). ^13^C NMR (100 MHz, DMSO-*d_6_*): δ 26.3 (1C, CH_3_), 29.7 (CH_2_–S), 30.7 (CH_2_, pyrazole), 55.5 (CH, pyrazole), 114.3–135.2 (11C, Ar–C), 143.8 (C=N, pyrazole), 150.2 (C=O), 153.3 (Ar–C–OH), 160.3 (C=N, thiadiazole–S), 162.3 (C=N, tetrazole), 176.3 (C=N, thiadiazole–N). MS: *m*/*z* = 527.07 [M+], base peak: 482 (100%). Anal. Calcd for C_21_H_18_ClN_9_O_2_S_2_ (527.07): C, 47.77; H, 3.44; N, 23.88%. Found C, 47.78; H, 3.48; N, 23.87%.

*N*-{{5-(4-Chlorophenyl)-1-{5-[(2-hydrazinyl-2-iminoethyl)thio]-1,3,4-thiadiazol-2-yl}-4,5-dihydro-1*H*-pyrazol-3-yl}}-*N*-(4-hydroxyphenyl)acetamide (**6**)

To a boiling ethanolic solution of compound **4** (4.84 g, 10 mmol) in (8 mL) EtOH, hydrazine hydrate (1.21 mL, 25 mmol) was added gradually with continuous stirring. The reaction mixture was heated under reflux for 4 h, during which progress was monitored by TLC. Upon completion, the hot mixture was allowed to cool to room temperature. The solid product that formed was filtered, washed thoroughly with cold ethanol, recrystallized from toluene, and dried to yield compound **6** as a white crystalline solid in 77% yield, m.p. 280–282 °C. IR (KBr, ν_max_ cm^−1^): 3483 (OH), 3263–3200 (NH_2_), 3020 (CH–Ar), 2985, 2922, 2851 (CH–aliphatic), 1741 (C=O), 1716 (C=NH), 1676 (C=N, multiple), 1643 (C=C–Ar). ^1^H NMR (DMSO-*d_6_*, 400 MHz, δ ppm): δ 2.12 (d, 2H, CH_2_–pyrazole), 2.59 (s, 3H, CH_3_), 3.13 (s, 2H, CH_2_–S), 3.39 (s, 2H, NH_2_, D_2_O exchangeable), 4.22 (t, 1H, CH–pyrazole), 4.27 (s, 1H, NH, D_2_O exchangeable), 7.56–8.08 (m, 8H, Ar–H), 9.17 (s, 1H, CH=NH, D_2_O exchangeable), 9.66 (br s, 1H, OH). ^13^C NMR (DMSO-*d_6_*, 100 MHz, δ ppm): δ 26.8 (1C, CH_3_), 31.8 (CH_2_–diazole), 34.2 (CH_2_–S), 55.5 (CH–pyrazole), 115.8–135.7 (C–Ar), 144.4 (C=N–pyrazole), 151.1 (C=O), 153.1 (Ar–C–OH), 159.6 (C=NH), 160.0 (C=N–thiadiazole–S), 176.1 (C=N–thiadiazole–N). Anal. Calcd for C_21_H_21_ClN_8_O_2_S_2_ (516.09): C, 48.79; H, 4.09; N, 21.67%. Found C, 48.81; H, 4.09; N, 21.67%.

*N*-{{{1-{{5-{[(2-Benzoyl-2*H*-tetrazol-5-yl)methyl]thio}-1,3,4-thiadiazol-2-yl}}-5-(4-chlorophenyl)-4,5-dihydro-1*H*-pyrazol-3-yl}}}-*N*-(4-hydroxyphenyl)acetamide (**7**)

An equimolar mixture of compound **5** (5.28 g, 10 mmol) and benzoyl chloride (1.16 mL, 10 mmol) was stirred in absolute ethanol (20 mL), in the presence of three drops triethylamine (TEA) as a base for 24 h at room temperature. The progress of the reaction was tracked by TLC until completion. Subsequently, the reaction mixture was cooled and carefully poured onto crushed ice, leading to the formation of a precipitate. The obtained solid was filtered, washed several times with cold ethanol, recrystallized from DCM, and dried under vacuum to afford compound **7** as a dark violet crystalline powder in 86% yield, m.p. > 300 °C. IR (KBr, ν_max_, cm^−1^): 3489 (OH), 3066, 3006 (CH–Ar), 2926, 2856 (CH–aliphatic), 1727, 1674 (2C=O), 1659 (4C=N), 1606 (C=C–Ar), 1450 (N=N, tetrazole), 1019–1084 (tetrazole ring). ^1^H NMR (DMSO-*d_6_*, 400 MHz, δ ppm): δ 2.05 (d, 2H, CH_2_–pyrazole), 2.68 (s, 3H, CH_3_), 3.78 (s, 2H, CH_2_–CN), 4.48 (t, 1H, CH–pyrazole), 7.18–7.84 (m, 8H, Ar–H), 9.61 (br s, 1H, OH). ^13^C NMR (DMSO-*d_6_*, 100 MHz, δ ppm): δ 26.0 (1C, CH_3_), 30.5 (CH_2_–S), 34.0 (CH_2_–pyrazole), 58.2 (CH–pyrazole), 119.8–137.2 (C–Ar, 17C), 144.0 (C=N–pyrazole), 150.2 (C=O, acyl, 2C), 153.3 (Ar–C–OH), 160.2 (C=N–thiadiazole–S), 162.8 (C=N–tetrazole), 168.3 (C=O–Ph), 176.1 (C=N–thiadiazole–N). Anal. Calcd for C_28_H_22_ClN_9_O_3_S_2_ (631.10): C, 53.20; H, 3.51; N, 19.94%. Found C, 53.20; H, 3.52; N, 19.96%.

*N*1-(5-(((5-(5-(4-Chlorophenyl)-3-(*N*-(4-hydroxyphenyl)acetamido)-4,5-dihydro-1*H*-pyrazol-1-yl)-1,3,4-thiadiazol-2-yl)thio)methyl)-2*H*-tetrazol-2-yl)succinamide (**8**)

To a well-stirred ethanolic suspension of compound **5** (5.28 g, 10 mmol) in (10 mL) EtOH was added *N*1-bromosuccinamide (1.95 g, 10 mmol) in the presence of anhydrous potassium carbonate (1.38 g, 10 mmol) as a mild base. The reaction mixture was subjected to stirring for 9 h at room temperature, during which the progress was monitored by TLC until complete consumption of the starting material. Upon cooling, the dark solution was slowly poured onto crushed ice, resulting in the immediate formation of a dense red precipitate. The solid product was filtered, thoroughly washed with cold ethanol, dried under reduced pressure, and recrystallized from ethanol to furnish compound **8** in 86% yield. m.p. > 300 °C, IR (KBr, ν_max_ cm^−1^): 3421 (OH), 3098, 3084 (CH–Ar), 2987, 2921 (CH–aliphatic), 1747, 1704 (2C=O), 1655, 1638 (3C=N), 1549 (C=C–Ar). ^1^H NMR (DMSO-*d_6_*, 400 MHz, δ ppm): δ 2.10 (d, 2H, CH_2_–pyrazole), 2.34 (t, 4H, 2CH_2_–C=O), 2.65 (s, 3H, CH_3_), 4.51 (s, 2H, CH_2_–S), 4.77 (t, 1H, CH–pyrazole), 6.61 (s, 1H, NH, D_2_O exchangeable), 7.22 (s, 2H, NH_2_, D_2_O exchangeable), 6.63–8.46 (m, 8H, Ar–H), 9.92 (br s, 1H, OH). ^13^C NMR (DMSO-*d_6_*, 100 MHz, δ ppm): δ 26.2 (1C, CH_3_), 29.7 (CH_2_–S), 30.7 (CH_2_–diazole), 35.6, 35.8 (2CH_2_–C=O), 55.5 (CH–pyrazole), 114.3–135.2 (C–Ar, 11C), 143.8 (C=N–pyrazole), 150.2 (C=O–acyl), 153.3 (Ar–C–OH), 159.9 (C=N–thiadiazole–S), 162.0 (C=N–tetrazole), 176.1 (C=N–thiadiazole–N), 179.0, 179.9 (2C=O–amide). Anal. Calcd for C_25_H_24_ClN_11_O_4_S_2_ (641.11): C, 46.76; H, 3.77; N, 24.00%. Found C, 46.78; H, 3.77; N, 24.01%.

*N*-(5-(4-chlorophenyl)-1-(5-((2-(3,5-dioxopyrazolidin-1-yl)-2-iminoethyl)thio)-1,3,4-thiadiazol-2-yl)-4,5-dihydro-1*H*-pyrazol-3-yl)-*N*-(4-hydroxyphenyl)acetamide (**9**)

A mixture of compound **6** (5.17 g, 10 mmol) and diethyl malonate (1.51 mL, 10 mmol) was refluxed in absolute ethanol (10 mL) in the presence of triethylamine as a base for 10 h. Upon completion (TLC monitoring), the hot reaction mixture was allowed to cool to room temperature, then poured onto crushed ice. The solid precipitate formed was filtered, washed thoroughly with cold ethanol, recrystallized from diethyl ether, and dried to afford compound **9** as a dark red crystalline powder in 69% yield, m.p. 266–268 °C. IR (KBr, ν_max_, cm^−1^): 3477 (OH), 3335 (NH), 3086, 3006 (CH–Ar), 2988, 2957, 2917, 2848 (CH–aliphatic), 1725, 1700 (C=NH, 3C=O), 1660 (3C=N), 1589 (C=C–Ar). ^1^H NMR (DMSO-*d_6_*, 400 MHz, δ ppm): δ 2.00 (s, 2H, CH_2_–pyrazolidine-3,5-dione), 2.15 (d, 2H, CH_2_–pyrazole), 2.69 (s, 3H, CH_3_), 3.15 (s, 2H, CH_2_–S), 4.25 (t, 1H, CH–pyrazole), 7.26–7.47 (m, 8H, Ar–H), 9.08 (s, 1H, NH=C, D_2_O exchangeable), 9.68 (br s, 1H, OH), 11.26 (s, 1H, NH–pyrazolidine-3,5-dione, D_2_O exchangeable). ^13^C NMR (DMSO-*d_6_*, 100 MHz, δ ppm): δ 26.4 (1C, CH_3_), 30.7 (CH_2_–diazole), 35.6 (CH_2_–S), 45.5 (CH_2_–pyrazolidine-3,5-dione), 56.0 (CH–pyrazole), 114.3–139.8 (12C, C–Ar), 145.0 (C=N–pyrazole), 150.1 (C=O), 153.3 (Ar–C–OH), 159.1 (C=NH), 162.3 (C=N–thiadiazole–S), 170.0, 172.6 (2C=O–pyrazolidine-3,5-dione), 176.1 (C=N–thiadiazole–N). Anal. Calcd for C_24_H_21_ClN_8_O_4_S_2_ (584.08): C, 49.27; H, 3.62; N, 19.15%. Found C, 49.28; H, 3.62; N, 19.16%.

*N*-(5-(4-Chlorophenyl)-1-(5-(((3-mercapto-1*H*-1,2,4-triazol-5-yl)methyl)thio)-1,3,4-thiadiazol-2-yl)-4,5-dihydro-1*H*-pyrazol-3-yl)-*N*-(4-hydroxyphenyl)acetamide (**10**)

A stirred mixture of compound **6** (5.17 g, 10 mmol) and carbon disulfide (0.6 mL, 10 mmol) was treated with potassium hydroxide (0.56 g, 10 mmol) in absolute ethanol (25 mL) and subjected to reflux for 5 h. The progress of the reaction was monitored by TLC using an ethyl acetate–n-hexane system. Upon completion, the reaction mixture was allowed to cool to room temperature, poured over crushed ice, and the separated solid was filtered, washed thoroughly with water, and dried. The obtained pale brown powder (compound **10**) was recrystallized from ethanol, affording a 71% yield, m.p. 245–247 °C. IR (KBr, cm^−1^): 3475 (OH), 3402 (NH), 3077, 3007 (CH–Ar), 2957, 2917, 2848 (CH–aliphatic), 2558 (SH), 1732 (C=O), 1664, 1612 (C=N), 1592 (C=C, Ar). ^1^H NMR (400 MHz, DMSO-*d_6_*): δ 2.01 (d, 2H, CH_2_–pyrazole), 2.66 (s, 3H, CH_3_), 3.94 (s, 2H, CH_2_–S), 4.20 (t, 1H, CH–pyrazole), 7.06–8.02 (m, 8H, Ar–H), 9.65 (br s, 1H, OH), 12.12 (s, 1H, SH), 12.72 (s, 1H, NH, D_2_O exchangeable). ^13^C NMR (100 MHz, DMSO-*d_6_*): δ 26.6 (1C, CH_3_), 29.9 (CH_2_–S), 31.4 (CH_2_–diazole), 53.0 (CH–pyrazole), 114.3–135.0 (C–Ar), 142.5 (C=N–pyrazole), 145.0 (C=N–triazole–SH), 151.0 (C=O), 153.0 (Ar–C–OH), 160.0 (C=N–thiadiazole–S), 163.0 (C–triazole–NH), 176.1 (C=N–thiadiazole–N). MS: *m*/*z* = 558.05 [M+], base peak: 166 (100%). Anal. Calcd for C_22_H_19_ClN_8_O_2_S_3_ (558.05): C, 47.26; H, 3.43; N, 20.04%. Found C, 47.28; H, 3.46; N, 20.04%.

*N*-(1-(5-((2-((2-Chloroethyl)imino)-2-(3,5-dioxopyrazolidin-1-yl)ethyl)thio)-1,3,4-thiadiazol-2-yl)-5-(4-chlorophenyl)-4,5-dihydro-1*H*-pyrazol-3-yl)-*N*-(4-hydroxyphenyl)acetamide (**11**)

Compound **9** (5.85 g, 10 mmol) was dissolved in absolute ethanol (25 mL), followed by the addition of 1,2-dichloroethane (0.79 mL, 10 mmol) and three drops of triethylamine as a base catalyst. The resulting mixture was stirred for 7 h room temperature, and the reaction progress was monitored by thin-layer chromatography (TLC) using an ethyl acetate/n-hexane (2:1) solvent system. After completion, the mixture was allowed to cool to room temperature, poured onto crushed ice, and the precipitated product was filtered, washed thoroughly with water, and dried. The obtained white solid that was recrystallized from ethanol, yielding compound **11** in 66% yield, m.p. 232–234 °C. IR (KBr, cm^−1^): 3477 (OH), 3335 (NH), 3086, 3026 (CH–Ar), 2958, 2917, 2848 (CH–aliphatic), 1726 (C=O), 1668, 1655 (C=N), 1593 (C=C, Ar). ^1^H NMR (400 MHz, DMSO-*d_6_*): δ 1.75 (t, 2H, CH_2_–N), 2.13 (d, 2H, CH_2_–pyrazole), 2.58 (s, 5H, CH_3_, CH_2_–pyrazolidine-dione), 3.05 (t, 2H, CH_2_–Cl), 3.13 (s, 2H, CH_2_–S), 4.22 (t, 1H, CH–pyrazole), 7.56–8.08 (m, 8H, Ar–H), 9.77 (br s, 1H, OH), 11.75 (s, 1H, NH, D_2_O exchangeable). ^13^C NMR (100 MHz, DMSO-*d_6_*): δ 26.7 (1C, CH_3_), 30.7 (CH_2_–pyrazole), 35.6 (CH_2_–S), 40.0, 40.3 (2CH_2_), 48.9 (CH_2_–pyrazolidine-dione), 55.5 (CH–pyrazole), 115.8–135.7 (C–Ar), 143.8 (C=N–pyrazole), 150.2 (C=O–acyl), 153.5 (Ar–C–OH), 156.5 (C=N), 160.3 (C=N–thiadiazole–S), 171.3, 173.2 (2 C=O–pyrazolidine-dione), 176.4 (C=N–thiadiazole–N). MS: *m*/*z* = 646.07 [M+], base peak: 302 (100%). Anal. Calcd for C_26_H_24_Cl_2_N_8_O_4_S_2_ (646.07): C, 48.23; H, 3.74; N, 17.30%. Found C, 48.23; H, 3.74; N, 17.30%.

*N*-(5-(4-Chlorophenyl)-1-(5-(((3-hydrazinyl-1*H*-1,2,4-triazol-5-yl)methyl)thio)-1,3,4-thiadiazol-2-yl)-4,5-dihydro-1*H*-pyrazol-3-yl)-*N*-(4-hydroxyphenyl)acetamide (**12**)

A solution of compound **10** (5.59 g, 10 mmol) was dissolved in absolute ethanol (8 mL), followed by the dropwise addition of hydrazine hydrate (1.94 mL, 40 mmol; typically 80% *w*/*w* N_2_H_4_ in water) under continuous stirring at room temperature. The mixture was then heated under reflux for 4 h, and the reaction progress was monitored by TLC (ethyl acetate:n-hexane, 3:1). After completion, the reaction mass was cooled to room temperature and poured onto crushed ice. The resulting precipitate was collected by filtration, washed thoroughly with cold ethanol, recrystallized from diethyl ether chloroform, and dried to give compound **12** as a white crystalline powder. Yield: 73%; m.p. 288–290 °C (decomp.) IR (KBr, cm^−1^): 3477 (OH), 3412, 3257, 3196 (NH_2_, 2NH), 3077, 3008 (CH–Ar), 2957, 2917, 2848 (CH–aliphatic), 1722 (C=O), 1664, 1612 (4 × C=N), 1592 (C=C Ar). ^1^H NMR (400 MHz, DMSO-*d_6_*): δ 2.31 (d, 2H, CH_2_-pyrazole), 2.69 (s, 3H, CH_3_), 3.50 (s, 2H, CH_2_-S), 4.22 (t, 1H, CH-pyrazole), 4.50 (s, 2H, NH_2_, D_2_O exchangeable), 7.28–7.51 (m, 8H, Ar-H), 9.22 (s, 1H, NH, D_2_O exchangeable), 9.51 (br s, 1H, OH), 14.15 (s, 1H, NH-triazole, D_2_O exchangeable). ^13^C NMR (100 MHz, DMSO-*d_6_*): δ 25.9 (1C, CH_3_), 29.2 (CH_2_-S), 31.1 (CH_2_-diazole), 52.2 (CH-pyrazole), 116.1–139.7 (C-Ar), 140.0 (Ar-C-CH-pyrazole), 146.1 (C=N-pyrazole), 146.3 (C=N-triazole-NH), 151.1 (C=O), 156.3 (Ar-C-OH), 160.1 (C=N-thiadiazole-S), 163.2 (C-triazole-NH), 176.5 (C=N-thiadiazole-N). MS: *m*/*z* = 556.10 [M+], base peak: 302 (100%). Anal. Calcd for C_22_H_21_ClN_10_O_2_S_2_ (556.10): C, 47.44; H, 3.80; N, 25.15%. Found C, 47.43; H, 3.80; N, 25.14%.

*S*-(5-(((5-(5-(4-Chlorophenyl)-3-(*N*-(4-hydroxyphenyl)acetamido)-4,5-dihydro-1*H*-pyrazol-1-yl)-1,3,4-thiadiazol-2-yl)thio)methyl)-1*H*-1,2,4-triazol-3-yl) ethanethioate (**13**)

A mixture of compound **10** (5.59 g, 10 mmol) was treated with an excess of glacial acetic acid (30 mL), which functioned simultaneously as both solvent and reagent, promoting intramolecular cyclization. The reaction mixture was refluxed for 8 h under continuous stirring, and its progress was monitored by TLC. After completion, the mixture was allowed to cool to room temperature and then poured slowly onto crushed ice. The resulting precipitate was filtered, washed several times with cold ethanol, recrystallized from diethyl ether chloroform, and dried to give compound **13** as a white crystalline powder in 67% yield; m.p. 244–246 °C. IR (KBr, cm^−1^): 3399 (ν OH), 3169 (ν NH), 3074 (ν CH–Ar), 2990, 2964, 2926 (ν CH–aliphatic), 1732 (ν 2C=O), 1648 (ν C=N), 1634 (ν C=C Ar). ^1^H NMR (DMSO-*d_6_*, 400 MHz, δ ppm): 2.20 (s, 3H, CH_3_), 2.51 (d, 2H, CH_2_ pyrazole), 2.66 (s, 3H, CH_3_), 4.30 (s, 2H, CH_2_–S), 5.01 (t, 1H, CH pyrazole), 6.94–7.47 (m, 8H, Ar–H), 9.60 (br s, 1H, OH), 12.25 (s, 1H, NH pyrazole, D_2_O exchangeable). ^13^C NMR (DMSO-*d_6_*, 100 MHz, δ ppm): 24.3, 25.3 (2C, 2CH_3_), 28.3 (CH_2_–S), 32.5 (CH_2_ diazole), 53.3 (CH pyrazole), 114.2–139.1 (11C, C–Ar), 146.0, 146.1 (2C=N pyrazole), 151.6 (C=O), 154.6 (Ar–C–OH), 157.0 (C=N thiadiazole–S), 164.3 (C=N triazole), 166.0 (C=N thiadiazole–N), 170 (1C, C=O-S). Anal. Calcd for C_24_H_21_ClN_8_O_3_S_3_ (601.12): C, 47.95; H, 3.52; N, 18.64%. Found C, 47.01; H, 3.54; N, 18.64%.

*S*-(5-(((5-(5-(4-Chlorophenyl)-3-(*N*-(4-hydroxyphenyl)acetamido)-4,5-dihydro-1*H*-pyrazol-1-yl)-1,3,4-thiadiazol-2-yl)thio)methyl)-1*H*-1,2,4-triazol-3-yl) 2-chloroethanethioate (**14**)

A mixture of compound **10** (5.59 g, 10 mmol) and chloroacetyl chloride (0.78 mL, 10 mmol) was refluxed in xylene (30 mL) for 9 h. In this reaction, chloroacetyl chloride served as cyclizing agent, while xylene acted as a high-boiling solvent facilitating the ring closure. The progress of the reaction was monitored by TLC. Upon completion, the reaction mixture was cooled to room temperature, concentrated under reduced pressure, and the resulting solid was filtered, washed with cold ethanol, recrystallized from diethyl ether petroleum ether, and dried to yield compound **14** as a black powder in 80% yield; m.p. > 300 °C. IR (KBr, cm^−1^): 3482 (ν OH), 3423, (ν NH), 3075, 3055 (ν CH–Ar), 2926, 2856 (ν CH–aliphatic), 1757, 1679 (ν 2C=O), 1617 (ν C=N), 1515, 1449 (ν C=C Ar). ^1^H NMR (DMSO-*d_6_*, 400 MHz, δ ppm): 2.21 (s, 3H, CH_3_), 2.38 (d, 2H, CH_2_ pyrazole), 3.89 (s, 2H, CH_2_–S), 4.53 (s, 2H, CH_2_-Cl), 4.82 (t, 1H, CH pyrazole), 7.16–7.52 (m, 8H, Ar–H), 9.63 (br s, 1H, OH), 12.12 (s, 1H, NH, D_2_O exchangeable). ^13^C NMR (DMSO-*d_6_*, 100 MHz, δ ppm): 26.6 (1C, CH_3_), 30.0 (CH_2_ diazole), 33.1 (CH_2_–S), 48.0 (1C, CH_2_-Cl), 55.1 (CH pyrazole), 113.9–139.1 (11C, C–Ar), 145.1, 146.60 (2C=N), 151.4 (C=O), 154.0 (Ar–C– OH), 156.1 (C=N thiadiazole–S), 163.1 (C=N triazole), 164.7 (C=N thiadiazole–N), 176.1 (1C, C=O-S). Anal. Calcd for C_24_H_20_C_l2_N_8_O_3_S_3_ (634.02): C, 45.36; H, 3.17; N, 17.63%. Found C, 45.35; H, 3.17; N, 17.60%.

### 3.2. Biological Activity

All in vitro experiments were comprehensively detailed in the Supplementary Material.

#### 3.2.1. Assessment of Antiproliferative Activity

The antiproliferative activity of the synthesized 2-1*H*-pyrazoline–thiadiazole derivatives (**1**–**14**) was evaluated against HeLa, HepG-2, MCF-7, and WI-38 cell lines using the MTT colorimetric assay [83,84,85,86,87]. The half-maximal inhibitory concentration (IC_50_) values were calculated through nonlinear dose-response curve analysis and compared with those of doxorubicin and sorafenib. Comprehensive experimental details, including cell seeding densities, incubation parameters, reagent preparation, and microplate handling procedures, were provided in the Supplementary Information.

#### 3.2.2. Effect of the 2-1*H*-Pyrazoline-thiadiazole-Based Derivative 13 on ERK2 and RIPK3 Activation and Necroptotic Signaling

ERK2 phosphorylation and RIPK3 levels were determined using commercial sandwich ELISA kits (PathScan^®^ Phospho-ERK2 [M92.1] (Thr202/Tyr204) [88], Cell Signaling Technology; Danvers; MA, USA; RIPK3 ELISA kit, MyBioSource, San Diego, CA, USA) [89] according to the manufacturers’ instructions. MCF-7 cells were treated with compound **13** (10 µM) for 24 h, lysed, and equal amounts of protein were analyzed. Absorbance was measured at 450 nm, and results were expressed relative to untreated control cells or calculated from standard curves, respectively. Data in details are provided in the Supplementary Information.

#### 3.2.3. Cell Cycle Perturbation and G2/M Phase Arrest of the 2-1*H*-Pyrazoline-thiadiazole-Based Derivative **13**

Cell cycle progression in MCF-7 cells was examined following treatment with derivative **13** using propidium iodide (PI)-based DNA content analysis by flow cytometry [90]. Detailed data were provided in the Supplementary Information.

#### 3.2.4. Induction of Apoptosis by the 2-1*H*-Pyrazoline-thiadiazole-Based Derivative **13**

Apoptosis was evaluated in MCF-7 cells treated with derivative **13** using Annexin V-FITC/PI dual staining and flow cytometric analysis [91]. Data in detail is provided in the Supplementary Information.

#### 3.2.5. Induction of p53 Tumor Suppressor Protein of the 2-1*H*-Pyrazoline-thiadiazole-Based Derivative **13**

The induction of p53 protein was assessed in treated MCF-7 cells using a human p53 sandwich ELISA kit (Sigma-Aldrich, CS0070, St. Louis, MO, USA) according to the manufacturer’s instructions. Briefly, cell lysates were prepared and analyzed by ELISA, and absorbance was measured at 450 nm. p53 levels were calculated from a standard curve and expressed relative to control cells [92,93]. Detailed experimental procedures are provided in the Supplementary Information.

#### 3.2.6. Modulation of Bcl-2 and BAX Expression by the 2-1*H*-Pyrazoline-thiadiazole-Based Derivative **13**

Bcl-2 and Bax protein levels were quantified using human sandwich ELISA kits according to the manufacturers’ instructions. Briefly, cell lysates were prepared and analyzed by ELISA based on antigen-antibody binding and HRP-mediated colorimetric detection. Absorbance was measured at 450 nm, and protein concentrations were calculated from standard curves [94,95]. Detailed assay procedures and performance characteristics are provided in the Supplementary Information.

#### 3.2.7. Antimicrobial Efficiency

##### In Vitro Antibacterial Activity

Antibacterial activity against MRSA USA300 and *A. baumannii* AB5057 was assessed [96,97], and MIC/MBC values were determined by the broth microdilution method following CLSI guidelines [96,98]. Experimental details are provided in the Supplementary Information.

##### Effect of 2-1*H*-Pyrazoline-thiadiazole-Based Analogues **7** and **13** on *MRSA* USA300 and *Acinetobacter baumannii* AB5057 Biofilm Activity

Biofilm inhibition and detachment activities of the tested compounds were evaluated using static crystal violet-based microtiter plate assays as previously described [99,100,101]. Assays were performed at sub-MIC concentrations, and biofilm biomass was quantified spectrophotometrically. Detailed experimental procedures and calculation methods are provided in the Supplementary Information.

##### In Vivo MRSA Skin Infection Model

The in vivo efficacy of compounds **7** and **13** was assessed in a murine MRSA skin infection model [102,103] following ethical approval (MI 4080), with treatment effects evaluated by CFU reduction. Detailed procedures are provided in the Supplementary Information.

##### Assay Against *S. aureus* DNA Gyrase on 2-1*H*-Pyrazoline-thiadiazole-Based Analogues **13** and **7**

The in vitro inhibitory activity of the most active derivatives **7** and **13** was evaluated against *Staphylococcus aureus* DNA gyrase using a DNA gyrase supercoiling assay kit (Inspiralis, Norwich, UK) according to the manufacturer’s instructions [104]. Detailed procedures are provided in the Supplementary Information.

### 3.3. Computational Studies

#### 3.3.1. Molecular Docking Simulation

The chemical structures of derivatives **7** and **13** were prepared and optimized using Molecular Operating Environment (MOE) software 2024.0601. Protein structures of ERK2 [105], RIPK3 [106], and *Staphylococcus aureus* DNA gyrase [107] were retrieved from the Protein Data Bank and prepared for molecular docking using standard protocols. Docking simulations were performed using the MOE docking module. Detailed computational procedures are provided in the Supplementary Information.

#### 3.3.2. Molecular Dynamics Simulations

Molecular dynamics (MD) simulations were carried out using GROMACS-2019 [108] with the CHARMM36 force field [109]. System setup and topology generation were accomplished through the CHARMM-GUI interface [110], followed by standard energy minimization and equilibration procedures before performing 100 ns production runs. A detailed description of the simulation protocol was provided in the Supplementary Information.

#### 3.3.3. Quantum Chemical Calculations and Electronic Analysis

Density Functional Theory (DFT) studies were performed using Gaussian 09 [111]. All molecular geometries were fully optimized at the B3LYP/6-311G++(d,p) level [112,113], and the resulting structures were confirmed as true minima by the absence of imaginary vibrational frequencies. Frontier molecular orbital (FMO) analysis was used to calculate key global reactivity descriptors, including the HOMO-LUMO energy gap (ΔE), electronegativity (χ), chemical hardness (η), softness (σ), electrophilicity index (ω), and ionization potential. Density of states (DOS) calculations were conducted to analyze electronic state distribution and frontier orbital characteristics. Molecular electrostatic potential (ESP) surfaces mapped onto electron density were generated to identify electrophilic and nucleophilic regions and to elucidate charge distribution. In addition, noncovalent interaction (NCI) analysis based on the reduced density gradient (RDG) method was applied to visualize weak interactions and distinguish attractive from repulsive regions.

## 4. Conclusions

The present study reports the design, synthesis, and comprehensive biological evaluation of a series of chalcone-derived, nitrogen-rich heterocyclic compounds as potential anticancer agents. Among the synthesized derivatives, compound **13** consistently emerged as the most potent and mechanistically versatile candidate in vitro, exhibiting strong and selective cytotoxic activity against MCF-7 breast cancer cells through modulation of key signaling pathways. Its biological profile was characterized by ERK2 activation, pronounced p53 up-regulation, G_2_/M cell-cycle arrest, disruption of the BAX/Bcl-2 balance in favor of mitochondrial-mediated apoptosis, and engagement of RIPK3-associated necroptotic signaling, collectively resulting in robust antiproliferative efficacy. In addition to the anticancer evaluation, selected derivatives were further assessed in vivo against methicillin-resistant *Staphylococcus aureus* (MRSA). Notably, compounds **7** and **13** demonstrated the strongest antibacterial activity in the murine infection model, highlighting their broad biological potential. Despite the comparable in vivo efficacy of both candidates, compound **13** was prioritized for advanced investigation owing to its superior multitarget profile, which integrates potent anticancer activity with in vivo anti-MRSA effectiveness. Overall, the integrated in vitro and in vivo findings underscore the therapeutic promise of compound **13** as a multifunctional bioactive scaffold. Moreover, the combined molecular docking, 100 ns molecular dynamics simulations, and DFT calculations provided complementary mechanistic insights into its target interactions and electronic features, thereby supporting its continued optimization as a promising lead for further preclinical development.

## Figures and Tables

**Table 1:** Cytotoxic activity of chalcone **1** and 2-1*H*-pyrazoline-thiadiazole-based analogues **2**–**14** against HeLa, HepG-2, and MCF-7 cancer cell lines and normal WI-38 lung fibroblasts, as determined by the MTT assay and expressed as IC_50_ values.

Comp.	In Vitro Cytotoxicity IC_50_ (µM)			
	WI-38	Hela	HePG-2	MCF-7
Doxorubicin	6.72 ± 0.5	5.57 ± 0.4	4.50 ± 0.2	4.17 ± 0.2
Sorafenib	10.65 ± 0.8	8.04 ± 0.5	9.18 ± 0.6	7.26 ± 0.3
**1**	39.84 ± 2.4	9.74 ± 0.8	8.61 ± 0.6	11.62 ± 0.9
**2**	65.29 ± 3.7	40.91 ± 2.5	34.81 ± 2.2	28.67 ± 1.9
**3**	45.69 ± 2.7	>100	80.53 ± 4.0	75.17 ± 3.8
**4**	55.13 ± 3.2	16.30 ± 1.2	12.71 ± 1.0	6.15 ± 0.4
**5**	>100	73.10 ± 3.9	63.64 ± 3.5	53.37 ± 3.1
**6**	27.75 ± 1.8	61.86 ± 3.5	51.48 ± 2.9	47.38 ± 2.7
**7**	58.60 ± 3.5	22.35 ± 1.6	18.13 ± 1.4	15.56 ± 1.3
**8**	35.04 ± 2.3	91.48 ± 4.6	84.11 ± 4.3	66.23 ± 3.5
**9**	82.27 ± 4.2	44.30 ± 2.7	57.21 ± 3.3	32.77 ± 2.1
**10**	>100	68.07 ± 3.7	76.29 ± 3.8	59.75 ± 3.3
**11**	17.88 ± 1.4	52.36 ± 3.1	42.15 ± 2.6	38.65 ± 2.4
**12**	78.55 ± 3.9	36.26 ± 2.3	21.46 ± 1.6	23.12 ± 1.7
**13**	41.42 ± 2.6	7.81 ± 0.6	5.06 ± 0.3	3.87 ± 0.2
**14**	60.58 ± 3.6	31.97 ± 1.9	29.70 ± 1.8	19.50 ± 1.5

SD stands for standard deviation; each number is the mean of three values; IC_50_ is the compound concentration needed to inhibit growth by 50%.

**Table 2:** In vitro antibacterial activity of 2-1*H*-pyrazoline-thiadiazole-based analogues **7** and **13** against MRSA USA300 and *Acinetobacter baumannii* AB5057 expressed as MIC values.

Compd. No.	MRSA USA300 (Gram +)	*A. baumannii* AB5057 (Gram −)
**7**	10 mg/mL (15.325 μM)	10 mg/mL (15.325 μM)
**13**	10 mg/mL (16.6254 μM)	10 mg/mL (16.6254 μM)

MIC values in μM were calculated from the corresponding mg/mL values based on the molecular weight of each compound. For both compounds, MBC values were identical to the corresponding MIC values.

**Table 3:** Inhibitory activity of the synthesized 2-1*H*-pyrazoline-thiadiazole-based analogues **13** and **7** against *S. aureus* DNA gyrase in comparison with ciprofloxacin.

Compound	IC_50_ (µM)
**13**	17.10 ± 0.17
**7**	21.85 ± 0.19
Ciprofloxacin	19.30 ± 0.72

**Table 4:** DFT-calculated HOMO-LUMO energies and global reactivity descriptors of 2-1*H*-pyrazoline-thiadiazole-based derivatives (**1**–**14**).

Cpd.	LUMO	HOMO	ΔE	A	I	X	η	S or σ	ω	ΔN max	ΔN
**1**	−0.1914	−0.21702	0.02562	0.1914	0.21702	0.20421	0.01281	78.06401	3.255404	7.970726	265.2533
**2**	−0.15438	−0.24586	0.09148	0.15438	0.24586	0.20012	0.04574	21.8627	0.875558	2.187582	74.33188
**3**	−0.15018	−0.23986	0.08968	0.15018	0.23986	0.19502	0.04484	22.30152	0.848189	2.174621	75.88069
**4**	−0.14888	−0.22895	0.08007	0.14888	0.22895	0.188915	0.040035	24.97814	0.891442	2.359373	85.06413
**5**	−0.15025	−0.23013	0.07988	0.15025	0.23013	0.19019	0.03994	25.03756	0.905664	2.380946	85.2505
**6**	−0.14968	−0.19984	0.05016	0.14968	0.19984	0.17476	0.02508	39.87241	1.217746	3.484051	136.0694
**7**	−0.17313	−0.22975	0.05662	0.17313	0.22975	0.20144	0.02831	35.32321	1.433348	3.557753	120.0735
**8**	−0.14998	−0.23019	0.08021	0.14998	0.23019	0.190085	0.040105	24.93455	0.900943	2.369842	84.90107
**9**	−0.15046	−0.23025	0.07979	0.15046	0.23025	0.190355	0.039895	25.0658	0.90826	2.3857	85.34459
**10**	−0.15166	−0.19461	0.04295	0.15166	0.19461	0.173135	0.021475	46.56577	1.395843	4.031083	158.9491
**11**	−0.15033	−0.22997	0.07964	0.15033	0.22997	0.19015	0.03982	25.11301	0.908012	2.387619	85.50791
**12**	−0.15161	−0.18583	0.03422	0.15161	0.18583	0.16872	0.01711	58.44535	1.663731	4.93045	199.6283
**13**	−0.1498	−0.21376	0.06396	0.1498	0.21376	0.18178	0.03198	31.26954	1.03327	2.842089	106.6013
**14**	−0.15018	−0.22858	0.0784	0.15018	0.22858	0.18938	0.0392	25.5102	0.914918	2.415561	86.87015

## Data Availability

The data presented in this study are available in the article and Supplementary Materials.

## Supplementary Materials

The following supporting information can be downloaded at: https://www.mdpi.com/article/10.3390/pharmaceutics18040424/s1, All data generated and analyzed in this study were included in the main manuscript and the Supplementary Information (SI). The SI contained complete spectroscopic characterization data, including IR, ^1^H NMR, ^13^C NMR, and mass spectra with the corresponding molecular structures (Figures S1–S47). Figure S48, IC_50_ dose-response curves for compounds **1**–**14**, detailed experimental procedures, and additional supporting tables (Tables S1–S17). Figure S49. Initial ethical approval certificate.
