# Supplementary material for: A Multi-Target Nitrogen-Fused Azole Drug Platform Derived from a Pyrazoline-Thiadiazole Moiety: In Vivo Antimicrobial Validation and Comprehensive Anticancer Investigation Supported by Computational Studies
Source: Pharmaceutics. 2026 Mar 30;18(4):424. doi: 10.3390/pharmaceutics18040424 (PMC13119528; doi:10.3390/pharmaceutics18040424)
Supplement: Supplementary file 1 [file pharmaceutics-18-00424-s001.zip › pharmaceutics-4188296-supplementary.pdf]

pharmaceutics

Supplementary Materials: A Multi-Target Nitrogen-Fused Azole Drug Platform Derived from a Pyrazoline-Thiadiazole Moiety: In Vivo Antimicrobial Validation and Comprehensive Anticancer Investigation Supported by Computational Studies

Hagar S. El-Hema, Marwa A. Abed, Mohamed A. Hawata, Eman S. Nossier, Najla A. Altwaijry, Asmaa Saleh, Mariam Hassan, Rasha A. Hashem, Modather F. Hussein, Ahmed T. Elhendawy and Adel A.-H. Abdel-Rahman

Content

- Experimental of chemistry, biological evaluation, docking study, and Dynamic stimulations study

- **Table S1.** Percentage cytotoxicity of compounds **1–14** against human HeLa, HepG-2, and MCF-7 cancer cell lines at different concentrations, as determined by the MTT assay.
- **Table S2.** Raw experimental data obtained from the ERK2 assay for the 2-1*H*-pyrazol-thiadiazole-based derivative **13**.
- **Table S3.** Quantitative evaluation of ERK2 levels in MCF-7 cells following treatment with 2-1*H*-pyrazol-thiadiazole derivative **13**, expressed as mean ± SD of three independent experiments.
- **Table S4.** Representative raw experimental output illustrating RIPK3 expression in MCF-7 cells treated with 2-1*H*-pyrazoline-thiadiazole based-derivative **13** compared with untreated control cells.
- **Table S5.** Quantitative evaluation of RIPK3 levels in MCF-7 cells following treatment with 2-1*H*-pyrazoline-thiadiazole based-derivative **13**, expressed as mean ± SD of three independent experiments, showing significant up-regulation compared with untreated control cells.
- **Table S6.** Effect of 2-1*H*-pyrazoline-thiadiazole based-derivative **13** on cell-cycle distribution in MCF-7 cells, expressed as the percentage of cells in the G0/G1, S, and G2/M phases compared with untreated control cells. Data are presented as mean ± SD of three independent experiments.
- **Table S7.** Induction of apoptosis in MCF-7 cells following treatment with 2-1*H*-pyrazoline-thiadiazole based-derivative **13**, as determined by Annexin V/PI staining. Data are expressed as mean ± SD of three independent experiments.
- **Table S8.** Raw experimental data obtained from the p53 assay for 2-1*H*-pyrazoline-thiadiazole-based derivative **13**.
- **Table S9.** Induction of the tumor suppressor protein p53 **in** MCF-7 cells following treatment with 2-1*H*-pyrazoline-thiadiazole based-derivative **13** compared with untreated control cells. Data are presented as mean ± SD of three independent experiments.

- **Table S10.** Representative raw experimental output illustrating Bcl-2 protein expression in MCF-7 cells treated with 2-1*H*-pyrazoline-thiadiazole based-derivative **13** compared with untreated control cells.
- **Table S11.** Quantitative modulation of Bcl-2 protein expression levels **in** MCF-7 **cells** following treatment with 2-1*H*-pyrazoline-thiadiazole based-derivative **13**, expressed as mean ± SD of three independent experiments.
- **Table S12.** Representative raw experimental output illustrating BAX protein expression in MCF-7 cells treated with 2-1*H*-pyrazoline-thiadiazole based-derivative **13** compared with untreated control cells.
- **Table S13.** Effect of 2-1*H*-pyrazoline-thiadiazole based-derivative **13** on BAX protein expression levels in MCF-7 cells, expressed as mean ± SD of three independent experiments, showing a marked increase compared with untreated control cells.
- **Table S14.** Representative gel images and quantitative analysis of DNA gyrase inhibition by 2-1*H*-pyrazoline-thiadiazole based-derivative **13** compared with ciprofloxacin.
- **Table S15.** Representative gel images and quantitative analysis of DNA gyrase inhibition by c2-1*H*-pyrazoline-thiadiazole based-derivative **7** compared with ciprofloxacin.

- **Table S16**. DFT-based electronic structure representations of the investigated compounds **1-14**. For each compound, the table includes five graphical outputs: the highest occupied molecular orbital (HOMO) distribution, the lowest unoccupied molecular orbital **(**LUMO**)** distribution, the electrostatic potential **(**ESP**)** surface, the density of states **(**DOS**)** spectrum, and the electron localization function **(**ELF**)** map.

- **Table S17**. Noncovalent interaction (NCI) and reduced density gradient (RDG) analyses of the investigated compounds **1-14**. For each compound, the table presents the NCI scatter plot illustrating attractive, weak, and repulsive noncovalent interactions, and 3D RDG isosurfaces mapped onto the molecular structure, highlighting regions associated with stabilizing and destabilizing intermolecular interactions.
- **Figure S1–S47**. Copies of IR, ^{1}H NMR, ^{13}C NMR, and mass spectra of Compounds.
- **Figure S48**. Dose-response IC_{50} curves of compounds **1–14**, Doxorubicin (Dox), and Sorafenib (Sor) against Hela, HepG-2, and MCF-7cell lines against normal WI-38 cell

lines. % cell viability was plotted versus log concentration (μM), and IC_{50} values were derived using GraphPad Prism (non-linear regression, n = 3, mean ± SD).

- **Figure S49**. An initial ethical approval certificate issued by the Research Ethics Committee for Experimental and Clinical Studies, Faculty of Pharmacy, Cairo University (REC-FOPCU), approving the in vivo murine experimental study under protocol number MI (4080).

3. Experimental Section

3.1. Chemistry

All reagents and solvents used in the synthesis were purchased from Sigma Aldrich (USA) and other standard commercial suppliers, including El Gomhoureya for Drugs Trade and Medical Supplies (Egypt), El-Nasr Pharmaceutical Chemicals Company (Egypt), Loba Chemie, Alpha Chemika, and Biochem GmbH, and were used without further purification unless otherwise stated. Solvents were dried prior to use when required. Melting points were determined in open-glass capillaries using Gallenkamp or Stuart SMP10 melting point apparatuses and are reported uncorrected.

Infrared (IR) spectra were recorded using KBr pellet/disc techniques on a JASCO FT/IR-6100 spectrometer (Japan) at Al-Azhar University, Cairo, Egypt, and on a Bruker FT-IR spectrometer at the Faculty of Science, Menoufia University, with spectra collected in the range of 4000–400 cm^{-1} and expressed as transmittance (%). Absorption frequencies are reported in cm^{-1}.

^{1}H and ^{13}C NMR spectra were recorded in DMSO-d_6 on JEOL (500 MHz for ^{1}H, 125 MHz for ^{13}C) or Bruker Avance III (400 MHz for ^{1}H, 100 MHz for ^{13}C) spectrometers at Mansoura University and the National Research Centre (NRC), Cairo. Chemical shifts (δ) are expressed in ppm relative to tetramethylsilane (TMS) as an internal standard, coupling constants (J) are given in Hertz (Hz), and signal multiplicities are designated as s, d, t, q, m, br, or dist.

Mass spectrometric analyses were carried out at the Faculty of Science, Al-Azhar University, Egypt, using a Thermo Scientific GC/MS ISQ LT single-quadrupole mass spectrometer equipped with a direct-probe controller inlet, with data processed using Thermo Xcalibur software. Elemental analyses (C, H, and N) were performed using a Perkin–Elmer 2400 elemental analyzer at the Microanalytical Units of Cairo University and Al-Azhar University, and the obtained results were within ±0.4% of the theoretical values.

Reaction progress and compound purity were monitored by thin-layer chromatography (TLC) on silica gel plates (60 GF_{254}, Merck or Macherey–Nagel), with spots visualized under UV light (254 nm) or by exposure to iodine vapor.

Human cancer cell lines HepG2 (hepatocellular carcinoma), HeLa (cervical carcinoma), and MCF-7 (breast carcinoma) were obtained from certified local suppliers in Egypt. All cell lines were

authenticated and confirmed to be mycoplasma-free prior to use at the Faculty of Pharmacy, Mansoura University, Egypt.

All cell-based biological assays, including enzyme inhibition studies, cell cycle analysis, apoptosis assays, and gene expression analysis, were conducted at VACSERA (Holding Company for Biological Products and Vaccines), Cairo, Egypt.

All microbiological experiments, including antibacterial, antibiofilm, and *in vivo* infection studies, were performed at the Department of Microbiology, Faculty of Pharmacy, Cairo University, Egypt.

Previously reported compound **A** was synthesized according to the literature procedure, and its structure was confirmed by comparison of the obtained spectral data with reported values.

3.2. Biological evaluation

3.2.1. Antiproliferative activity

The cell lines were purchased from the American Type Culture collection as follows: cervical carcinoma (HeLa), liver carcinoma (HepG-2), breast carcinoma (MCF-7), and normal lung fibroblast cells (WI-38). Cytotoxic activity screening was performed using MTT assay. Exponentially, cells were placed in 10^4 cells/ well for 24 h, and then add fresh medium which containing different concentrations of the tested sample. Serial two-fold dilutions of the tested sample were added using a multichannel pipette. Moreover, all cells were cultivated at 37 °C, 5% CO_2 and 95% humidity. Also, incubation of control cells occurred at 37 °C. However, after incubation for 24 h, different concentrations of samples (100, 50, 25, and 12.5 μM) were added and continued the incubation for 48 h, then, add the crystal violet solution 1% to each well for 0.5 h to examine viable cells. Rinse the wells using water until no stain. After that, add 30% glacial acetic acid to all wells with shaking plates on Microplate reader (TECAN, Inc.) to measure the absorbance, using a test wavelength of 490 nm. Besides, compare the treated samples with the control cell. The cytotoxicity was estimated by IC_{50} in (μM), the concentration that inhibits 50% of growth of cells.

3.2.2. Effect of 2-1H-pyrazol-thiadiazole based-derivatives 13 on ERK2 and RIPK3 and necroptosis pathway

The effect of 2-1*H*-pyrazol-thiadiazole based-derivatives **13** on ERK2 activation was evaluated using the PathScan® Phospho-p44/42 MAPK (Thr202/Tyr204) Sandwich ELISA Kit (Cell Signaling

Technology, #7300) following the manufacturer's instructions. Briefly, MCF-7 cells were cultured in complete DMEM supplemented with 10% FBS and seeded in 6-well plates at a density of 2×10^5 cells/well. After overnight incubation for cell attachment, the cells were treated with compound 13 (10 μM) for 24 hours. Untreated cells served as the control group. Following treatment, cells were lysed using ice-cold lysis buffer supplied with the kit, and the protein concentration was determined using the BCA protein assay. Equal amounts of total protein (50 μg) were loaded into the wells of the pre-coated ELISA plate. The assay was performed according to the kit protocol to quantify the phosphorylated form of ERK1/2 (Thr202/Tyr204). The absorbance was measured at 450 nm using a microplate reader. Data were normalized to the total protein concentration and expressed as the percentage of ERK1/2 phosphorylation relative to the untreated control. All experiments were conducted in triplicate, and results were represented as mean ± SD.

Human Receptor-Interacting Serine/Threonine-Protein Kinase 3 (RIPK3) concentrations in serum, plasma, tissue homogenates, and cell lysates were measured using a commercial ELISA kit (Catalog No. MBS926196, MyBioSource). The assay employs a sandwich ELISA format, where RIPK3 binds to a microplate-coated capture antibody and is detected by a biotin-conjugated antibody followed by HRP-avidin. Color development with TMB substrate is proportional to the RIPK3 concentration and measured at 450 nm. Samples were prepared according to the manufacturer's instructions, including dilution with Sample Diluent when necessary. All samples and standards were assayed in duplicate, and concentrations were calculated from a standard curve using four-parameter logistic regression. The assay has high sensitivity (<3.9 pg/mL), specificity, and intra- and inter-assay precision (CV <8% and <10%, respectively), allowing accurate quantification of RIPK3 for apoptotic pathway analysis.

3.2.3. Cell cycle analysis

Cell cycle analysis and apoptosis study were carried out using flow cytometry. MCF-7 cells were seeded at 8×10^4 and incubated at 37°C in 5% CO_2 overnight. After treatment with the tested compound **13** for 24 h, cell pellets were collected and centrifuged (300 g, 5 min). For cell cycle analysis cell pellets were fixed with 70% ethanol on ice for 15 min and collected again. The pellets were incubated with propidium iodide (PI) staining solution at room temperature for 1 h and analyzed by a Gallios flow cytometer (Beckman Coulter, Brea, CA, USA).

3.2.4. Apoptosis induction

Apoptosis detection was carried out by FITC AnnexinV/PI commercial kit (Becton Dickenson, Franklin Lakes, NJ, USA) following the manufacturer protocol. The samples were analyzed by fluorescence-activated cell sorting (FACS) with a Gallios flow cytometer (Beckman Coulter, Brea, CA, USA) within 1 h after staining. Data were analyzed using Kaluza v 1.2 (Beckman Coulter).

3.2.5. Induction of p53 tumor suppressor protein

The induction of p53 tumor suppressor protein was evaluated using a Human p53 ELISA Kit (Product No. CS0070, Sigma-Aldrich, St. Louis, MO, USA), a solid-phase sandwich enzyme-linked immunosorbent assay (ELISA) designed to detect and quantify total p53 protein levels independent of phosphorylation status.

Sample Preparation and Cell Lysate Extraction

Cells were collected by centrifugation (for non-adherent cells) or by scraping from culture flasks (for adherent cells), followed by two washes with cold phosphate-buffered saline (PBS). Cell pellets were lysed in Cell Extraction Buffer (10 mM Tris, pH 7.4; 100 mM NaCl; 1 mM EDTA; 1 mM EGTA; 1 mM NaF; 20 mM $Na_4P_2O_7$; 2 mM Na_3VO_4; 1% Triton X-100; 10% glycerol; 0.1% SDS; 0.5% deoxycholate; 1 mM PMSF; protease inhibitor cocktail) on ice for 30 minutes, with vortexing at 10-minute intervals. Lysates were clarified by centrifugation at 13,000 rpm for 10 minutes at 4°C, and the supernatants were collected and aliquoted for analysis. Lysates were stored at –70°C if not analyzed immediately.

ELISA Procedure

Before the assay, all reagents and samples were equilibrated to room temperature (15–30°C). The ELISA was performed using the following steps:

1. Plate Setup: Monoclonal anti-human p53 antibody-coated 96-well plates were used. Standards, controls, and samples (diluted at least 1:10 in Standard Diluent Buffer) were loaded in duplicate wells. Zero wells received 100 µL of Standard Diluent.

2. First Incubation: 100 µL of standards or samples were added to each well, gently mixed, covered, and incubated for 2 hours at room temperature (or overnight at 4°C if desired). Wells were then washed four times with working wash buffer to remove unbound material.

3. Detection Antibody: 100 µL of anti-human p53 detection antibody (rabbit-derived) was added to each well (except chromogen blanks), incubated for 1 hour at room temperature, and washed four times.
4. HRP Conjugation: 100 µL of Anti-Rabbit IgG-HRP working solution was added, incubated for 30 minutes at room temperature, and washed four times.
5. Substrate Reaction: 100 µL of stabilized TMB substrate was added and incubated for 30 minutes at room temperature in the dark. The reaction was stopped by adding 100 µL of Stop Solution, producing a yellow color.
6. Measurement: Absorbance was measured at 450 nm using a microplate reader. The average net optical density (OD) for each sample was calculated by subtracting the OD of chromogen blanks. p53 concentrations were determined from a standard curve generated from serial dilutions of the provided p53 standard (125–8000 pg/mL). Samples exceeding the highest standard were diluted and re-analyzed.

3.2.6. Modulation of Bcl-2 and BAX expression

The human Bcl-2 and Bax ELISA kits were used for quantitative detection of these proteins in cell lysates, serum, or plasma. Bcl-2 is an anti-apoptotic protein associated with cancer progression and therapy resistance, whereas Bax is a pro-apoptotic protein that regulates the balance between cell survival and death. Both assays employ a sandwich ELISA format: the target protein binds to a microplate-coated capture antibody, followed by a biotin-conjugated detection antibody, and then streptavidin-HRP. Color development with TMB substrate was measured at 450 nm. The kits provide all necessary reagents, including antibodies, standards, buffers, wash solutions, and substrates. Samples and standards were prepared according to the manufacturer’s instructions, including proper dilution to fit the standard curve range. Plates were washed between steps to minimize background. The results were calculated using a standard curve, applying any necessary sample dilution factors. Both kits showed high sensitivity (Bcl-2 <0.5 ng/mL; Bax 10.1 pg/mL), good intra-assay precision (CV $\leq 14\%$), acceptable inter-assay reproducibility, reliable spike recovery (71–102%), and stable sample handling under recommended storage conditions. These assays allow accurate quantification of Bcl-2 and Bax for evaluating apoptotic regulation in experimental samples.

3.2.7. Antimicrobial Efficiency

3.2.7.1. In vitro Antibacterial Activity

The antibacterial activity of the tested compounds against highly virulent methicillin-resistant *Staphylococcus aureus* (MRSA USA300) and *Acinetobacter baumannii* AB5057 was investigated. The minimum inhibitory concentration (MIC) and minimum bactericidal concentration (MBC) were determined using the broth microdilution method according to the guidelines of the Clinical and Laboratory Standards Institute.

3.2.7.2. Effect of 2-1H-pyrazoline-thiadiazole-based analogues 7 and 13 on MRSA USA300 and Acinetobacter baumannii AB5057 biofilm activity

This assay investigated the relevant compounds ability to prevent biofilm formation. A static biofilm formation assay was performed in a flat-bottom 96-well ELISA plate as described before Briefly, the bacterial suspension 10^8 CFU/mL (in tryptic soy broth (TSB) for MRSA and lysogeny broth (LB) for *Acinetobacter baumannii*) was loaded in plates (120 uL/well). Different concentrations of the tested compounds below the MIC were added to the bacterial suspension (12 uL/well) and incubated at 37°C for 24h at static conditions. Different sub-MIC concentrations (2.5 – 0.078125 mg/ml) of the tested compounds were tested. DMSO was added to the bacterial suspension in control wells (100 % reference values). After incubation, the absorbance was measured at 600nm. To quantify biofilm formation, wells were washed three times with saline then dried thoroughly to remove all non-adherent cells. Biofilm was stained with crystal violet (0.1% w/v, 150uL/well) for 30 min at room temperature, then rinsed three times with distilled water and then dried thoroughly. The crystal violet in the biofilm was solubilized by adding absolute ethanol (150uL/well) and incubating for 20 min at 4°C and the absorbance was then measured at 550nm. The OD550 of crystal violet solutions was divided by OD600 of the grown cultures for normalization. The experiment was repeated three independent times. The biofilm inhibition % was calculated using the following equation:

$$Biofilm\ inhibition\ \% = \frac{OD\ Control - OD\ Test}{OD\ Control} \times 100$$

Biofilm detachment assay

The biofilm detachment assay was used to evaluate the activity of the tested compounds to detach previously established biofilm. The assay was performed as reported before. The bacterial culture (10^6 CFU/ml) was loaded in a flat-bottom 96-well ELISA plate (120 uL/well) and incubated at 37°C for 24 h without shaking to allow the cells to attach to the surface. Following incubation, the absorbance was measured at 600nm and the contents of each well were then emptied by aspiration. Wells were rinsed three times with sterile distilled water to remove non-adherent cells. Different concentrations (1.25 – 0.0390625 mg/ml) of the tested compounds were prepared in fresh medium then added to the biofilm plate (120 uL/well). Untreated biofilm obtained by using DMSO instead of the tested extracts (100% reference value). The plate was then incubated at 37°C for 24 h. After the treatment period, the wells were stained by crystal violet and measurements were done as described above. The experiment was repeated three independent times. The biofilm detachment % was calculated using the following equation:

$$Biofilm\ detachment\ \% = \frac{OD\ Control - OD\ Test}{OD\ Control} \times 100$$

3.2.7.3. In-vivo MRSA skin infection model

The *in-vivo* efficacy of compounds **7** and **13** was investigated using murine MRSA skin infection model. All the experiments and animal procedures were approved by the Research Ethics Committee of the Faculty of Pharmacy Cairo University (**Approval# MI 4080**) following the "Guide for the Care and Use of Laboratory Animals" published by the Institute of Laboratory Animal Research (Washington, DC, USA). The methicillin-resistant *Staphylococcus aureus* (MRSA) skin infection model was performed as reported before. Thirty BALB/C male mice (7 weeks old) were housed with food and water at ambient temperature. Prior to the experiment, the dorsal backs of the mice were shaved. The lower backs of the mice were injected intradermally with 100 μL

Staphylococcus aureus (MRSA USA300) suspended in sterile saline (9×10^8 CFU). Mice were randomly distributed into five groups (seven mice per group, n=6). Forty-eight hr. post-infection and formation of open wound, the first group was treated topically with compound 7 (50 mg/ml). The second group was treated with compound **13** (50 mg/ml). The third group was treated with vancomycin (25mg/ml). The forth group was treated with 25% DMSO (vehicle). The fifth group was used as the negative control (did not receive any treatment). All groups were topically treated at the site of infection using 100 μL of the assigned treatment once daily for three days. After 24 h from the last treatment, the experiment was terminated and the animals were euthanized; the skin lesion was removed, then homogenized using 0.5 ml saline (homogenizer, DAIHAN-scientific-pacificlab). Samples were diluted 10 folds and tested for aerobic viable count by plating on mannitol salt agar (MSA) followed by incubation at 37 °C. After 24 h incubation, the plates were inspected for colony-forming units (CFU) and the results of the tested groups were analysed and compared.

3.2.7.4 Assay against S. aureus DNA gyrase on 2-1H-pyrazoline-thiadiazole-based analogues 13 and 7

DNA Gyrase Inhibition Assay

The DNA gyrase inhibitory activity of the tested compounds was evaluated using a *Staphylococcus aureus* DNA gyrase supercoiling assay kit (Inspiralis, Norwich, UK) according to the manufacturer's instructions.

Enzyme and Substrate

Purified *Staphylococcus aureus* DNA gyrase ($GyrA_2GyrB_2$ holoenzyme) and relaxed plasmid DNA substrate supplied with the kit were used. Enzyme aliquots were stored at −70 °C, while plasmid DNA was stored at 4 °C in accordance with the manufacturer's recommendations.

Reaction Conditions

Assays were performed in a final reaction volume of 20 μL containing the recommended gyrase assay buffer, relaxed plasmid DNA substrate, sterile distilled water, and the tested compounds at the indicated concentrations. DNA gyrase was added last on ice to initiate the reactions.

Incubation and Termination

Reaction mixtures were incubated at 37 °C for 30–60 min and terminated by the addition of stop buffer containing SDS, followed by proteinase K digestion to remove enzyme–DNA complexes.

Electrophoretic Analysis

Reaction products were resolved by electrophoresis on 1% agarose gels prepared in TAE buffer. Gels were stained with ethidium bromide after electrophoresis, and DNA bands corresponding to relaxed, supercoiled, and linear forms were visualized using a UV transilluminator.

Controls

Reactions lacking DNA gyrase were included as negative controls. Ciprofloxacin was used as a positive control DNA gyrase inhibitor, while solvent controls containing equivalent concentrations of DMSO were also included.

Data Interpretation

DNA gyrase inhibition was assessed based on the reduction in supercoiled DNA formation and/or the appearance of linear DNA bands relative to control reactions.

3.3. In silico studies

3.3.1. Molecular docking simulation

The 2D structures of 2-1*H*-pyrazol-thiadiazole derivative **7** and **13** were drawn through Chem. Draw. The protonated 3D was employed using standard bond lengths and angles, using Molecular Operating Environment (MOE-Dock) software version 2024.0601. Then, the geometry optimization and energy minimization were applied to get the Conf Search module in MOE, followed by saving of the moe file for upcoming docking process. The co-crystallized structures of ERK2, and RIPK3 with

their ligands 7-(1-propyl-1*H*-pyrazol-4-yl)-2-(pyridin-4-yl)-5*H*-pyrrolo[2,3-*b*]pyrazine (35X) and GSK'843 were downloaded (PDB codes: 4QP9 and 7XM3, respectively) from protein data bank. All minimizations were performed using MOE until an RMSD gradient of 0.05 kcal·mol^{-1}Å^{-1} with MMFF94x force field and the partial charges were automatically calculated. Preparation of the enzymes' structures were done for molecular docking using Protonate 3D protocol with the default options in MOE. London dG scoring function and Triangle Matcher placement method were used in the docking protocol. Initially, the validation of the docking processes were established by docking the native ligands, followed by docking the derivative **13** within the ATP-binding sites after eliminating the co-crystallized ligands.

For confirmation of the antimicrobial activity, the crystal structure of *Staphylococcus aureus* DNA gyrase was obtained from the RCSB database (PDB code: 2XCT), following the previously mentioned method to complete the docking processes of the promising compounds **7** and **13**.

3.3.2. Molecular dynamics simulations

The molecular dynamics (MD) simulations were performed following a standard multistep protocol. Initially, the crystal structures of ERK2, RIPK3, and DNA gyrase were retrieved from the Protein Data Bank and structurally refined prior to simulation. System preparation was carried out using CHARMM-GUI, where protein-ligand complex topology files were generated employing the CHARMM36 all-atom force field. Each complex was solvated in a cubic simulation box using TIP3P water molecules, and appropriate numbers of Cl^- counterions were added to ensure overall charge neutralization.

Energy minimization was performed for 5000 steps using the steepest-descent algorithm to remove unfavorable steric contacts. This was followed by a two-step equilibration protocol consisting of NVT equilibration for 125 ps at 310 K, followed by NPT equilibration under constant pressure and temperature conditions. Subsequently, production MD simulations were carried out for 100 ns exclusively on the protein–ligand complexes of ERK2, RIPK3, and DNA gyrase. A 2 fs integration timestep was applied, with all hydrogen-containing bonds constrained using the LINCS algorithm, and long-range electrostatic interactions treated using the Particle Mesh Ewald (PME) method.

Trajectory analyses were performed using GROMACS analysis tools, including calculations of root mean square deviation (RMSD), radius of gyration (Rg), solvent-accessible surface area

(SASA), and root mean square fluctuation (RMSF), providing a comprehensive assessment of the structural stability and dynamic behavior of the simulated protein-ligand complexes.

Tables

Table S1. Percentage cytotoxicity of compounds **1–14** against human HeLa, HepG-2, and MCF-7 cancer cell lines at different concentrations, as determined by the MTT assay.

Conc.(µM)	WI-38	Hela	HePG2	MCF-7
		DOX		
1.56	7.8	7.3	6.3	6.2
3.125	14.5	12.1	11.2	10.9
6.25	21.2	18.9	14.1	14.3
12.5	34.0	30.8	28.3	26.9
25	50.3	51.7	45.8	41.5
50	64.9	62.4	57.6	58.4
100	87.4	74.0	71.2	69.1
		SOR		
1.56	13.1	7.9	9.2	8.1
3.125	17.8	18.1	16.6	15.2
6.25	29.0	23.9	25.7	23.0
12.5	40.3	34.6	37.2	34.8
25	63.7	56.5	61.3	51.6
50	78.6	72.1	72.9	68.9
100	96.5	87.7	95.4	87.5
		1		

1.56	33.6	7.9	8.7	12.9
3.125	45.1	19.3	16.8	21.8
6.25	56.0	24.4	28.4	29.7
12.5	69.4	38.5	34.5	40.3
25	91.3	57.6	60.4	67.2
50	100	84.4	70.7	81.4
100	100	97.4	89.2	98.1
2				
1.56	42.9	32.5	30.4	26.8
3.125	55.8	45.6	42.5	35.7
6.25	67.4	58.9	53.7	50.6
12.5	80.5	70.4	66.7	67.1
25	94.7	90.3	87.9	82.0
50	100	100	99.6	95.3
100	100	100	100	100
3				
1.56	100	100	100	100
3.125	50	50	50	50
6.25	25	25	25	25
12.5	12.5	12.5	12.5	12.5
25	6.25	6.25	6.25	6.25
50	3.125	3.125	3.125	3.125
100	1.56	1.56	1.56	1.56
4				
1.56	38.7	18.3	13.7	7.7
3.125	51.4	26.5	22.8	14.5

6.25	65.2	39.1	31.2	21.3
12.5	79.5	51.4	46.9	34.9
25	97.8	69.7	62.3	45.7
50	100	86.3	87.2	60.5
100	100	100	99.3	87.4
		5		
1.56	53.1	45.6	39.4	37.7
3.125	77.5	57.2	55.3	53.2
6.25	84.7	71.4	71.5	62.5
12.5	99.3	84.7	93.9	75.1
25	100	98.1	100	96.8
50	100	100	100	100
100	100	100	100	100
		6		
1.56	25.3	38.2	38.7	36.2
3.125	36.7	55.4	49.3	49.3
6.25	50.2	70.2	61.5	60.7
12.5	64.8	92.4	78.8	74.4
25	80.4	100	96.1	87.2
50	96.3	100	100	100
100	100	100	100	100
		7		
1.56	40.7	23.0	21.4	20.0
3.125	54.2	35.3	28.9	29.4
6.25	64.3	46.7	41.5	37.2
12.5	77.0	57.4	50.6	46.5

25	92.6	71.8	72.1	67.9
50	100	90.5	91.7	86.6
100	100	100	100	100
8				
1.56	27.9	49.4	48.3	40.7
3.125	41.8	65.6	60.2	58.1
6.25	56.0	83.5	77.2	70.6
12.5	71.7	97.3	85.6	81.9
25	83.6	100	98.5	98.8
50	100	100	100	100
100	100	100	100	100
9				
1.56	46.1	34.7	36.1	30.4
3.125	61.7	45.0	55.4	41.6
6.25	79.4	60.2	68.7	53.2
12.5	86.2	76.6	78.3	65.5
25	98.1	94.5	93.6	79.8
50	100	100	100	98.3
100	100	100	100	100
10				
1.56	55.4	41.5	47.1	43.6
3.125	69.7	59.3	55.9	52.4
6.25	82.3	69.2	76.7	62.3
12.5	95.2	84.8	89.6	79.2
25	100	99.4	100	99.5
50	100	100	100	100

100	100	100	100	100
11				
1.56	20.3	36.8	33.8	31.6
3.125	27.8	51.9	49.5	41.2
6.25	39.1	62.7	56.6	61.5
12.5	52.9	78.6	69.9	70.4
25	73.6	96.5	83.8	86.3
50	91.5	100	98.7	99.7
100	100	100	100	100
12				
1.56	47.7	31.3	25.1	23.7
3.125	58.3	41.8	31.9	37.9
6.25	72.4	52.6	45.6	49.3
12.5	84.5	74.0	57.2	53.8
25	98.2	88.2	70.4	70.6
50	100	99.7	91.5	92.4
100	100	100	100	100
13				
1.56	100	100	100	100
3.125	50	50	50	50
6.25	25	25	25	25
12.5	12.5	12.5	12.5	12.5
25	6.25	6.25	6.25	6.25
50	3.125	3.125	3.125	3.125
100	1.56	1.56	1.56	1.56
14				

**1.56**	**38.6**	**29.7**	**24.8**	**20.5**
**3.125**	**54.4**	**41.0**	**39.2**	**31.8**
**6.25**	**69.1**	**53.1**	**51.4**	**42.6**
**12.5**	**88.3**	**65.4**	**68.5**	**54.2**
**25**	**100**	**78.6**	**79.3**	**71.7**
**50**	**100**	**98.3**	**97.6**	**92.3**
**100**	**100**	**100**	**100**	**100**

Table S2. Raw experimental data obtained from the ERK2 assay for the 2-1*H*-pyrazoline-thiadiazole-based derivative **13**.

	1	2	3	4	5	6	7	8	9	10	11	12
A	ST1	**s13**	--	--	--	--	--	--	--	--	--	--
B	ST2	**s13**	--	--	--	--	--	--	--	--	--	--
C	ST3	**cont**	--	--	--	--	--	--	--	--	--	--
D	ST4	**cont**	--	--	--	--	--	--	--	--	--	--
E	ST5	---	--	--	--	--	--	--	--	--	--	--
F	ST6	---	--	--	--	--	--	--	--	--	--	--
G	ST7	---	--	--	--	--	--	--	--	--	--	--
H	B	---	--	--	--	--	--	--	--	--	--	--

**ST.**	CONC.ug/ml
St.1	**10**
St.2	**5**
St.3	**2.5**
St.4	**1.25**
St.5	**0.63**
St.6	**0.313**
St.7	**0.157**

Detailed Results:

Plate map

Sample ODs

	1	2	3	4	5	6	7	8	9	10	11	12
A	2.773	1.053	**0**	**0**	**0**	**0**	**0**	**0**	**0**	**0**	**0**	**0**
B	1.895	0.982	**0**	**0**	**0**	**0**	**0**	**0**	**0**	**0**	**0**	**0**
C	1.342	0.629	**0**	**0**	**0**	**0**	**0**	**0**	**0**	**0**	**0**	**0**
D	0.808	0.644	**0**	**0**	**0**	**0**	**0**	**0**	**0**	**0**	**0**	**0**
E	0.514	0	**0**	**0**	**0**	**0**	**0**	**0**	**0**	**0**	**0**	**0**
F	0.346	0	**0**	**0**	**0**	**0**	**0**	**0**	**0**	**0**	**0**	**0**
G	0.284	0	**0**	**0**	**0**	**0**	**0**	**0**	**0**	**0**	**0**	**0**
H	0.027	0	**0**	**0**	**0**	**0**	**0**	**0**	**0**	**0**	**0**	**0**

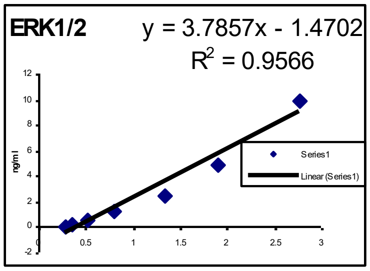

**STANDARDS**	OD	ng/ml	**Slope**	**Intercept**	**R2**
St.1	2.773	10	0.244666317	0.476631037	0.9566
St.2	1.895	5			
St.3	1.342	2.5			

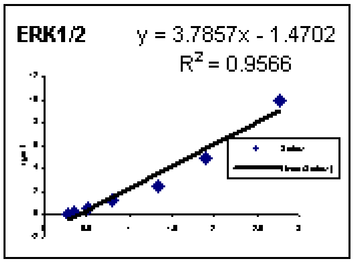

St.4	0.808	1.25
St.5	0.514	0.63
St.6	0.346	0.313
St.7	0.284	0.157
	0.027	

Plate map

	**1**	**2**
A	St.1	**s13**
B	St.2	**s13**
C	St.3	**cont**
D	St.4	**cont**
E	St.5	--
F	St.6	--
G	St.7	--
H	B	--

Samples ODs

	**1**	**2**
A	2.773	1.053
B	1.895	0.982
C	1.342	0.629
D	0.808	0.644
E	0.514	0
F	0.346	0
G	0.284	0
H	0.027	0

results

	**1**	**2**
A	9.470	2.440

B	5.882	2.150
C	3.621	0.707
D	1.439	0.769
E	0.237	-1.864
F	-0.449	-1.864
G	-0.703	-1.864
H	-1.753	-1.864

Table S3. Quantitative evaluation of ERK2 levels in MCF-7 cells following treatment with 2-1*H*-pyrazoline-thiadiazole derivative **13**, expressed as mean ± SD of three independent experiments.

**Sample code**	**ERK 2**	**Fld.**
**13/MCF7**	2.295±0.09	3.17
**cont.MCF7**	0.738±0.03	1.00

Table S4. Representative experimental output showing activation of RIPK3 and necroptosis pathway in MCF-7 cells treated with 2-1*H*-pyrazoline-thiadiazole based-derivative **13**. The elevated RIPK3 signal intensity confirms induction of regulated necrotic cell death.

Detailed Results:

**STANDARDS**	CONC.pg/ml
St.1	1000
St.2	500
St.3	250
St.4	125
St.5	62.5
St.6	31.3
St.7	15.6

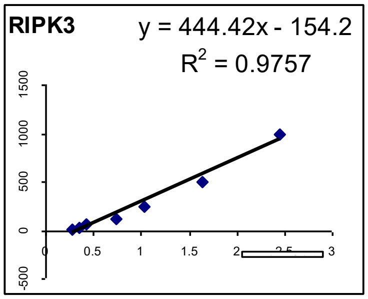

Plate map

	1	2	3	4	5	6	7	8	9	10	11	12
A	St.1	**s13**	--	--	--	--	--	--	--	--	--	--
B	St.2	**s13**	--	--	--	--	--	--	--	--	--	--
C	St.3	**cont.**	--	--	--	--	--	--	--	--	--	--
D	St.4	**cont.**	--	--	--	--	--	--	--	--	--	--
E	St.5	--	--	--	--	--	--	--	--	--	--	--
F	St.6	--	--	--	--	--	--	--	--	--	--	--
G	St.7	--	--	--	--	--	--	--	--	--	--	--
H	B	--	--	--	--	--	--	--	--	--	--	--

Samples OD results

	1	2	3	4	5	6	7	8	9	10	11	12
A	2.439	1.077	0	0	0	0	0	0	0	0	0	0
B	1.633	1.159	0	0	0	0	0	0	0	0	0	0
C	1.028	0.428	0	0	0	0	0	0	0	0	0	0
D	0.736	0.407	0	0	0	0	0	0	0	0	0	0
E	0.424	0	0	0	0	0	0	0	0	0	0	0

F	0.351	0	0	0	0	0	0	0	0	0	0	0
G	0.283	0	0	0	0	0	0	0	0	0	0	0
H	0.046	0	0	0	0	0	0	0	0	0	0	0

RIPK3

STANDARDS	OD	ng/ml
St.1	2.439	1000
St.2	1.633	500
St.3	1.028	250
St.4	0.736	125
St.5	0.424	62.5
St.6	0.351	31.3
St.7	0.283	15.6
	0.046	

Slope	Intercept	R2
0.002195457	0.362476317	0.9757

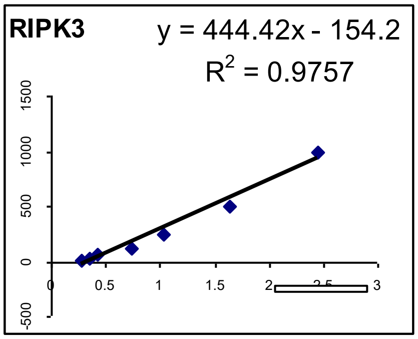

Plate map

	1	2
A	st 1	**s13**
B	st 2	**s13**
C	st 3	**cont.**
D	st 4	**cont.**
E	st 5	--
F	st 6	--
G	st 7	--
H	B	--

Samples ODs

	1	2
A	2.439	1.077

B	1.633	1.159
C	1.028	0.428
D	0.736	0.407
E	0.424	0
F	0.351	0
G	0.283	0
H	0.046	0

results

	**1**	**2**
A	949.839	329.467
B	582.718	366.817
C	307.149	33.857
D	174.147	24.292
E	32.035	-161.091
F	-1.215	-161.091
G	-32.188	-161.091
H	-140.139	-161.091

Table S5. Quantitative evaluation of RIPK3 levels in MCF-7 cells following treatment with 2-1*H*-pyrazoline-thiadiazole based-derivative **13**, expressed as mean ± SD of three independent experiments, showing significant up-regulation compared with untreated control cells.

**Sample code**	**RIPK3 (pg/mL)**	**fld**
**s13/MCF7**	348.14±12.53	11.97
**cont.MCF7**	29.07±1.41	1.00

Table S6. Effect of 2-1*H*-pyrazoline-thiadiazole based-derivative **13** on cell-cycle distribution in MCF-7 cells, expressed as the percentage of cells in the G0/G1, S, and G2/M phases compared with untreated control cells.

Sample code	DNA content			
	%G0-G1	%S	%G2/M	Comment
**13/MCF7**	37.51	22.33	40.16	Cell cycle arrest@G2/M
**cont.MCF7**	59.28	26.57	14.15	---

Table S7. Induction of apoptosis in MCF-7 cells following treatment with 2-1*H*-pyrazoline-thiadiazole based-derivative **13**, as determined by Annexin V/PI staining.

Code	Apoptosis			Necrosis
	Total	Early	Late	
**s13/MCF7**	32.89	11.02	18.36	3.51
**cont.MCF7**	2.84	0.49	0.18	2.17

Table S8. Detailed results of p53 enzyme assay of 2-1*H*-pyrazoline-thiadiazole based-derivative **13**.

STANDARDS	CONC.pg/ml
St.1	4000
St.2	2000
St.3	1000
St.4	500
St.5	250
St.6	125
St.7	62.5

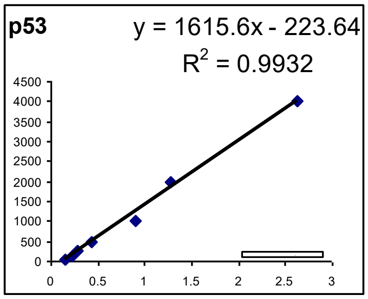

Plate map

	1	2	3	4	5	6	7	8	9	10	11	12
A	St.1	**s13**	**--**	--	--	--	--	--	--	--	--	--
B	St.2	**s13**	**--**	--	--	--	--	--	--	--	--	--
C	St.3	**cont.**	**--**	--	--	--	--	--	--	--	--	--
D	St.4	**cont.**	**--**	--	--	--	--	--	--	--	--	--
E	St.5	**--**	**--**	--	--	--	--	--	--	--	--	--
F	St.6	**--**	**--**	--	--	--	--	--	--	--	--	--
G	St.7	**--**	**--**	--	--	--	--	--	--	--	--	--
H	B	**--**	**--**	--	--	--	--	--	--	--	--	--

Samples OD results

	1	2	3	4	5	6	7	8	9	10	11	12
A	2.482	0.436	0	0	0	0	0	0	0	0	0	0
B	1.237	0.444	0	0	0	0	0	0	0	0	0	0
C	0.741	0.261	0	0	0	0	0	0	0	0	0	0
D	0.266	0.242	0	0	0	0	0	0	0	0	0	0
E	0.184	0	0	0	0	0	0	0	0	0	0	0
F	0.141	0	0	0	0	0	0	0	0	0	0	0
G	0.088	0	0	0	0	0	0	0	0	0	0	0
H	0.017	0	0	0	0	0	0	0	0	0	0	0

STANDARDS	OD	ng/ml
St.1	2.618	4000
St.2	1.277	2000
St.3	0.903	1000
St.4	0.432	500
St.5	0.272	250
St.6	0.221	125
St.7	0.159	62.5
	0.002	

Slope	Intercept	R2
0.000614752	0.143201149	0.9966

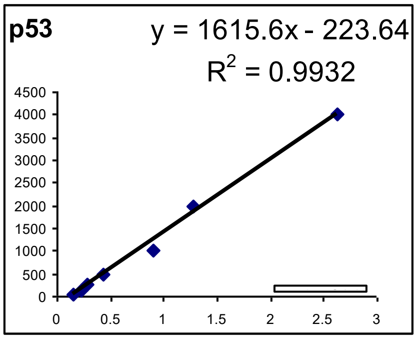

Plate map

	1	2
A	st 1	**s13**
B	st 2	**s13**
C	st 3	**cont.**
D	st 4	**cont.**
E	st 5	--
F	st 6	--
G	st 7	--
H	B	--

Samples ODs

	1	2
A	2.618	0.732
B	1.277	0.694
C	0.903	0.289
D	0.432	0.318
E	0.272	0
F	0.221	0
G	0.159	0

H	0.002	0

results

	1	2
A	4026.480	958.575
B	1845.112	896.762
C	1236.736	237.959
D	470.573	285.133
E	210.306	-232.149
F	127.345	-232.149
G	26.492	-232.149
H	-228.896	-232.149

Table S9. Induction of the tumor suppressor protein p53 in MCF-7 cells following treatment with 2-1*H*-pyrazoline-thiadiazole based-derivative **13** compared with untreated control cells. Data are presented as mean ± SD of three independent experiments.

**Sample code**	**p53 (pg mL^{-1})**	**Fld.**
**13/MCF7**	927.66±32.06	3.54
**cont.MCF7**	261.54±9.87	1.00

Table S10. Representative raw experimental output illustrating Bcl-2 protein expression in MCF-7 cells treated with 2-1*H*-pyrazoline-thiadiazole based-derivative **13** compared with untreated control cells.

STANDARDS	Conc. ng/ml
St.1	32.0
St.2	16.0
St.3	8.0
St.4	4.0
St.5	2.0
St.6	1.0

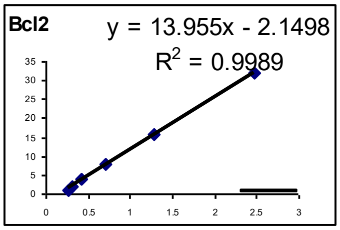

Plate map

	1	2	3	4	5	6	7	8	9	10	11	12
A	St.1	**s13**	--	--	--	--	--	--	--	--	--	--
B	St.2	**s13**	--	--	--	--	--	--	--	--	--	--
C	St.3	**C**	--	--	--	--	--	--	--	--	--	--
D	St.4	**C**	--	--	--	--	--	--	--	--	--	--
E	St.5	--	--	--	--	--	--	--	--	--	--	--
F	St.6	--	--	--	--	--	--	--	--	--	--	--
G	B	--	--	--	--	--	--	--	--	--	--	--
H	B	--	--	--	--	--	--	--	--	--	--	--

Samples OD results

	1	2	3	4	5	6	7	8	9	10	11	12
A	2.531	0.443	0	0	0	0	0	0	0	0	0	0
B	1.369	0.426	0	0	0	0	0	0	0	0	0	0
C	0.774	1.289	0	0	0	0	0	0	0	0	0	0
D	0.348	1.372	0	0	0	0	0	0	0	0	0	0
E	0.222	0	0	0	0	0	0	0	0	0	0	0
F	0.125	0	0	0	0	0	0	0	0	0	0	0
G	0.018	0	0	0	0	0	0	0	0	0	0	0
H	0.029	0	0	0	0	0	0	0	0	0	0	0

STANDARDS	OD	ng/ml	Slope	Intercept	R2
St.1	2.463	32	0.071585643	0.154850746	0.9989
St.2	1.282	16			
St.3	0.697	8			
St.4	0.417	4			
St.5	0.318	2			
St.6	0.262	1			
St.7	0.022				
	0.037				

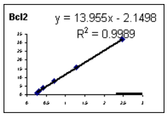

Plate map

	1	2
A	St.1	**s13**
B	St.2	**s13**
C	St.3	**C**
D	St.4	**C**
E	St.5	--
F	St.6	--
G	B	--
H	B	--

Samples ODs

	1	2
A	2.463	0.443
B	1.282	0.426
C	0.697	1.289
D	0.417	1.372
E	0.318	0
F	0.262	0
G	0.022	0

H	0.037	0

results

	1	2
A	32.246	4.028
B	15.748	3.790
C	7.576	15.846
D	3.664	17.005
E	2.281	-2.161
F	1.499	-2.161
G	-1.853	-2.161
H	-1.644	-2.161

Table S11. Quantitative modulation of Bcl-2 protein expression levels in MCF-7 cells following treatment with 2-1*H*-pyrazoline-thiadiazole based-derivative **13**, expressed as mean ± SD of three independent experiments.

**Compound code**	**Bcl-2 (ng/ml)**	**FLD**
**s13/MCF7**	3.91±0.13	0.24
**cont.MCF7**	16.43±0.56	1.00

Table S12. Representative raw experimental output illustrating BAX protein expression in MCF-7 cells treated with 2-1*H*-pyrazoline-thiadiazole based-derivative **13** compared with untreated control cells.

STANDARDS	Conc.<br>Pg/ml
St.1	2000
St.2	1000
St.3	500
St.4	250
St.5	125
St.6	62.5

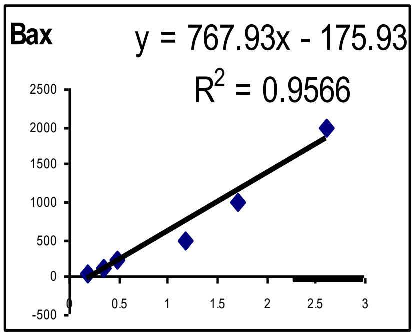

Plate map

		mcf7										
	**1**	**2**	3	4	5	6	7	8	9	10	11	12
A	St.1	**s13**	--	--	--	--	--	--	--	--	--	--
B	St.2	**s13**	--	--	--	--	--	--	--	--	--	--
C	St.3	**cont**	--	--	--	--	--	--	--	--	--	--
D	St.4	**cont**	--	--	--	--	--	--	--	--	--	--
E	St.5	**--**	--	--	--	--	--	--	--	--	--	--
F	St.6	**--**	--	--	--	--	--	--	--	--	--	--
G	B	**--**	--	--	--	--	--	--	--	--	--	--
H	B	**--**	--	--	--	--	--	--	--	--	--	--

Samples OD results

		HCT										
	**1**	**2**	3	4	5	6	7	8	9	10	11	12
A	2.606	1.159	0	0	0	0	0	0	0	0	0	0
B	1.713	1.216	0	0	0	0	0	0	0	0	0	0
C	1.185	0.361	0	0	0	0	0	0	0	0	0	0
D	0.476	0.335	0	0	0	0	0	0	0	0	0	0
E	0.335	0	0	0	0	0	0	0	0	0	0	0
F	0.187	0	0	0	0	0	0	0	0	0	0	0

G	0.029	0	0	0	0	0	0	0	0	0	0	0
H	0.036	0	0	0	0	0	0	0	0	0	0	0

STANDARDS	OD	ng/ml
St.1	2.606	2000
St.2	1.713	1000
St.3	1.185	500
St.4	0.476	250
St.5	0.335	125
St.6	0.187	62.5
St.7	0.029	
	0.036	

Slope	Intercept	R2
0.001245657	0.26620398	0.9566

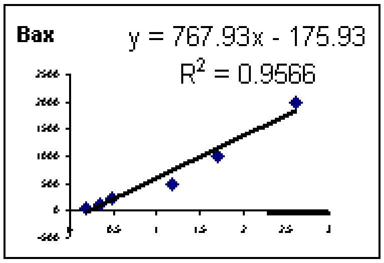

Plate map

	1	2
A	St.1	**s13**
B	St.2	**s13**
C	St.3	**cont**
D	St.4	**cont**
E	St.5	--
F	St.6	--
G	B	--
H	B	--

Samples ODs

	1	2
A	2.606	1.159
B	1.713	1.216
C	1.185	0.361

D	0.476	0.335
E	0.335	0
F	0.187	0
G	0.029	0
H	0.036	0

results

	1	2
A	1888	726
B	1171	772
C	747	85
D	178	65
E	65	-204
F	-54	-204
G	-181	-204
H	-176	-204

Table S13. Effect of 2-1*H*-pyrazoline-thiadiazole based-derivative **13** on BAX protein expression levels in MCF-7 cells, expressed as mean ± SD of three independent experiments, showing a marked increase compared with untreated control cells.

Compound code	BAX pg/mL	FLD
**s13/MCF7**	748.88±29.10	9.99
**cont.MCF7**	74.94±2.91	1.00

Table S14. Representative gel images and quantitative analysis of DNA gyrase inhibition by 2-1*H*-pyrazoline-thiadiazole based-derivative **13** compared with ciprofloxacin.

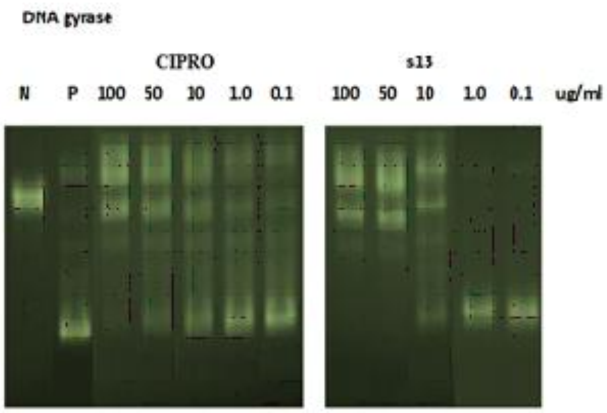

DNA Gyrase

code	IC50	conc	log	%inh
			[redacted]	
code	IC50	conc	log	%inh
s13		100	2	92.2
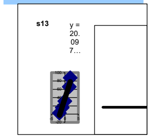		50	1.7	79.9
		1	0	51.5
		0.1	-1	22.4
		0.01	-2	7.82
EC				0
		[redacted]		[redacted]
code	IC50	conc	log	%inh
ciprofloxacin		100	2	91.4
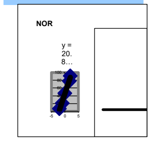		50	1.7	77.9
		1	0	46.2
		0.1	-1	20.9
		0.01	-2	6.97
EC				0
		[redacted]		[redacted]

Table S15. Representative gel images and quantitative analysis of DNA gyrase inhibition by 2-1*H*-pyrazoline-thiadiazole based-derivative **7** compared with ciprofloxacin.

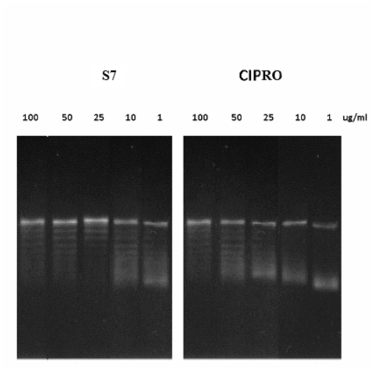

DNA Gyrase

code	IC50	conc	log	%inh
s7		100	2	92.2
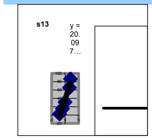		50	1.7	79.9
		1	0	51.5
		0.1	-1	22.4
		0.01	-2	7.82
EC				0

code	IC50	conc	log	%inh
ciprofloxacin		100	2	91.4
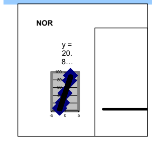		50	1.7	77.9
		1	0	46.2
		0.1	-1	20.9
		0.01	-2	6.97
EC				0

[redacted] [redacted]

Table S16. DFT-based electronic structure representations of the investigated compounds **1-14**. For each compound, the table includes five graphical outputs: the highest occupied molecular orbital (HOMO) distribution, the lowest unoccupied molecular orbital **(**LUMO**)** distribution, the electrostatic potential **(**ESP**)** surface, the density of states **(**DOS**)** spectrum, and the electron localization function **(**ELF**)** map.

File name	LUMO	HOMO	ESP/ELF
1	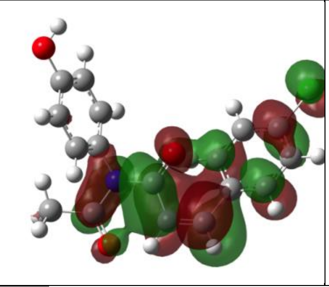	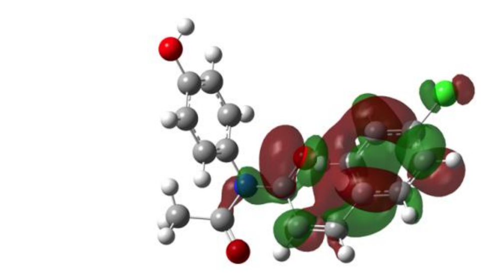	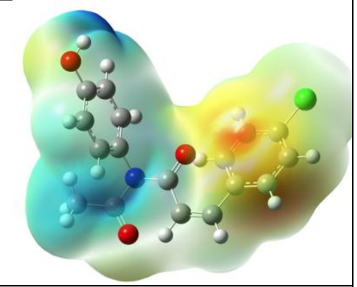
	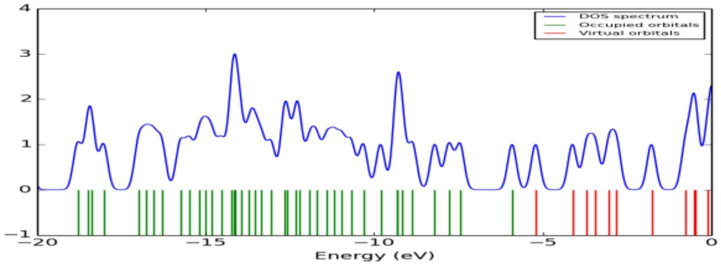		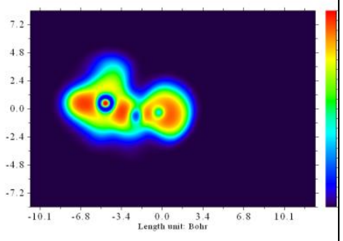
2	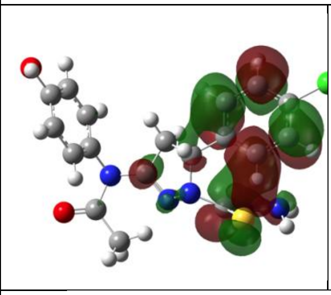	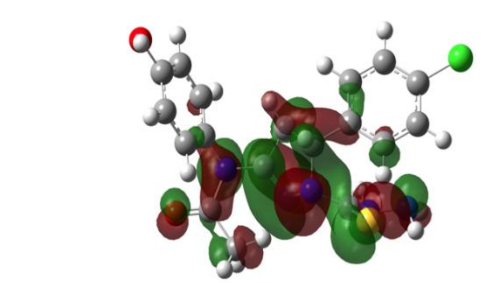	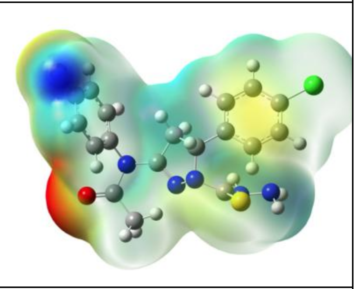
	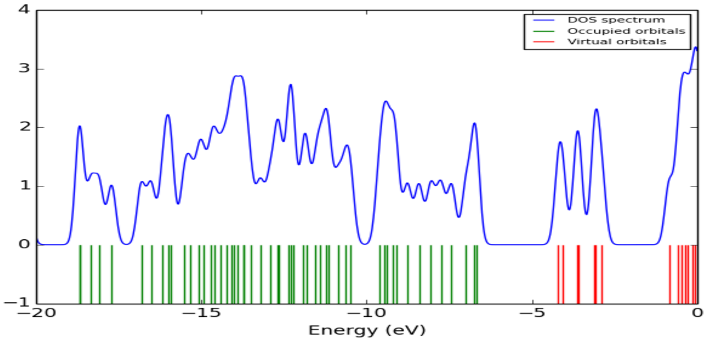		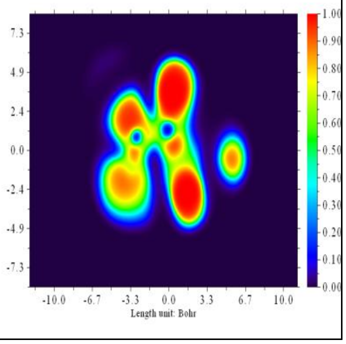

<table>
<tr><td rowspan="2">3</td><td>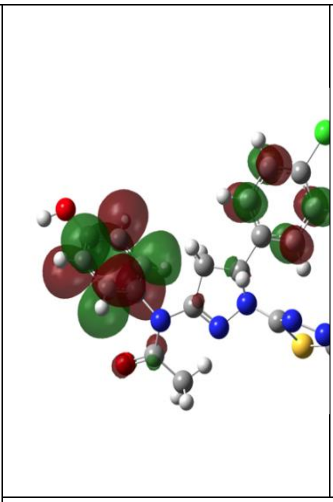</td><td>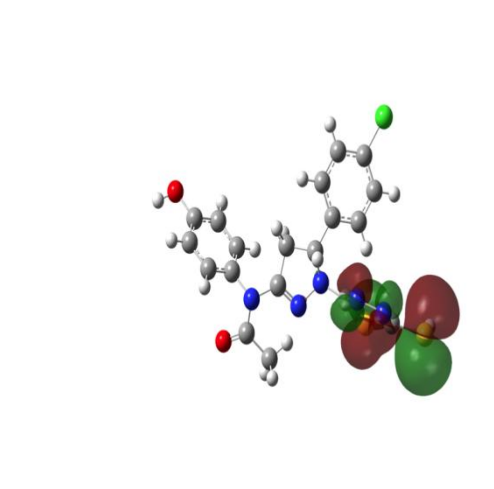</td><td>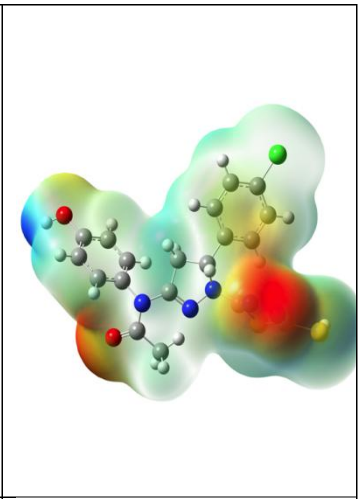</td></tr>
<tr><td colspan="2">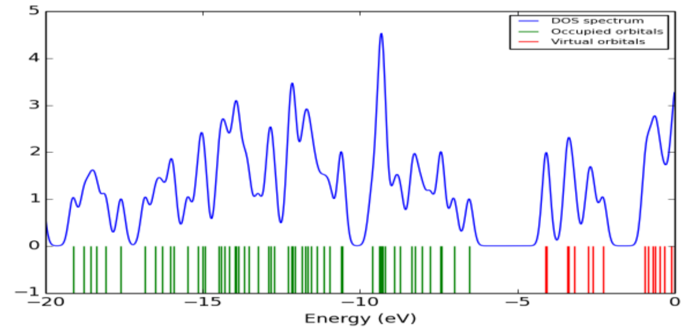
</td><td>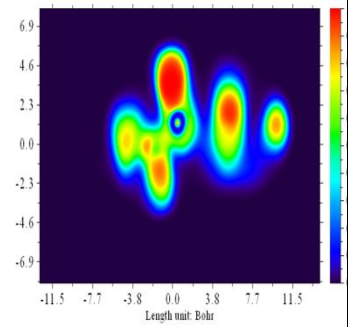
</td></tr>
<tr><td rowspan="2">4</td><td>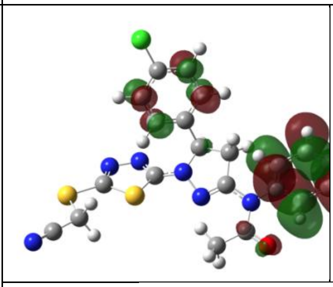</td><td>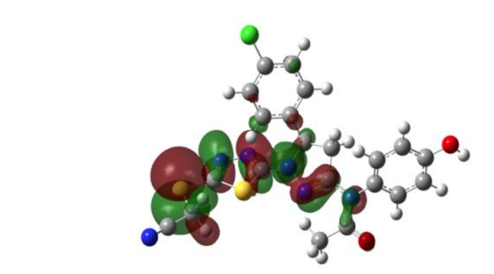</td><td>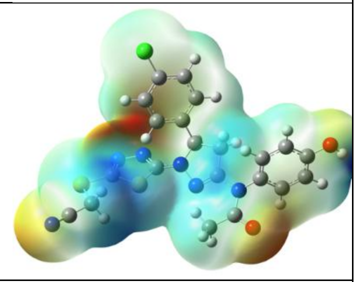</td></tr>
<tr><td colspan="2">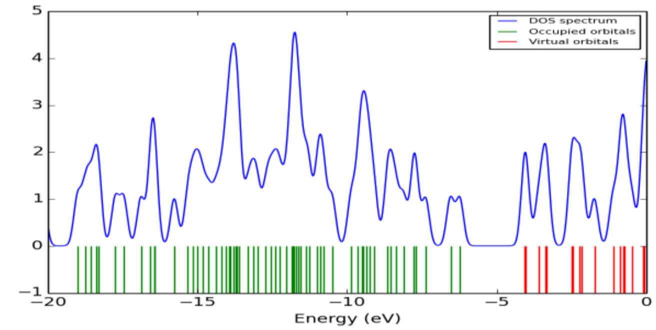
</td><td>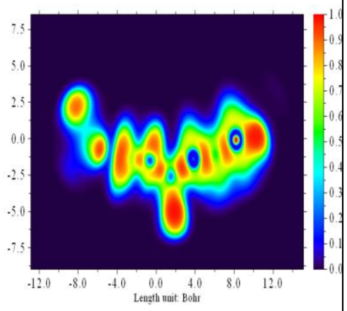
</td></tr>
<tr><td>5</td><td>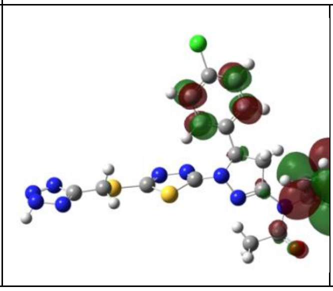</td><td>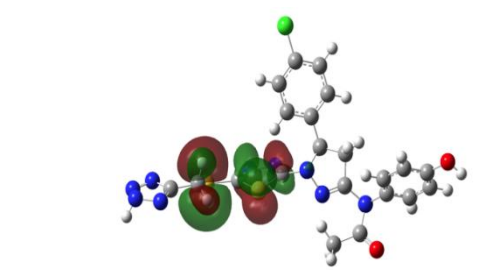</td><td>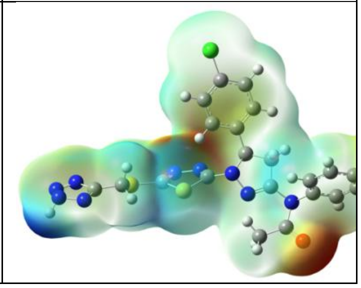</td></tr>
</table>

	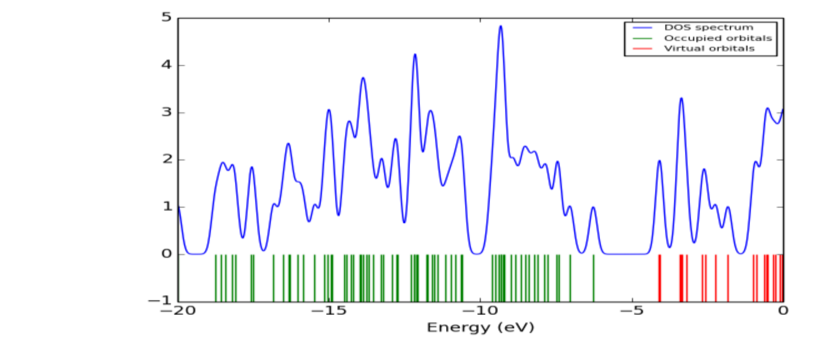DOS spectrum<br>Occupied orbitals<br>Virtual orbitals<br>Energy (eV)	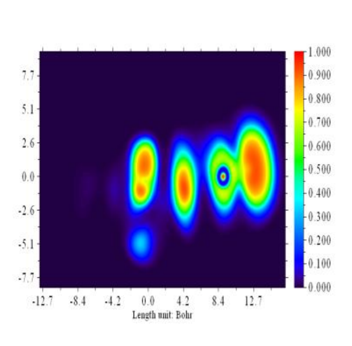Length unit: Bohr
6	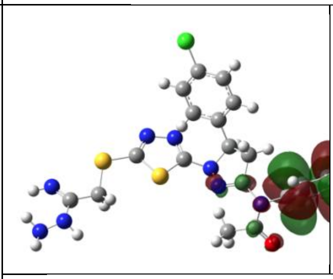 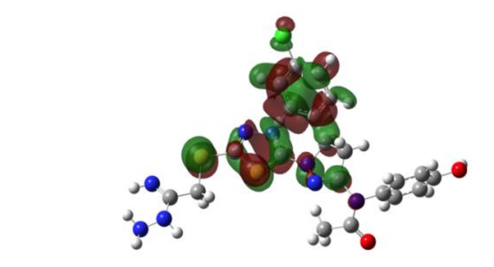	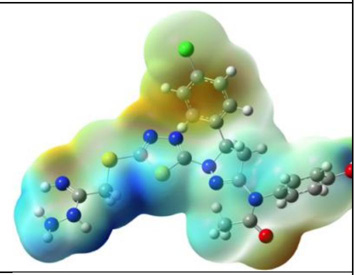
	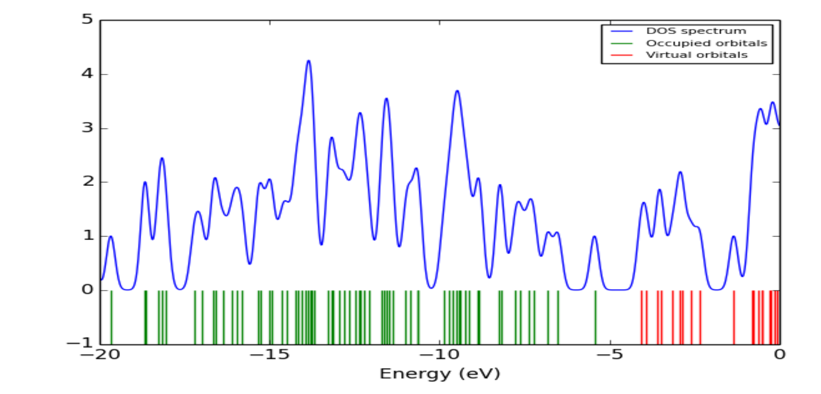DOS spectrum<br>Occupied orbitals<br>Virtual orbitals<br>Energy (eV)	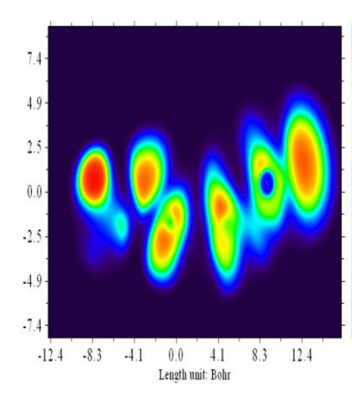Length unit: Bohr
7	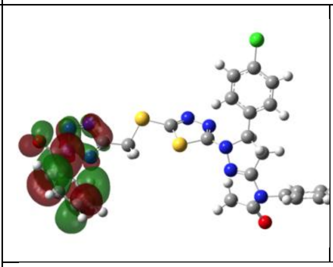 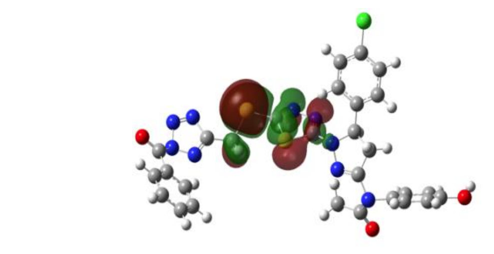	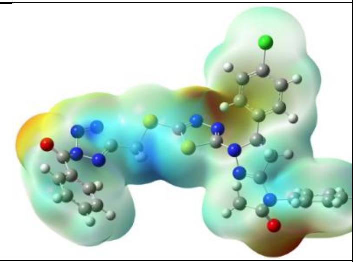
	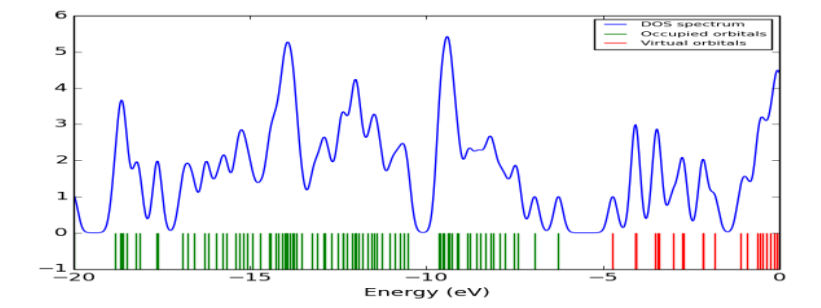DOS spectrum<br>Occupied orbitals<br>Virtual orbitals<br>Energy (eV)	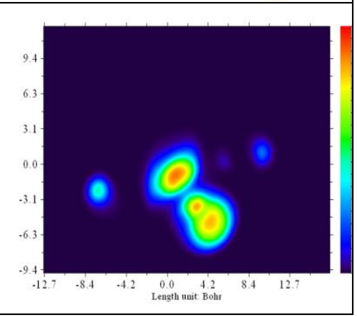Length unit: Bohr

8

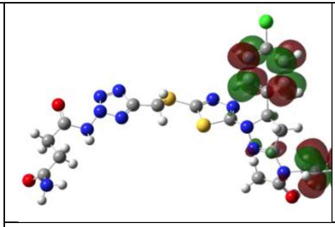

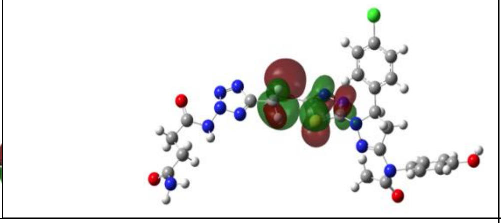

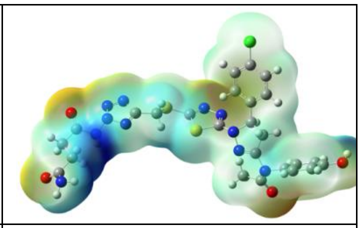

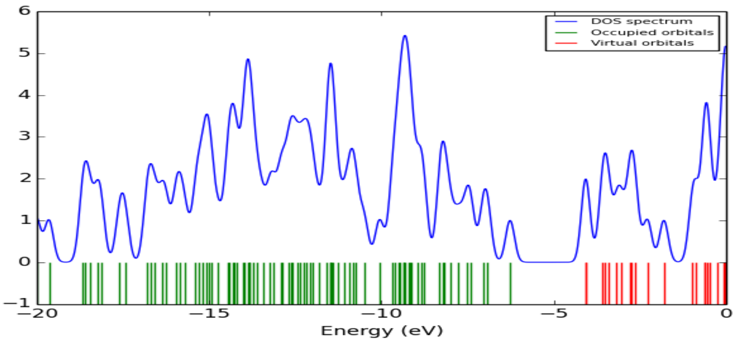
DOS spectrum
Occupied orbitals
Virtual orbitals
Energy (eV)

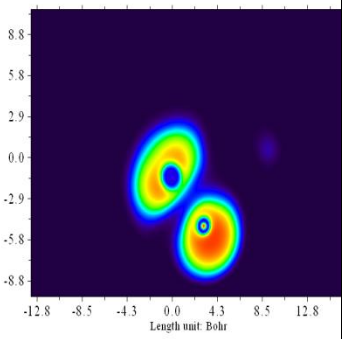
Length unit: Bohr

9

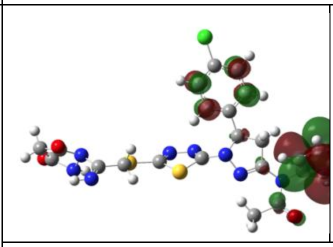

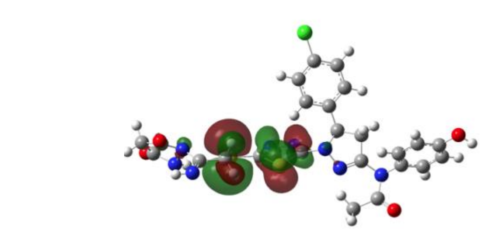

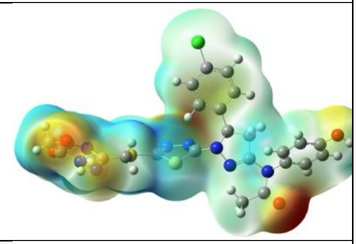

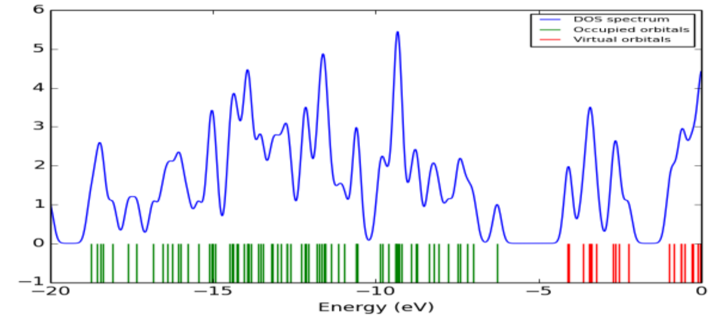
DOS spectrum
Occupied orbitals
Virtual orbitals
Energy (eV)

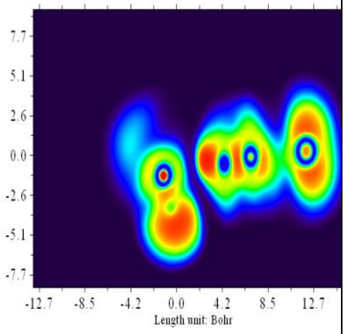
Length unit: Bohr

10

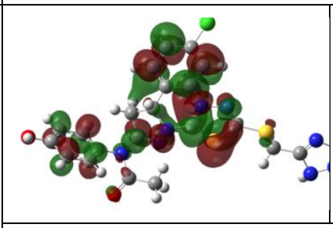

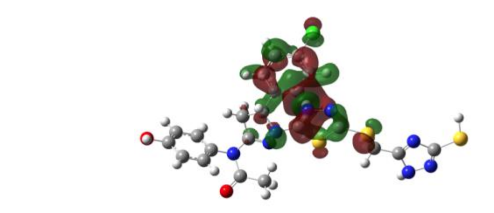

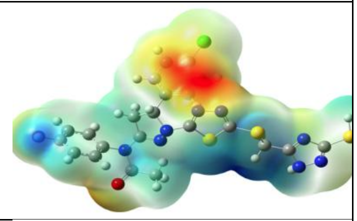

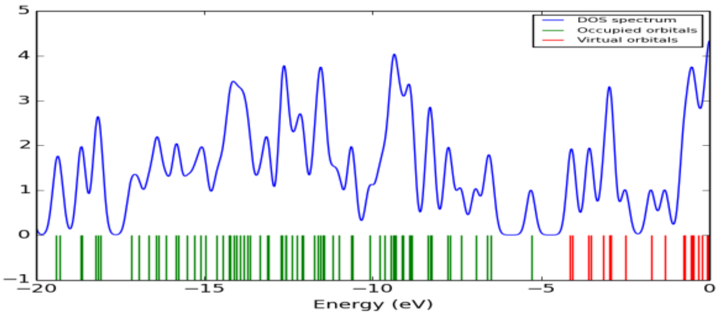
DOS spectrum
Occupied orbitals
Virtual orbitals
Energy (eV)

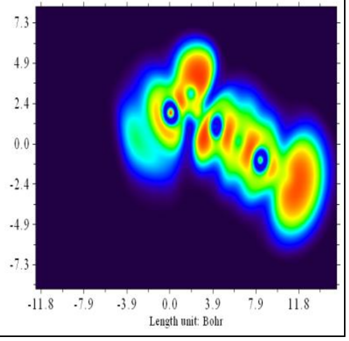
Length unit: Bohr

11	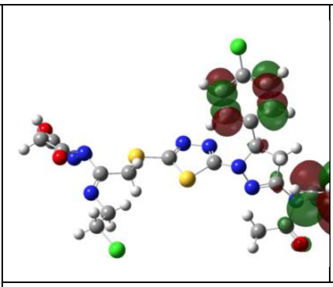	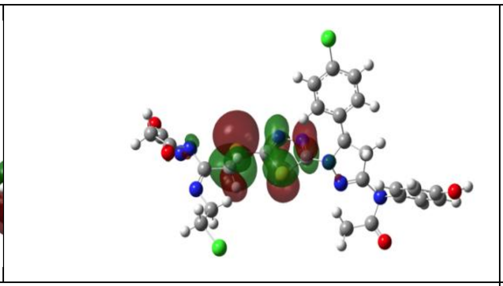	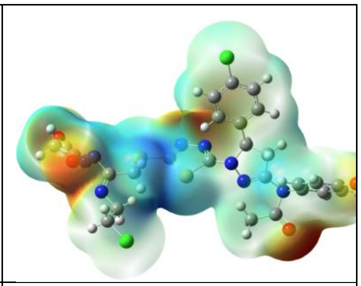
	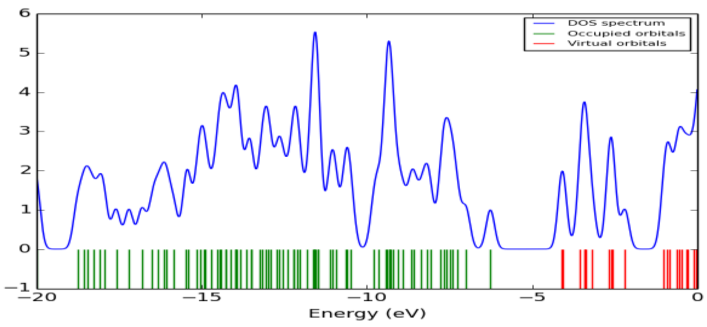 DOS spectrum Occupied orbitals Virtual orbitals Energy (eV)		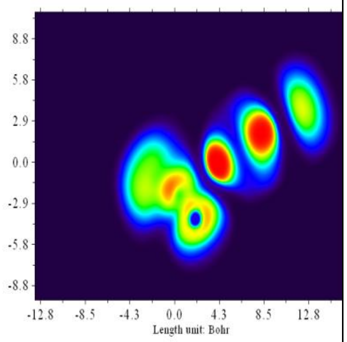 Length unit: Bohr
12	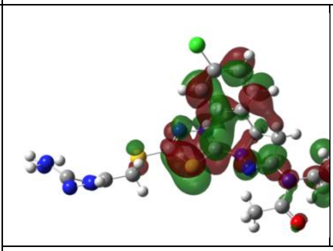	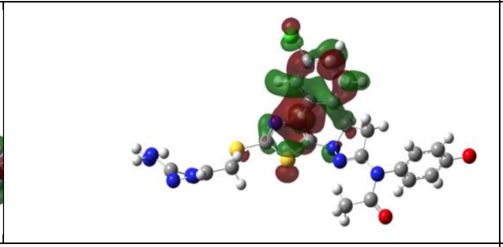	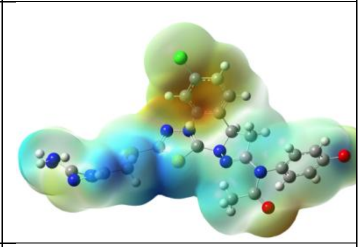
	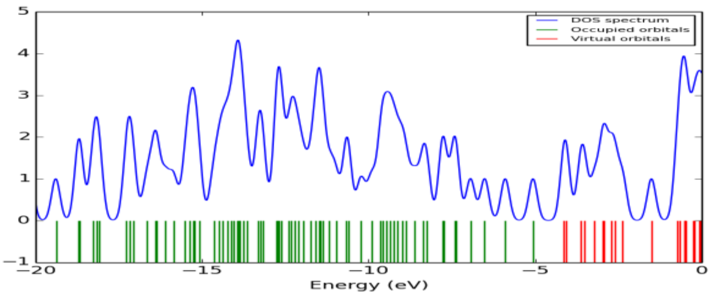 DOS spectrum Occupied orbitals Virtual orbitals Energy (eV)		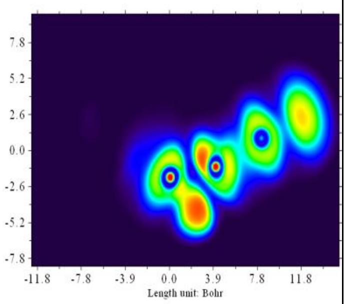 Length unit: Bohr
13	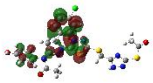	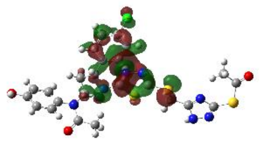	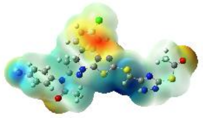

	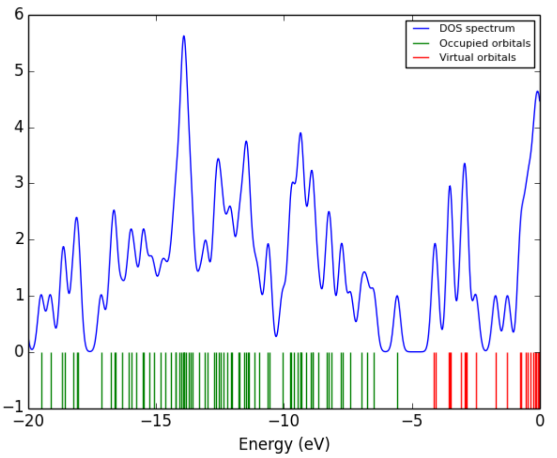		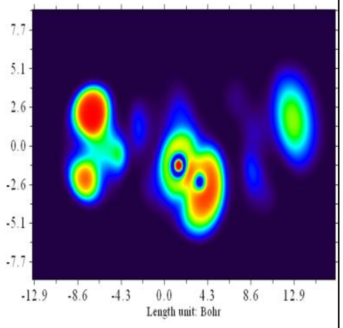
14	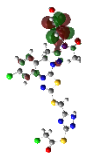	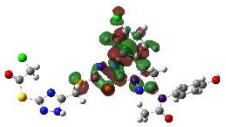	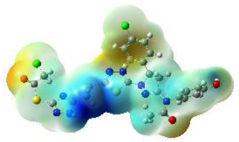
	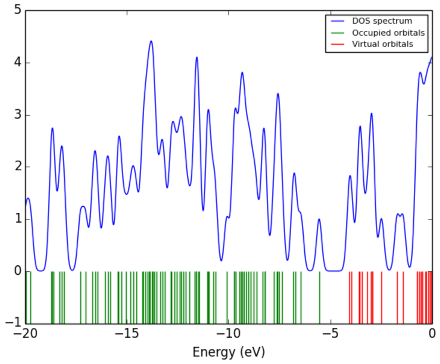		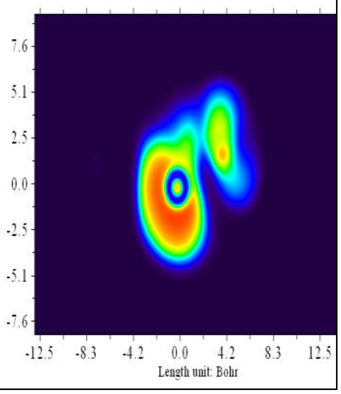

Table S17. Noncovalent interaction (NCI) and reduced density gradient (RDG) analyses of the investigated compounds **1-14**. For each compound, the table presents the NCI scatter plot illustrating attractive, weak, and repulsive noncovalent interactions, and 3D RDG isosurfaces mapped onto the molecular structure, highlighting regions associated with stabilizing and destabilizing intermolecular interactions.

File name	NCI	RDG
1	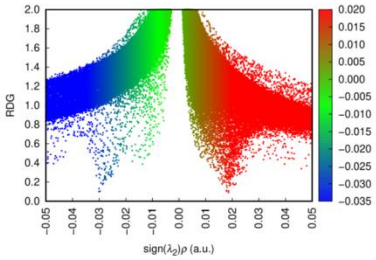	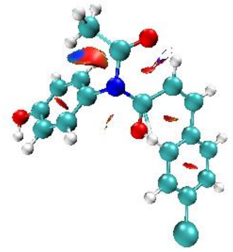
2	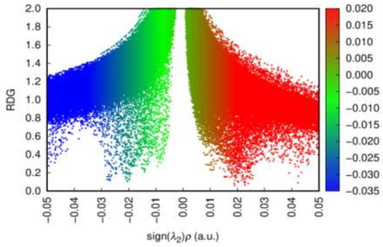	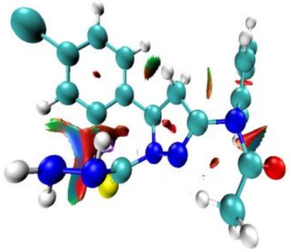
3	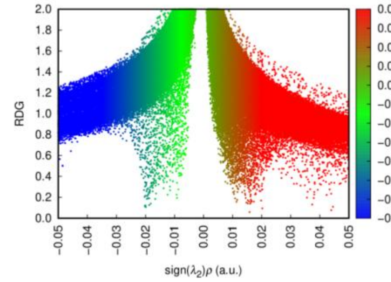	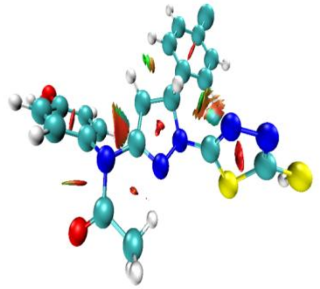
4	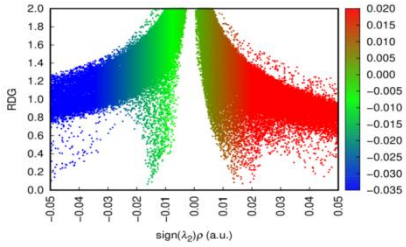	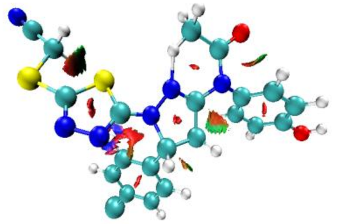

**5**	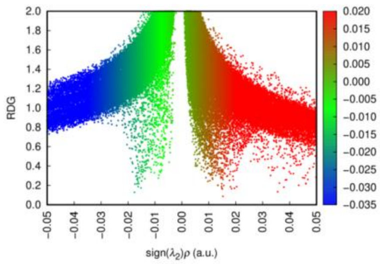 RDG sign(λ_2)ρ (a.u.)	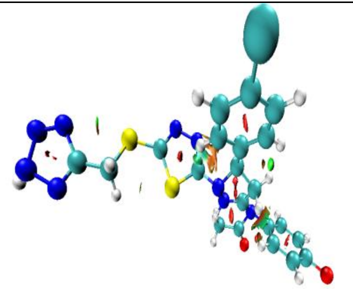
**6**	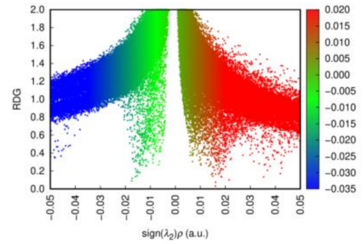 RDG sign(λ_2)ρ (a.u.)	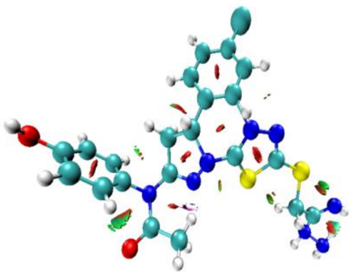
**7**	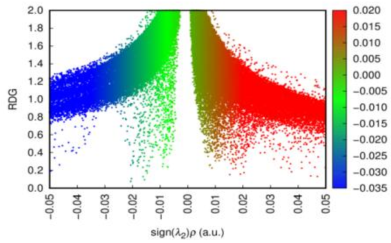 RDG sign(λ_2)ρ (a.u.)	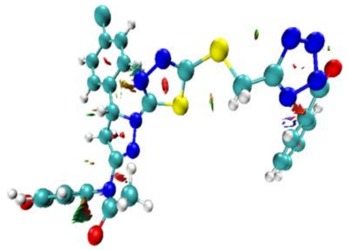
**8**	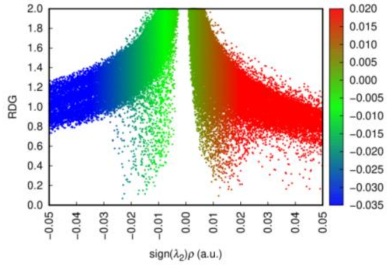 RDG sign(λ_2)ρ (a.u.)	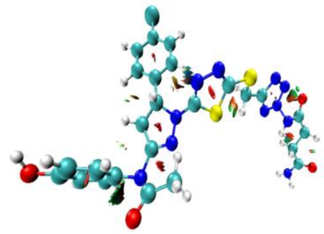
**9**	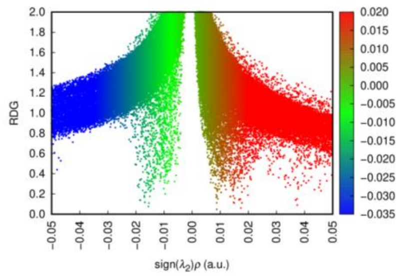 RDG sign(λ_2)ρ (a.u.)	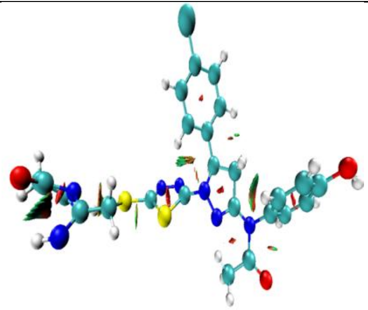

**10**	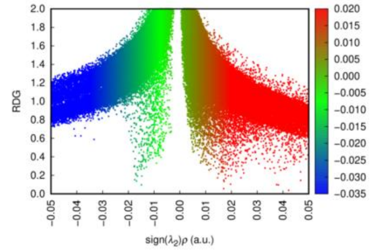	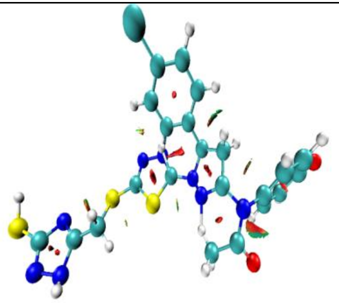
**11**	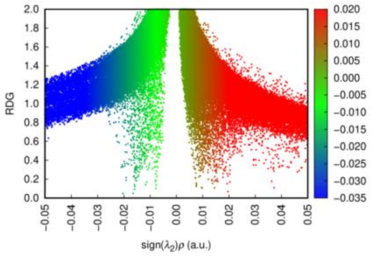	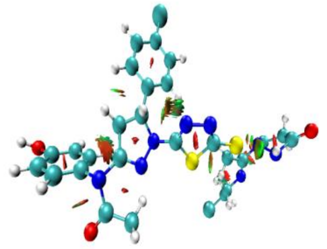
**12**	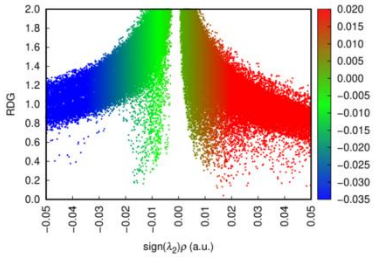	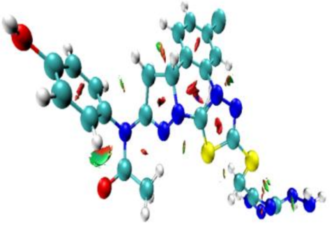
**13**	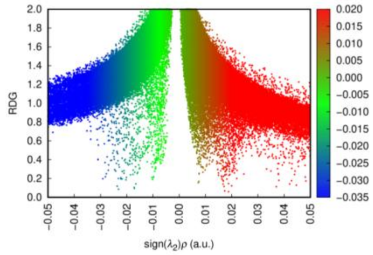	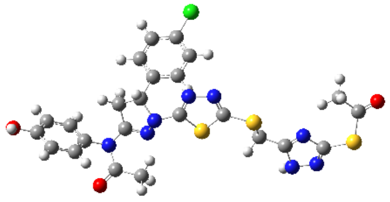
**14**	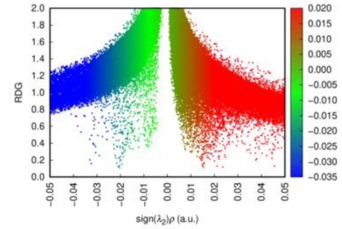	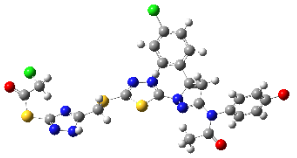

Copies of IR, ^{1}H NMR, ^{13}C NMR, and mass spectra of Compounds

Characterization of Compound 1:

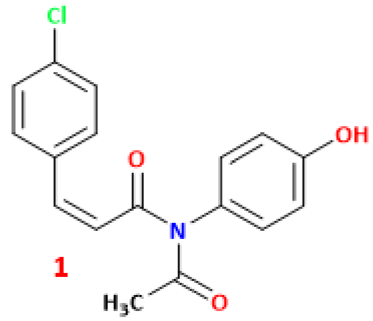

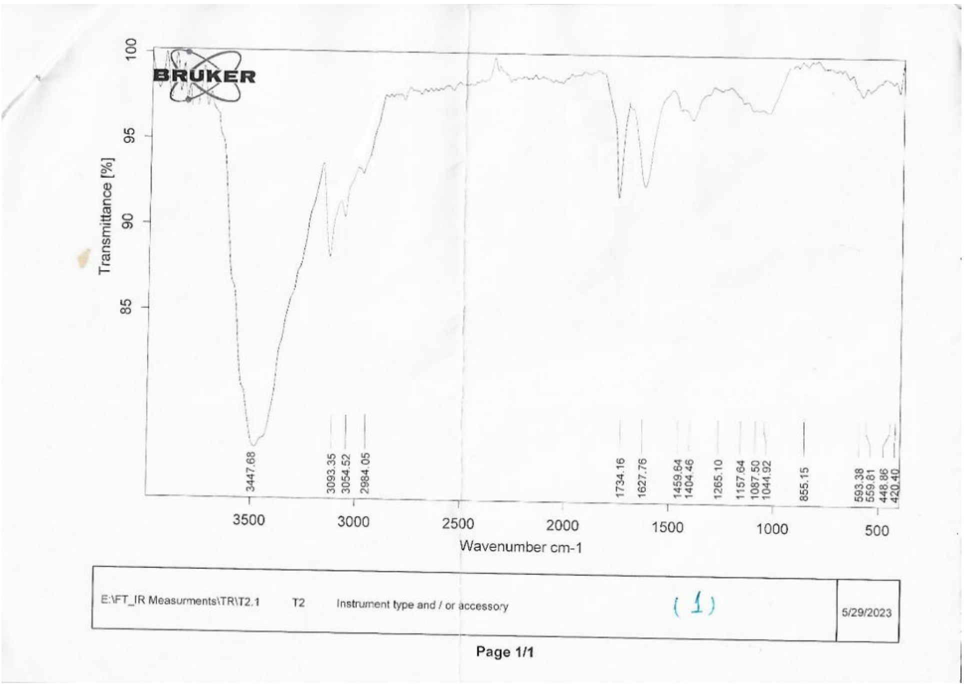

Figure S1. IR of Compound **1**

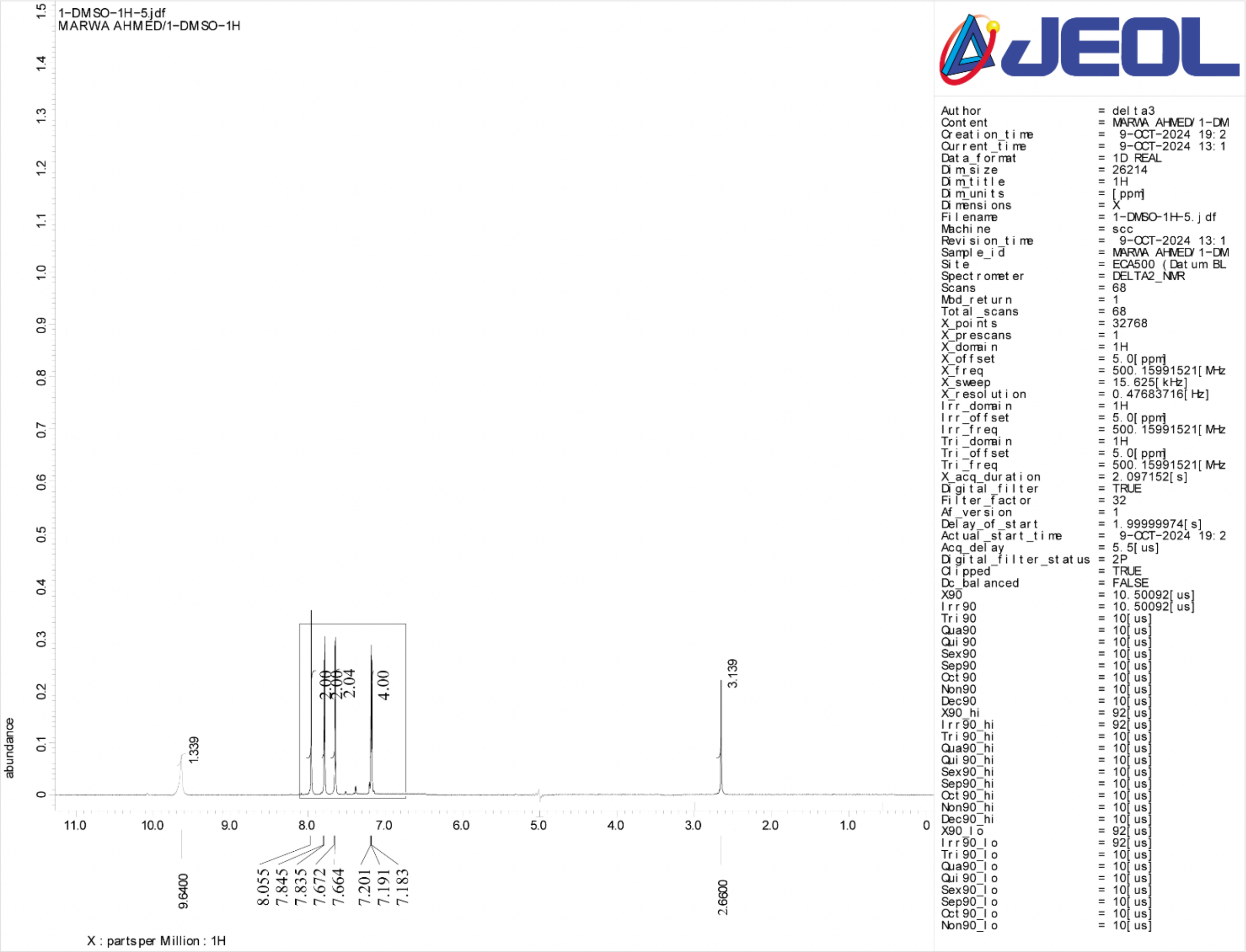

Figure S2. ^{1}H NMR spectrum (400 MHz, DMSO) of compound **1**

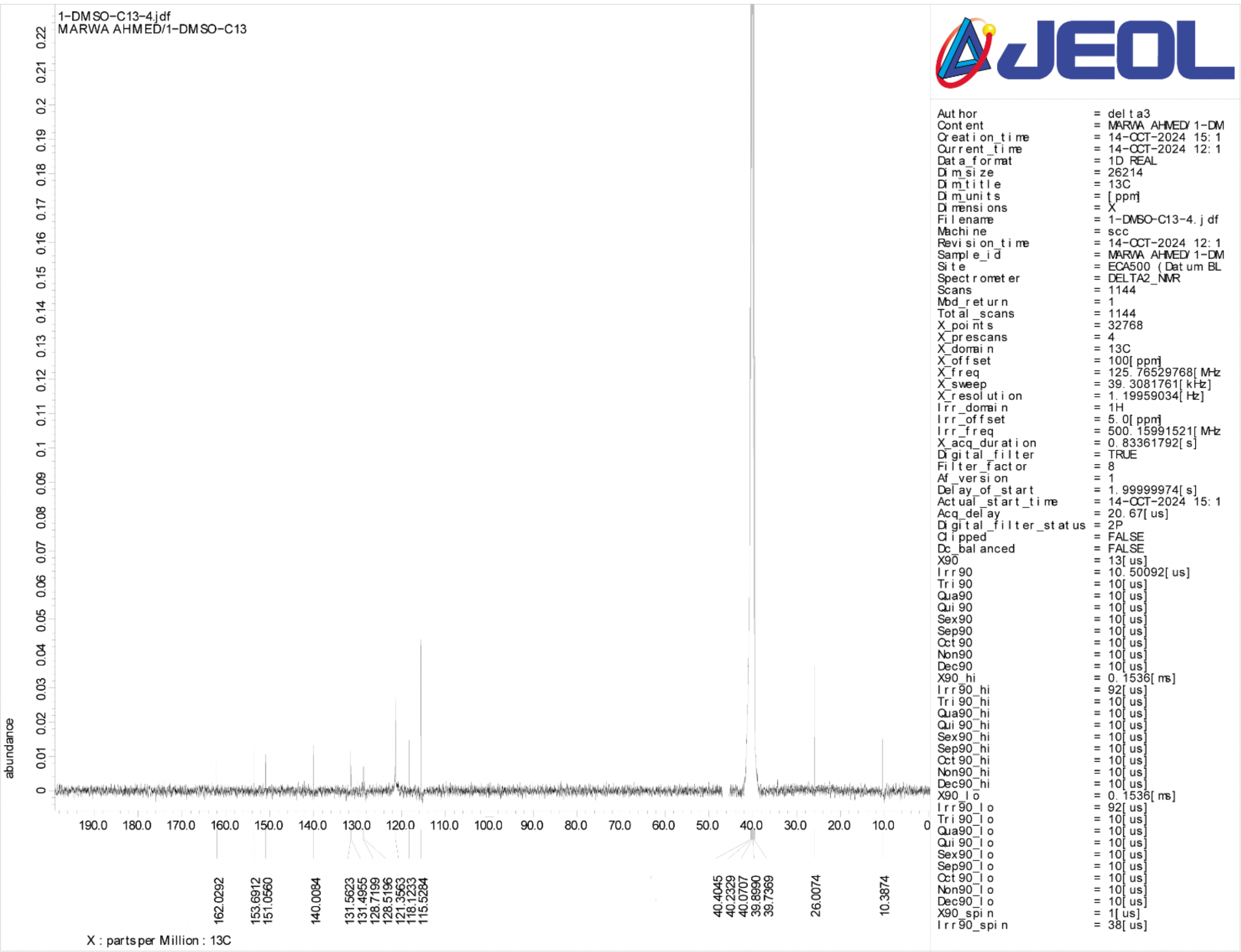

Figure S3. ^{13}C NMR spectrum (100 MHz, DMSO) of compound **1**

Characterization of Compound 2:

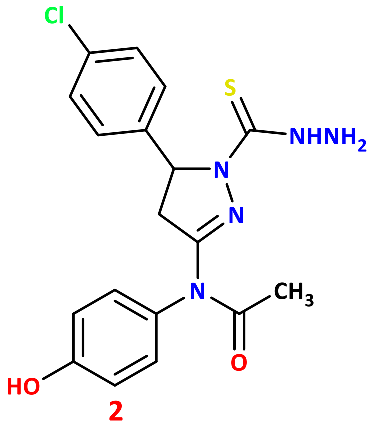

2

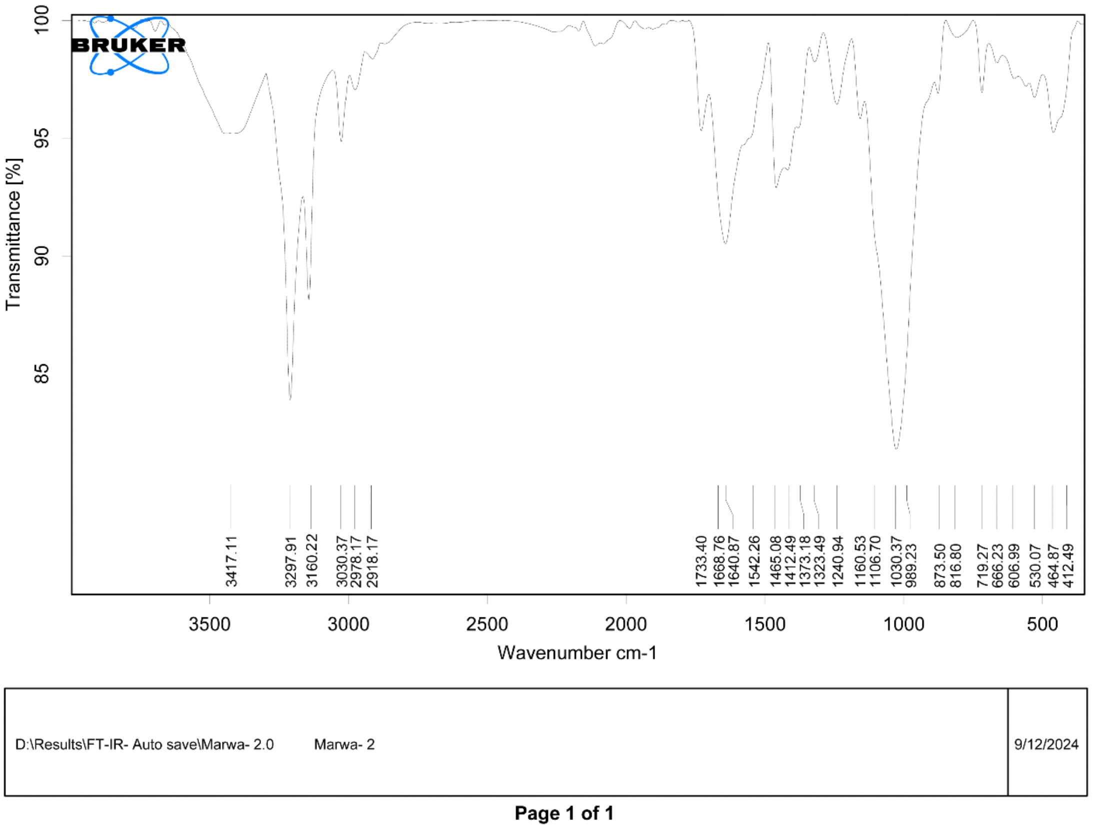

Figure S4. IR of Compound **2**

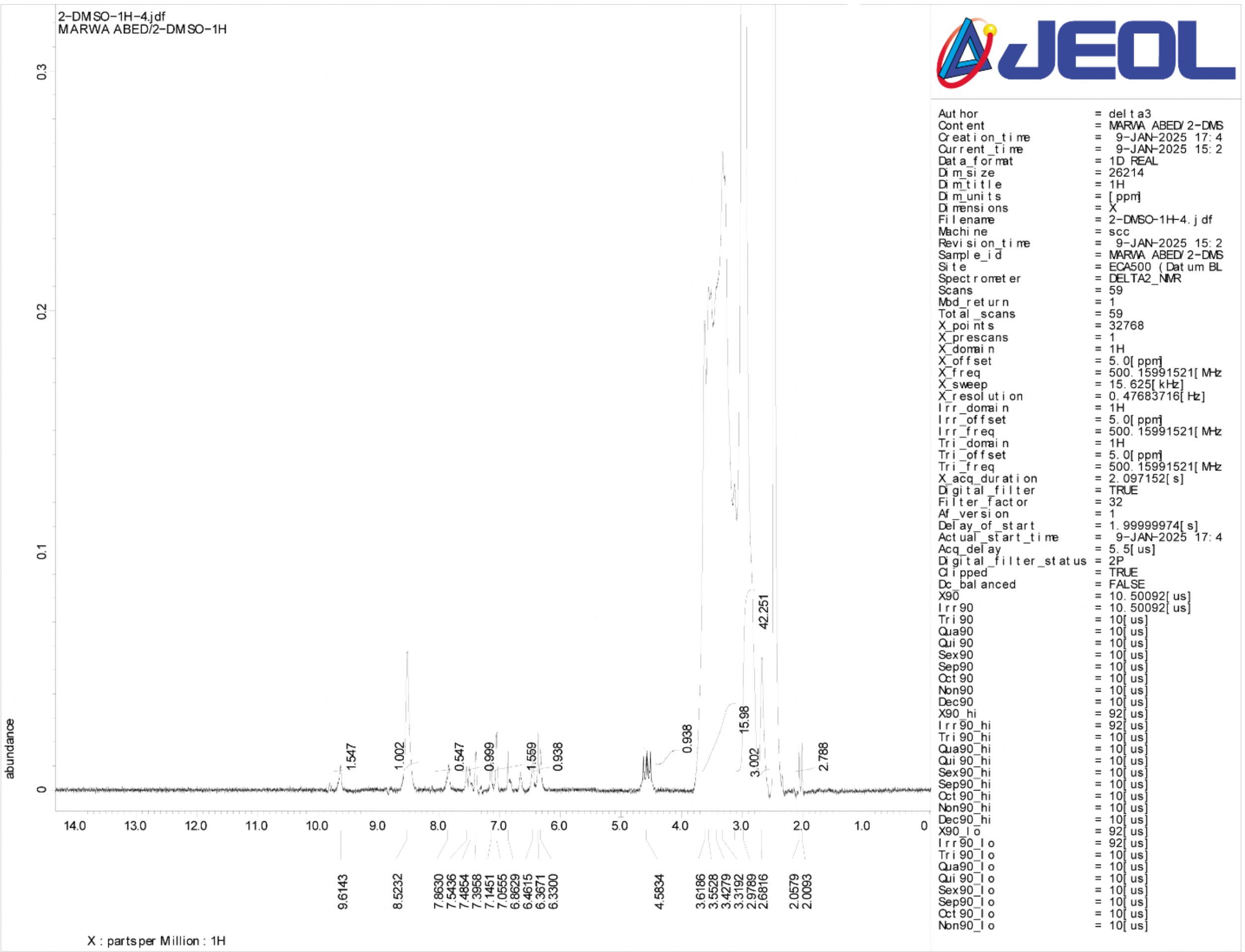

Figure S5. ^{1}H NMR spectrum (400 MHz, DMSO) of compound **2**

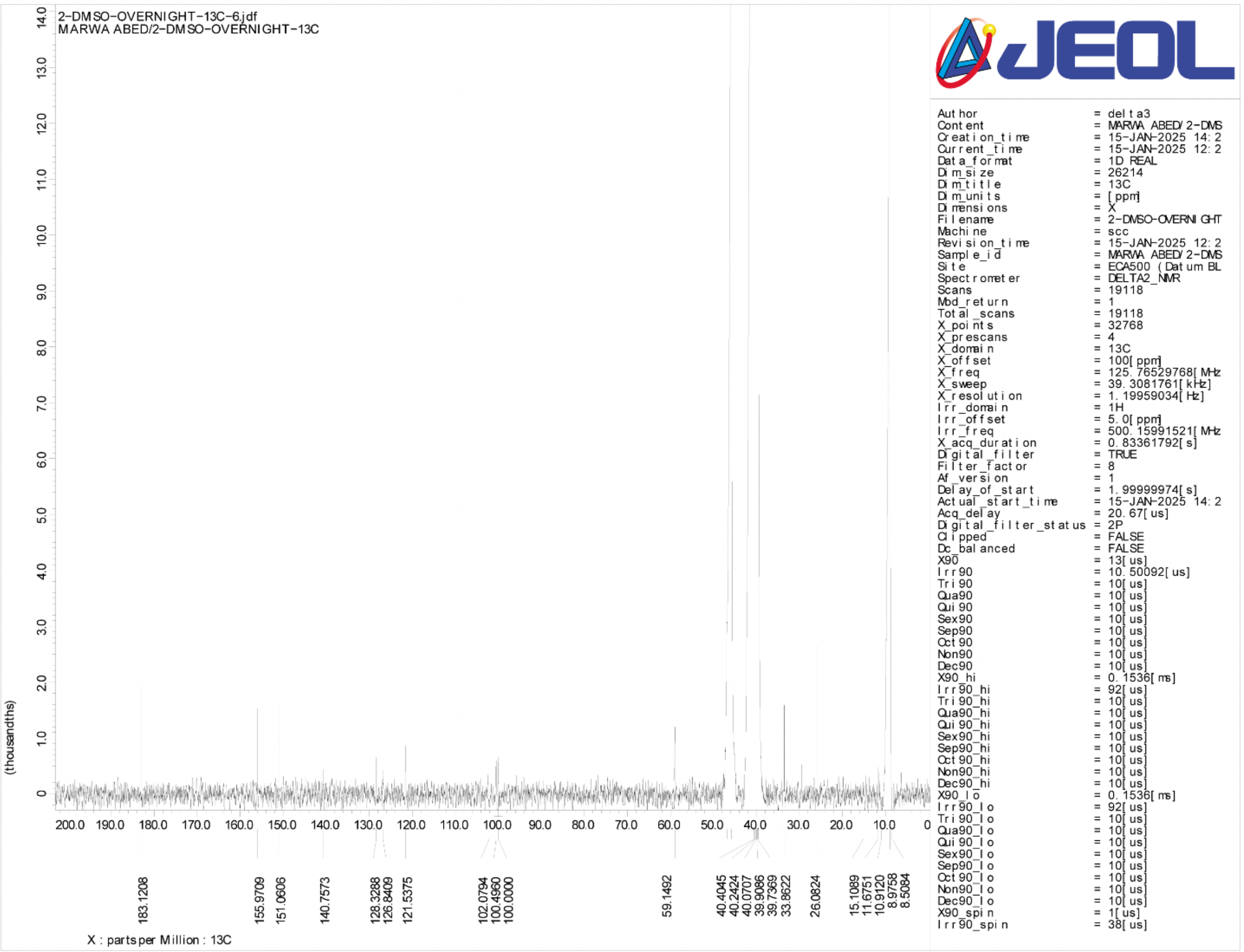

Figure S6. ^{13}C NMR spectrum (100 MHz, DMSO) of compound **2**

Characterization of Compound 3:

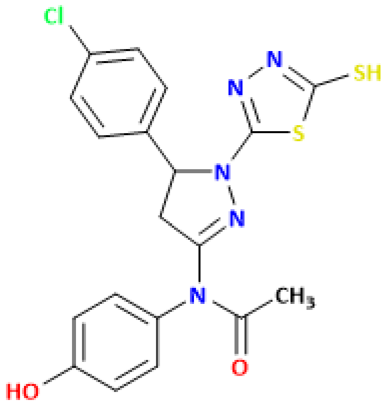

3

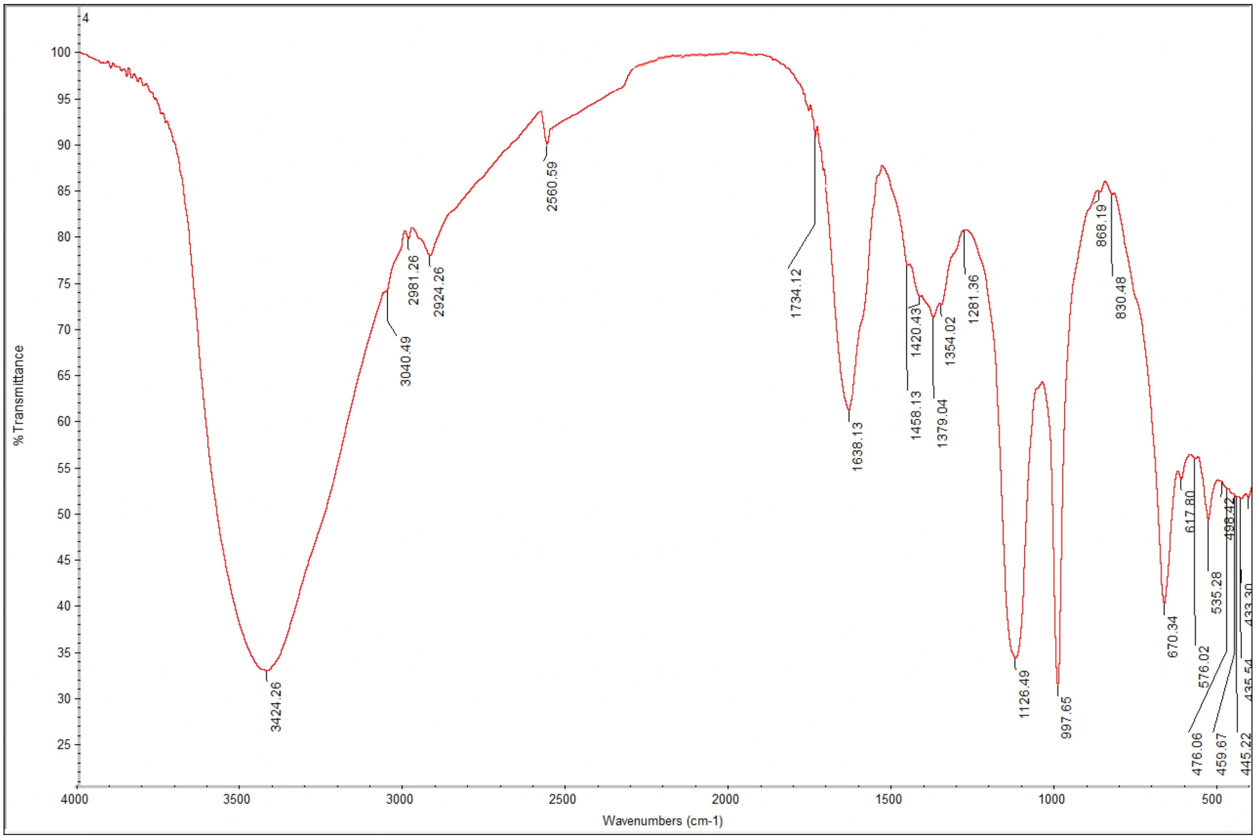

Figure S7. IR of Compound **3**

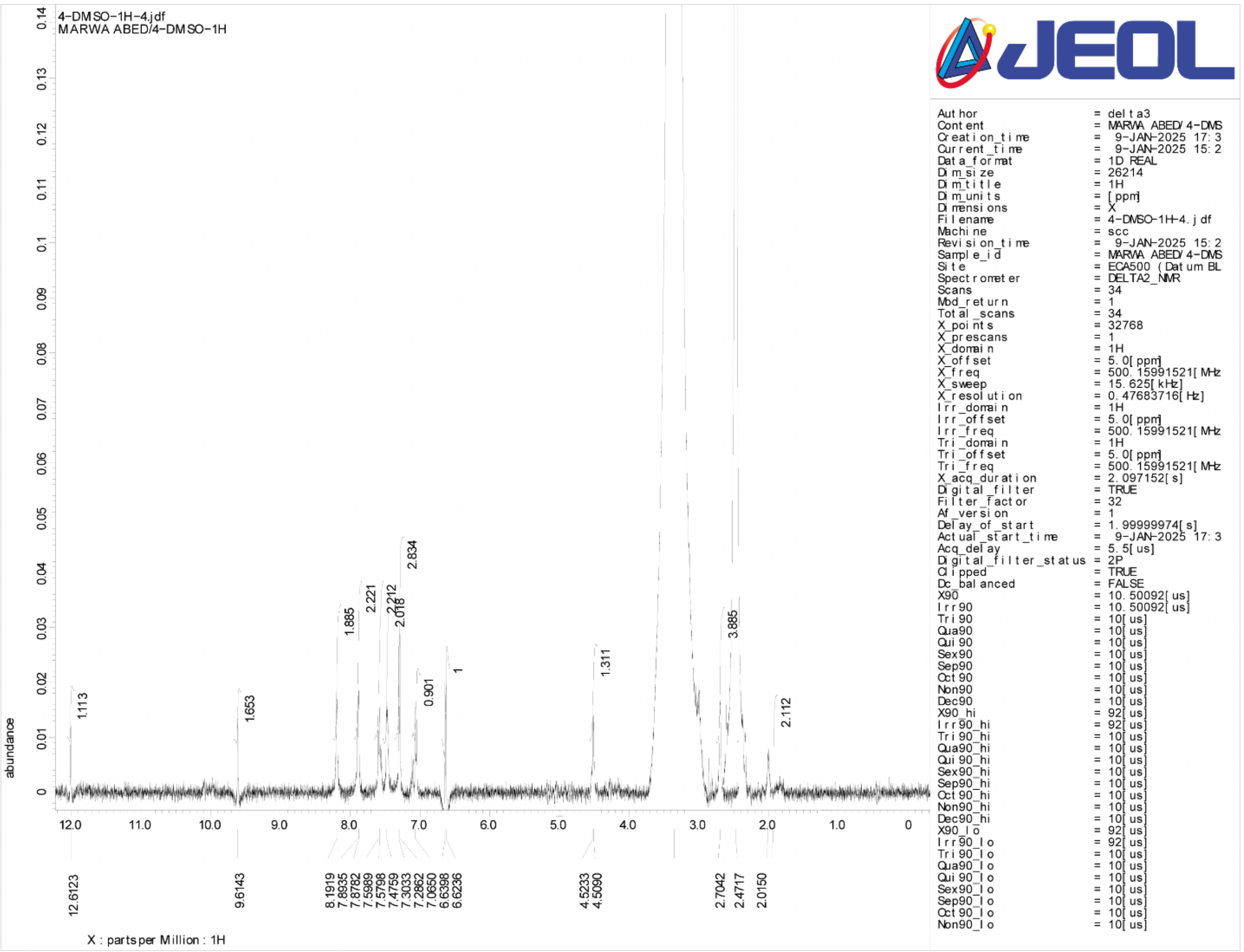

Figure S8. ^{1}H NMR spectrum (400 MHz, DMSO) of compound **3**

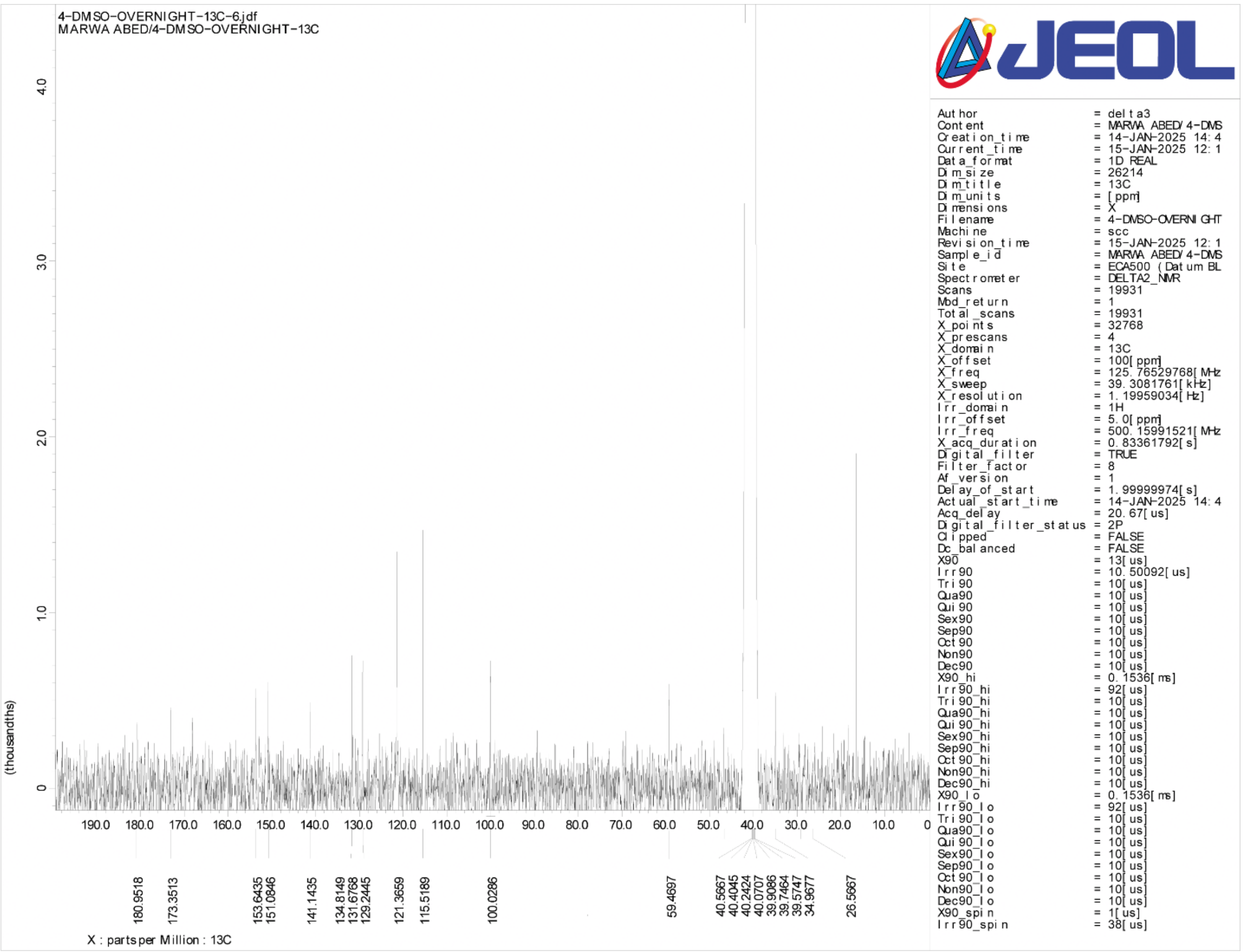

Figure S9. ^{13}C NMR spectrum (100 MHz, DMSO) of compound **3**

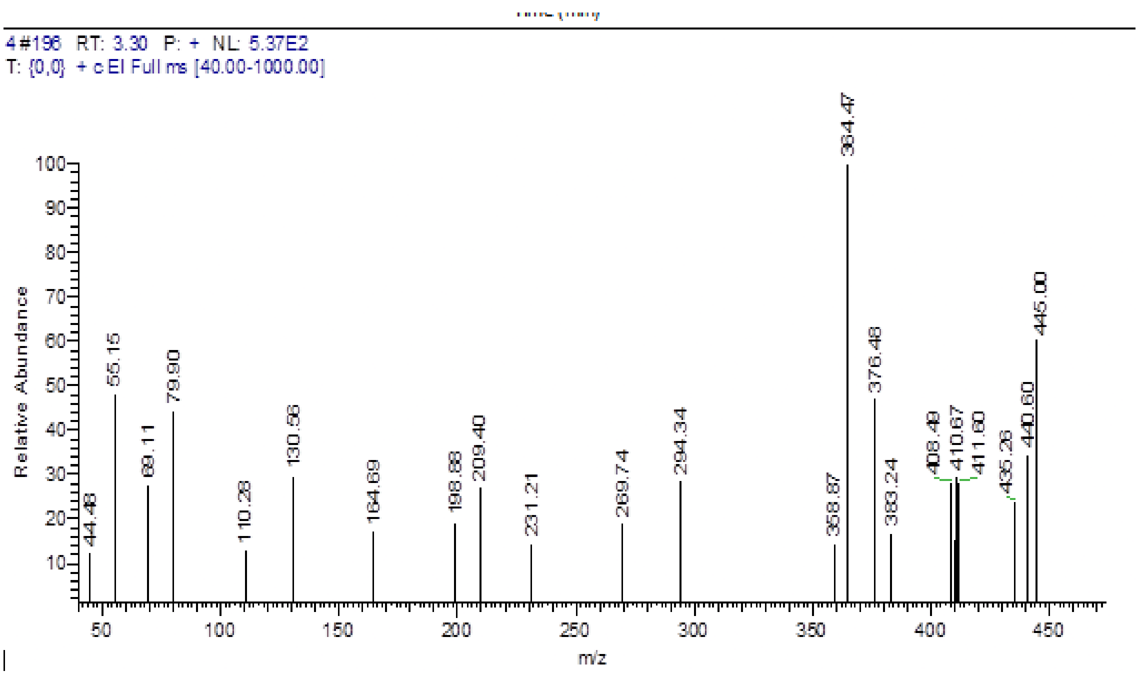

Figure S10. Mass spectrum of compound 3

Characterization of Compound 4:

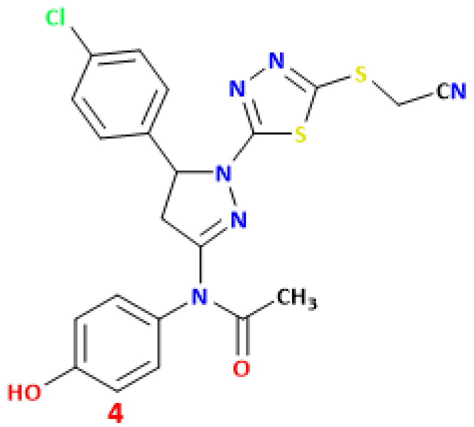

4

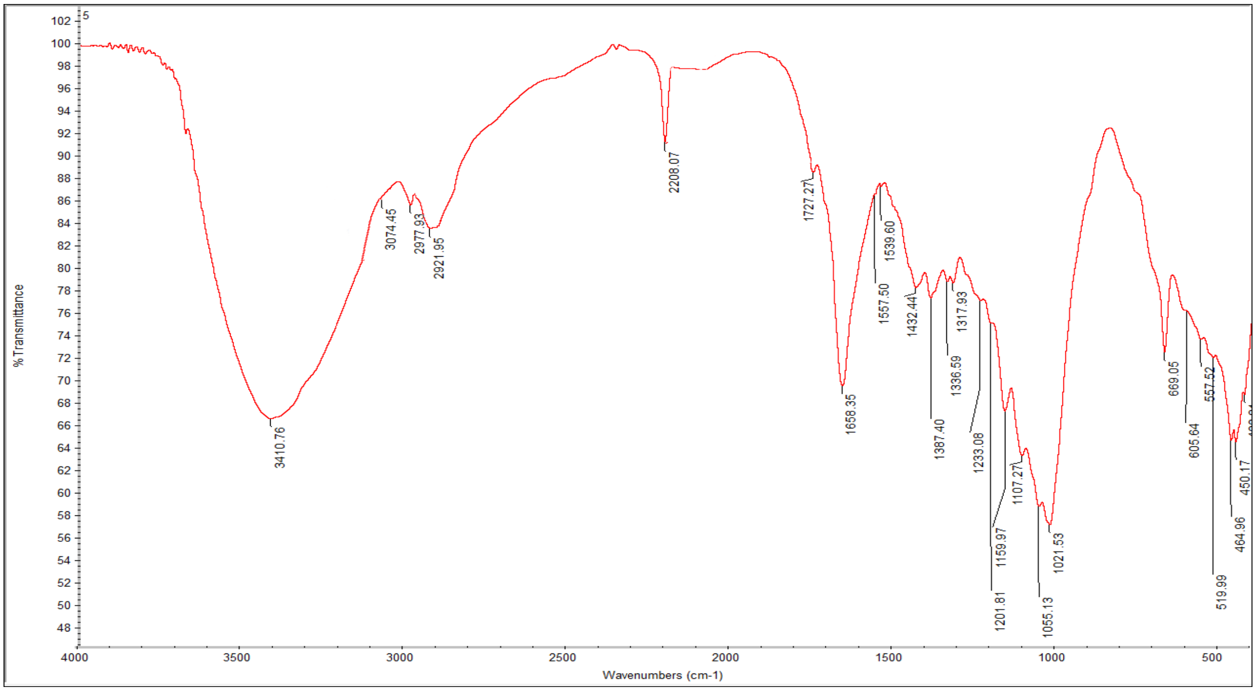

Figure S11. IR of Compound **4**

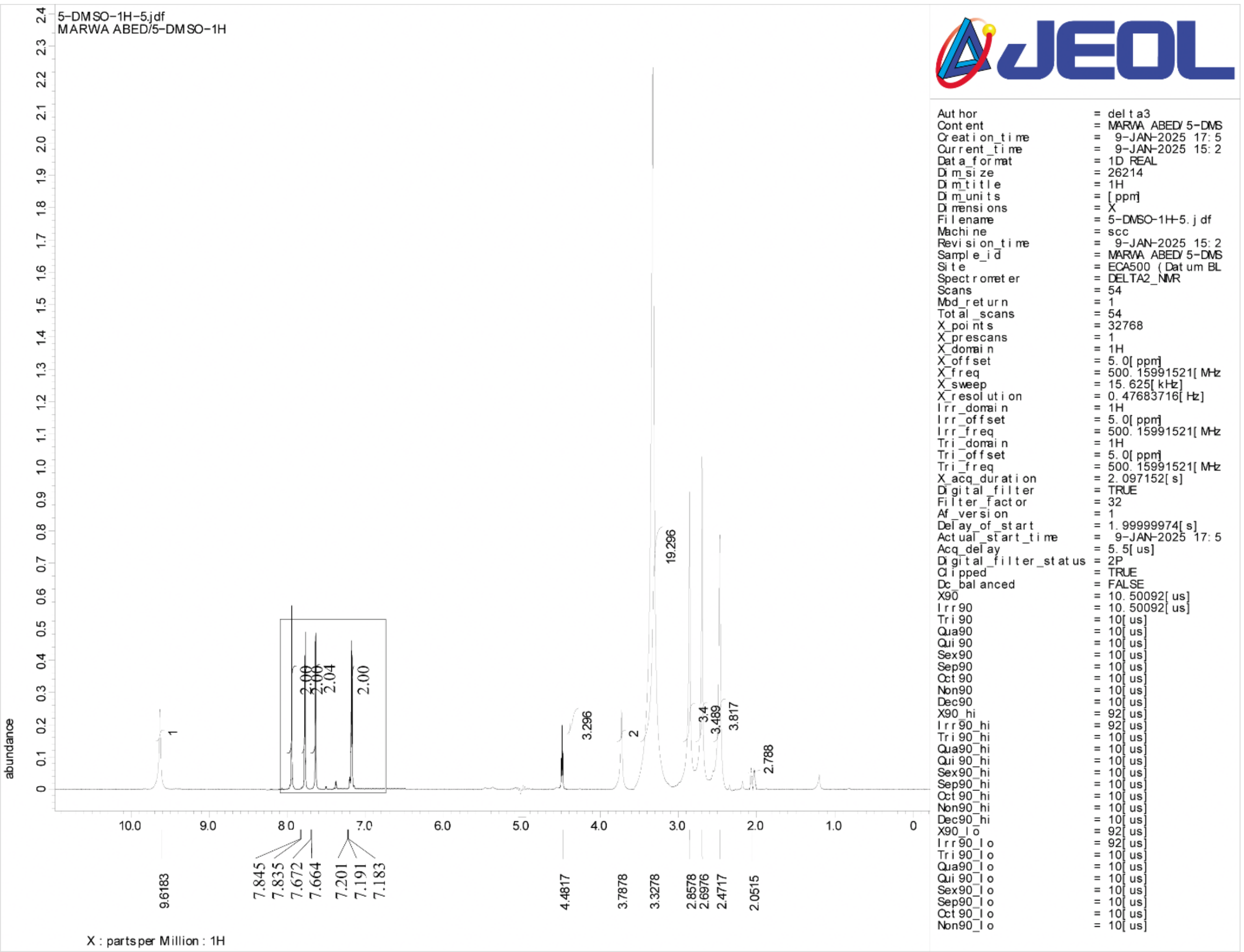

Figure S12. ^{1}H NMR spectrum (400 MHz, DMSO) of compound **4**

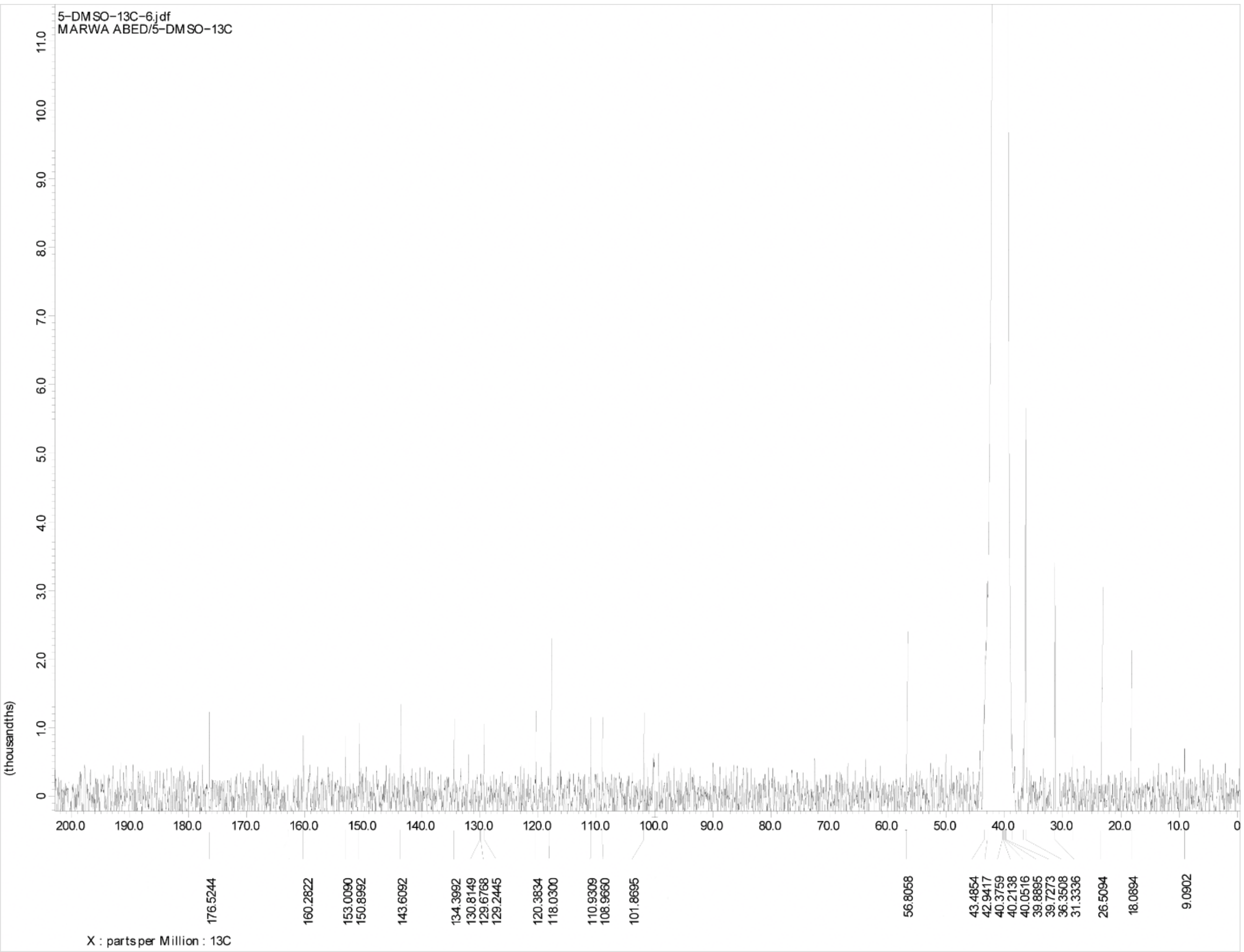

Figure S13. ^{13}C NMR spectrum (100 MHz, DMSO) of compound **4**

Characterization of Compound 5:

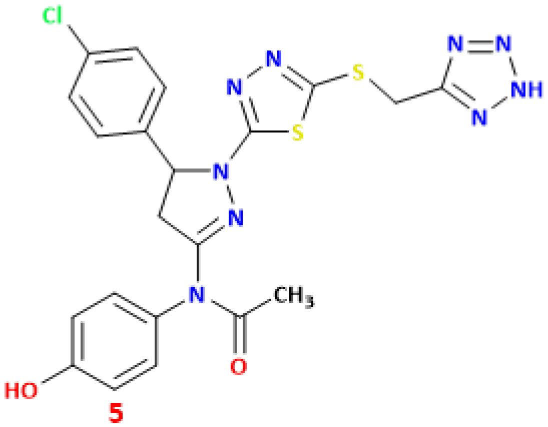

5

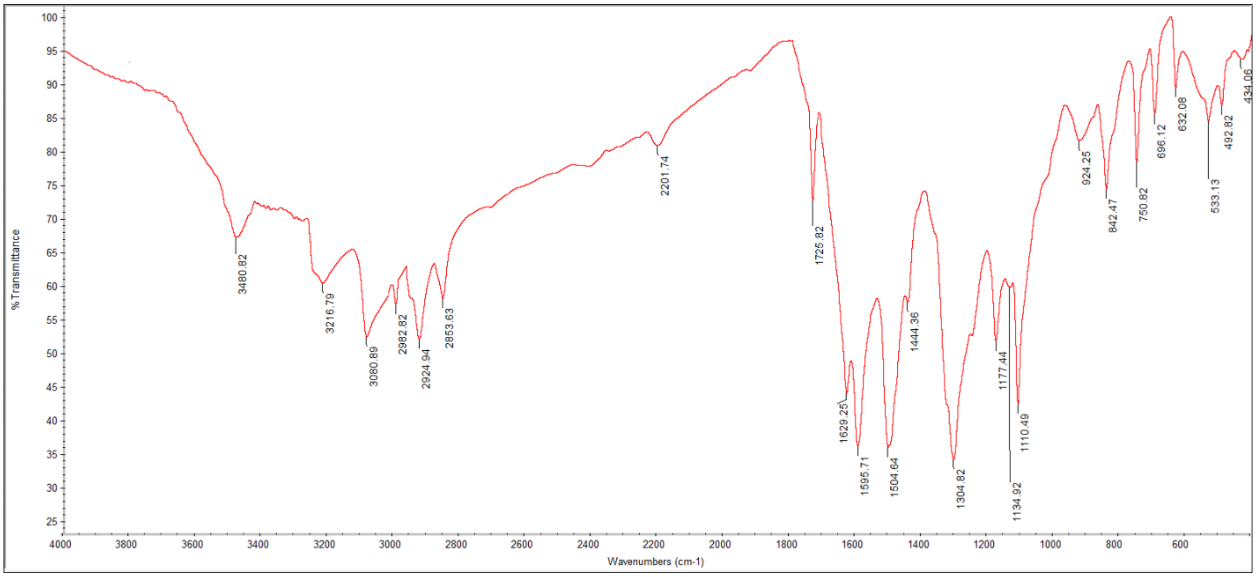

Figure S14. IR of Compound 5

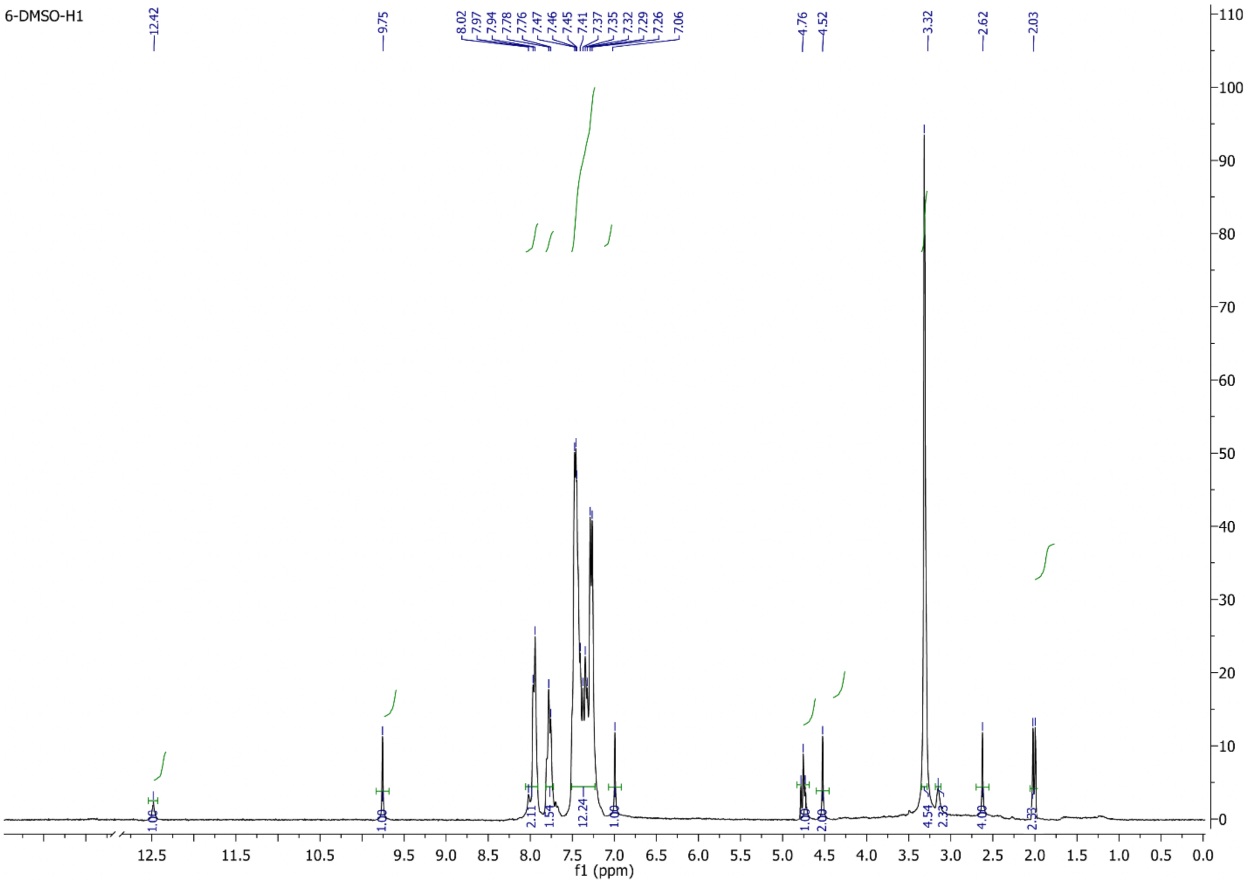

Figure S15. ^{1}H NMR spectrum (400 MHz, DMSO) of compound **5**

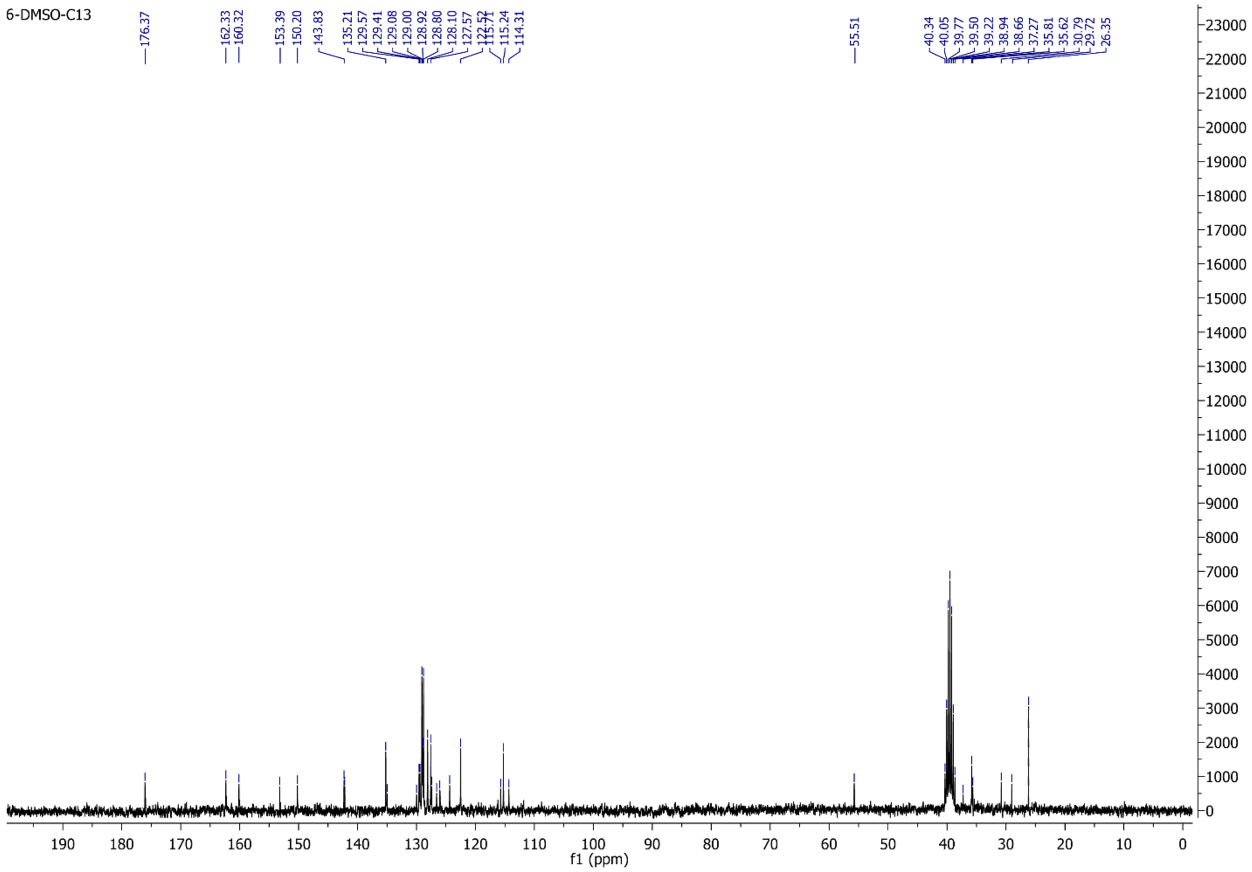

Figure S16. ^{13}C NMR spectrum (100 MHz, DMSO) of compound **5**

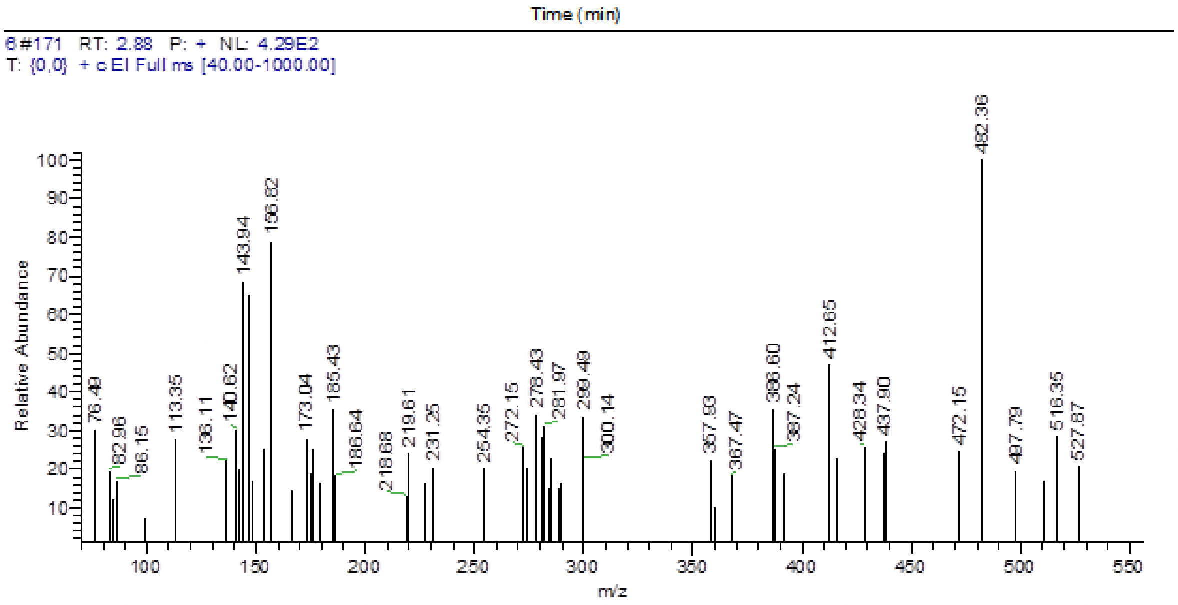

Figure S17. Mass spectrum of compound 3

Characterization of Compound 6:

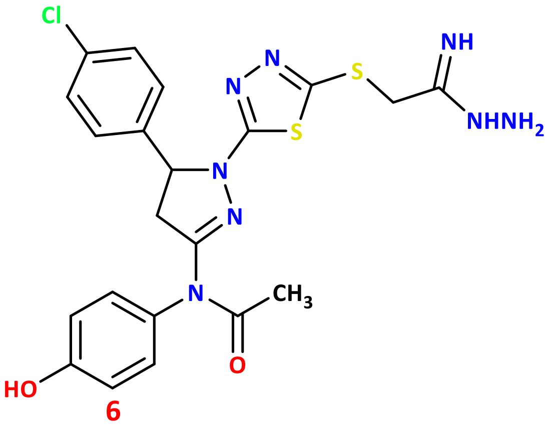

6

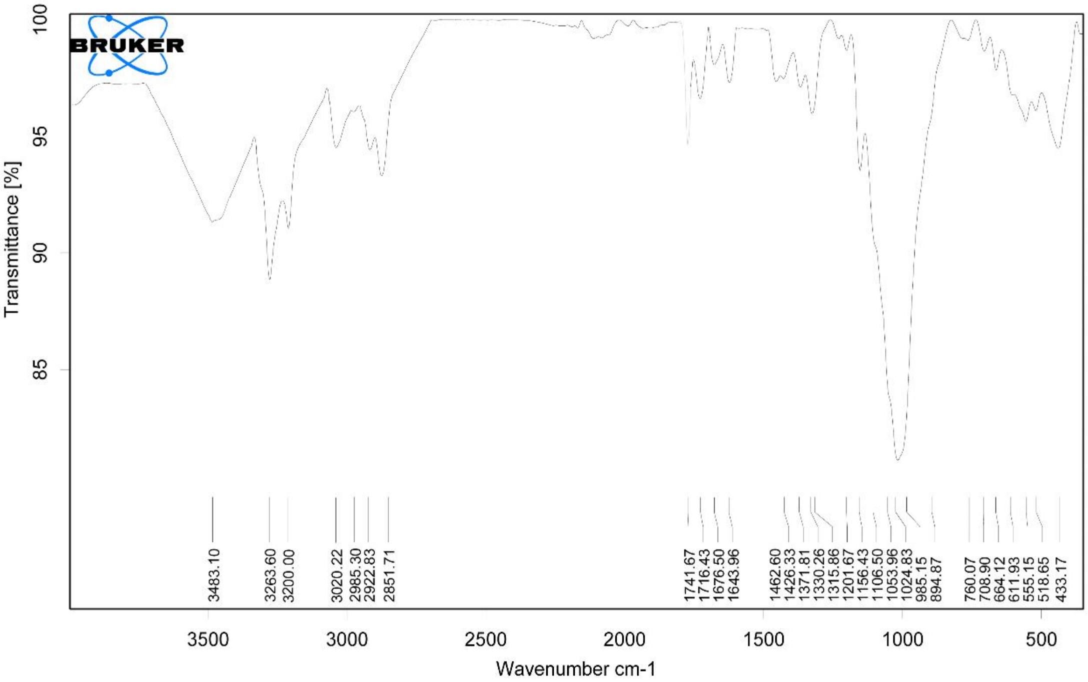

D:\Results\FT-IR- Auto save\Marwa- 7.0 Marwa- 7	9/12/2024

Page 1 of 1

Figure S18. IR of Compound **6**

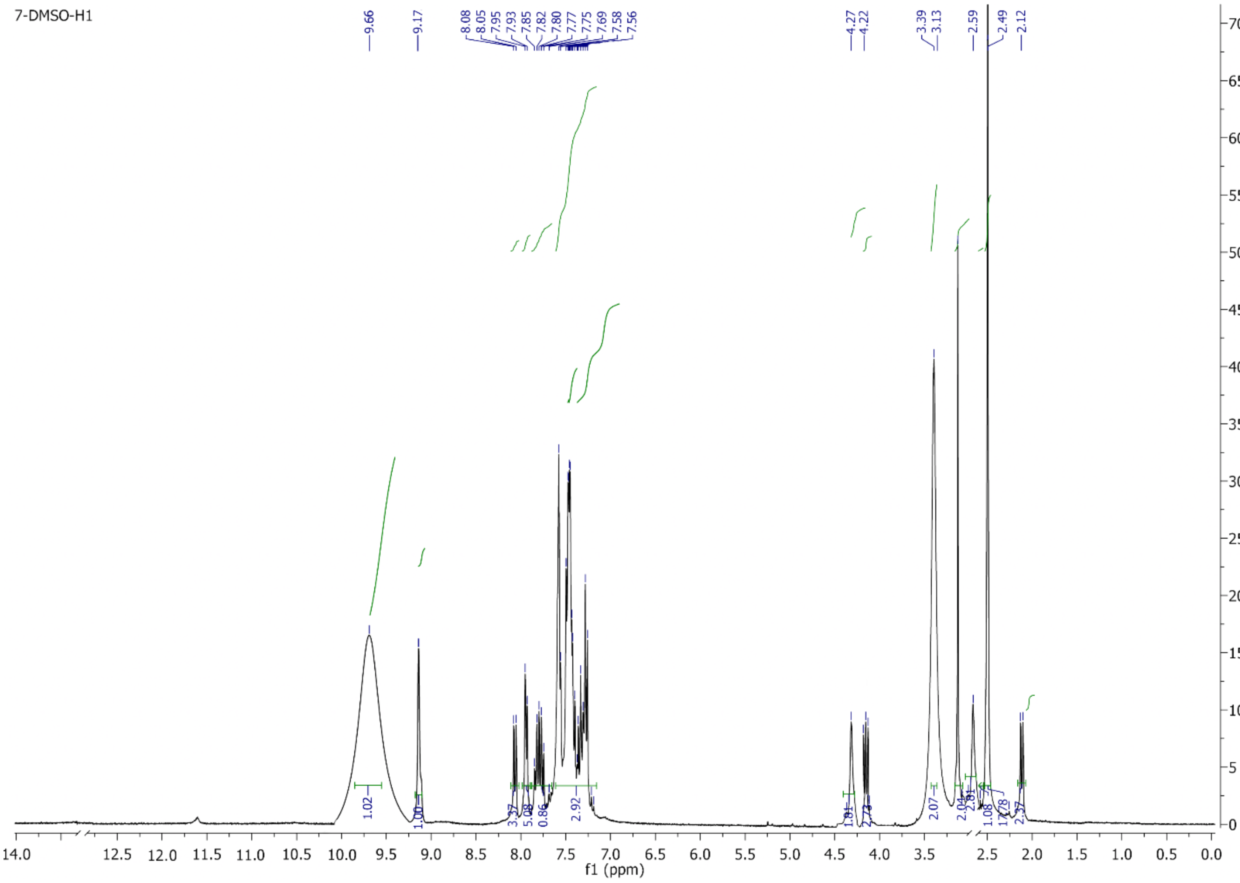

Figure S19. ^{1}H NMR spectrum (400 MHz, DMSO) of compound **6**

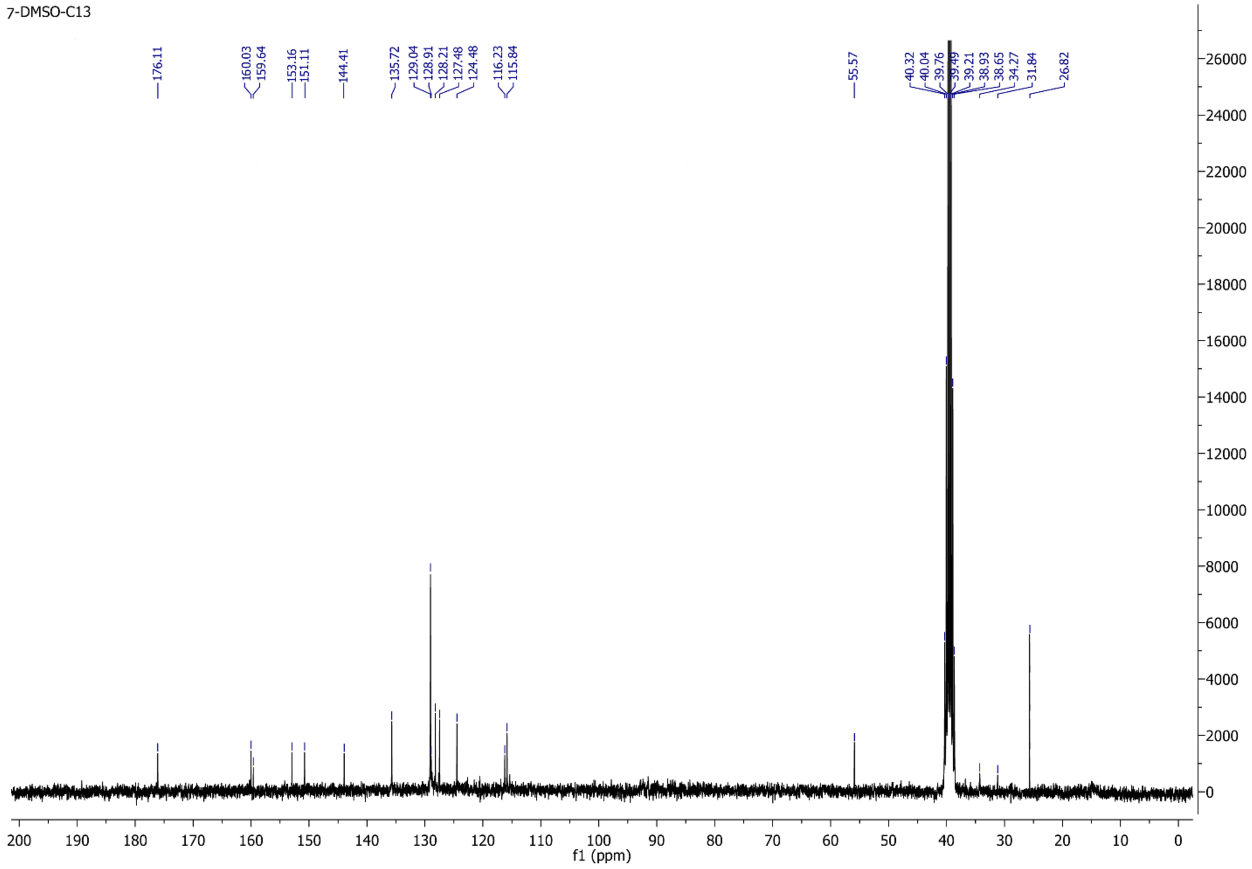

Figure S20. ^{13}C NMR spectrum (100 MHz, DMSO) of compound **6**

Characterization of Compound 7:

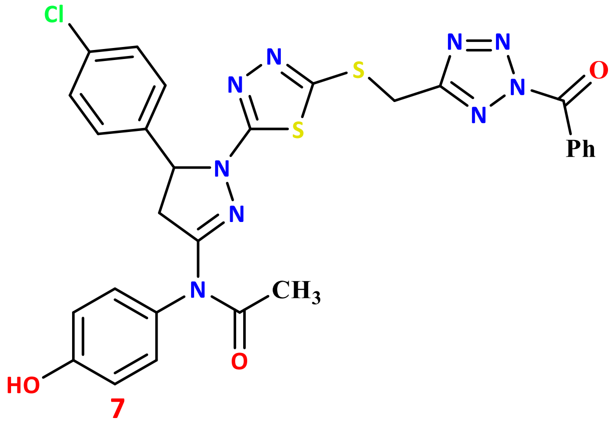

7

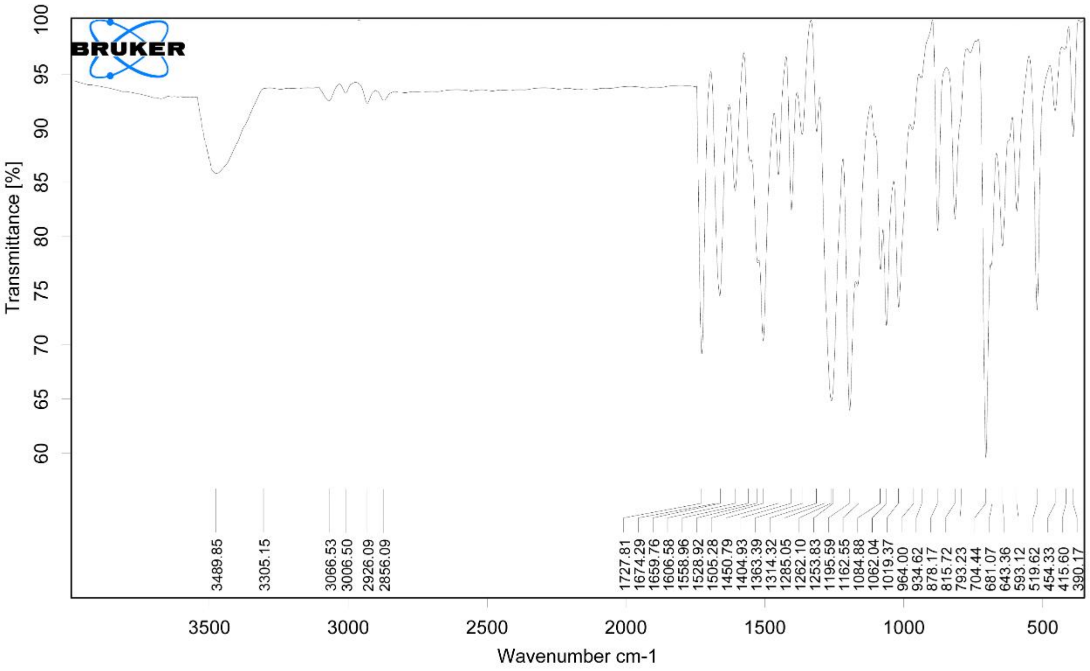

D:\Results\FT-IR- Auto save\Marwa- 8.0 Marwa- 8	9/12/2024

Page 1 of 1

Figure S21. IR of Compound **7**

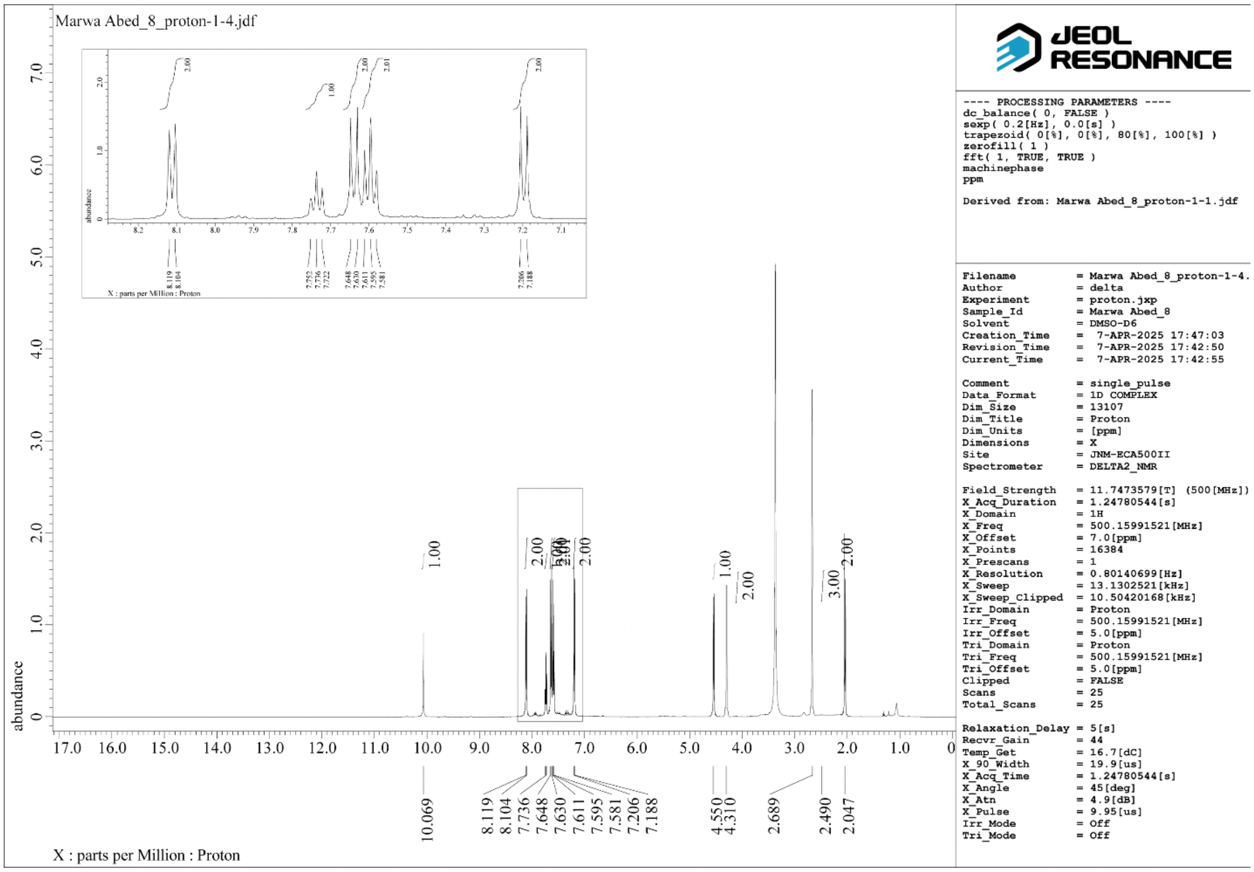

Figure S22. ^{1}H NMR spectrum (400 MHz, DMSO) of compound **7**

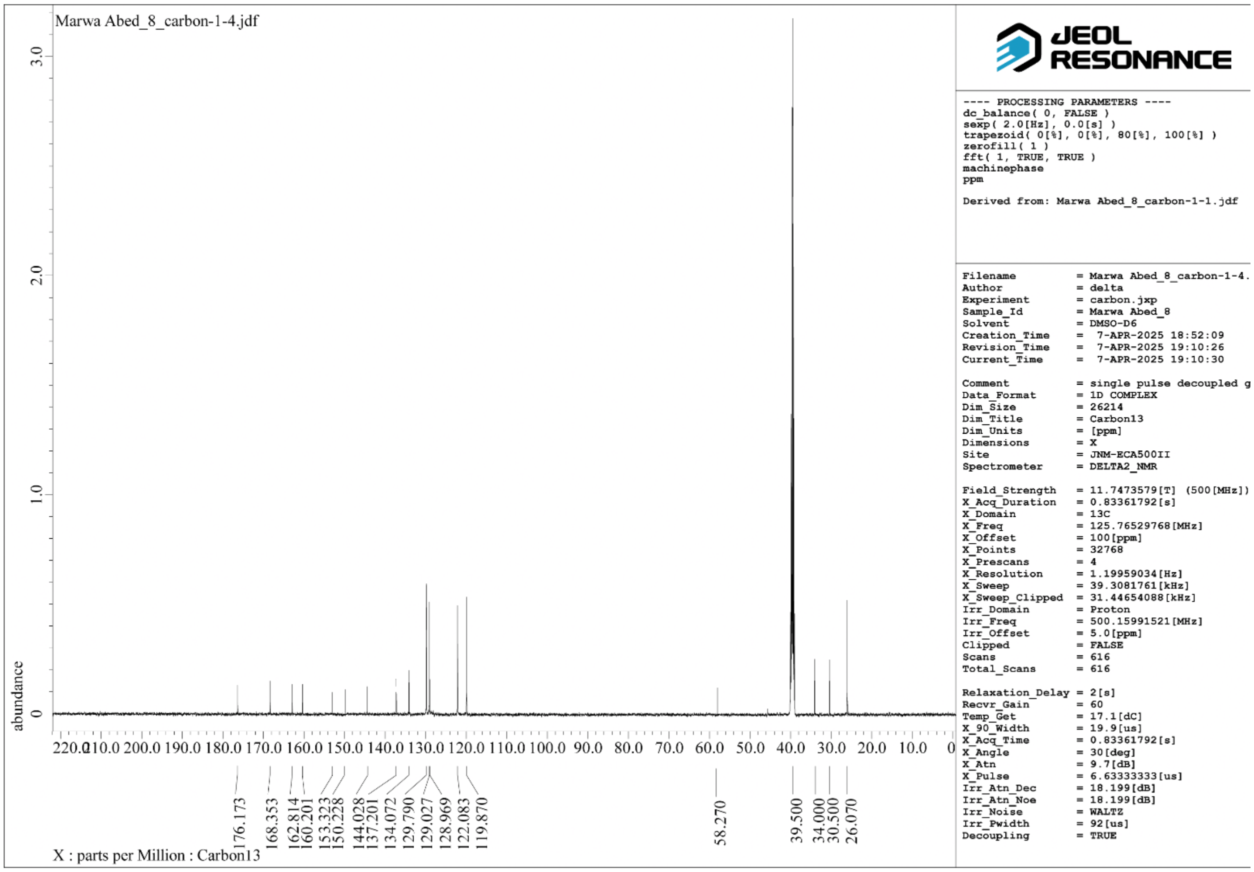

Figure S23. ^{13}C NMR spectrum (100 MHz, DMSO) of compound **7**

Characterization of Compound 8:

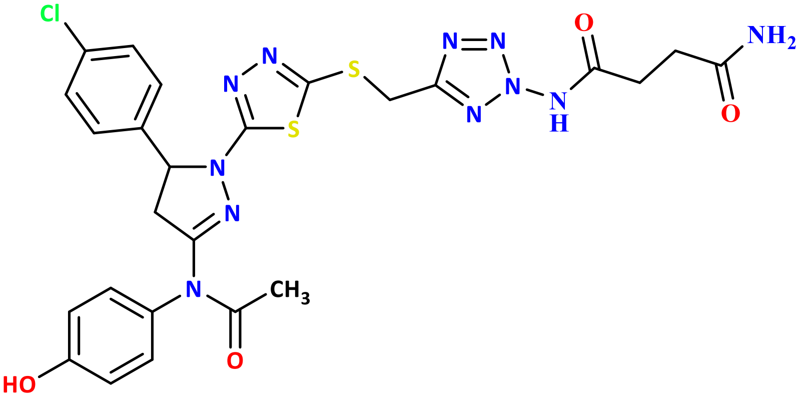

8

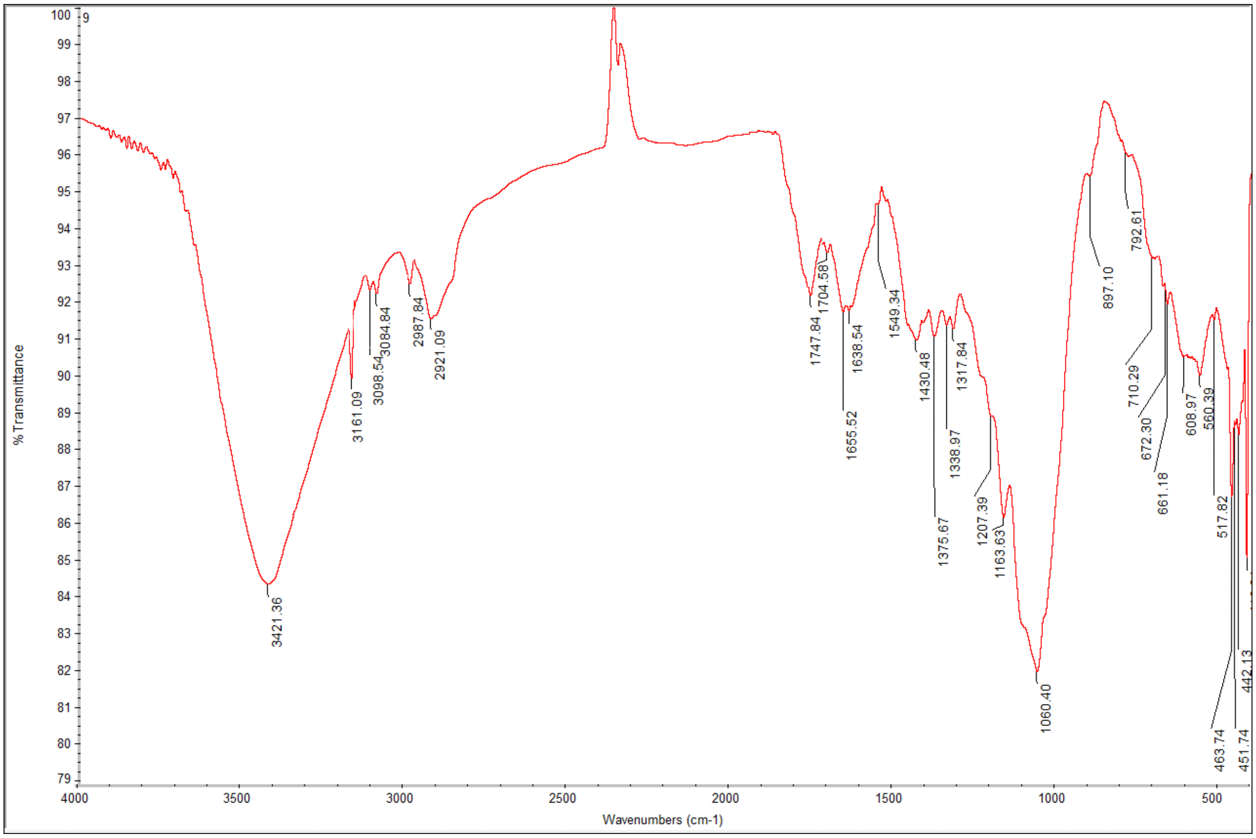

Figure S24. IR of Compound **8**

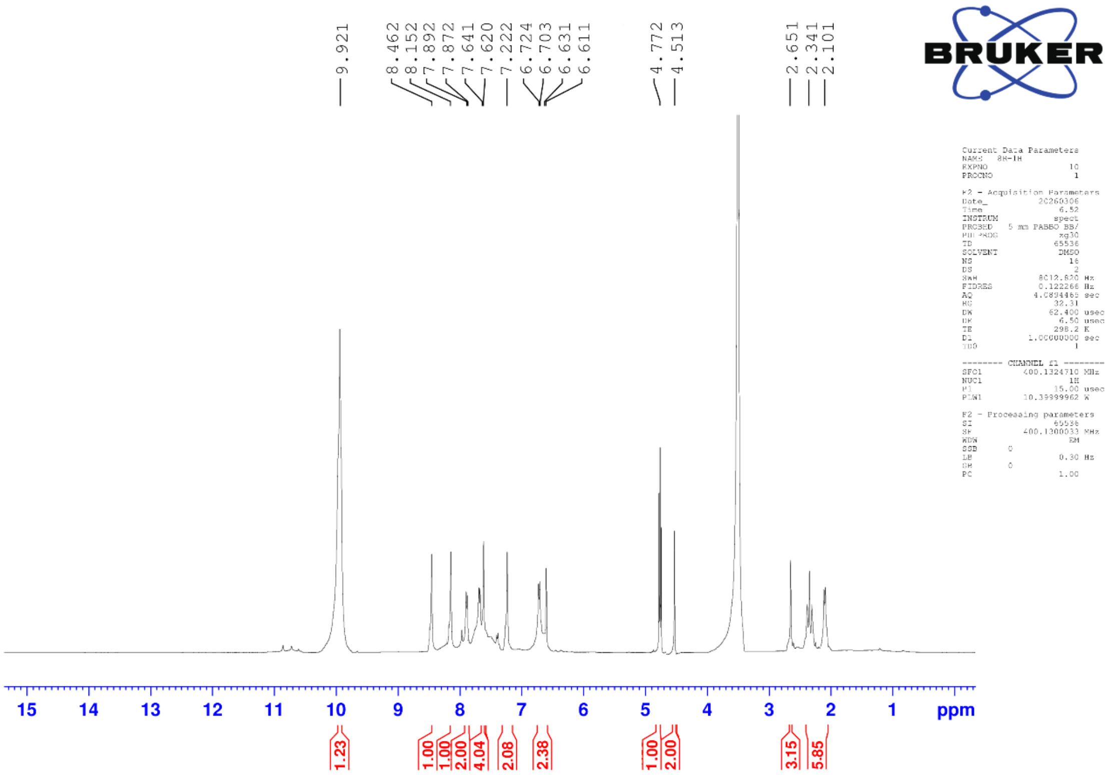

Figure S25. ^{1}H NMR spectrum (400 MHz, DMSO) of compound **8**

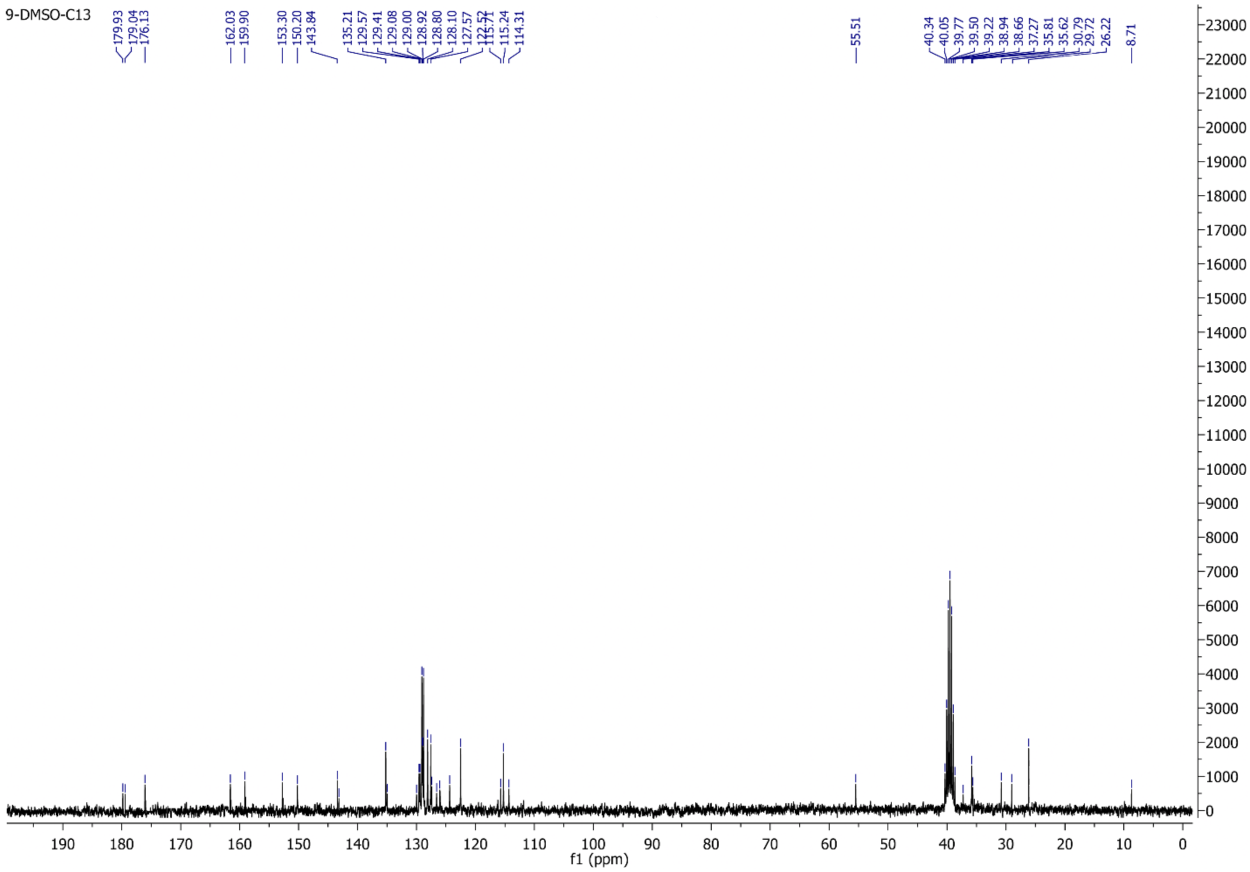

Figure S26. ^{13}C NMR spectrum (100 MHz, DMSO) of compound **8**

Characterization of Compound 9:

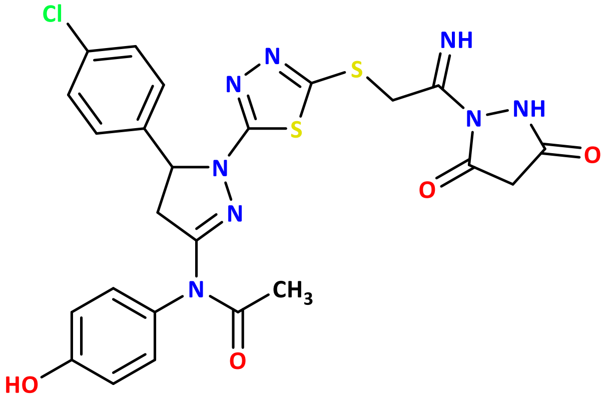

9

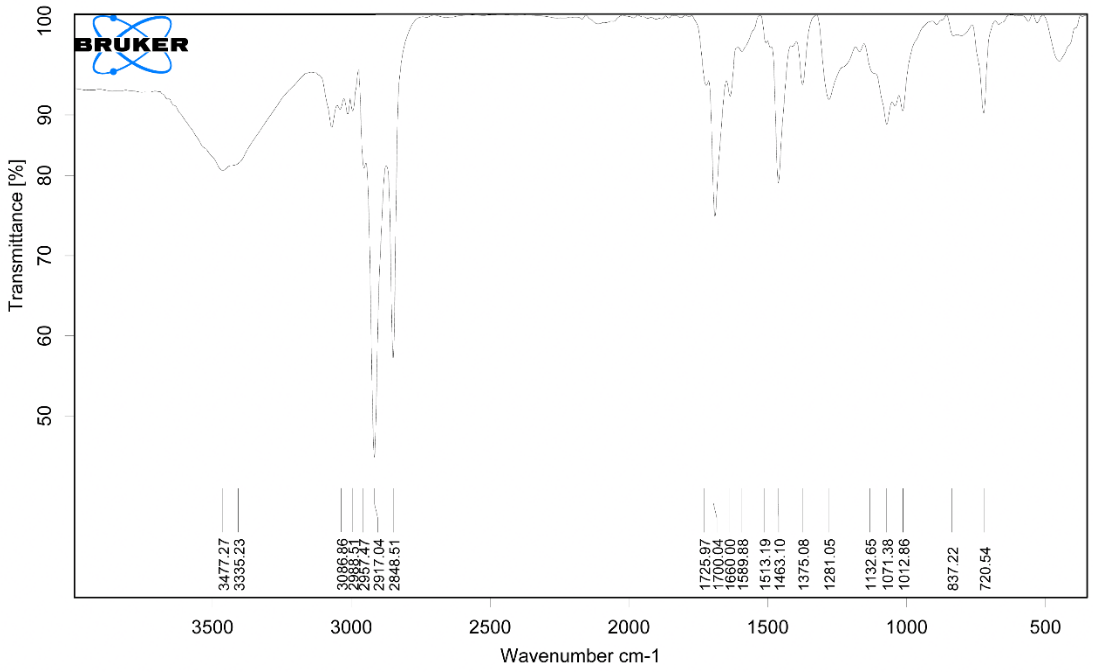

D:\Results\FT-IR- Auto save\Marwa- 10.0	Marwa- 10	9/12/2024

Page 1 of 1

Figure S27. IR of Compound **9**

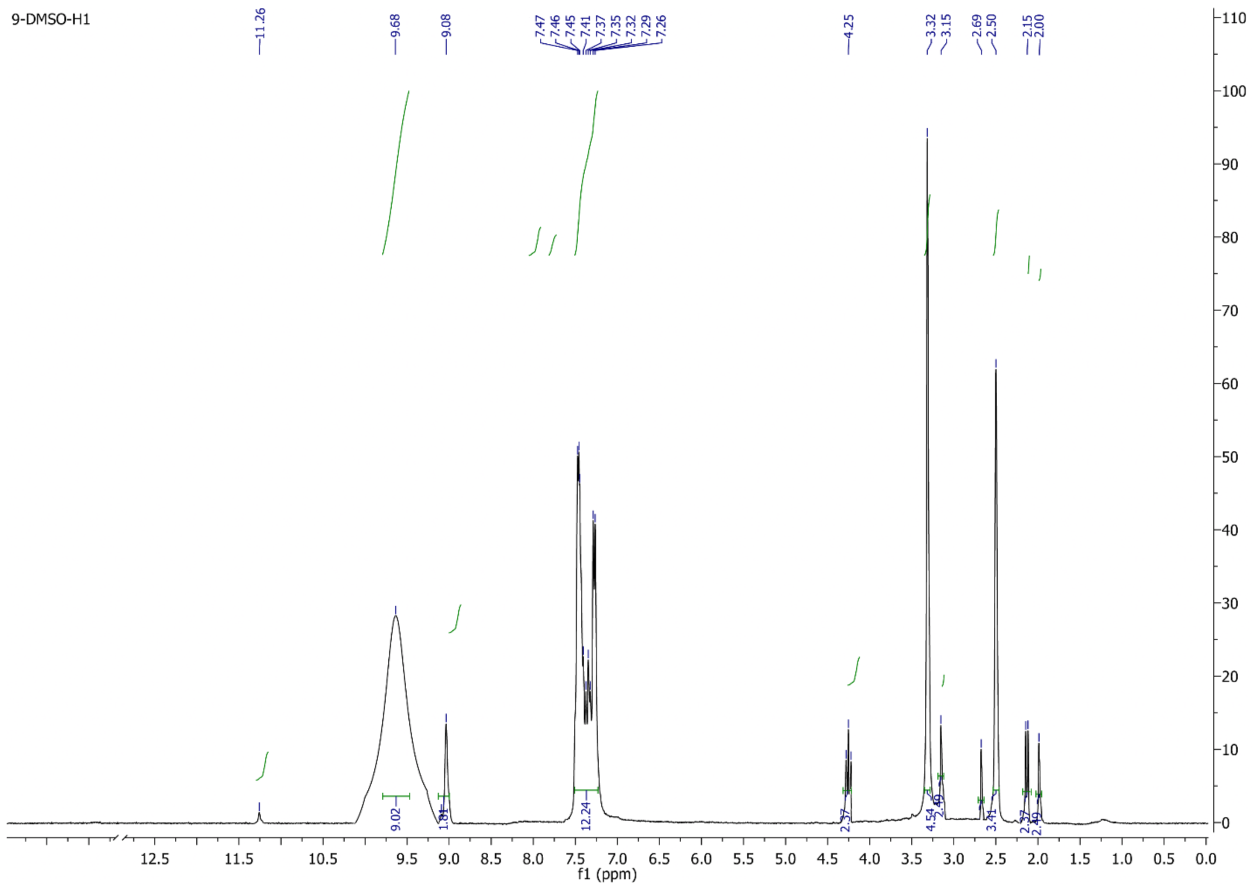

Figure S28. ^{1}H NMR spectrum (400 MHz, DMSO) of compound **9**

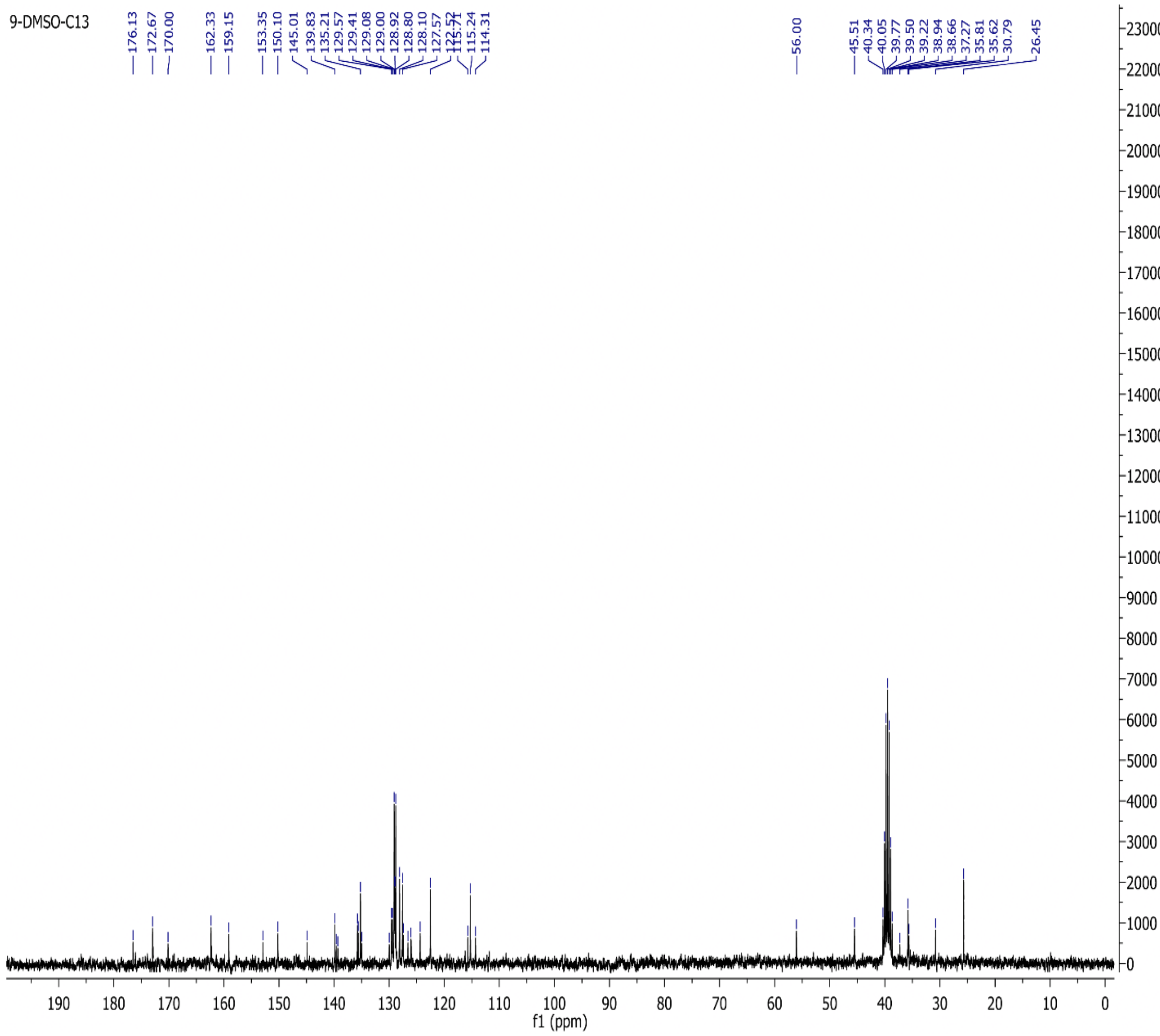

Figure S29. ^{13}C NMR spectrum (100 MHz, DMSO) of compound **9**

Characterization of Compound 10:

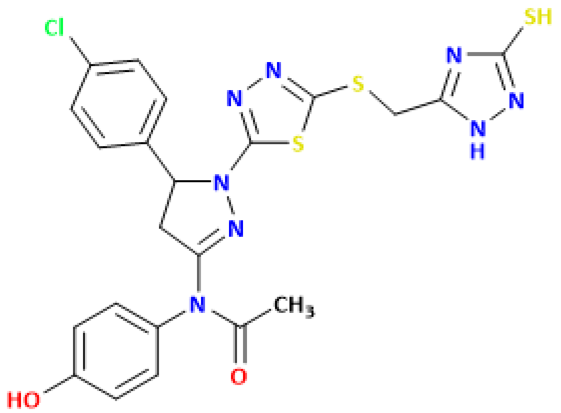

10

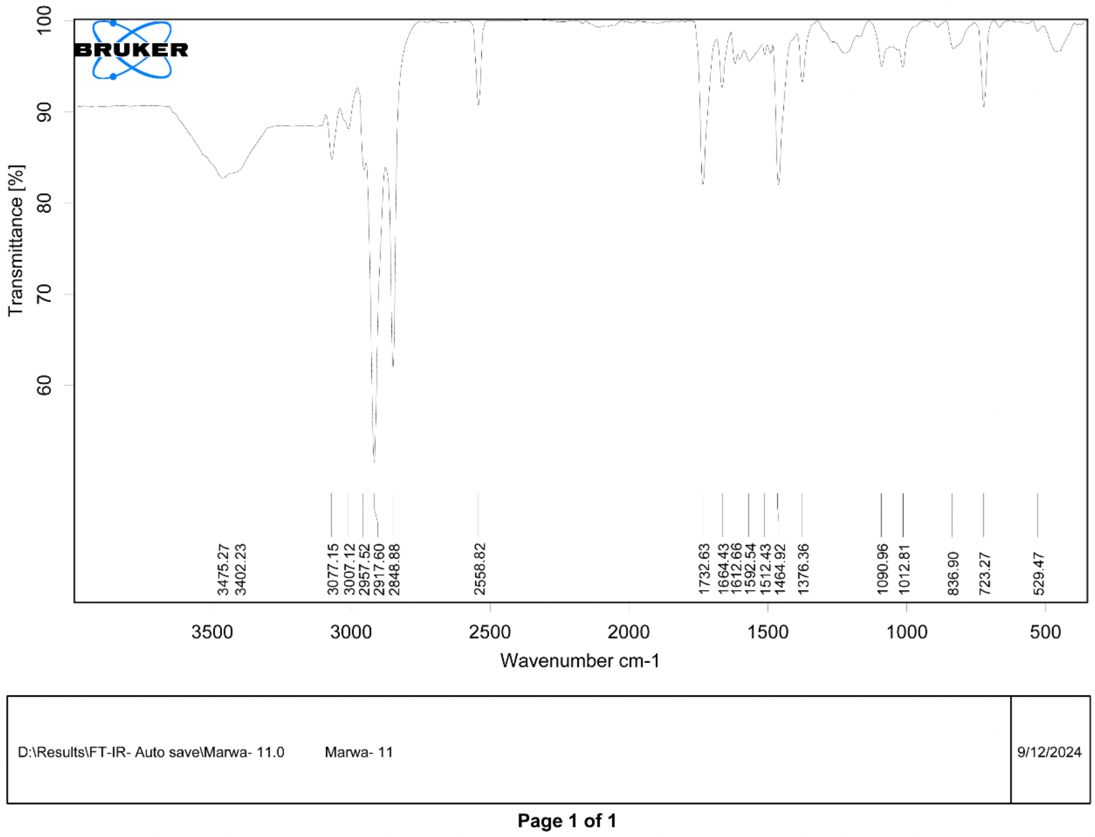

Figure S30. IR of Compound **10**

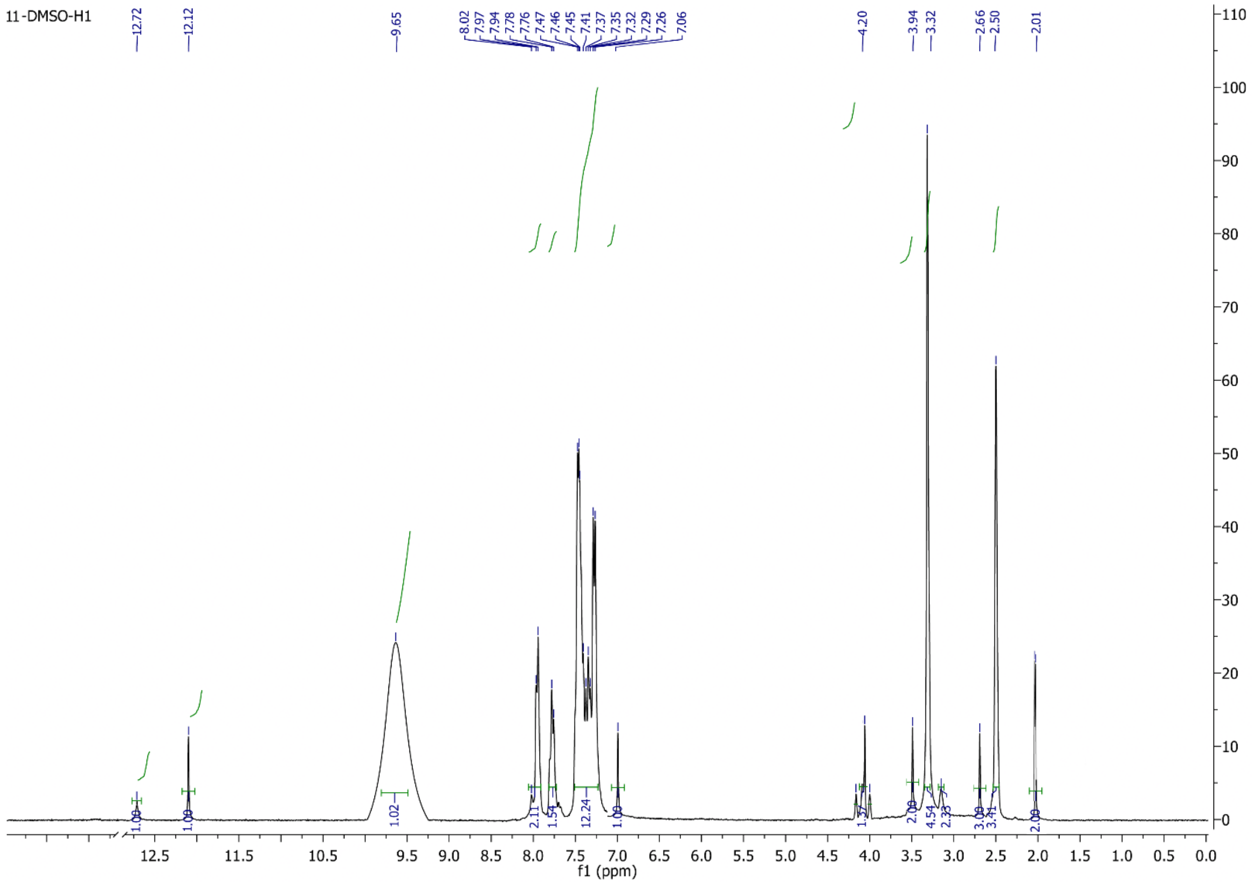

Figure S31. ^{1}H NMR spectrum (400 MHz, DMSO) of compound **10**

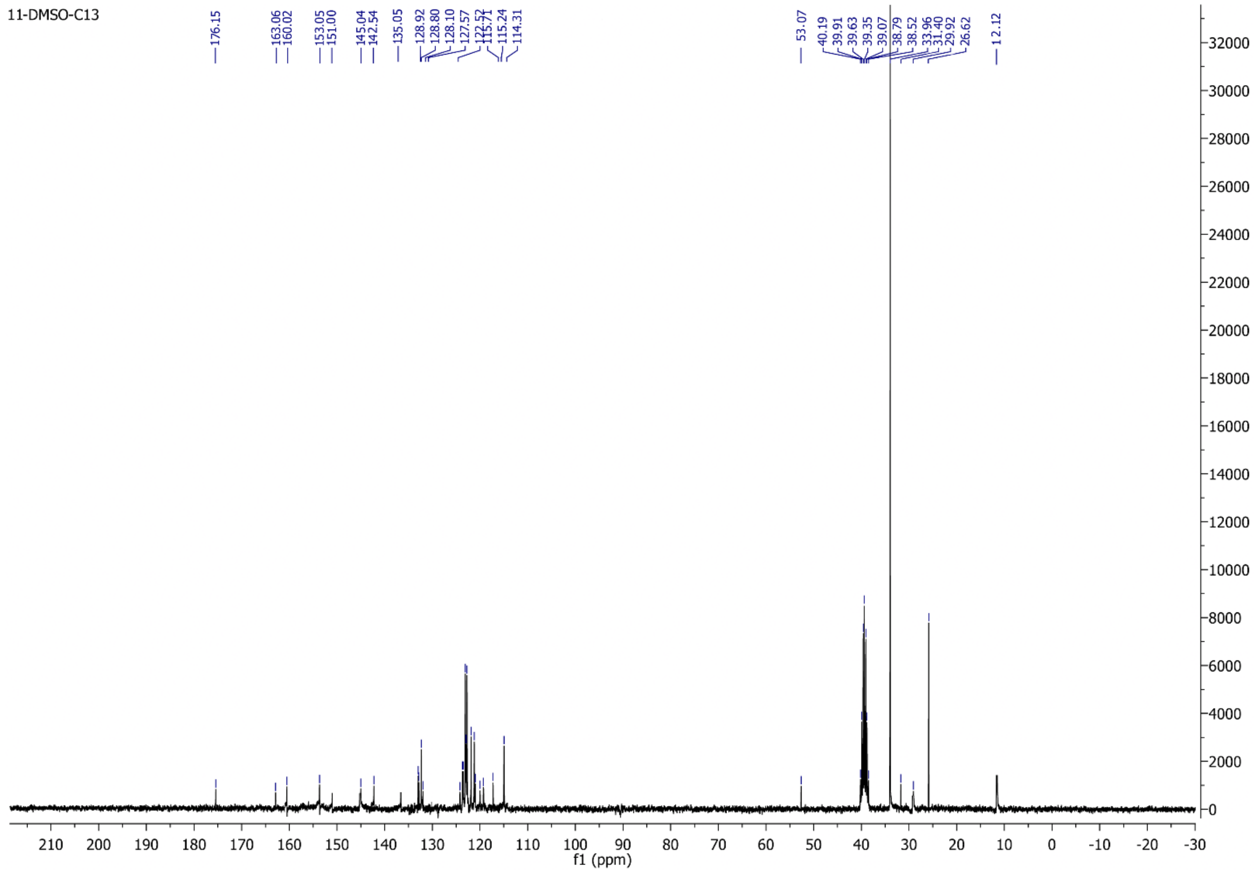

Figure S32. ^{1}H NMR spectrum (400 MHz, DMSO) of compound **10**

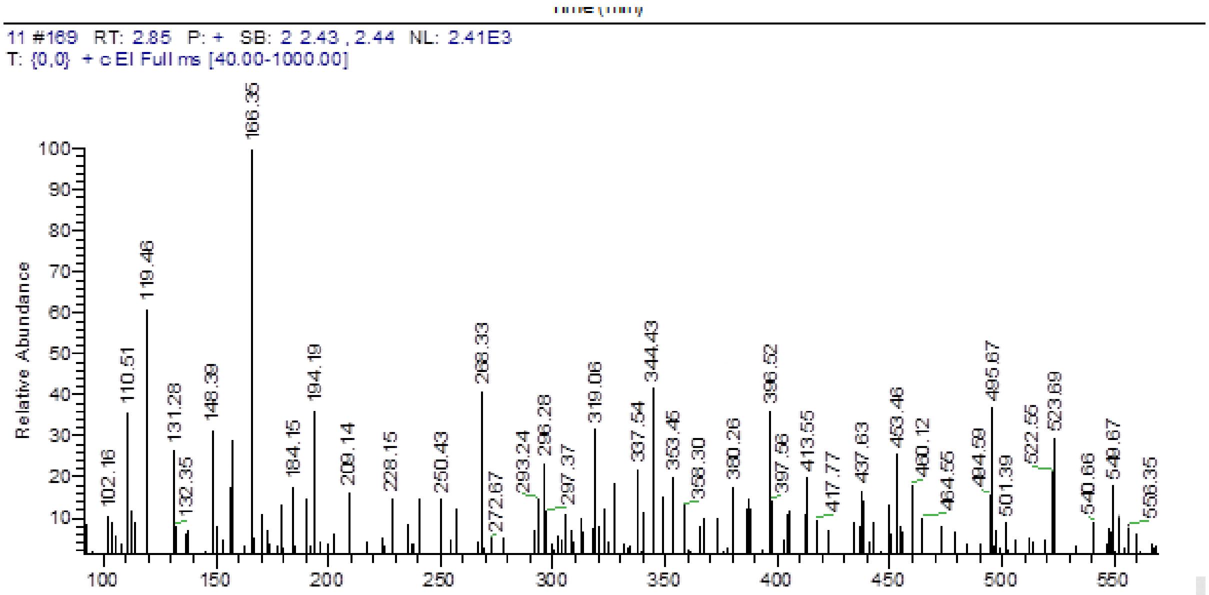

Figure S33. Mass spectrum of compound 10

Characterization of Compound 11:

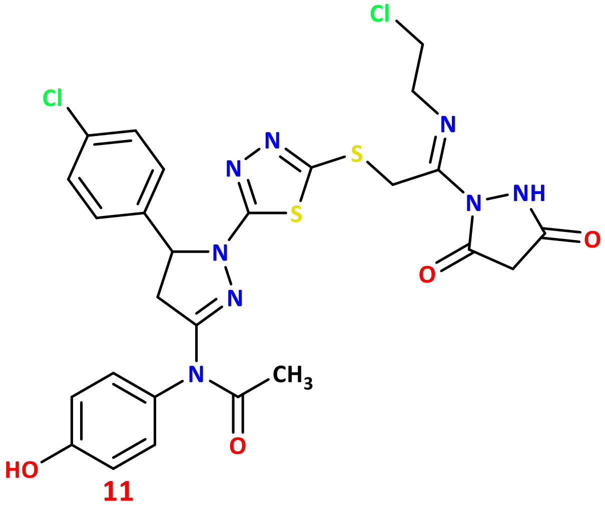

11

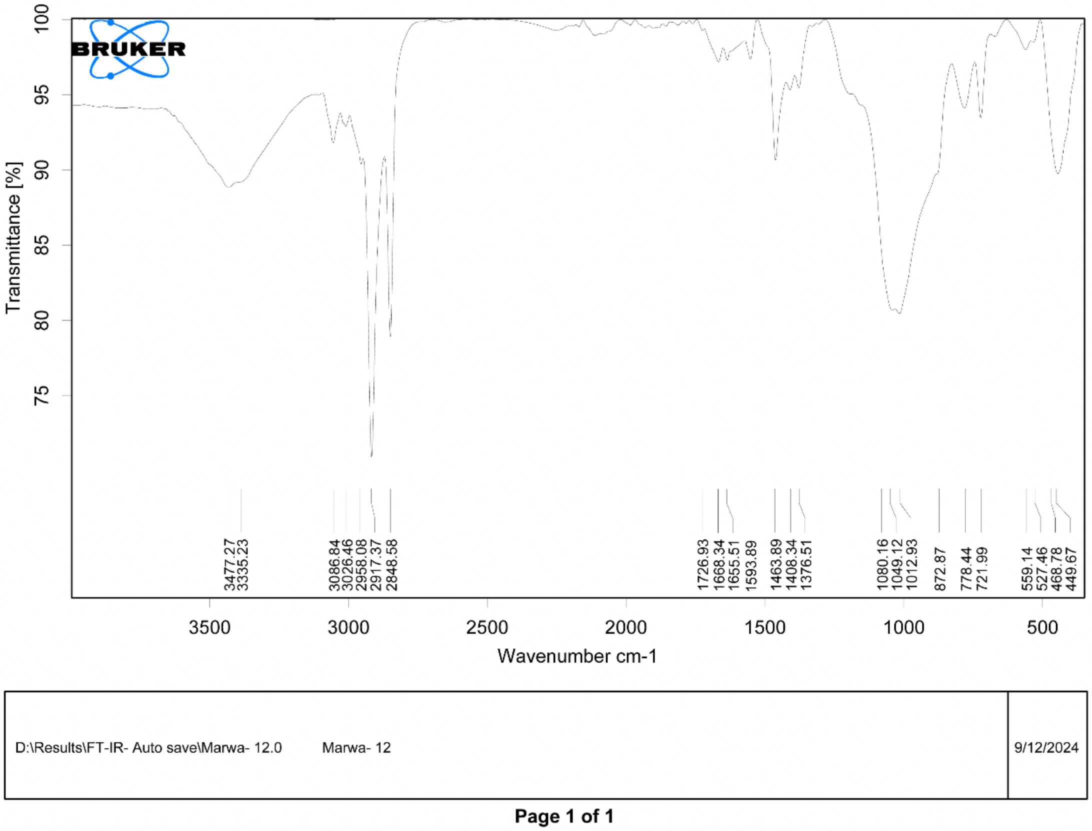

Figure S34. IR of Compound **11**

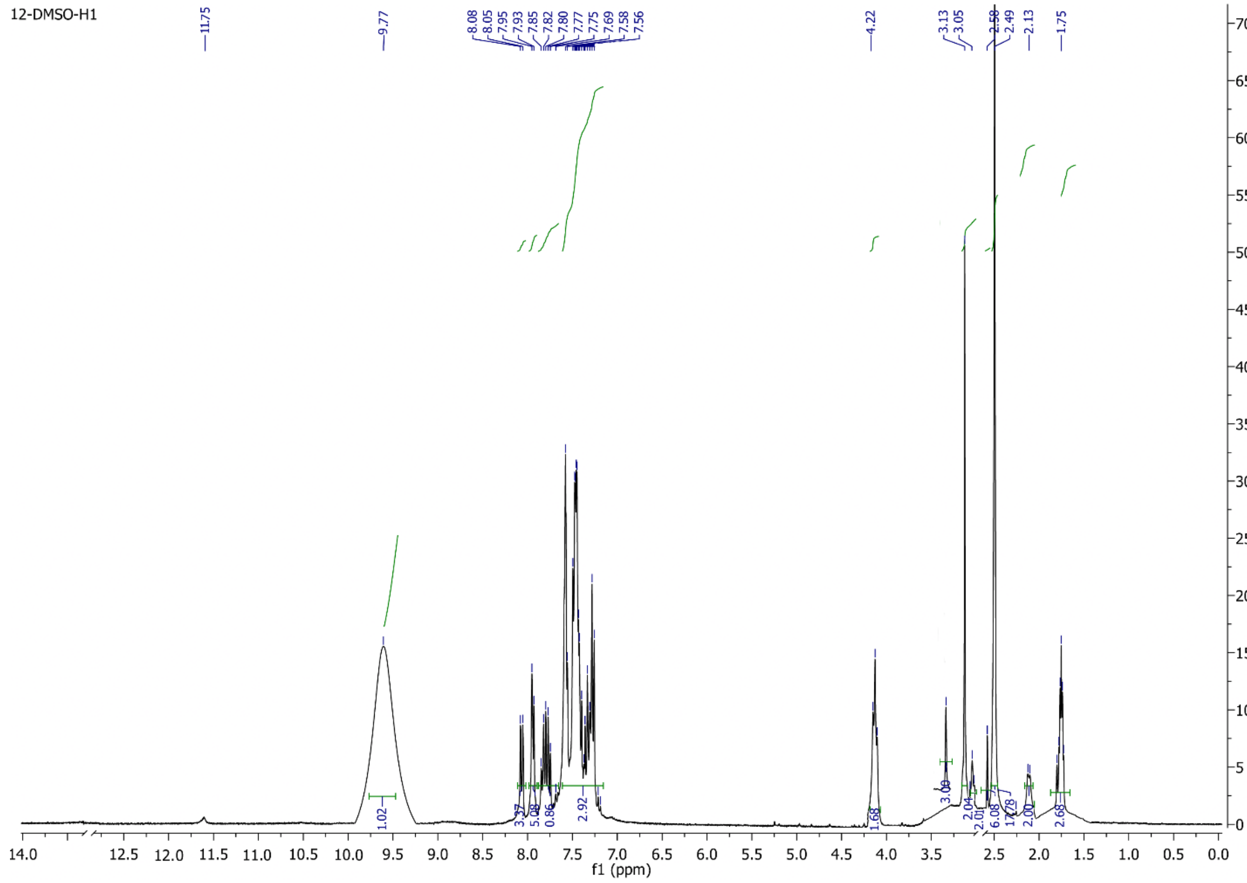

Figure S35. ^{1}H NMR spectrum (400 MHz, DMSO) of compound **11**

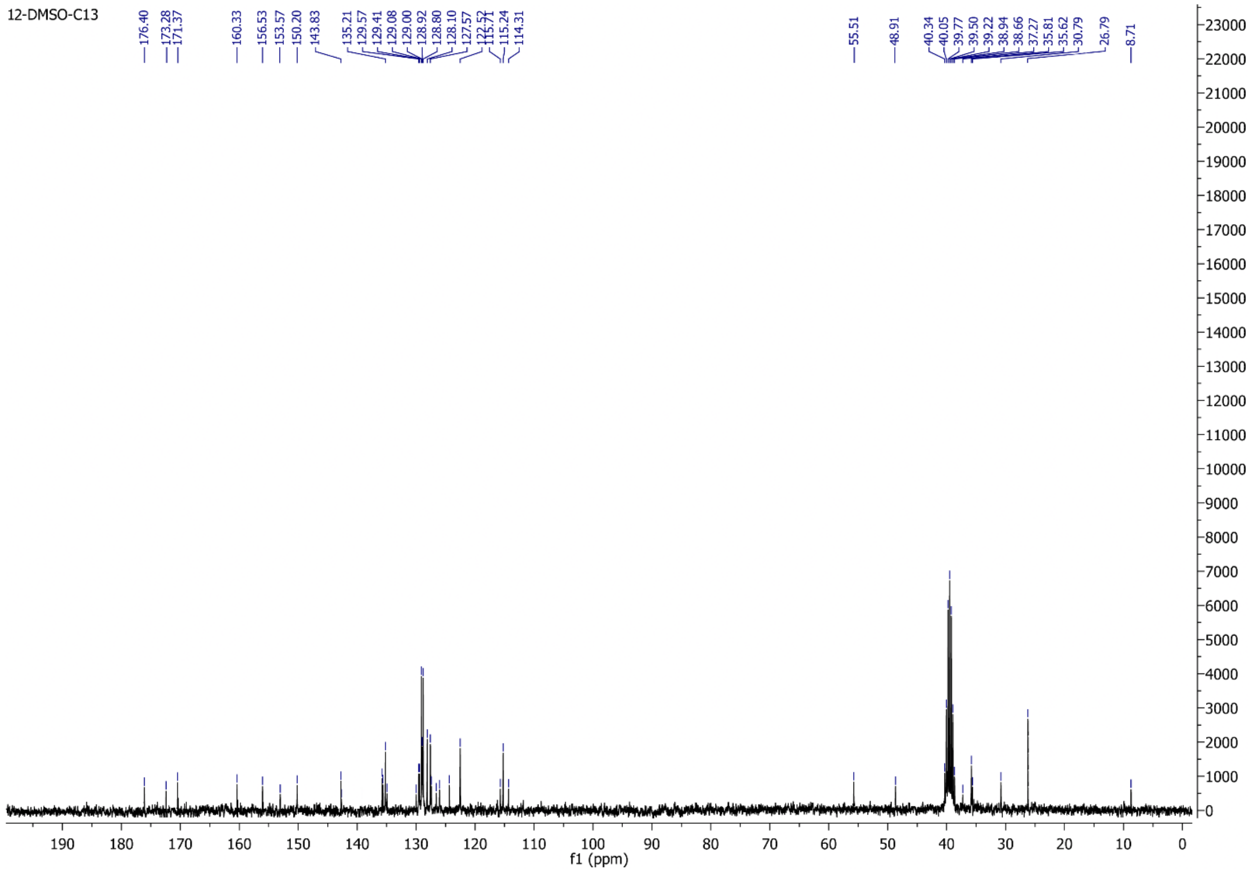

Figure S36. ^{13}C NMR spectrum (100 MHz, DMSO) of compound **11**

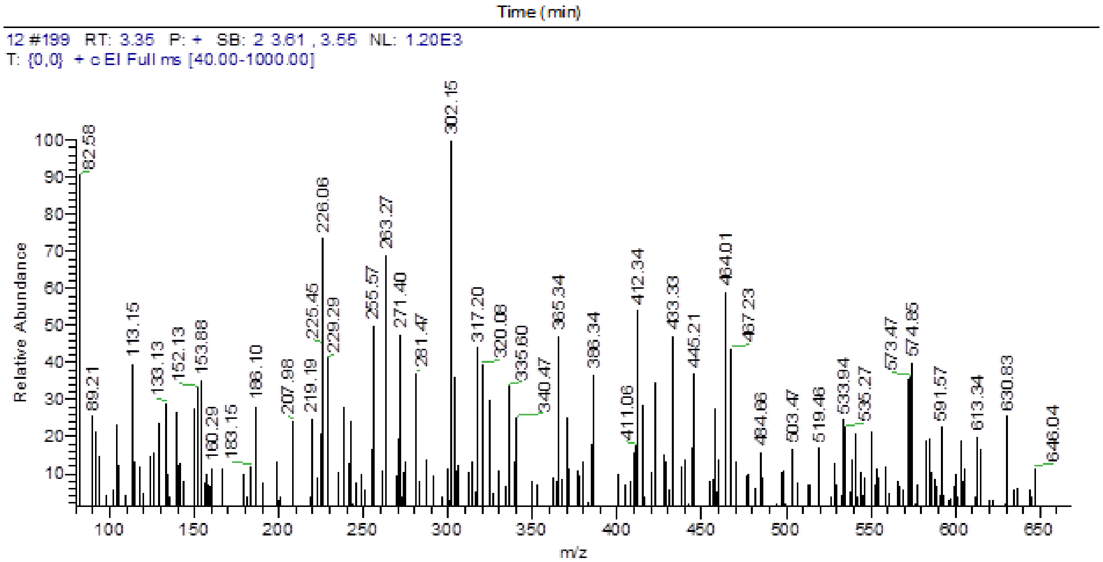

Figure S37. Mass spectrum of compound 11

Characterization of Compound 12:

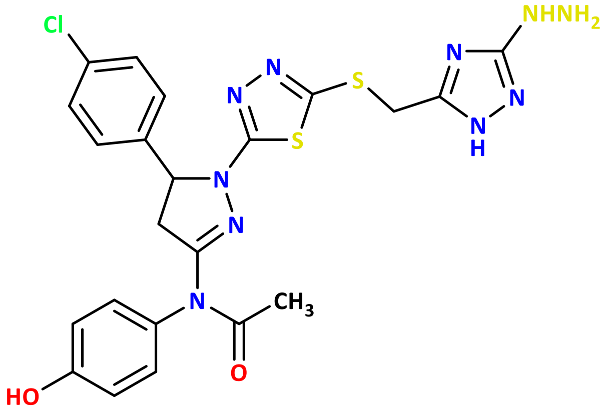

12

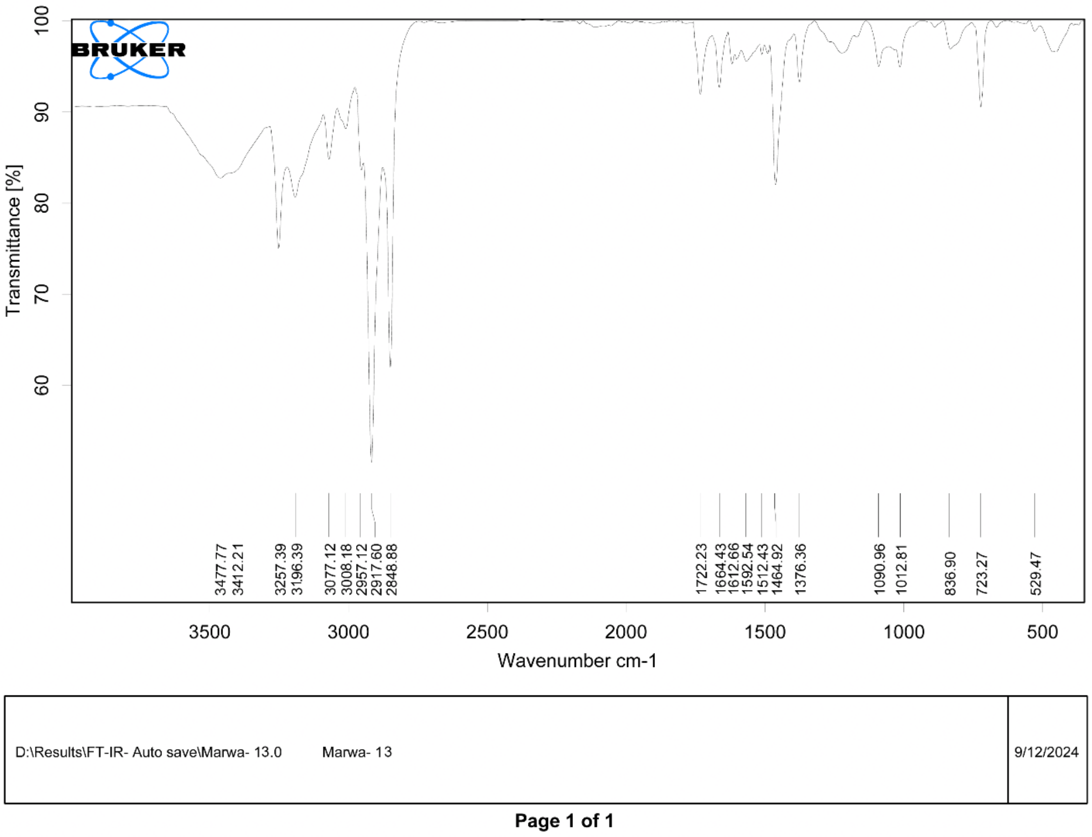

Figure S38. IR of Compound **12**

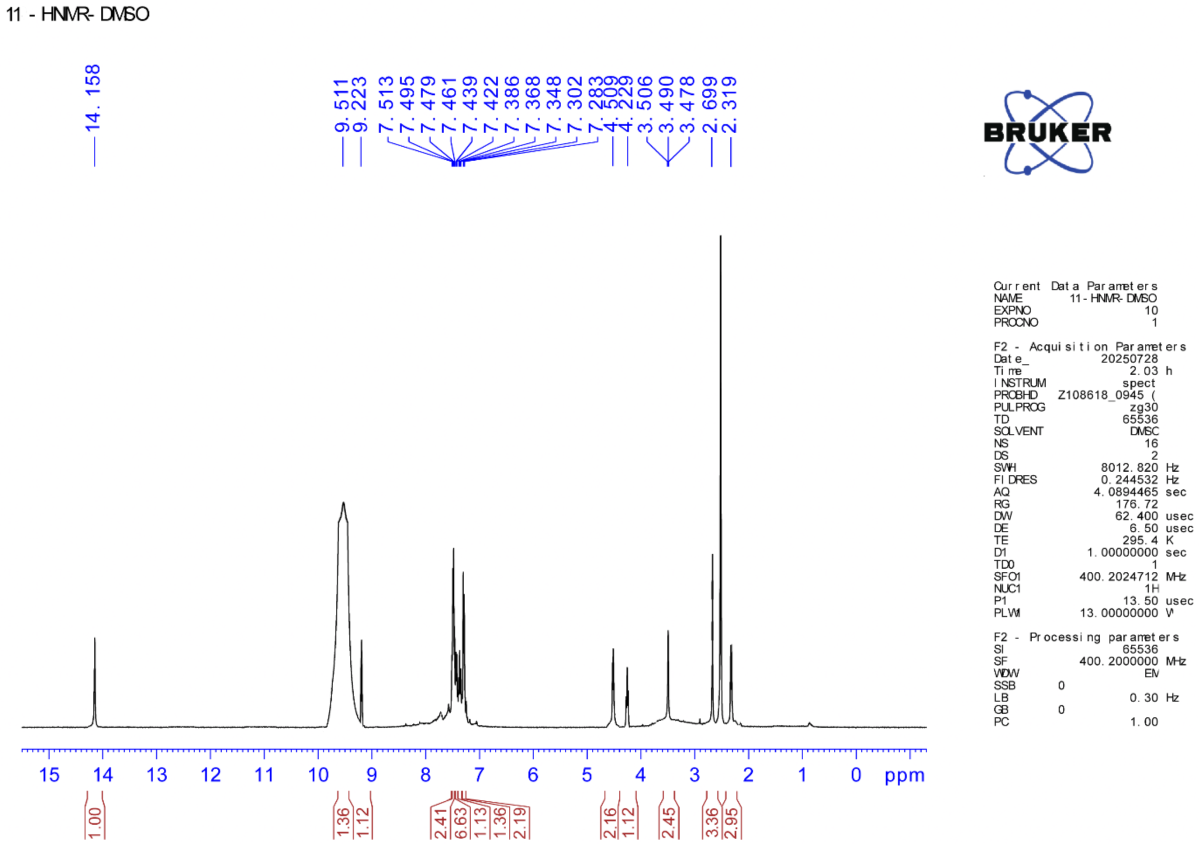

Figure S39 ^{1}H NMR spectrum (400 MHz, DMSO) of compound **12**

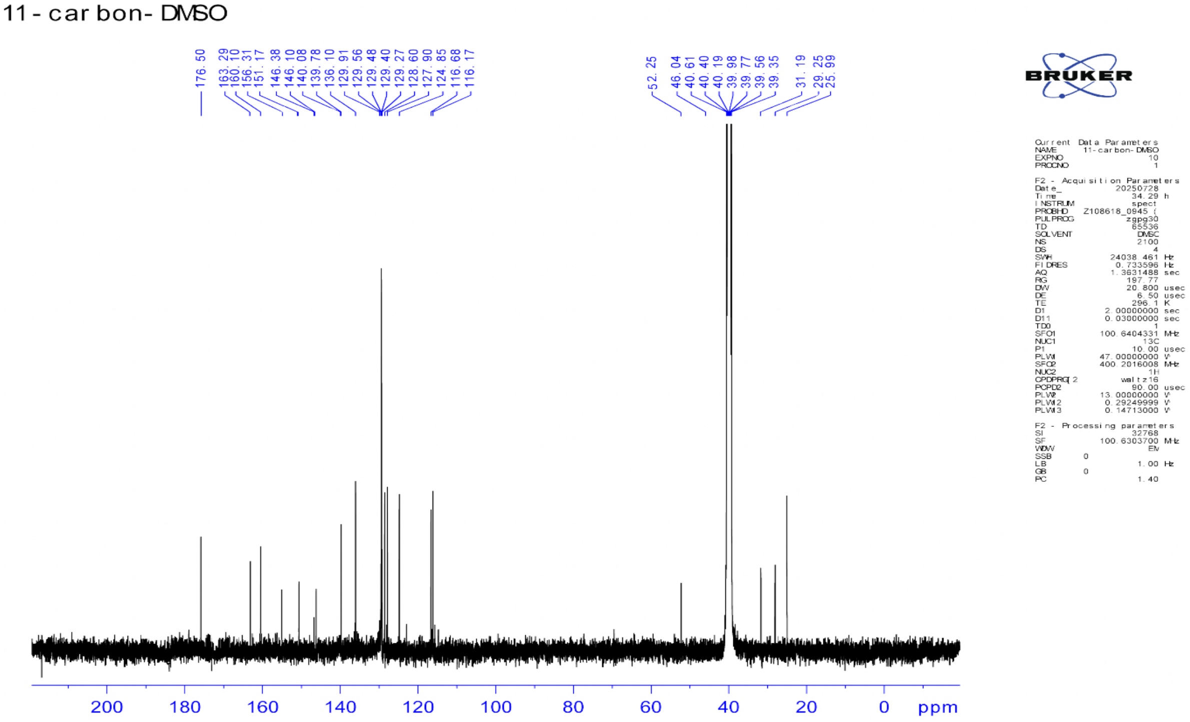

Figure S40. ^{13}C NMR spectrum (100 MHz, DMSO) of compound **11**

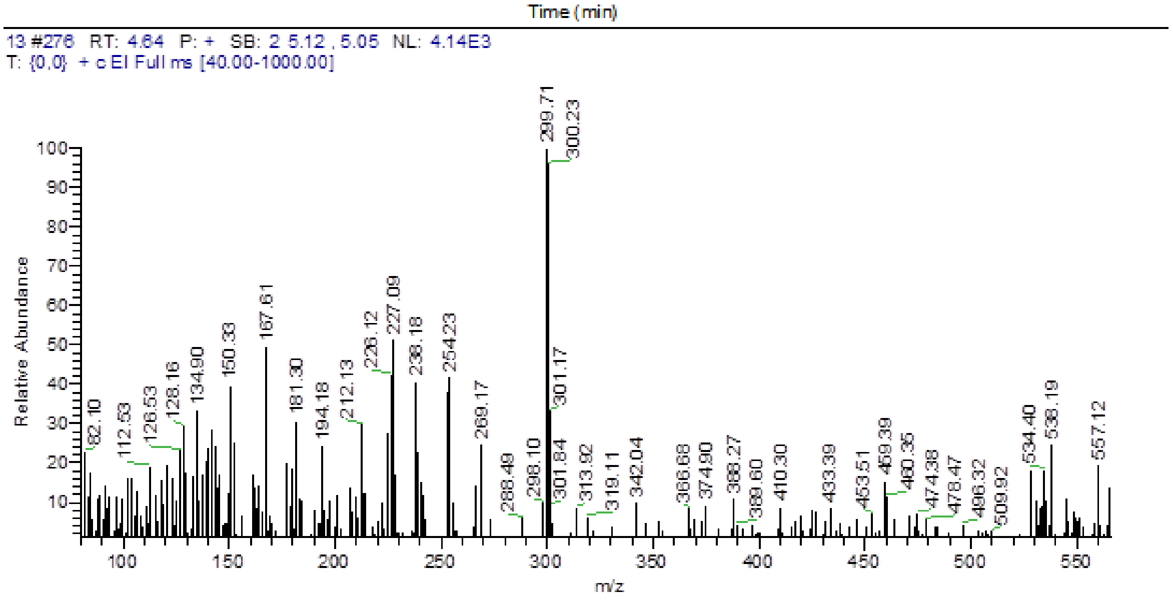

Figure S41. Mass spectrum of compound 12

Characterization of Compound 13:

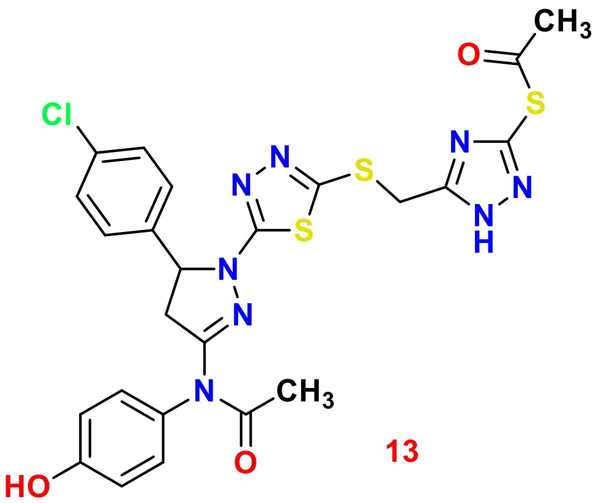

13

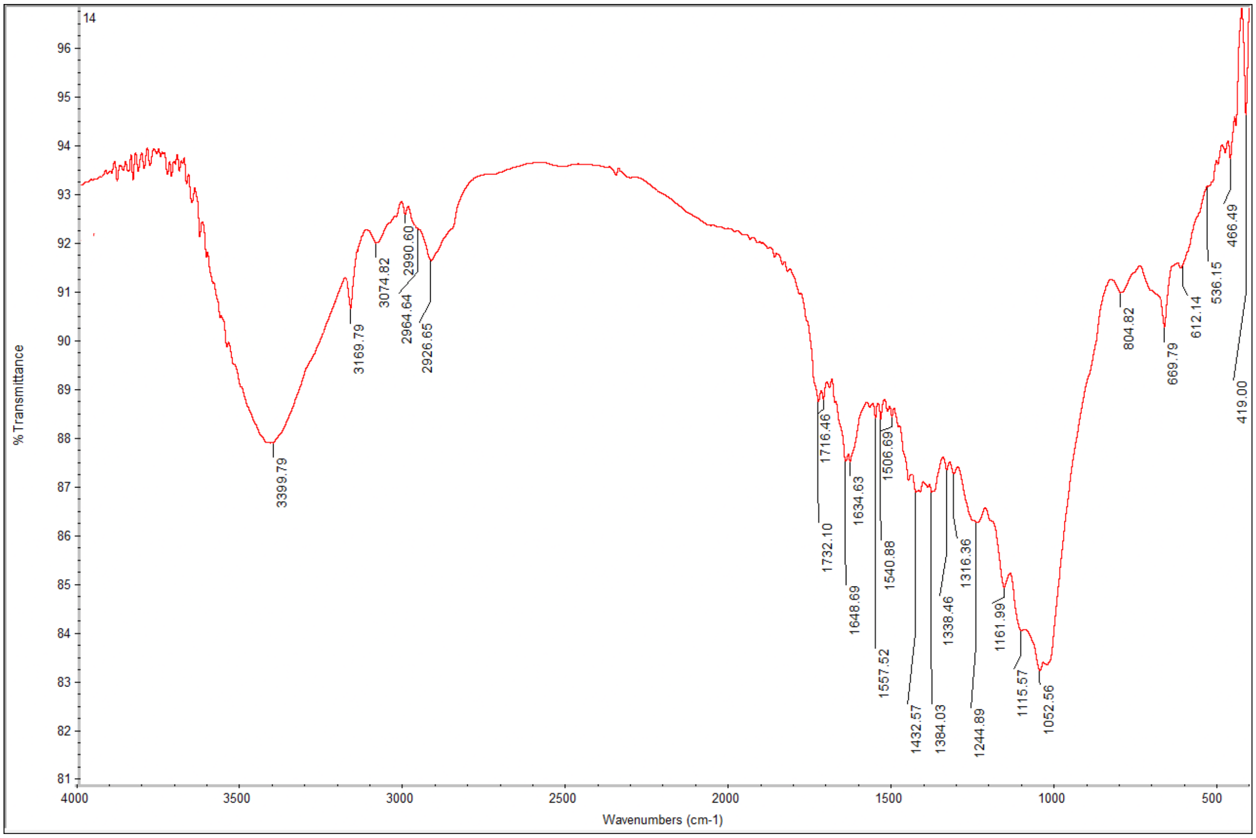

Figure S42. IR of Compound **13**

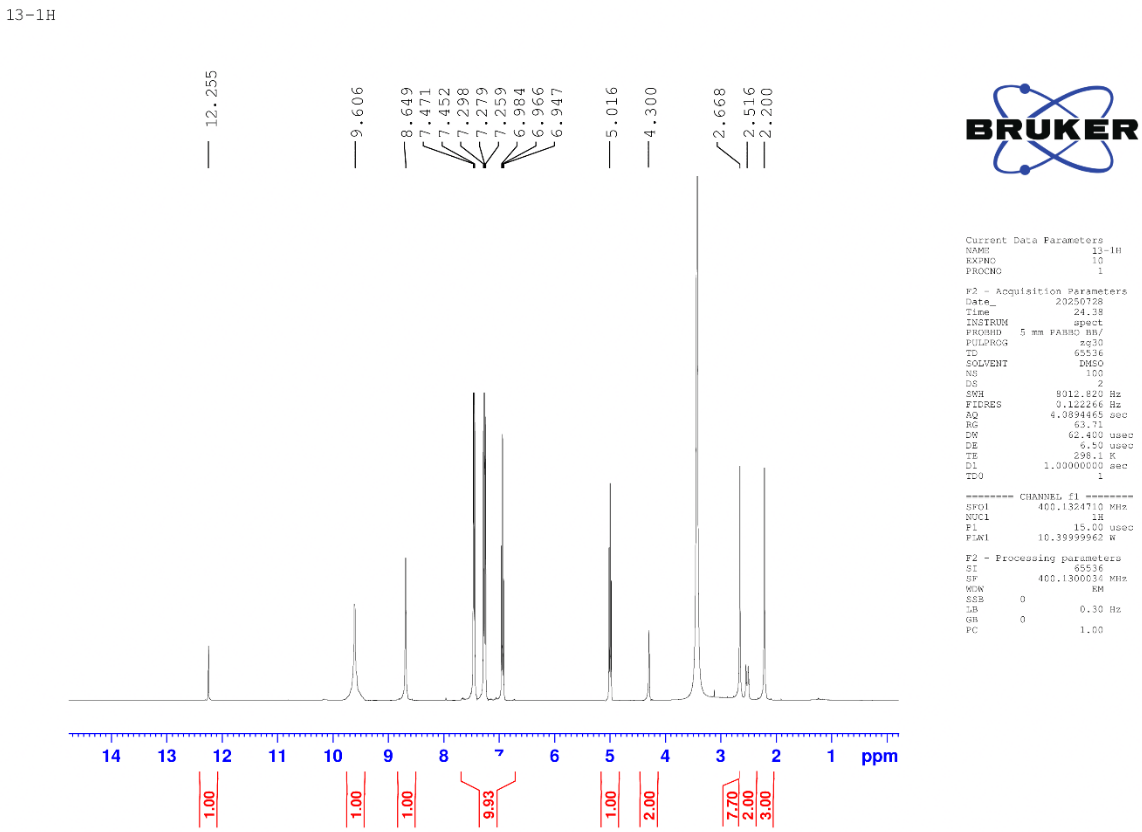

Figure S43. ^{1}H NMR spectrum (400 MHz, DMSO) of compound **13**

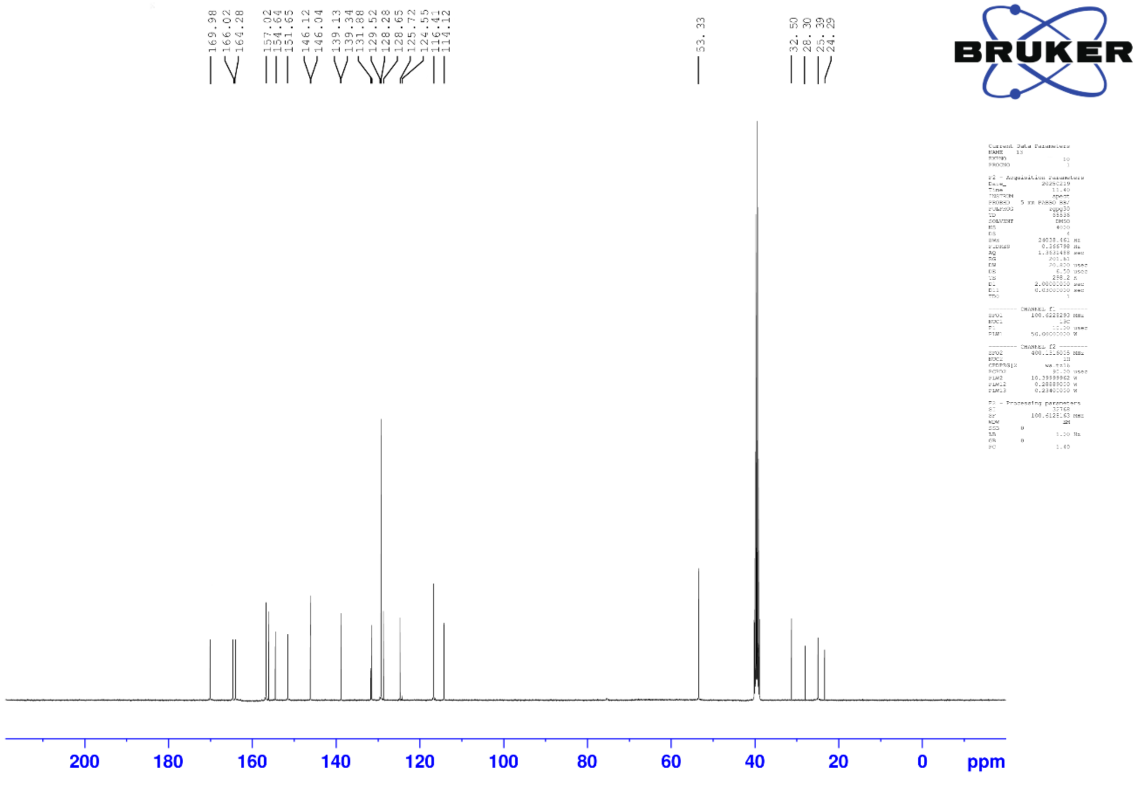

Figure S44. ^{13}C NMR spectrum (100 MHz, DMSO) of compound **13**

Characterization of Compound 14:

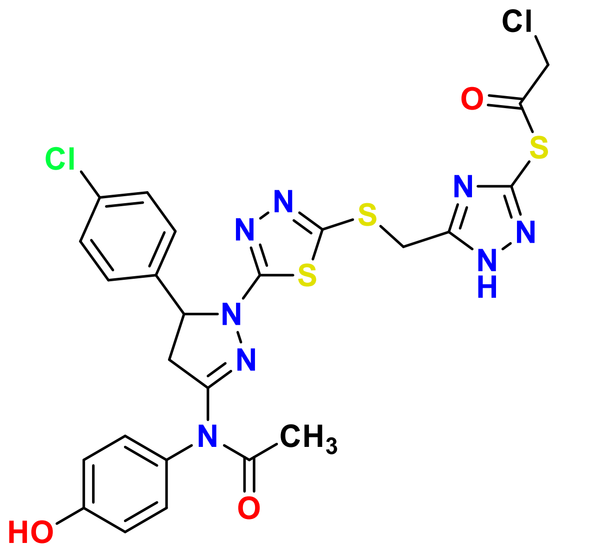

14

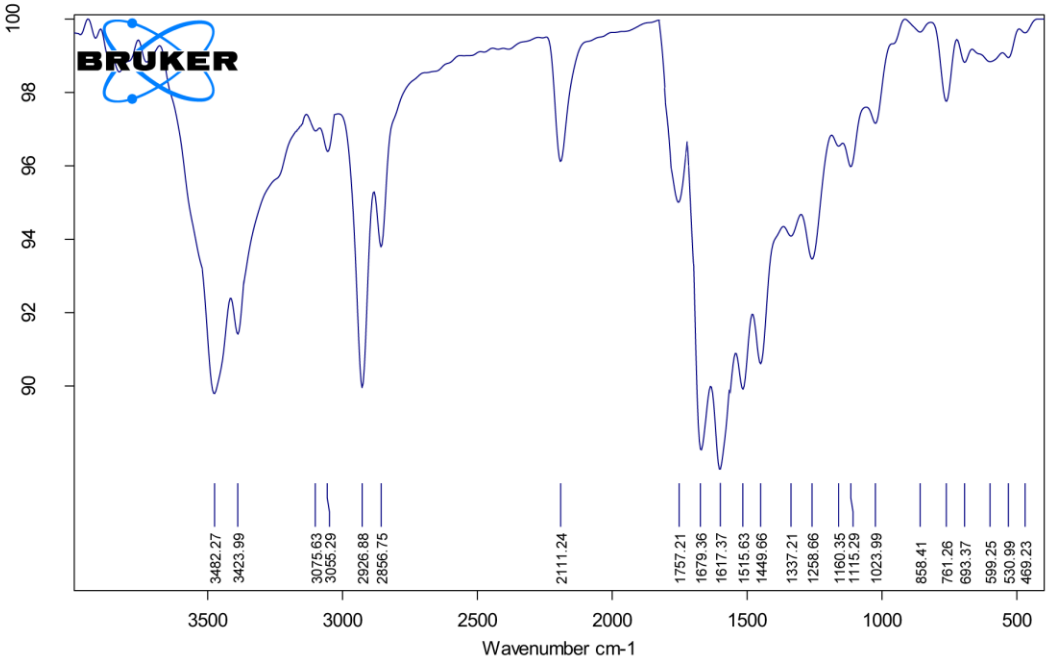

E:\FT_IR Measurments\TR\1	1	Instrument type and / or accessory	9/29/2025

Page 1/1

Figure S45. IR of Compound **14**

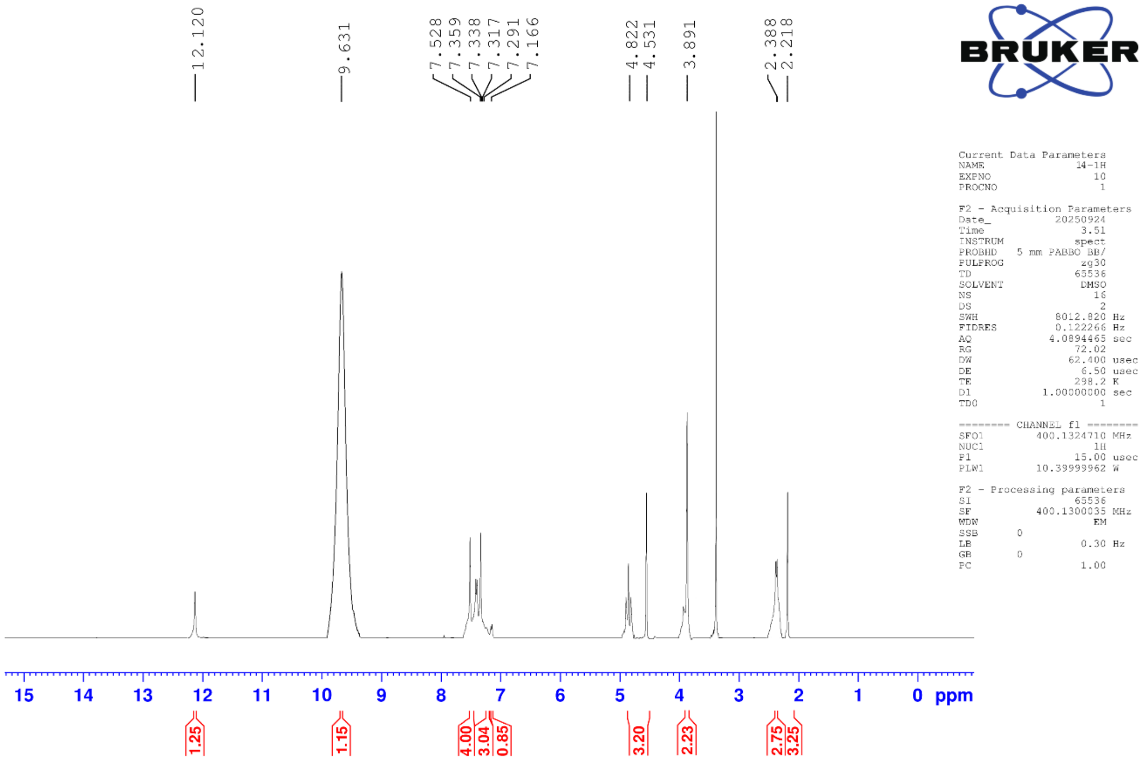

Figure S46. ^{1}H NMR spectrum (400 MHz, DMSO) of compound **14**

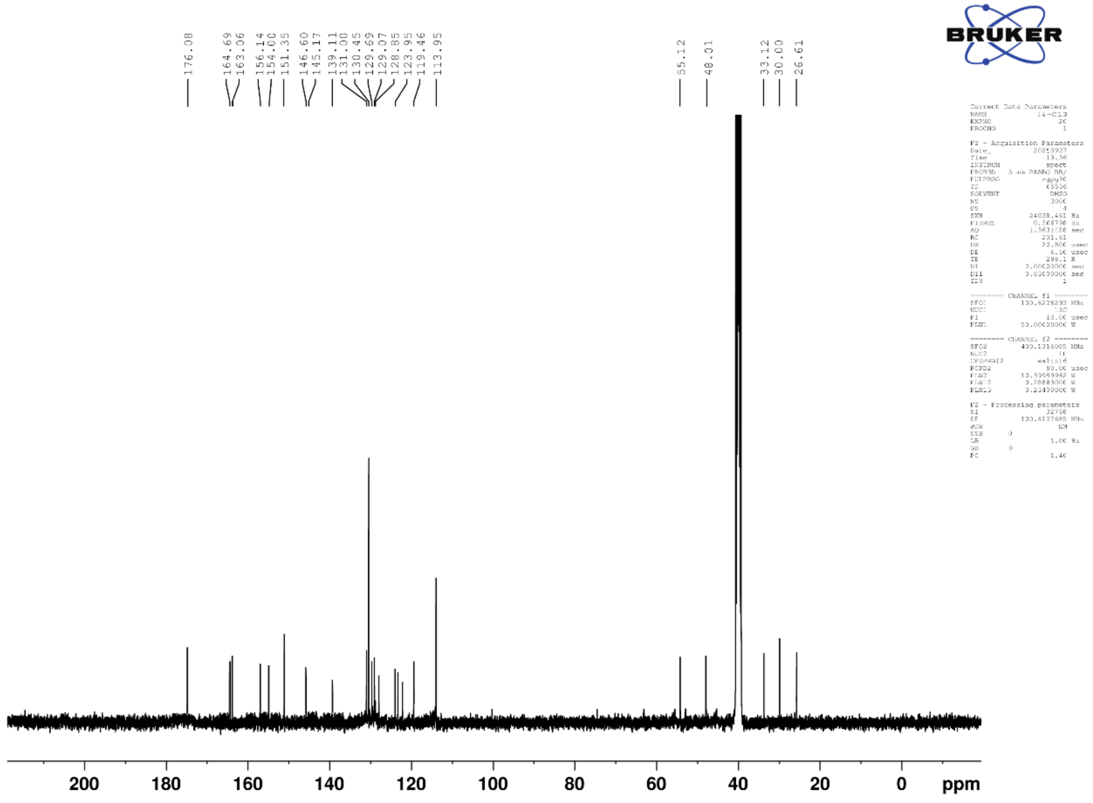

Figure S47. ^{1}H NMR spectrum (400 MHz, DMSO) of compound **14**

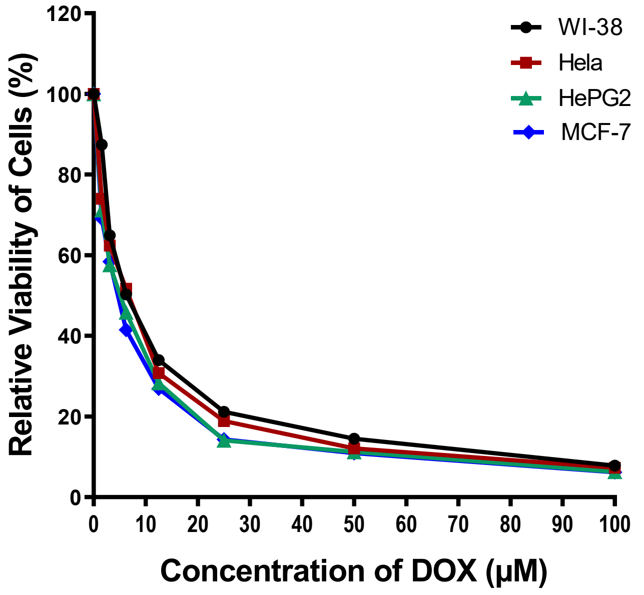

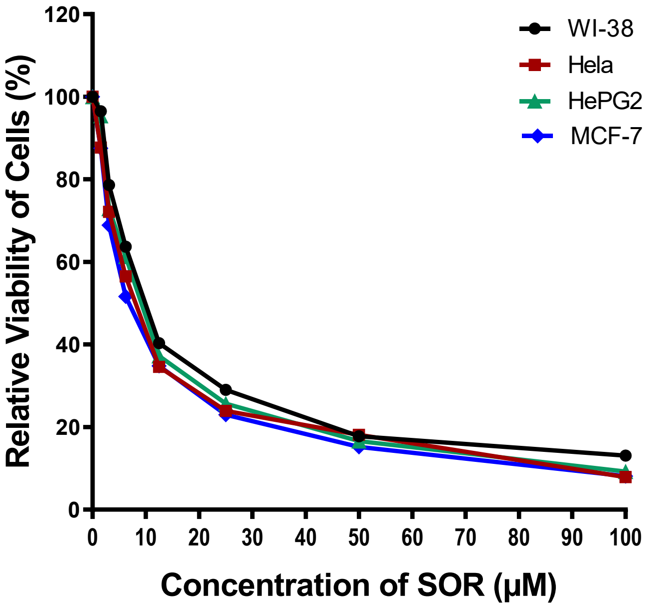

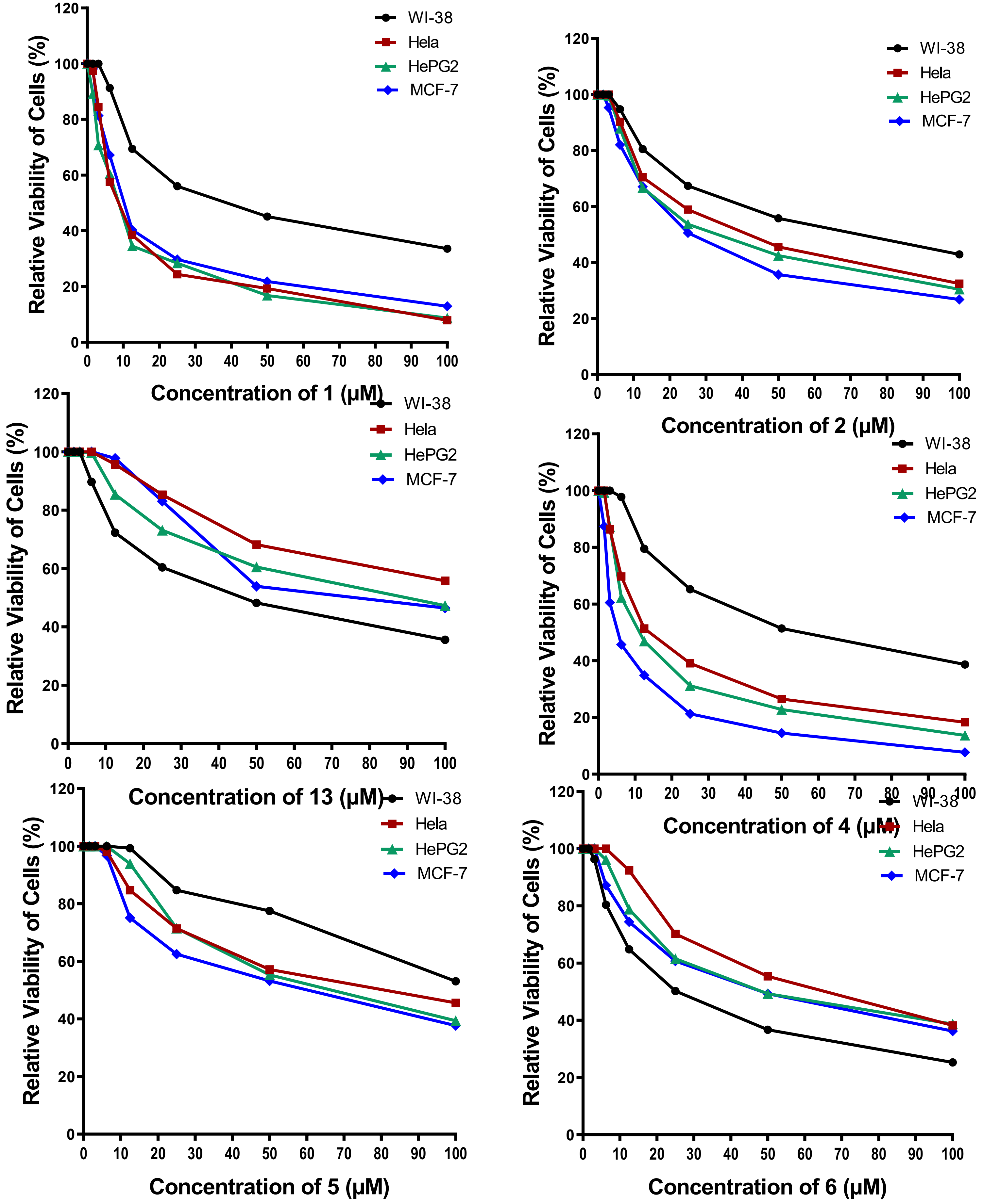

WI-38
Hela
HePG2
MCF-7
Relative Viability of Cells (%)
Concentration of 1 (μM)
Concentration of 2 (μM)
Concentration of 13 (μM)
Concentration of 4 (μM)
Concentration of 5 (μM)
Concentration of 6 (μM)

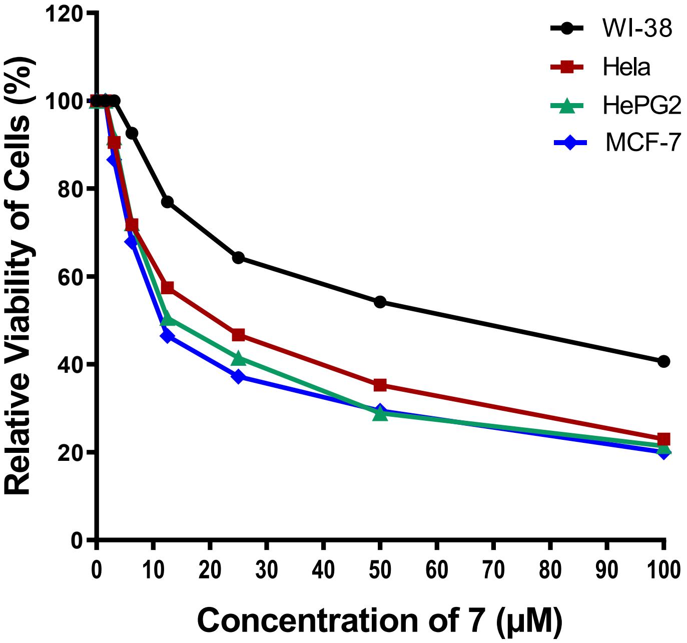
Relative Viability of Cells (%)
Concentration of 7 (μM)
WI-38
Hela
HePG2
MCF-7

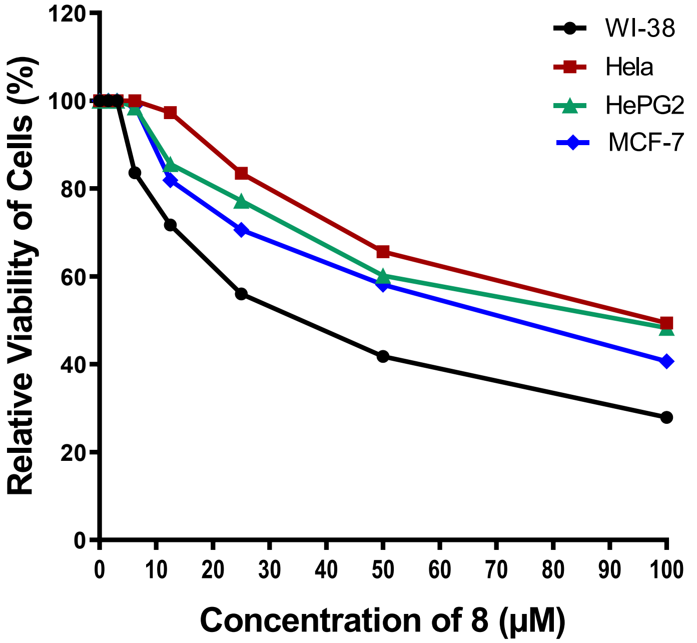
Relative Viability of Cells (%)
Concentration of 8 (μM)
WI-38
Hela
HePG2
MCF-7

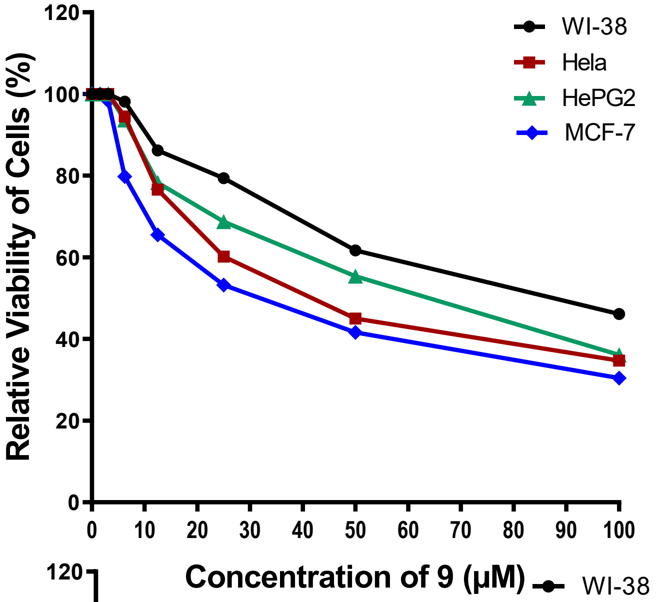
Relative Viability of Cells (%)
Concentration of 9 (μM)
WI-38
Hela
HePG2
MCF-7

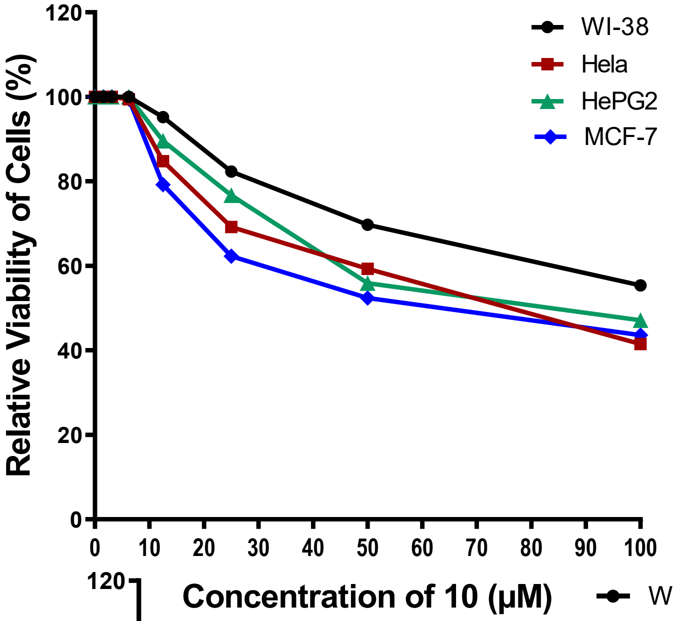
Relative Viability of Cells (%)
Concentration of 10 (μM)
WI-38
Hela
HePG2
MCF-7

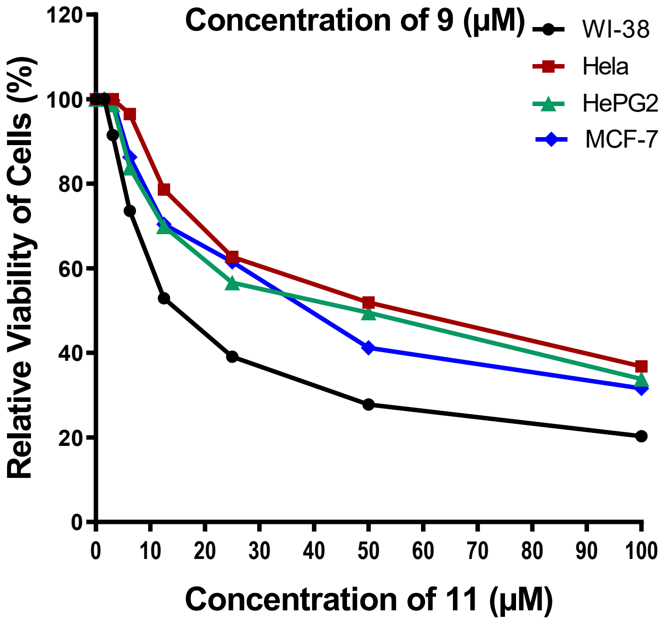
Relative Viability of Cells (%)
Concentration of 11 (μM)
WI-38
Hela
HePG2
MCF-7

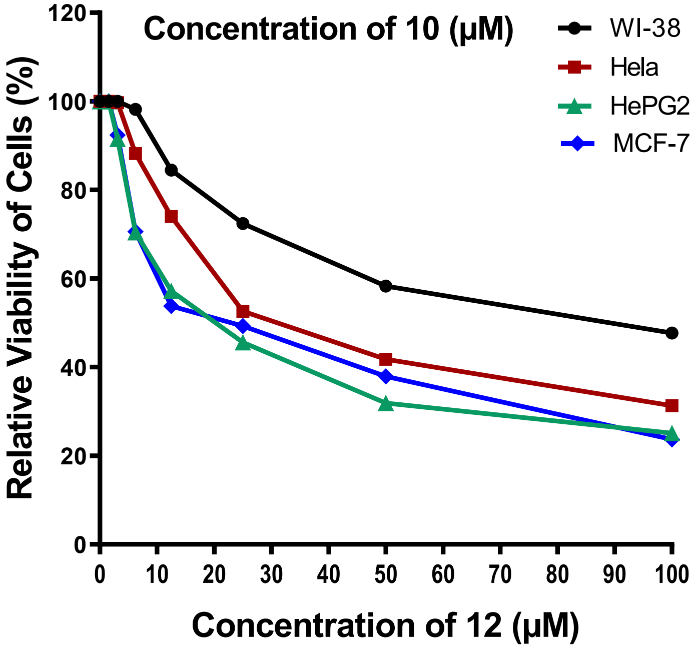
Relative Viability of Cells (%)
Concentration of 12 (μM)
WI-38
Hela
HePG2
MCF-7

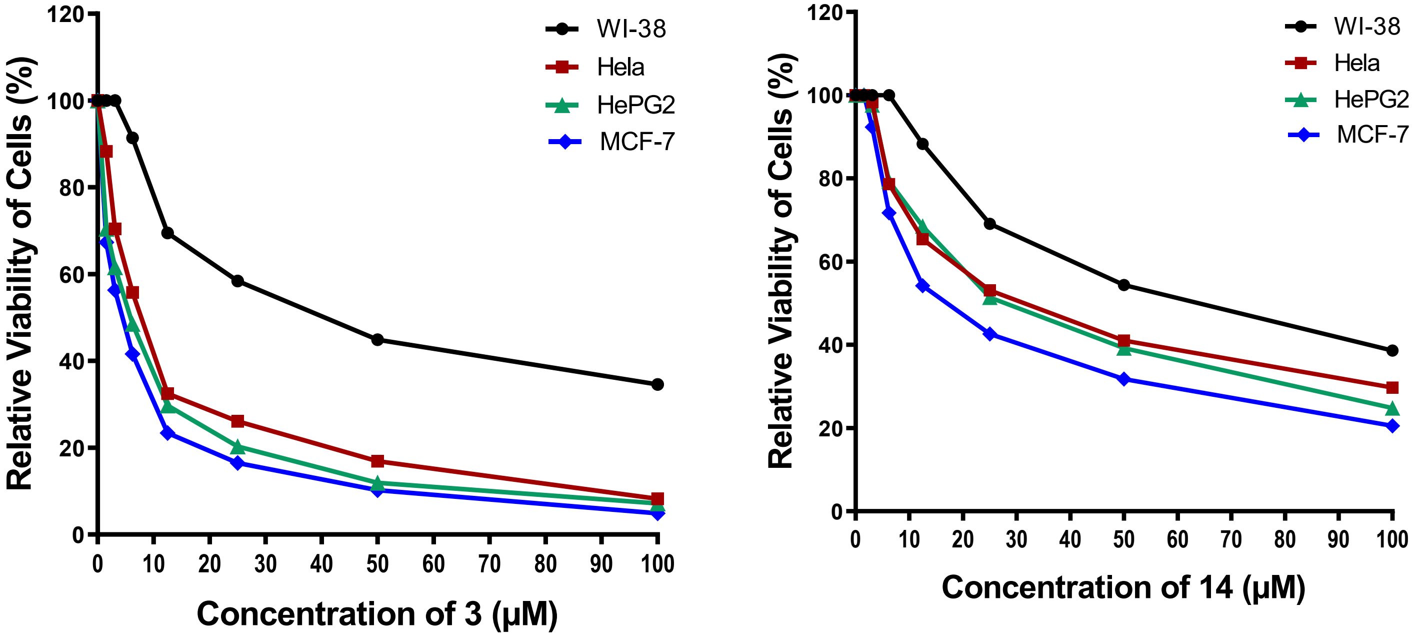

Figure S48. Dose-response IC_{50} curves of compounds **1–14**, Doxorubicin (Dox), and Sorafenib (Sor) against Hela, HepG-2, and MCF-7cell lines against normal WI-38 cell lines. % cell viability was plotted versus log concentration (μM), and IC_{50} values were derived using GraphPad Prism (non-linear regression, n = 3, mean ± SD).

Research Ethics Committee
REC-FOPCU

كلية الصيدلة جامعة القاهرة
FACULTY OF PHARMACY
CAIRO UNIVERSITY

Initial Approval for Experimental Studies

Serial number of the protocol: MI (4080)

Name of the Researchers: Mariam Hassan

Title of the research:

Studying the antimicrobial activity of two chalcone derivatives using murine skin infection model

Approval Valid from 24/11/2025

This is to certify that the research ethics committee for experimental and clinical studies at Faculty of Pharmacy Cairo University-Cairo-Egypt has approved your research protocol.

Please be aware that study conduction will be monitored by the REC.

Any changes in the study must receive review and approval prior to implementation unless the change is unnecessary for the safety of the experiment.

This is an initial approval. The final approval sheet will be provided upon the completion of your research project.

Chair of the Committee

Prof. Dr. Hala F. Zaki

Dean of Faculty of Pharmacy Cairo University

Prof. Dr. Ahmed Hassan Elshafeey

Prof. Dr. Ahmed Hassan Elshafeey

Figure S49. Initial ethical approval certificate issued by the Research Ethics Committee for Experimental and Clinical Studies, Faculty of Pharmacy, Cairo University (REC-FOPCU), approving the in vivo murine experimental study under protocol number MI (4080).
